# Fluorescence-illuminated diffraction tomography using explicit neural fields

**DOI:** 10.1038/s41467-026-78326-x

**Published:** 2026-10-10

**Authors:** Renzhi He, Yucheng Li, Junjie Chen, Truc M. Dinh, Charlotte R. Stevens, Aidan E. Gilchrist, Yi Xue

**Affiliations:** Department of Biomedical Engineering, University of California, Davis, Davis, CA USA

**Keywords:** Multiphoton microscopy, Imaging and sensing, Biophotonics

## Abstract

Multimodal fluorescence and phase imaging provides complementary information about biological samples. However, existing simultaneous fluorescence-phase techniques primarily operate in transmission mode and are limited to thin specimens, restricting their use in thick tissues and in vivo applications. Although multiphoton microscopy enables deep-tissue fluorescence imaging, combining it with three-dimensional refractive-index (RI) imaging remains challenging because few methods can reconstruct the RI of bulky, label-free tissues in reflection mode at subcellular resolution. Here, we introduce fluorescence-illuminated diffraction tomography (FDT), which reconstructs the 3D RI of label-free and fluorescence-labeled objects from diffracted fluorescence images acquired in reflection mode under two-photon excitation. FDT uses the transport of intensity equation and a self-supervised neural network based on explicit neural fields. Compared with state-of-the-art implicit neural fields, this approach improves computational speed, reconstruction accuracy, and interpretability. We demonstrate FDT in two experimental regimes: reconstructing the 3D RI of label-free tissues up to 300 *μ*m thick placed on a fluorescent substrate, and reconstructing the 3D RI of fluorescence-labeled, 3D-cultured cells without transmission-side access using fluorescence emitted by the cells themselves. FDT establishes a foundation for accessible reflection-mode multimodal fluorescence-phase imaging in complex biological systems.

## Abstract (web summary)

Fluorescence-illuminated diffraction tomography uses fluorescence within a sample to reconstruct label-free 3D refractive index, enabling reflection-mode fluorescence-phase imaging in thick samples without transmission access.

## Introduction

Multimodal microscopy for fluorescence and phase imaging provides distinct and complementary signals of biological samples^1–13^. Fluorescence microscopy provides molecule-specific information of the sample by imaging intrinsic or exogenous fluorescence, while quantitative phase microscopy measures the phase or refractive index (RI) of the sample, which can be used as a biomarker for cell biomechanical properties and mass transportation. Most multimodal microscopy techniques for fluorescence and phase imaging are in transmission mode and designed for imaging thin transparent samples^1–11^. In these techniques, 3D RI is reconstructed by optical diffraction tomography (ODT)^14,15^ or intensity diffraction tomography (IDT)^16,17^, while fluorescence images are acquired by widefield illumination^1–9^. ODT relies on interferometric measurements, whereas IDT reconstructs phase and RI from intensity-only images based on the transport-of-intensity equation (TIE) and related models^16,18–20^. TIE relates the transverse phase distribution to the axial derivative of intensity, enabling quantitative phase reconstruction from intensity-only measurements acquired at multiple defocus planes without interferometry. In biological samples, scattering is predominantly forward-directed, making transmission detection advantageous for phase/RI imaging; by contrast, reflection-mode phase/RI imaging must rely on weak backscattered signals, often with reduced signal-to-noise ratio (SNR). On the other hand, fluorescence emission is isotropic with no preference for forward or backward detection.

However, the transmission geometry of these multimodal fluorescence-phase microscopy limits their applications to imaging bulky tissue or in vivo animals, where transmitted photons are very few or the transmission side of the sample is not accessible. As the thickness of tissue increases, the number of photons propagating through the bulky tissue decreases exponentially, and the number of fluorescence or back-scattered photons after propagating back through the bulky sample is even fewer. The second challenge for multimodal fluorescence-phase imaging of bulky tissue is optical sectioning to distinguish signals from a specific deep layer while suppressing background from other depths. For reflection-mode phase imaging, optical sectioning ability has been achieved by using low-coherence light sources^21–25^, confocal detection^26^, interference^22,27,28^, and computational reconstruction^29^, sometimes in combination. Most of these reflection-mode phase imaging measures intrinsic backscattered light from biological samples^21,22,24–28^, while some enhance backscattered signal using a reflective substrate^23,29^, which is incompatible with in vivo imaging due to the need for transmission-side access. Moreover, only a subset of reflection-mode methods provide quantitative 3D RI^25,29^, which are able to decompose the spatial variance of 3D RI and optical path length (i.e., 3D morphology) from phase. For fluorescence microscopy, optical sectioning ability has been achieved by confocal detection^30^, multiphoton excitation^31^, and structured illumination^32^. The third challenge is that these reflection-mode phase imaging techniques are incompatible with simultaneous fluorescence imaging on the same setup, because differences in illumination sources could cause severe crosstalk if both are turned on concurrently unless they are spectrally well separated. Furthermore, the illumination schemes (i.e., widefield, structured illumination, or point scanning) and acquisition modalities (i.e., single-shot, multi-image tomography, or *z*-stack acquisition) used in reflection-mode phase imaging differ from those in standard fluorescence microscopy, further hindering simultaneous fluorescence-phase imaging.

Achieving simultaneous fluorescence and 3D RI imaging in reflection geometry, therefore, requires a diffraction-tomography technique that enables quantitative 3D RI reconstruction in bulky, scattering specimens without transmission-side access, while remaining fully compatible with fluorescence microscopy in terms of excitation, detection, and acquisition. Our recent work, bi-functional refractive index and fluorescence (BRIEF) microscopy^12^, is an IDT-based approach in which both fluorescence and 3D RI are decoded from the same set of diffracted fluorescence images acquired in epi (reflection) geometry. Instead of sequentially capturing fluorescence and phase images of labeled and label-free structures, BRIEF enables truly simultaneous fluorescence-RI imaging. BRIEF first sequentially excites sparsely-distributed fluorescent beads as internal illumination sources within the sample. As fluorescence propagates through label-free tissue, it encodes the tissue’s heterogeneous RI in the resulting diffracted fluorescence images. This process is modeled using the well-established multi-slice model with Born approximation^33–36^. BRIEF reconstructs 3D RI by solving the inverse problem with a conventional gradient-based optimization^37^, and reconstructs fluorescence bead images by localization from defocused fluorescence images similar to super-resolution localization microscopy^38,39^. BRIEF establishes a proof-of-concept for simultaneous, multimodal fluorescence and RI imaging of thin, transparent samples with sparsely-distributed fluorescent beads.

However, applying BRIEF to image bulky tissue with dense fluorescence expression introduces substantial challenges in both hardware and software. First, to remove the requirement for sparsely distributed fluorescent beads and extend BRIEF to arbitrary fluorescence shapes and distributions, the optical hardware must shift from relying on fluorescent beads to generating a diffraction-limited excitation focus that produces an effective point-source fluorescence illuminator at any labeled location within the sample. Second, quantitative 3D RI recovery in thick specimens demands improved axial resolution and *z*-sectioning, which in turn requires hundreds of measurements over a wide range of illumination positions/angles to more fully fill the optical transfer function (OTF) and suppress aliasing artifacts. This shift to substantially larger datasets makes computational efficiency a central limitation: the conventional gradient-descent optimization used in BRIEF requires hours for tens of images and becomes impractical for the hundreds of measurements needed for adequate OTF coverage. Finally, multiple scattering of fluorescence in bulky tissue reduces the SNR of diffracted fluorescence images and increases the complexity of the inverse problem. Together, these constraints call for a reconstruction algorithm to efficiently process the large dataset, jointly estimate fluorescence illumination positions, account for the partially coherent/incoherent nature of fluorescence, and process low SNR images.

To tackle these challenges, we developed fluorescence-illuminated diffraction tomography (FDT) as an IDT technique that reconstructs the 3D RI of bulky tissue from diffracted two-photon fluorescence images acquired in epi (reflection) geometry using an explicit neural-field representation coupled to a multi-slice beam-propagation model. FDT uses two-photon excitation to generate a diffraction-limited fluorescence point source within the specimen; emitted fluorescence then propagates back toward the objective and is diffracted by RI heterogeneity before being recorded at a defocused plane. By scanning the excitation focus to hundreds of positions, FDT acquires diverse perspective views that enable tomographic recovery of 3D RI. In reconstruction, we model fluorescence propagation and multiple scattering using the well-established multi-slice model with Born approximation^12,33–36^, while representing the unknown 3D RI using explicit neural fields to efficiently solve the inverse problem. Neural fields (often implemented as coordinate-based multilayer perceptrons (MLP), i.e., implicit neural fields) have recently enabled 3D scene reconstruction from multi-view measurements^40^ and have been adapted to microscopy^41–45^, including IDT-based RI reconstruction^46^. More recently, explicit neural fields have been introduced to represent 3D scenes using grid-based structures augmented by lightweight neural networks, substantially improving computational efficiency compared with implicit neural fields^47^. For FDT, explicit neural fields offer three practical advantages over implicit MLP-based representations:

1. Explicit neural fields provide a locally parameterized, multi-voxel representation of the RI volume, whereas implicit neural fields encode the entire volume in a global coordinate MLP. Because RI in biological specimens varies predominantly locally, a local parameterization improves numerical conditioning and yields more stable first-order or Gauss-Newton-style updates than a global MLP, whose parameters can couple distant spatial locations.

2. Explicit neural fields allow physically meaningful regularization to be applied directly to the reconstructed RI volume (e.g., voxel-wise or patch-wise priors). Applying regularization in the spatial domain improves robustness to low-SNR measurements. In contrast, enforcing comparable priors on implicit MLP weights is indirect and typically less effective.

3. Explicit neural fields enable faster and more scalable optimization: rendering and updates can be parallelized on the underlying grid, whereas implicit neural fields require repeated backpropagation through a deep network for each coordinate query. As a result, explicit representations can reduce reconstruction time from hours to minutes in our setting, which gives explicit neural fields good scalability for processing large datasets and resolving RI in bulky samples.

Furthermore, we upgrade the explicit neural fields with three key innovations, including a coarse-to-fine strategy for alias-free 3D reconstruction, a self-calibration of illumination sources for precise reconstruction, and a partially coherent mask as a weighting factor to improve convergence robustness. These innovations are explained in detail in the section “Model architecture of FDT for RI reconstruction”.

We first validate FDT through simulations and experiments using a 3D UCDavis pattern and silica micro-beads with known RI. We then quantitatively compare FDT using explicit neural fields to FDT using implicit neural fields by reconstructing the 3D RI of silica micro-beads and live label-free Madin-Darby Canine Kidney (MDCK) cells (~ 40 μm thick) on a fluorescence substrate from experimentally acquired images. Next, we demonstrate 3D RI reconstruction of bulky, 3D-cultured label-free bovine myotubes on a fluorescent substrate over a volume up to ~ 300 μm thick with cellular resolution. Finally, we reconstruct the 3D RI of 3D-cultured fluorescence-labeled HEK cells at a depth of ~ 70 μm without a fluorescent substrate, using fluorescence originating from the cells themselves and requiring no transmission-side access. By integrating fluorescence excitation with IDT, FDT provides an approach for reconstructing the 3D RI of bulky tissue from fluorescence images. FDT is a critical step toward multimodal fluorescence and phase/RI microscopy for bulky tissue and ultimately for in vivo imaging in the future.

## Results

### Optical setup and fluorescence-illuminator characterization for FDT

**Fig. 1:**
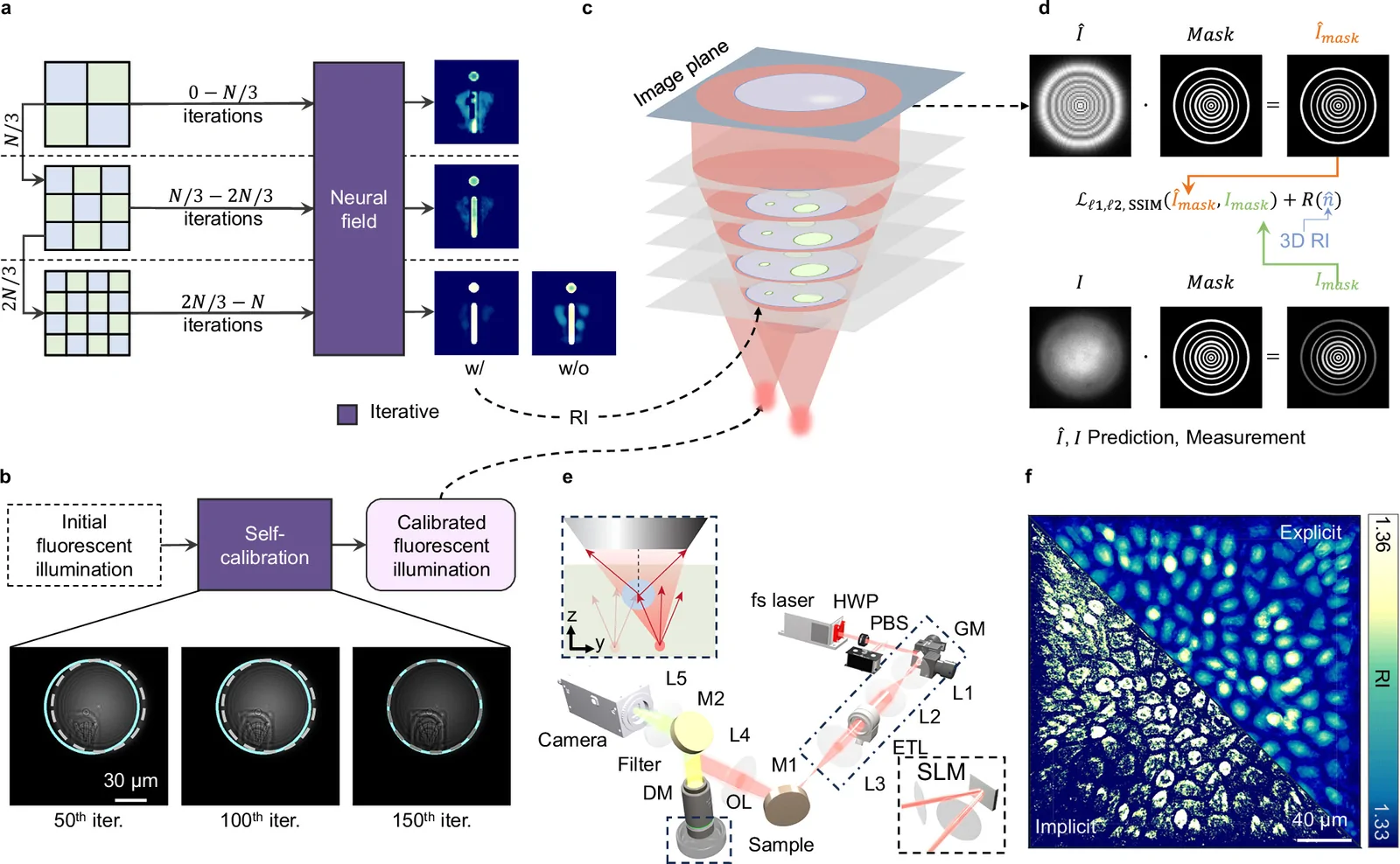
Overview of FDT using explicit neural fields. **a–d** Reconstructing 3D refractive index (RI) from fluorescence images consists of four key components. **a** The coarse-to-fine structure represents the unknown RI ($\hat{n}$) with explicit neural fields and resolves it through three stages with increasing resolution as the number of iterations (*N*) increases to mitigate aliasing. **b** Self-calibration adjusts the parameters to accurately model the fluorescent illumination for each measurement. These variables are then set as iterative parameters and optimized during the training process. **c** The rendering equation is based on a differential multi-slice model, which takes the RI from (**a**) (shown as gray slices) and the fluorescent illumination sources from (**b**) (shown as two red spots) as inputs. The model calculates the light field as it is modulated by the heterogeneous RI on each slice using the Born approximation. Fresnel propagation is used to calculate light propagation between slices. **d** A partially coherent light mask (*Mask*) is applied to both the predicted ($\hat{I}$) and measured images (*I*) to reduce the model mismatch. The masked images (${\hat{I}}_{mask},{I}_{mask}$) are used to calculate the loss function (${\mathcal{L}}$), incorporating *ℓ*_1_, *ℓ*_2_, Structural Similarity Index Measure (SSIM), and regularization terms ($R(\hat{n})$). **e** The optical diagram of FDT. A femtosecond (fs) laser is used for two-photon excitation. A half-wave plate (HWP) and a polarizing beam splitter (PBS) are used to adjust the laser power. A 2-axis galvo mirror (GM) and the electrically tunable lens (ETL) on the conjugate back aperture enables random access to any 3D location. Excitation light is focused by an objective lens (OL) to focus on the sample. Diffracted fluorescence is captured on a defocused plane by a camera in reflection geometry. L1–L5 lenses, M1 mirror, DM dichroic mirror. **f** Comparison of the reconstructed 3D RI of live MDCK cells using FDT with (left) implicit neural fields representations and (right) explicit neural fields representations (ours), respectively. The explicit neural fields achieve significantly improved reconstruction quality.

The excitation path of the optical setup for FDT is identical to that of a standard point-scanning two-photon microscope (Fig. 1e). A femtosecond pulsed laser beam is collimated and scanned laterally by galvanometer mirrors and axially by an electrically tunable lens. Because FDT acquires each image with a 20–300 ms exposure time, the galvanometer mirrors and the electrically tunable lens can be functionally replaced by a spatial light modulator for 3D beam steering, as both approaches provide equivalent control of the excitation focus over the image acquisition time (Fig. 1e, inset). At each scanning position, the beam is focused to a diffraction-limited spot after the objective lens (20×, *NA*_*obj*_ = 1.0) and excites fluorescence via a two-photon process, a nonlinear process in which the fluorescence intensity scales quadratically with the excitation intensity. We experimentally measured the point-spread function (PSF) of the two-photon excitation focus at a 900 nm excitation wavelength using 175 nm-diameter green fluorescent microspheres. The measured PSF is 1.16 × 1.16 × 2.2 μm^3^ (Supplementary Fig. 1), close to the theoretical diffraction limit of 0.55 × 0.55 × 1.8 μm^3^. Notably, these fluorescent microspheres are used only for two-photon PSF characterization, not for RI imaging. Because the imaging system acts as a low-pass filter, the measured PSF provides an upper bound on the size of the fluorescence sources. Accordingly, the actual size of the fluorescence sources can be smaller than the measured PSF. Under the standard definition of spatial coherence, a source whose effective extent is at or below the diffraction limit is spatially coherent across the collection pupil and can be modeled as emitting a well-defined wavefront. We therefore model the fluorescence source excited by each two-photon excitation focus as a single effective point emitter, rather than treating individual fluorescent molecules within the focus volume as independent point sources. For the depth range explored in this work (≤300 μm in 3D cultured tissue), scattering of the near-infrared excitation light is negligible^48–52^. Therefore, the two-photon-excited fluorescence source can be treated as a partially coherent point source within the sample, regardless of the shape and distribution of the fluorescence labeling.

Unlike the near-infrared excitation light, the emitted fluorescence lies in the visible band and undergoes multiple scattering as it propagates through bulky tissue toward the objective. We leverage this scattering of fluorescence to sense 3D RI: as the fluorescence field propagates through the tissue, heterogeneous RI diffracts the fluorescence field, which is converted into measurable intensity variations on a defocused plane relative to the fluorescence sources (Fig. 1c and e, insert). We model this process using the well-established multi-slice multiple-scattering framework under the Born approximation^12,33–36^ (see next Section). Accordingly, the diffracted fluorescence is recorded in epi (reflection) geometry by a camera positioned at a defocused plane (Fig. 1c and e), rather than imaging the excitation focus as in conventional two-photon microscopy. Only fluorescence is detected and the backscattered excitation light is blocked by a color filter. The defocused, diffracted fluorescence intensity image is the raw measurements for RI reconstruction within a TIE-based IDT framework^18–20^. By scanning the two-photon focus to multiple positions, FDT acquires diverse perspective views of the same volume for tomographic recovery of the 3D RI.

The imaging parameters of the defocused, diffracted fluorescence intensity images are directly related to 3D RI recovery (Supplementary Fig. 2). First, the full-frame size (i.e., detection field-of-view (FOV)) is 780 × 780 μm^2^, consisting of 2400 × 2400 pixels (0.33 μm/pixel), set by the camera sensor and detection magnification. Within the detection FOV, the fluorescence-illuminated region is circular (Supplementary Fig. 3), and its diameter (*D*, Supplementary Fig. 2a, b) is determined by the objective NA (*NA*_*obj*_) and the axial separation between the fluorescence source and the detection plane (*Z*, Supplementary Fig. 2b), which varies with source position. For fixed *NA*_*obj*_, increasing *Z* increases *D* but reduces the raw measurement SNR because the same fluorescence power is distributed over a larger area. It also increases defocus for structures far from the image plane, which appear blurred in the raw measurements and consequently reduces the effective resolution. We experimentally evaluate the dependence of SNR and RI reconstruction in a later section. Because the illuminated regions are smaller than the detection FOV and remain near its center, vignetting is negligible in FDT (Supplementary Fig. 3). We define the reconstruction FOV as the union of all illuminated regions across measurements, which depends on both *D* and the maximum lateral separation between fluorescence sources (*L*, Supplementary Fig. 2a, b).

Second, the number of raw measurements is critical because each measurement fills only a subset of the OTF (Supplementary Fig. 2c). Increasing the number of measurements improves OTF coverage and suppresses aliasing artifacts, at the cost of increased acquisition time and computational complexity. The center of the reconstruction FOV is typically measured more times than the periphery (Supplementary Fig. 2a). Denser excitation scanning increases the number of measurements but also lengthens acquisition. For a fixed number of measurements, more uniformly distributed perspectives yield more uniform and less biased OTF filling. We experimentally explore these effects in the section “Evaluation with experimental data of biological samples”. All imaging parameters are summarized in Supplementary Table 1.

### Model architecture of FDT for RI reconstruction

FDT reconstructs 3D RI from diffracted fluorescence images by combining an explicit neural field representation of the RI volume with a multi-slice model with Born approximation (Fig. 1a–d). The multi-slice model serves as the forward renderer: given an estimated RI volume and fluorescence-source parameters, it predicts the diffracted fluorescence images, consistent with prior IDT formulations based on the same framework^12^. The key computational advance of FDT is an inverse-problem solver that jointly estimates the unknown 3D RI and fluorescence-source parameters directly from the measured diffracted fluorescence images. First, we represent the unknown RI volume with a learnable 3D grid (an explicit neural field; Supplementary Fig. 5), which directly parameterizes the RI at voxel-level granularity. To improve reconstruction fidelity and avoid aliasing, we introduce a coarse-to-fine strategy that progressively increases the grid resolution over iterations (“Methods” “Differential multi-slice model for 3D rendering”). After coarse-to-fine refinement, the final lateral grid spacing is set to 0.33 μm, matching with the camera sampling, while the axial grid spacing is chosen between 0.5 and 12.5 μm, depending on sample thickness (Supplementary Table 1). This coarse-to-fine strategy improves the reconstruction accuracy by avoiding aliasing, as shown in the reconstructed 3D RI with and without the coarse-to-fine process from the simulated UCDavis object (Fig. 1a, see next section for details). The result (letter i) shows the coarse-to-fine process successfully solves both low and high spatial frequency components, compared to the aliasing result without the coarse-to-fine process. It improves the mean-squared error (MSE) between the reconstructed RI and the ground-truth RI from 1.84 × 10^−3^ to 1.53 × 10^−3^ (Supplementary Table 4).

Next, we develop a self-calibration method (Fig. 1b) to estimate fluorescence-source positions, analogous in spirit to illumination-angle calibration in plane-wave IDT. In FDT, the illumination angle is determined by the relative position between the fluorescence source and the phase objects. We treat the source positions as learnable parameters and optimize them jointly with the 3D RI. As the number of iterations increases, the position of illumination sources is fine tuned until the illuminated area (white dashed circle) at the image plane in the predicted fluorescence image completely aligns with the illumination area (blue solid circle) from the measured fluorescence image (Fig. 1b). This procedure localizes fluorescence sources in 3D from defocused fluorescence images and can also be used to reconstruct 3D fluorescence distributions, as in our prior work for multimodal fluorescence-RI imaging ^12^. In simulations of the UCDavis object, self-calibration reduces the RI MSE from 2.2373 × 10^−2^ to 2.9116 × 10^−3^ and improves the structural similarity index measure (SSIM) between predicted and ground-truth fluorescence images from 0.8812 to 0.9658 within 20 min (additional quantitative metrics are summarized in Supplementary Table 4 and Supplementary Fig. 6).

Given the estimated RI and source positions, we predict diffracted fluorescence images using the established multi-slice multiple-scattering model with Born approximation^12,33–36^ (Fig. 1c; equations in “Methods” “Differential multi-slice model for 3D rendering”). The multi-slice model treats the 3D RI of a bulky tissue as a stack of 2D slices that modulates the input fluorescence field from the slice below (the first slice modulates the light field from fluorescent illumination sources)^12^. We model fluorescence sources as quasi-monochromatic point emitters (centered at the fluorescence band after the emission filter). We neglect fluorescence that initially propagates away from the objective and is subsequently redirected back toward the detection path, as this contribution is small compared with the directly collected fluorescence. In this work, absorption is negligible for in vitro samples and the thickness range studied (≤300 μm), but absorption can be incorporated when needed, as shown in Eq. (2). The multi-slice model is embedded into the neural network for joint optimization of the RI volume and fluorescence-source positions. To improve efficiency, we use batch training and schedule the optimization by assigning separate learning rates to the RI and calibration parameters. We activate self-calibration starting from the second coarse-to-fine stage to prioritize RI reconstruction.

To improve convergence speed and robustness, we apply an intensity-based weighting mask (i.e., partially coherent mask) to both predicted and measured images before computing the loss (Fig. 1d and Supplementary Section 5.5). Because the effective fluorescence source size is comparable to or slightly larger than the diffraction limit (upper bound 1.16 × 1.16 × 2.2 μm^3^ from the measured two-photon PSF; Supplementary Fig. 1), the fluorescence source is a partially coherent point source. The detected intensity can be approximated as an incoherent sum of a dominant central component and weaker, shifted components, $I({\bf{r}})\approx \mathop{\sum }\nolimits_{m=0}^{M}{w}_{m}\,{I}_{0}\,\left({\bf{r}}-\Delta {{\bf{r}}}_{m}\right),{\sum }_{m}{w}_{m}=1,\ {w}_{m}\ge 0$ with *w*_0_ ≫ *w*_*m*_ for *m* > 0, such that $I({\bf{r}})\approx {w}_{0}\,{I}_{0}\,\left({\bf{r}}\right)$. Given this dominance, instead of computing the incoherent sum, we generate a weighting mask to emphasize the high-intensity region associated with the central component while down-weighting low-intensity regions that are more susceptible to noise and model mismatch. Specifically, we generate a binary mask from the predicted coherent illumination pattern at the image plane in the absence of phase objects, apply it to the predicted image $\hat{I}$ and the measured image *I* to obtain the weighted images ${\hat{I}}_{mask}$ and *I*_*mask*_. We then compute *ℓ*_1_, *ℓ*_2_, and SSIM losses between ${\hat{I}}_{mask}$ and *I*_*mask*_ with a total variation (TV) regularizer to the predicted RI along all three dimensions (Fig. 1d; details in Supplementary Section 5.5). We quantify reconstruction fidelity using accumulated forward-model consistency, a well-established metric in IDT^1–9^. This metric quantifies the agreement between experimentally measured and predicted diffracted fluorescence intensity images. Such an evaluation is necessary because the intrinsic heterogeneity of biological specimens precludes direct assessment of absolute RI accuracy and sensitivity. Because hundreds of images are acquired at different illumination positions and angles, the reconstruction is constrained by a large and diverse set of independent measurements. If a single estimated 3D RI distribution consistently reproduces the measurements across all illumination conditions, this demonstrates that the reconstruction is strongly constrained by and consistent with the complete experimental dataset. In the following section, we validate this approach using simulations and silica microspheres with known ground-truth RI values.

To compare our method with the state-of-the-art IDT approaches that use the same multi-slice model but solve the inverse problem with implicit neural fields, we also constructed FDT with implicit neural representations following established methods^40,46^ in which spatial coordinates after positional encoding are mapped to RI values by a 6-layer MLP. We compared the reconstructed RI of MDCK cells from experimentally captured images using these two methods, respectively (Fig. 1f), where FDT with the explicit model shows more accurate reconstruction compared to that reconstructed using the implicit model. More examples are provided in the next section. In summary, our model integrates the multi-slice forward model with the explicit neural-field inverse solver to enable accurate and efficient 3D RI reconstruction from diffracted fluorescence images.

### Model validation with simulations and experiments using UCDavis patterns and microspheres of known RI

**Fig. 2:**
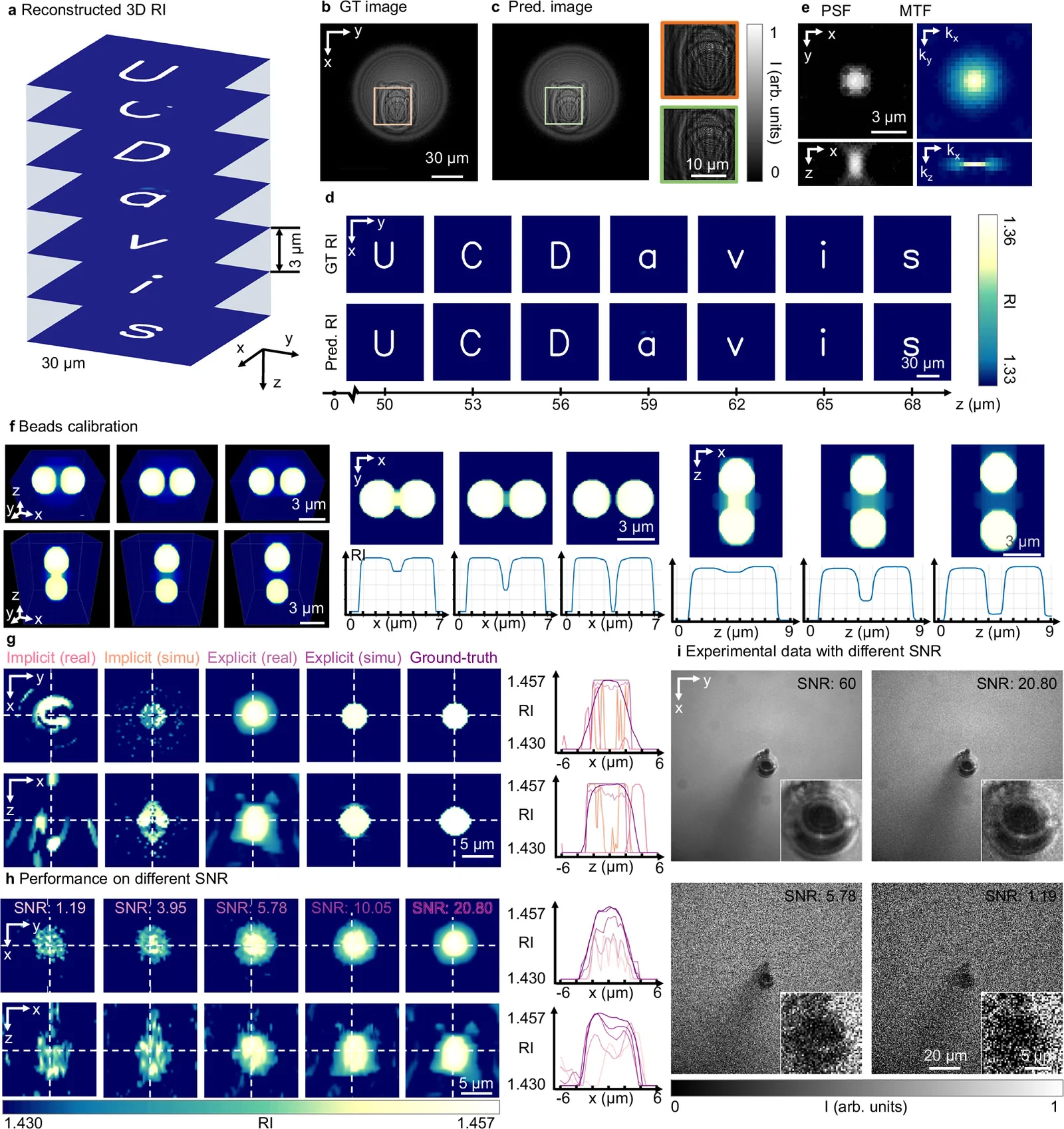
Reconstruction of the 3D RI of a UCDavis pattern and microspheres of known RI in simulation and experiment. Each reconstruction shown was performed once. **a** Reconstructed 3D RI distribution of the UCDavis pattern in 3D. **b** A representative ground-truth (GT) fluorescence measurement. A total of 400 fluorescence measurements were used in the 3D reconstruction. **c** Predicted fluorescence measurement together with zoomed-in views of the regions highlighted by the orange and green boxes. **d** Comparison of the ground-truth RI (top row) and the predicted RI (bottom row) on each *z*-plane, as indicated by the axis below. **e** Simulated point spread function (PSF, left) and modulation transfer function (MTF, right) of the reconstructed 3D microbead using FDT. **f** Volume renderings, cross-sections, and line profiles of the reconstructed 3D RI of two microbeads that are laterally adjacent or axially overlapping at different separations. The lateral separations are 0.1, 0.2, and 0.3 μm, and the axial separations are 0.5, 1, and 1.5 μm. **d–f** Use the same color bar for RI. **g** Comparison of the 3D RI of silica beads reconstructed using explicit and implicit neural fields. From left to right: implicit neural fields (experiment), implicit neural fields (simulation), explicit neural fields (experiment), explicit neural fields (simulation), ground truth (simulation). The dotted lines in the images mark the *x*- and *z*-directions along which the line profiles are plotted. **h** Comparison of the reconstructed 3D RI using forward images at various signal-to-noise ratios (SNR), ranging from 1.19 up to 20.80. The dotted lines again indicate the *x*- and *z*-directions used to extract the line profiles. **i** Forward images of the same beads at different SNR levels. The top-left image (SNR = 60) is used for the reconstruction in (**g**), whereas the other three images are used for the reconstruction in (**h**).

We first validate FDT by reconstructing the 3D RI of a simulated UCDavis pattern as the phase object with known RI (Fig. 2a–d). The ground-truth RI model of the UCDavis pattern consists of 14 layers spaced 3 μm apart, sampled on a 512 × 512 grid with 0.33 μm pixel resolution. Each letter of UCDavis is centered in the odd-numbered layers, while even-numbered layers are homogeneous (Fig. 2d, top row). The RI is set to 1.36 for the letter and 1.33 for the background, approximating the RI of biological cells and culture media^53^. We set 400 illumination sources (a 20 × 20 array with 4.2 μm spacing) 60 μm below the bottom layer of the UCDavis pattern and generated 400 ground-truth fluorescence images using the ground-truth RI under these illuminations (one representative image is shown in Fig. 2b). All images are captured on the same image plane, corresponding to the top layer of the UCDavis pattern. Our model is trained on the 400 ground-truth fluorescence images to reconstruct the 3D RI (Fig. 2a and d, bottom row) and predict the corresponding fluorescence images (one represented predicted image is shown in Fig. 2c), following the procedure described in Fig. 1. The reconstructed RI (Fig. 2a) clearly resolves each letter with sharp edges and no crosstalk between adjacent *z*-layers. The predicted forward image (Fig. 2c; zoomed-in view on the bottom right) closely matches the ground-truth image (Fig. 2b; zoomed-in view on the top right), indicating a high forward-model consistency (MSE = 1.0238 × 10^−3^, SSIM = 0.9814, LPIPS = 0.0078, PSNR = 29.8980). The reconstructed RI of each letter closely matches the ground-truth RI (Fig. 2d) with a MSE of 1.8381 × 10^−3^, a SSIM of 0.9830, a LPIPS of 0.0513, and a PSNR of 27.3563, indicating FDT reconstructs the 3D RI of UCDavis pattern accurately (Supplementary Table 3). We also perform an ablation study to demonstrate the effectiveness of coarse-to-fine strategy and the self-calibration of illumination sources in the reconstruction algorithm. Detailed quantitative evaluation is provided in the Supplementary Table 4.

To quantitatively evaluate the resolution of RI reconstruction using FDT, we next reconstruct the 3D RI of simulated microbeads with known RI. The theoretical lower bounds of the resolution are determined by the objective numerical aperture, *NA*_*obj*_ (*NA*_*obj*_ = 1), and the illumination numerical aperture, *NA*_*illu*_, according to prior work^34,54–56^: Δ*x* = *λ*/[2(*NA*_*obj*_ + *NA*_*illu*_)] in the lateral dimension and $\Delta z=\lambda /(2n-\sqrt{{n}^{2}-N{A}_{obj}^{2}}-\sqrt{{n}^{2}-N{A}_{illu}^{2}})$ in the axial dimension. In FDT, *NA*_*illu*_ is determined by the fluorescence-source positions and the detection plane ($N{A}_{illu}=n\sin ({\theta }_{illu})$, Supplementary Fig. 2). In the experiments, the measured *NA*_*illu*_ ranges from 0.42 to 0.79, resulting in theoretical lower-bound RI resolutions of 0.18–0.22 μm laterally and 0.89–1.22 μm axially. Under the same parameters, the PSF of the reconstructed RI is 0.20 μm laterally and 1.31 μm axially (Fig. 2e, left), indicating that FDT achieves high-resolution RI reconstruction. The corresponding modulation transfer function (MTF) (Fig. 2e, right) shows that FDT fills the missing cone, thereby achieving *z*-sectioning capability, as also demonstrated in the UCDavis result. We further reconstruct the 3D RI of two adjacent 3 μm-diameter microbeads with different lateral and axial separations (Fig. 2f). As the lateral separation between the two microbeads increases from 0.1 to 0.3 μm, the reconstructed RI changes from connected to fully separable. Similarly, for two microbeads overlapping along the axial direction, as their separation increases from 0.5 to 1.5 μm, the reconstructed RI changes from inseparable to separable. These results further demonstrate that FDT enables high-resolution 3D RI reconstruction with good *z*-sectioning ability.

To quantitatively evaluate reconstruction accuracy, we simulated five microbeads with RI contrasts relative to the background ranging from Δ*RI* = 0.01 to 0.05, in increments of 0.01 (Supplementary Fig. 8). We evaluated the reconstruction accuracy using the standard deviation (STD) of the reconstructed voxel-wise Δ*RI*, as well as the root-mean-square error (RMSE) between the ground-truth and reconstructed Δ*RI*. The reconstruction results show that the STD of the reconstructed RI is on the order of 10^−4^ (*n* = 2500 pixels) and that the RMSE remains small when Δ*RI* is small. For example, the reconstructed RI of a microbead with a ground-truth Δ*RI* of 0.02 is 0.0202 ± 5.29 × 10^−4^, indicating accurate reconstruction for a weakly scattering object. The RMSE increases for larger Δ*RI*, indicating that reconstruction accuracy decreases for strongly scattering objects. Detailed data are summarized in Supplementary Table 7. Together, these results show that FDT achieves high-accuracy 3D RI reconstruction for weakly scattering objects.

To further validate FDT under realistic conditions, we next reconstruct the 3D RI of silica beads (RI = 1.4570 at 635 nm) embedded in PDMS (RI = 1.4295 at 635 nm) on a microscope slide pre-coated with a homogeneous fluorescence layer (central wavelength 635 nm), located 140 μm beneath the top of the sample (Supplementary Fig. 4a). Using 419 experimentally captured fluorescence images, we reconstructed the 3D RI of a 5 μm silica bead with both explicit and implicit neural field models and compared the results to simulations (Fig. 2g). Reconstructing silica beads using FDT is challenging due to the large RI contrast and the self-focusing effects induced by the bead. Nonetheless, the results show explicit neural fields outperform implicit ones in both accuracy and speed. With experimentally captured images (SNR = 60, Fig. 2i, top left; Supplementary Fig. 3a), the RI reconstructed by the explicit neural fields exhibits fewer artifacts than that reconstructed by the implicit neural fields (Fig. 2g, 1st vs 3rd column). The reconstructed bead appears elongated along the axial direction with slight sidelobes arise mainly from the limited frequency support of the OTF in the *k*_*z*_ direction from finite number of experimental measurements. Using simulated data, explicit neural fields reconstruct RI distributions nearly identical to ground truth (Fig. 2g, 4th vs. 5th column) from 600 diffracted fluorescence images, whereas the implicit model shows aliasing and anisotropy (Fig. 2g, 2nd column). Quantitative comparison further shows that the explicit neural-field model reconstructs the RI more accurately than the implicit neural-field model, improving the MSE from 6.1572 × 10^−3^ to 5.6387 × 10^−4^ and the SSIM from 0.8724 to 0.9834 (additional metrics are listed in Supplementary Table 5). Line profiles across *x* and *z* (Fig. 2g, 6th column) confirm that explicit neural fields yield sharper bead edges and more homogeneous RI within the beads compared with implicit neural fields, indicating improved recovery of both high- and low-spatial frequency RI features. Furthermore, with the same forward model, the same raw measurements, and the same RI volume (i.e., identical voxel count), replacing the implicit-neural-field inverse solver with the explicit-neural-field solver reduces the reconstruction time from 3 h to 50 min (with 90% accuracy achieved in just 20 min) on the same computer (see Supplementary Section 5.5). In summary, these results quantitatively demonstrate that FDT with explicit neural fields enables accurate, efficient, and near-isotropic 3D RI reconstruction, significantly improving performance over implicit neural fields.

To evaluate how the SNR of diffracted fluorescence images affects reconstruction quality, we test the robustness of FDT with explicit neural fields using experimental images across a range of noise levels (SNR 1.19–20, Fig. 2i), generated by adding Gaussian noise to a high SNR image (SNR = 60, Fig. 2i). The result shows RI reconstruction accuracy improves significantly with increasing SNR (Fig. 2h). When the SNR is low, the reconstructed RI shows overfitting to the high-frequency noise present in the low-SNR images, resulting in loss of low-frequency components and domination of speckle-like patterns. When the SNR is high, the reconstructed RI preserves both low- and high-frequency components but with slight elongation in the axial direction as discussed above. Quantitative analysis shows both the RMSE and STD decrease substantially as the SNR increases (Supplementary Table 6 and Supplementary Fig. 7). For example, when the SNR is low (SNR = 1.19), the reconstructed RI of the silica bead is 1.4442 ± 0.0048, whereas at high SNR (SNR = 10.05), the reconstructed RI of the silica bead is 1.4584 ± 0.00128 (ground-truth RI: 1.4570), indicating the reconstruction error is substantially reduced. Notably, even under severe noise, explicit neural fields outperform implicit ones with high SNR images (Fig. 2g), providing higher overall fidelity. These results confirm that, despite relying on relatively dim fluorescence images in epi (reflection) geometry compared to standard ODT using bright field transmission images, FDT with explicit neural fields can achieve accurate RI reconstruction, offering an advancement in integrating fluorescence imaging with RI reconstruction.

### Evaluation with experimental data of biological samples

In this section, we evaluate FDT by reconstructing the 3D RI of biological samples from experimentally acquired diffracted fluorescence images. First, we demonstrate that FDT quantitatively reconstructs the RI of label-free, live MDCK cells on a fluorescence substrate and resolves RI differences between live and dead MDCK cells in the same volume. Second, we demonstrate 3D RI reconstruction of a label-free, about 300 μm-thick 3D cultured bovine myotube on a fluorescence substrate, highlighting FDT’s ability to reconstruct the RI of bulky tissue. Third, we reconstruct the 3D RI of fluorescence-labeled HEK cells without a fluorescence substrate by using the fluorescence originating from the cells as internal illuminators, demonstrating epi (reflection) geometry RI reconstruction without transmission-side access. Detailed information on sample preparation and the optical setup is provided in Supplementary Section 5, and imaging/reconstruction parameters are summarized in Supplementary Table 1.

#### 3D RI reconstruction and analysis of live MDCK cells

**Fig. 3:**
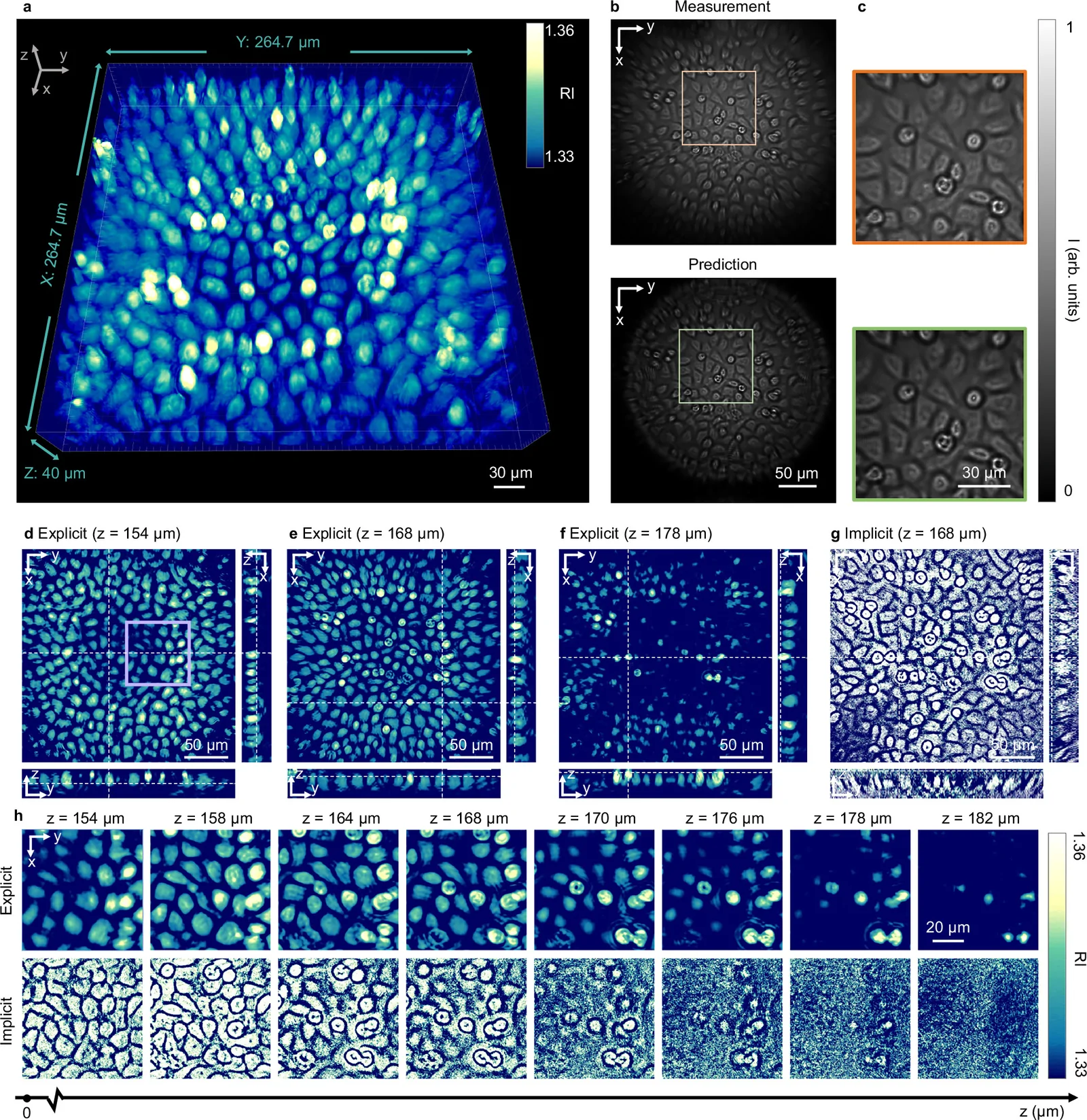
3D RI reconstruction of a thin layer of live MDCK cell sample. The reconstruction shown was performed once on this sample and is presented as a demonstration of FDT in live cells. **a** 3D visualization of the RI distribution of MDCK cells within a 264.7 × 264.7 × 40 μm^3^ volume. The RI of the cells ranges between 1.33 and 1.36. **b** Comparison of the measured (top) and predicted (bottom) images. **c** Zoomed-in views of the highlighted region: the measured image (top) and the predicted image (bottom). **d–f** Cross-sectional views of the RI distribution of MDCK cells on three representative planes, showing optical sectioning ability and high 3D resolution. **g** Cross-sectional RI slices reconstructed using the implicit neural fields at the same location with (**e**) for direct comparison, which shows high-frequency artifacts and severe crosstalk from adjacent planes. **h** Zoomed-in view of the image *z*-stack of cells in the boxed region in (**d**) reconstructed by the explicit model (top row) and the implicit model (bottom row). The images again highlight the optical sectioning and high resolution of FDT using explicit neural fields. See the Supplementary Movies 1–4 for a better 3D visualization.

We first experimentally validate FDT using 2D cultured, label-free live MDCK cells (Fig. 3) above a fluorescence-paint substrate. The fluorescence-paint layer is outside of the petri dish and located about 150 μm below the MDCK cells (Supplementary Fig. 4b). We scan the two-photon focus on the fluorescence paint and acquire one diffracted fluorescence image at each scan position. All fluorescence images are taken on a single image plane slightly above the MDCK cells. A total of 390 fluorescence images are selected for reconstructing the 3D RI of the MDCK cells. The reconstructed RI (Fig. 3a) consists of 20 slices with a sampling grid of 800 × 800 pixels, corresponding to a physical volume of 264.7 × 264.7 × 40 μm^3^. The reconstructed RI ranges from 1.33 to 1.36, demonstrating FDT is capable of quantitatively reconstructing 3D RI over a large FOV. With the reconstructed RI, the predicted diffracted fluorescence images (Fig. 3b, bottom; zoomed-in view in Fig. 3c) closely match the experimentally measured fluorescence images (Fig. 3b, top and Supplementary Fig. 3c) with MSE = 6.2900 × 10^−4^, SSIM = 0.9160, LPIPS = 0.1417, and PSNR = 32.0135 (Supplementary Table 9), indicating an accurate reconstruction of the RI. The representative cross-sectional views of the RI in the *x*–*y*, *x*–*z*, and *y*–*z* planes (Fig. 3d–f) reveal individual cells clearly, highlighting FDT’s ability to achieve high resolution in 3D and *z*-sectioning ability. In the zoomed-in *z*-stack (Fig. 3h), cells can be observed appearing and disappearing. Most cells are viable and attached to the bottom of the dish (Fig. 3e, f), while in the top several layers (Fig. 3h, top row), dead cells shrink, disassemble their focal adhesions, and float above the substrate. The relatively higher RI of dead cells compared to live cells probably indicates intracellular condensation during cell death, and illustrat the sensitivity of RI contrast to cell state.

Moreover, we compare FDT using explicit neural fields with FDT using implicit neural fields (Fig. 3g, h, bottom row). The implicit approach exhibits severe crosstalk from adjacent planes and high-frequency artifacts, consistent with the reconstruction artifacts observed in the silica beads experiment. We also conduct an ablation study to quantitatively evaluate the partially coherent weighting mask by comparing predicted and experimentally measured fluorescence images with and without the mask (Supplementary Fig. 9). Incorporating the weighting mask in the training process improves the MSE from 1.3453 × 10^−3^ to 6.2900 × 10^−4^, the SSIM from 0.8177 to 0.9160, the LPIPS from 0.1776 to 0.1417, and the PSNR from 28.7119 to 32.0135 (Supplementary Table 8). Furthermore, we evaluate how the number and spatial distribution of fluorescence sources affect RI reconstruction (Supplementary Fig. 10). Under uniform random distribution, the reconstruction RI shows less aliasing artifact as the number of fluorescence sources increases (Supplementary Fig. 10a, b, d). For a fixed number of measurements, uniformly distributed source positions yield less distortion than biased sampling (Supplementary Fig. 10c, d). Together, these results experimentally and quantitatively demonstrate FDT enables aliasing-suppressed 3D RI reconstruction with strong *z*-sectioning, high resolution, and high sensitivity.

#### 3D RI reconstruction and analysis of a bulky 3D-cultured bovine myotube

**Fig. 4:**
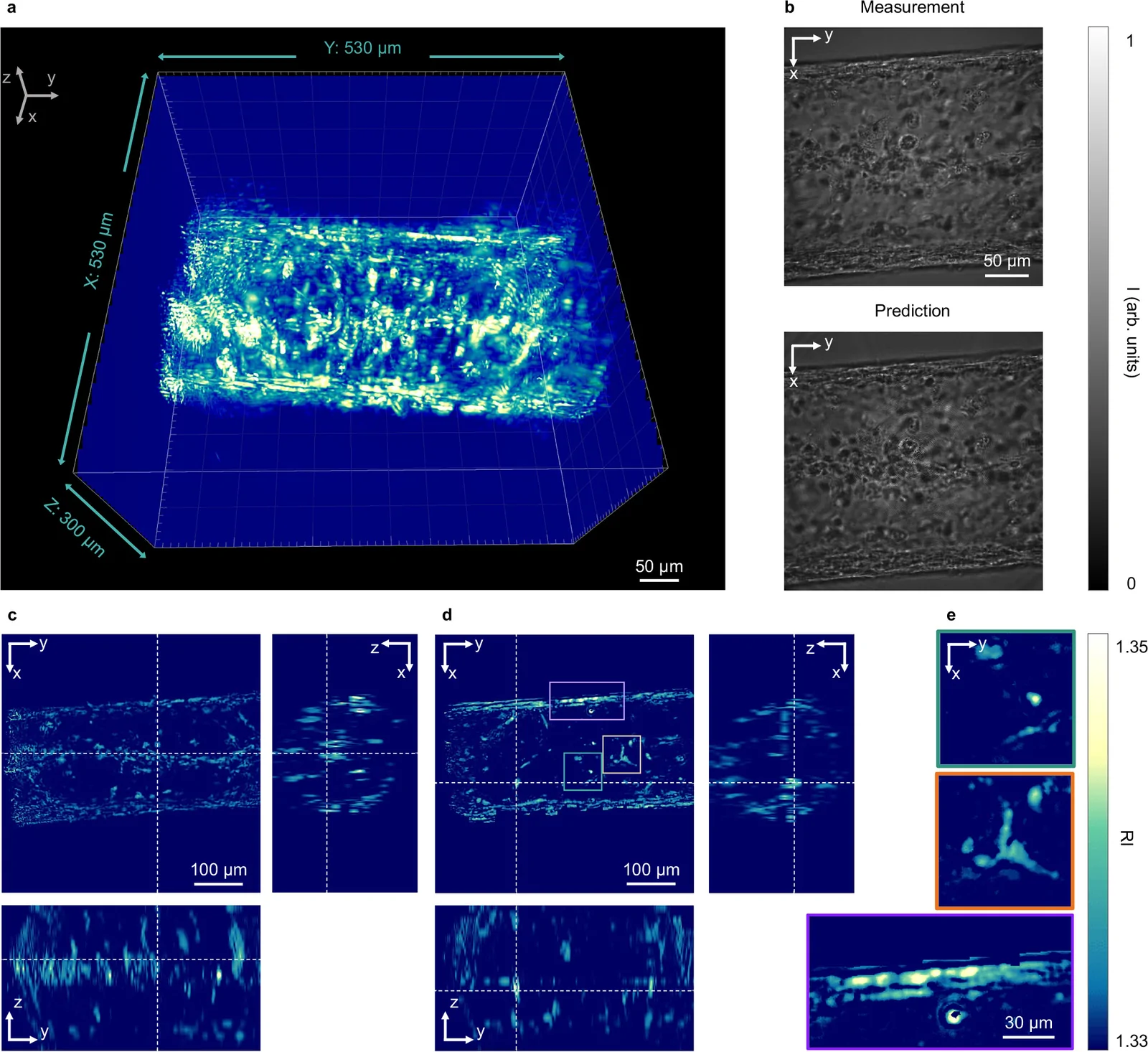
3D RI reconstruction of a 3D cultured bovine myotube. The reconstruction shown was performed once on this sample and is presented as a demonstration of FDT in thick biological tissue. **a** 3D visualization of the RI of 3D cultured bovine myotube within a volume of 530 × 530 × 300 μm^3^. **b** Comparison between the measured (top) and predicted (bottom) images of a representative region. **c**, **d** Cross-sectional views of the reconstructed RI on two representative planes, showing high resolution and optical sectioning ability. **e** Zoomed-in details of the highlighted regions in (**d**), labeled by corresponding colors, showing different morphologies of stem cells in the 3D cultured bovine myotube during proliferation and differentiation. The results indicate that FDT can accurately reconstruct various structures across a wide range of spatial frequencies. See the Supplementary Movies 5 and 6 for a better 3D visualization.

We next evaluate FDT by reconstructing the 3D RI of a bulky 3D-cultured bovine myotube (Fig. 4). Unlike the thin layer of cells discussed in the previous section, the bovine myotube is significantly thicker (about 300 μm, Fig. 4a) and consists of multiple layers of muscle cells. These muscle cells are differentiated from label-free muscle stem cells cultured in 3D. The myotube is placed on a fluorescence-paint substrate located about 90 μm below the bottom of the myotube (Supplementary Fig. 4c). Given that the bovine myotube is much thicker and more complex than the MDCK cells, all diffracted fluorescence images are acquired on a single image plane positioned in the middle of the myotube rather than near the top of the sample to ensure high resolution reconstruction through the whole volume and optimize the SNR of forward images. Correspondingly, the reconstruction process implements a back-propagation operation (Eq. (6)). After assessing the SNR of images, 348 fluorescence images are selected for 3D RI reconstruction in a 530 × 530 × 300 μm^3^ volume, consisting of 24 *z*-slices with a sampling grid of 1024 × 1024 pixels. Individual cells are not distinguishable in the scrambled forward images (Fig. 4b, top and Supplementary Fig. 3c), but appear as bright and dark blurry shadows in the image depending on if they are above or below the image plane. The predicted image (Fig. 4b, bottom) calculated with the reconstructed 3D RI closely matches the experimentally measured image, with MSE = 7.7843 × 10^−3^, SSIM = 0.7361, LPIPS = 0.3024, and PSNR = 22.7127 (Supplementary Table 9). These results indicate strong agreement between the measured data and the forward predictions from the reconstructed 3D RI, supporting the accuracy of the reconstructed RI. Cross-sectional views (Fig. 4c, d) show the reconstructed RI of the 3D cultured bovine myotube on two representative *z*-planes that are 50 μm apart in the entire volume. These orthogonal views clearly reveal the tubular morphology of the sample. The circular cross-section in the *x*–*z* view confirms the integrity of the tube structure, while the clear delineation between the top and bottom of the tube highlights the effectiveness of our method’s *z*-sectioning ability. The distinctive distribution of cells inside the tube on the two different *z*-planes also shows the *z*-sectioning ability of FDT. The reconstruction also achieves high 3D resolution. Individual cells are visible and distinguishable after reconstruction, whereas they are indistinguishable in the forward images.

Our results reveal individual cells with diverse morphological structures and RI during the processes of differentiation and aggregation (Fig. 4e). This demonstrates that FDT not only achieves cellular resolution but also effectively reconstructs structures across both low and high spatial frequencies. The morphology of the muscle cells within the myotube can be categorized into three distinct differentiation stages: individual stem cells exhibiting membrane spreading (Fig. 4e, top; Fig. 4d, green box), interconnected cells forming pre-differentiation clusters within the matrix (Fig. 4e, middle; Fig. 4d, orange box), and elongated, stable muscle cells forming axial connections (Fig. 4e, bottom; Fig. 4d, purple box). A control experiment on a myotube cultured under the same conditions, followed by immunofluorescence staining, corroborates that these morphological transitions track molecular markers of myogenic differentiation (Supplementary Fig. 11). FDT thus links morphological remodeling with quantitative RI changes during differentiation. In our reconstructions, individual stem cells and elongated muscle cells exhibit relatively higher RI compared to the interconnected cells, suggesting RI as a potential label-free biomarker of differentiation state. Together, these results show that FDT resolves cellular structure in thick specimens while quantitatively measuring RI variations associated with cellular state.

#### 3D RI reconstruction of fluorescence-labeled HEK cells without transmission-side access

**Fig. 5:**
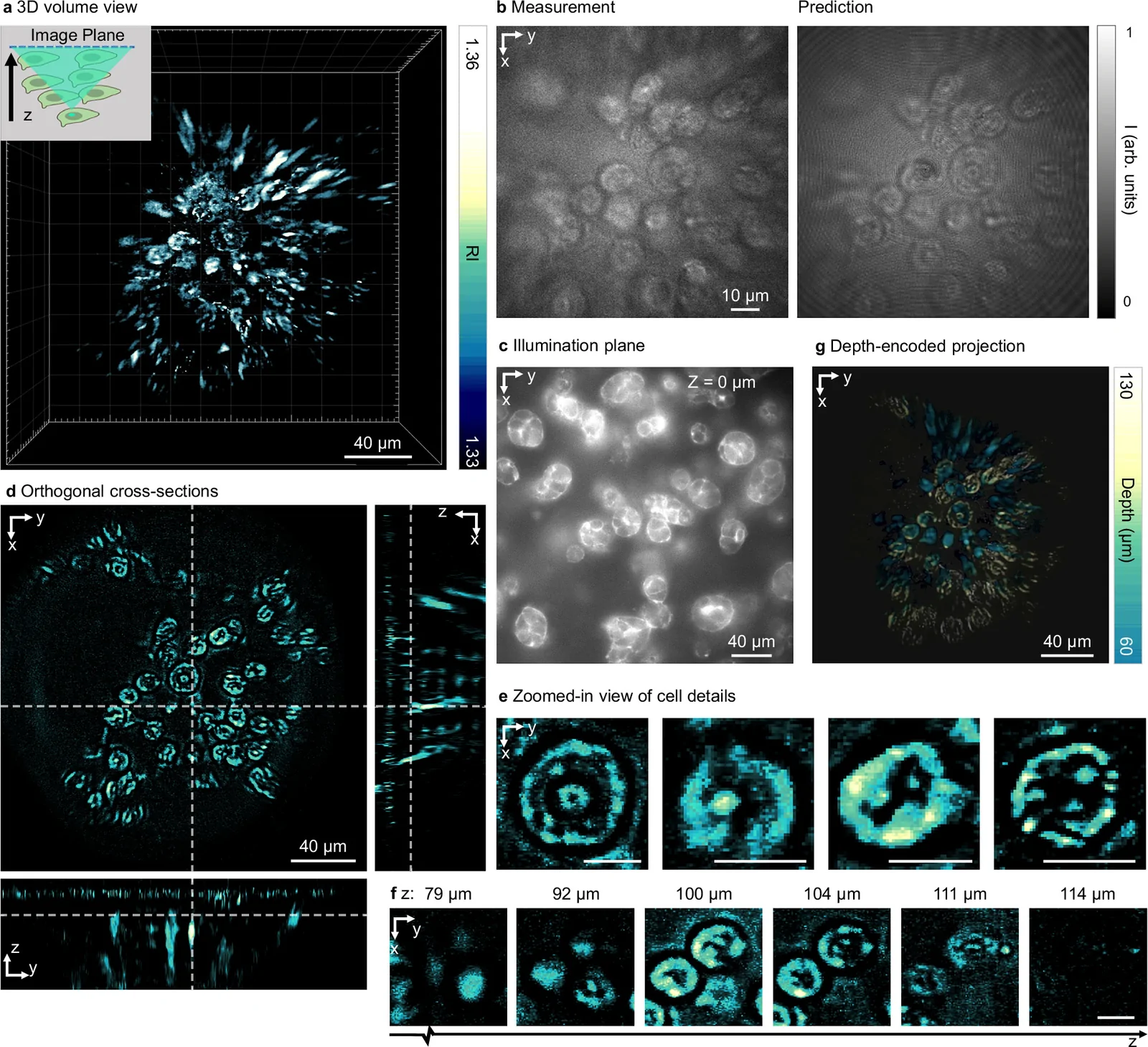
3D RI reconstruction of ActinGreen 488-labeled HEK cells illuminated by fluorescence originating from the sample itself. The reconstruction shown was performed once on this sample and is presented as a demonstration of FDT in 3D cultured cells without transmission-side access. **a** Reconstructed 3D RI of HEK cells over a 198 × 198 × 70 μm^3^ volume. Inset: imaging geometry in which fluorescence-labeled cells serve as internal illuminators; their emitted fluorescence is diffracted by overlying cells and recorded as defocused images near the sample surface (see Supplementary Fig. 4d for details). **b** Representative diffracted fluorescence measurement (left) and corresponding prediction (right). The reconstruction shown in (**a**) was performed once using 282 fluorescence measurements acquired with different fluorescence illuminators within the same sample. **c** One-photon wide-field fluorescence image of HEK cells at *z* = 0 that act as fluorescence illuminators. This image characterizes the fluorescence illuminators used for the reconstruction in (**a**) and was acquired once from this sample. **d** Orthogonal RI cross-sections (*x*–*y*, *x*–*z*, *y*–*z*); dashed lines indicate slice locations. **e** Zoomed-in RI images of representative cells showing clearly resolved membrane-scale features. Scale bar, 10 μm. **f** Zoomed-in RI *z*-stack demonstrating *z*-sectioning by resolving three cells at different depth. Scale bar, 10 μm. **g** Depth-encoded projection of the reconstructed RI volume.

To validate that FDT can reconstruct 3D RI when fluorescence originates within a thick specimen, we applied FDT to HEK293T cells cultured in a 3D Matrigel matrix and labeled with ActinGreen 488 (Fig. 5c and Supplementary Fig. 4d). Unlike the MDCK and bovine myotube experiments above, this measurement does not require access to the transmission side to apply fluorescent paint, making the approach applicable to specimens for which transmission-side access is not available. We used two-photon excitation to generate localized fluorescence within the cells at a depth of 130 μm below the sample surface (Fig. 5c). As fluorescence propagates upward toward the objective, it is diffracted by the RI variations of overlying cells. All diffracted fluorescence images were acquired on a single camera plane near the sample surface (Fig. 5b, left and Supplementary Fig. 3d), and 282 images were selected based on SNR for 3D RI reconstruction.

Reconstructing the HEK-cell RI is more challenging than in the experiments above because the fluorescence images have lower SNR (Supplementary Fig. 3d and Fig. 5b), as ActinGreen-labeled actin is substantially dimmer than fluorescent paint. As shown in Fig. 2, low-SNR measurements can degrade RI reconstruction due to overfitting to noise. To mitigate this effect, we apply an early-stopping strategy: empirically, the model recovers physically consistent RI structure within the first several iterations, whereas prolonged optimization increasingly fits noise and degrades reconstruction quality. We therefore stop the optimization at iteration 60 (out of a maximum of 300), which also reduces reconstruction time.

By leveraging the early-stopping strategy, we reconstructed the 3D RI of HEK cells from low-SNR diffracted fluorescence images over a large volume (Fig. 5a, 198 × 198 × 70 μm^3^, 600 × 600 × 40 voxels). Forward-model consistency supports reconstruction fidelity, which shows the predicted diffracted fluorescence images closely match the experimental measurements with a MSE of 3.5858 × 10^−3^ (Fig. 5b and Supplementary Table 9). Orthogonal cross-sections (Fig. 5d) resolve individual HEK cells with clear, enclosed cell boundaries. Representative zoomed-in *x*–*y* view further reveals cellular features down to ~ 1–2 μm (Fig. 5e), demonstrating high-resolution RI recovery. Moreover, a zoomed-in RI stack distinguishes cells at different axial planes without out-of-plane crosstalk (Fig. 5f). At the volume scale, the depth-encoded projection (Fig. 5g) localizes cells across the full axial extent, highlighting the strong *z*-sectioning of FDT in thick samples. Together, these results demonstrate that FDT can reconstruct 3D RI in thick biological specimens using fluorescence originating from the sample itself, without transmission-side access.

## Discussion

We have developed FDT as a reflection-mode multimodal fluorescence-phase imaging technique. As an IDT technique, FDT reconstructs 3D RI from intensity-only measurements without interferometry, while leveraging two-photon-excited fluorescence as low-coherence internal illumination within bulky tissue, providing a complementary alternative to transmission-mode IDT for specimens in which transmission-side access is unavailable. Two-photon excitation allows us to model the fluorescence source as point-like, even for extended fluorescent objects, because of the near-infrared excitation wavelength and the nonlinear excitation process, while the emitted fluorescence is diffracted (encoded) by heterogeneous RI, enabling us to decode the 3D RI from diffracted fluorescence images. For reconstruction, FDT uses the established multi-slice forward model under the Born approximation to model fluorescence diffraction^12,33–36^, and solves the inverse problem by representing the unknown RI as an explicit neural field jointly optimized with fluorescence-source positions. The optimization is considered converged when the reconstructed RI and fluorescence-source parameters reproduce the measured diffracted fluorescence images through the multislice forward model, as quantified by accumulated forward-model consistency, which evaluates the agreement between the predicted and measured images across all illumination views. We validated that accumulated forward-model consistency reflects RI reconstruction accuracy by reconstructing 3D RI from simulated data and experimental measurements of silica beads with known ground-truth RI values. Together with coarse-to-fine optimization and partially coherent weighting masks, this formulation substantially accelerates reconstruction, enabling the use of many more measurements to improve OTF coverage and thereby achieve high-resolution, high-accuracy, and high-sensitivity RI recovery. We have demonstrated that FDT enables 3D RI reconstruction from diffracted fluorescence images acquired in two separate experimental regimes: (1) label-free tissue within a 530 × 530 × 300 μm^3^ volume placed on a fluorescent substrate and (2) fluorescence-labeled, 3D-cultured HEK cells within a 198 × 198 × 70 μm^3^ volume imaged without transmission-side access, using fluorescence emitted by the cells themselves. In the second regime, the two-photon-excited fluorescence serves not only as an incoherent illumination source for RI reconstruction but also provides spatially localized, molecule-specific information about actin within the cells. This enables fluorescence and RI information from the same specimen to be acquired simultaneously through the same excitation and detection platform, potentially allowing biologists to investigate relationships between fluorescence-labeled structures and surrounding label-free structures. FDT not only opens opportunities for multimodal fluorescence-RI imaging but also enables broad dissemination by repurposing standard two-photon microscopy hardware, with a reconstruction algorithm that can be widely adopted for 3D rendering from multiple views.

Compared to our previous method BRIEF^12^, which uses the same multi-slice forward model but solves the inverse problem with conventional gradient-descent optimization^37^, FDT represents the RI volume with explicit neural fields and scales to ~ 10 × more diffracted fluorescence images. This enables faster and more accurate reconstructions and extends RI imaging to substantially thicker specimens. Furthermore, by leveraging two-photon excitation, FDT overcomes the constraint of requiring sparsely distributed fluorescent beads, allowing broader, more realistic choices of fluorescence expression. We also benchmarked the performance of the explicit model against the implicit model used in the state-of-the-art IDT^46^ under the same multi-slice forward model and the same diffracted fluorescence measurements. Our results demonstrate that the explicit model achieves substantially higher reconstruction fidelity, suppresses high-frequency aliasing artifacts, and reduces computational time. Finally, in contrast to conventional ODT systems that operate in transmission mode, FDT operates in epi (reflection) geometry without transmission-side access, which will facilitate future in vivo imaging applications.

In this work, FDT targets weakly scattering specimens, where the two-photon-excited fluorescence illuminator can be approximated as a point source and fluorescence propagation can be modeled using a multi-slice framework under the Born approximation. Highly scattering regimes in which even the near-infrared two-photon excitation light is strongly scattered (e.g., at depths of 500–1000 μm in the mouse brain^48,49^) are beyond the scope of this study. The largest volume reconstructed here is 530 × 530 × 300 μm^3^ in 3D-cultured bovine myotubes. Extending quantitative RI reconstruction to highly scattering tissues will require advances in both the physical forward model and the computational algorithm.

FDT could be further optimized for broader applications. The current implementation faces memory limitations (and correspondingly runtime limitations), which constrains the maximum number of voxels that can be reconstructed in a single pass. Both memory consumption and per-iteration computational cost scale approximately linearly with the total number of voxels for fixed batch size. Quantitative evaluation of the runtime and peak GPU memory usage is summarized in Supplementary Table 2 of the Supplementary Section 5.5. This challenge may be addressed by developing memory-efficient algorithms to improve the scalability of the approach. Addressing this limitation would significantly enhance the practicality and applicability of FDT in a broad range of biological imaging scenarios.

In summary, we present FDT as a multimodal imaging modality for reconstructing the 3D RI of biological specimens from diffracted fluorescence images acquired in an epi-detection (reflection-mode) geometry. We demonstrate FDT in two complementary configurations, in which fluorescence originated either from a fluorescent substrate beneath a label-free specimen or from fluorescence-labeled cells within the specimen. FDT has the potential to enable in vivo multimodal fluorescence-RI imaging using standard two-photon microscopy hardware.

## Methods

### Differential multi-slice model for 3D rendering

To improve computational efficiency and flexibility, we extend the conventional multi-slice model used in BRIEF^12^ to the PyTorch framework. The differentiable multi-slice model with automated backpropagation mechanisms allows us to adjust the model’s parameters arbitrarily. This flexibility enables fine-tuning of the RI resolution using the coarse-to-fine strategy and optimizing the self-calibration parameters for fluorescent illumination, as discussed earlier. In the multi-slice model, the 3D phase object is modeled as a stack of multiple *z*-layers, each with an unknown RI ${\hat{n}}_{k}({\bf{r}})$, where *k* = 1, 2, ..., *N*_*z*_. The 3D RI of a total of *N*_*z*_ layers is:

1 $$\hat{n}\triangleq {\left\{{\hat{n}}_{k}({\bf{r}})\right\}}_{k=1}^{{N}_{z}},\quad {\bf{r}}=(x,y),$$

where **r** = (*x*, *y*) is the spatial coordinates. As light propagates through each layer, it is altered according to the transmission function *t*_*k*_(**r**) at *k*^*th*^ layer:

2 $${t}_{k}({\bf{r}})=A({\bf{r}})\exp \left(\dfrac{j2\pi }{\lambda }\Delta z({\hat{n}}_{k}({\bf{r}})-{n}_{b})\right),\quad k=1,2,\ldots,{N}_{z},$$

where *A*(**r**) is the spatially-varying absorption at this layer, *λ* is the wavelength of the light, Δ*z* is the thickness of each layer, and *n*_*b*_ is the RI of the background medium. As the absorption coefficient of mammalian cells and tissues used in the work is orders of magnitude lower than the scattering coefficient at visible to near-infrared wavelengths^57^, it is reasonable to neglect absorption in our current reconstruction model and set *A*(**r**) = 1. We then use the operator ${{\mathcal{P}}}_{\Delta z}$ to represent the Fresnel propagation of the field over a distance Δ*z*:

3 $${{\mathcal{P}}}_{\Delta z}\{\cdot \}={{\mathcal{F}}}^{-1}\left\{\exp \left(-j2\pi \Delta z\sqrt{{\left(\dfrac{1}{\lambda }\right)}^{2}-| | {\bf{k}}| {| }^{2}}\right)\cdot {\mathcal{F}}\{\cdot \}\right\},\quad {\bf{k}}=({k}_{x},{k}_{y}),$$

where ${\mathcal{F}}\{\cdot \}$ and ${{\mathcal{F}}}^{-1}\{\cdot \}$ denote the Fourier transform and its inverse, respectively, and **k** = (*k*_*x*_, *k*_*y*_) is the spatial frequency coordinates. The electric field from the *i*^*th*^ fluorescent source after propagating through the *k*^*th*^ layer is described by:

4 $${\hat{E}}_{k,i}({\bf{r}})={{\mathcal{P}}}_{\Delta z}\left\{{t}_{k}({\bf{r}})\cdot {\hat{E}}_{k-1,i}({\bf{r}})\right\},$$

where ${\hat{E}}_{k-1,i}({\bf{r}})$ is the electric field at the (*k*−1)^*th*^ layer and ${\hat{E}}_{k,i}({\bf{r}})$ is the electric field at the *k*^*th*^ layer. If the image plane is at the top surface of the phase object (the final *z*-layer), the electric field at the camera under *i*^*th*^ fluorescent illumination can be calculated by applying the pupil function *p*(**k**) to the field at the final layer:

5 $${\hat{E}}_{i}({\bf{r}})={{\mathcal{F}}}^{-1}\left\{p({\bf{k}})\cdot {\mathcal{F}}\left\{{\hat{E}}_{{N}_{z},i}({\bf{r}})\right\}\right\},$$

where ${\hat{E}}_{{N}_{z},i}({\bf{r}})$ is the electric field at the final layer. If the image plane is Δ*Z*_*c*_ below the final layer, as is the case when the phase object is thick, the field is first back-propagated over a distance Δ*Z*_*c*_ before passing through the pupil and arriving at the camera:

6 $${\hat{E}}_{i}({\bf{r}})={{\mathcal{F}}}^{-1}\left\{p({\bf{k}})\cdot {\mathcal{F}}\left\{{{\mathcal{P}}}_{-\Delta {Z}_{c}}\left\{{\hat{E}}_{{N}_{z},i}({\bf{r}})\right\}\right\}\right\},$$

where ${{\mathcal{P}}}_{-\Delta {Z}_{c}}$ is the back-propagation operator. Since the camera can only detect the intensity of light field, the intensity image captured under *i*^*th*^ fluorescent illumination is described as:

7 $${\hat{I}}_{i}({\bf{r}})=| {\hat{E}}_{i}({\bf{r}}){| }^{2}.$$

This mathematical model enables the simulation of the electric field’s propagation through the layers of a sample within the PyTorch framework. However, the model assumes coherent light, while in experiments, two-photon excited fluorescence is partially coherent/incoherent light sources and spatially varying (depending on the local fluorescent label), leading to a mismatch between the model and actual experiments. We address this issue using a partially coherent mask.

### Coarse-to-fine process

The coarse-to-fine process divides the training into three stages with a gradually increasing sampling grid in the *xy* plane. In the simulation experiment of the UCDavis pattern, the total number of training iterations is 300. During the first 100 iterations, a coarse sampling grid of 128 × 128 pixels is used to model the unknown RI. For the subsequent 100–200 iterations, the sampling grid is increased to 256 × 256 pixels. Finally, during the 200-300 iterations, the highest sampling grid of 512 × 512 pixels is used. The number of *z*-layers remains at 14 throughout all three stages. At the transition points, bilinear interpolation is employed to upsample the RI to ensure smooth transitions between different sampling grids. Both experiments use the Adam optimizer with a learning rate of 5 × 10^−3^, and each training run takes approximately 20 min for 300 iterations.

### Self-calibration of fluorescent illumination

In the simulated dataset of UCDavis with known ground-truth positions of fluorescence illumination, to mimic the uncertainty of initial positions in the realistic case, the initial position ${{\hat{\boldsymbol{p}}}}_{{\boldsymbol{i}}}=({\hat{p}}_{ix},{\hat{p}}_{iy},{\hat{p}}_{iz})$ is randomly drawn from a Gaussian distribution ${\mathcal{N}}(0,{\sigma }^{2})$ with uncertainty relative to the ground-truth position, defined as ${{\hat{\boldsymbol{p}}}}_{{\boldsymbol{i}}}={{\boldsymbol{p}}}_{{\boldsymbol{i}}}+{\mathcal{N}}(0,{\sigma }^{2})$. The ground-truth position ***p***_***i***_ is normalized to the range [−0.5, 0.5], while the STD *σ* is set to be 0.1 to simulate the case where the initial estimation of the position is significantly different from the ground-truth. In the experimental data (Section “Evaluation with experimental data of biological samples”), the initial position ${{\hat{\boldsymbol{p}}}}_{{\boldsymbol{i}}}$ is determined by fitting a 2D Gaussian function to the defocused fluorescence images. The mean of the 2D Gaussian represents the lateral position of the fluorescent source, while the STD corresponds to the defocus distance in the *z*-axis. For one defocused fluorescence image *I*_*i*_, the initial fluorophore position ${{\hat{\boldsymbol{p}}}}_{{\boldsymbol{i}}}$ is solved by:

8 $${\mathop{{\rm{arg}}{\rm{min}}}\limits_{{\hat{p}}_{ix},{\hat{p}}_{iy},{\hat{p}}_{iz},A}}\mathop{\sum }\limits_{x=1}^{N}\mathop{\sum }\limits_{y=1}^{N}{\left| \left| {I}_{i}(x,y)-A\exp \left\{-\dfrac{1}{2{\sigma }_{z}{({\hat{p}}_{iz})}^{2}}\left[{(x-{\hat{p}}_{ix})}^{2}+{(y-{\hat{p}}_{iy})}^{2}\right]\right\}\right| \right| }_{2}^{2}.$$

These positions are then set as iterative parameters that are updated continuously throughout the training process.

### Loss function with partially coherent masks

The loss function is designed to optimize the 3D RI and the fluorescence-source parameters by minimizing the discrepancy between the measured and predicted diffracted fluorescence images generated using the multi-slice model under the Born approximation. To improve computational efficiency and robustness, rather than directly comparing the predicted and measured diffracted fluorescence images, we apply a weighting factor (i.e., the partially coherent mask) to both images. This mask is generated by binarizing the predicted coherent illumination pattern at the image plane in the absence of phase objects (i.e., for a homogeneous RI):

9 $$M({\bf{r}})=1\,\left({\hat{I}}_{i,{\mathrm{homo}}}({\bf{r}}) > T\right),\qquad M({\bf{r}})\in \{0,1\},$$

where the threshold *T* is typically set to half of the normalized peak intensity of ${\hat{I}}_{i,{\rm{homo}}}({\bf{r}})$. We then apply the mask to both the measured and predicted diffracted fluorescence images:

10 $${I}_{{i},{\rm{mask}}}\,({{\bf{r}}})={M}\,({{\bf{r}}})\,{I}_{i}\,({{\bf{r}}}),\quad {\hat{I}}_{i,{\mathrm{mask}}} ({{\bf{r}}})={M}\,({{\bf{r}}})\,{\hat{I}}_{i}\,({{\bf{r}}}).$$

The weighted loss for each measurement-prediction pair is then calculated as

11 $$\begin{array}{cc}{\mathcal{L }}(\hat{n};{I}_{i,\mathrm{mask}}) &=\alpha \parallel {\hat{I}}_{i,{\mathrm{mask}}}-{I}_{i,{\mathrm{mask}}}{\parallel }_{1}+\beta \parallel {\hat{I}}_{i,{\mathrm{mask}}}-{I}_{i,{\mathrm{mask}}}{\parallel }_{2}^{2}\\ &+\gamma {{\mathscr{\ell}}}_{i,{\mathrm{ssim}}}(\hat{n};{I}_{i,{\mathrm{mask}}})+{\tau }_{xy}{R}_{xy}(\hat{n})+{\tau }_{z}{R}_{z}(\hat{n}),\end{array}$$

where $\hat{n}$ denotes the predicted RI. Correspondingly, the overall loss is

12 $${\mathcal{ L }}(\hat{n};{I}_{1,{\mathrm{mask}}},{I}_{2,{\mathrm{mask}}},\ldots,{I}_{{N}_{B},{\mathrm{mask}}})=\sum _{i=1}^{{N}_{B}}{\mathcal{ L }}(\hat{n};{I}_{i,{\mathrm{mask}}}).$$

Each term in the loss function is weighted by specific parameters (*α*, *β*, *γ*, *τ*_*xy*_, and *τ*_*z*_) to balance their contributions based on the model’s objectives. These hyperparameters are carefully tuned to optimize performance for the specific application. The L1 loss, representing the mean absolute error, promotes sparsity in predictions, while the L2 loss, representing the mean squared error, helps minimize large deviations. Initially, *α* is set to 0 to focus on reducing large errors, and it is gradually increased during training to shift the emphasis towards sparsity and fine-tuning with *β* decreases correspondingly, where *α* + *β* = constant. This dynamic adjustment allows the model to start by making broad corrections and then progressively refine its outputs, creating a robust framework for achieving high-quality predictions. Additionally, the SSIM loss is included to assess the similarity between predicted and ground-truth images, capturing perceptual differences and preserving image quality, as described below:

13 $${{\mathscr{\ell }}}_{{\rm{ssim}}}(\hat{n};{I}_{i})=1-\,{\mathrm{SSIM}}\,(\hat{{I}_{i}},{I}_{i}),\,\,\,{\mathrm{SSIM}}\,(x,y)=\dfrac{(2{\mu }_{x}{\mu }_{y}+{c}_{1})(2{\sigma }_{xy}+{c}_{2})}{({\mu }_{x}^{2}+{\mu }_{y}^{2}+{c}_{1})({\sigma }_{x}^{2}+{\sigma }_{y}^{2}+{c}_{2})}.$$

Furthermore, to promote smoothness in the RI along the *x*, *y*, and *z* axes, we incorporate TV regularization terms. Given the differing resolutions in the *xy* and *z* planes, we employ two separate weighting terms to control the contribution of these regularizers, which are defined as:

14 $${R}_{xy}(\hat{n})=\parallel {\nabla }_{x}\hat{n}{\parallel }_{1}+\parallel {\nabla }_{y}\hat{n}{\parallel }_{1},\,\,{R}_{z}(\hat{n})=\parallel {\nabla }_{z}\hat{n}{\parallel }_{1}.$$

### Cell line sources

MDCK GII cells (female) were provided by Dr. Soichiro Yamada at the University of California, Davis, who obtained them from the James Nelson laboratory at Stanford University. Primary bovine muscle stem cells used to generate the 3D-cultured myotube samples were provided by Dr. Jiandi Wan at the University of California, Davis. The donor animal was a 15-month-old Angus heifer. Skeletal muscle tissue was collected from the shank post-slaughter at the UC Davis Meat Lab, and the stem cells were isolated in Professor Lucas Smith’s laboratory on July 25, 2023 (sample ID: ISO10 7252023). HEK293T cells were provided by Dr. Aidan Gilchrist at the University of California, Davis; these cells were purchased from ATCC (cat. no. CRL-3216).

### Reporting summary

Further information on research design is available in the Nature Portfolio Reporting Summary linked to this article.

## Supplementary information

Supplementary Information

Description of Additional Supplementary Files

Supplementary Movie 1

Supplementary Movie 2

Supplementary Movie 3

Supplementary Movie 4

Supplementary Movie 5

Supplementary Movie 6

Reporting Summary

Transparent Peer Review file

## Data Availability

The source data generated in this study are provided in the main text and Supplementary Information and are also available on the project website https://xue-lab-cobi.github.io/fdt/.

## Code availability

The code generated in this study has been deposited in the GitHub repository (https://github.com/Xue-Lab-COBI/fdt_code) and archived on Zenodo under the permanent https://doi.org/10.5281/zenodo.21930595^58^. No restrictions apply to code access.
