# Supplementary material for: Fluorescence-illuminated diffraction tomography using explicit neural fields
Source: Nat Commun. 2026 Oct 10;17:11519. doi: 10.1038/s41467-026-78326-x (PMC13745583; doi:10.1038/s41467-026-78326-x)
Supplement: Supplementary file 1 [file 41467_2026_78326_MOESM10_ESM.pdf]

nature portfolio

Peer Review File

Fluorescence-illuminated Diffraction Tomography using Explicit Neural Fields

Corresponding Author: Professor Yi Xue

This file contains all reviewer reports in order by version, followed by all author rebuttals in order by version.

Version 0:

Reviewer comments:

Reviewer #1

(Remarks to the Author)
I have carefully read the document submitted by Dr. Yi Xue and his co-authors, entitled: "Fluorescence-illuminated Diffraction Tomography using Explicit Neural Fields"

As I understand it, the 3D distribution of the refractive index is estimated by first recording a series of defocused diffracted fluorescence images for each position of the laser beam focus, and then using a postprocessing approach based on the use of "explicit neural fields". Compared with previously published work, the authors have made hardware and software improvements that 1) make refractive index reconstruction: more reliable and faster and 2) enable this imaging technique to be used for a wider range of samples: possibility of denser labelling, imaging at greater depths using signals collected in epi (backward direction).

In particular, the authors first mention the use of an explicit neural network, which they claim reproduces what would be done by solving the problem numerically using the intensity transport equation, or by using an implicit neural network, which, again according to the authors, should give similar results to the numerical approach, but at a lower computational cost and better results than with an implicit neural network. Secondly, to achieve better results with dense labeling and at greater depth, the other authors propose the use of two-photon excited fluorescence, which enables emission from a single point source to be obtained robustly through the two-photon absorption process, and limits signal emission to the proximity of the beam focus for each beam position scanned in the sample.

The work presented by the authors is original and interesting, but I have two main concerns about the manuscript presented in its current form: it's not clear that the authors are actually demonstrating that two-photon excited fluorescence can be used for phase imaging on thick tissue, as the fluorescence seems to come from a fine distribution of fluorophore placed under the samples, so point sources would be obtained in all cases I guess (which I don't think is clearly mentioned in the text and methods)? Furthermore, a convincing demonstration would use the fluorescence of the sample itself, whether endogenous or exogenous, which seems to be implicitly stated at the beginning of the abstract.
The authors also need to better describe in which situations this approach would or would not work, and which parameters or physical aspects should conceptually limit the performance of phase imaging by detecting fluorescence emitted by source points.

Please find below my remarks, comments and detailed questions to the authors:

The authors need to better describe in which situation this approach would or would not work and which parameters or physical aspects should conceptually limit the performance of phase imaging by fluorescence detection, in particular it seems that the detection path is particularly important. They could, for example, develop some of the following aspects, or others if they consider them more relevant:

• How should sample properties affect the reconstruction: density and spatial homogeneity of the labeling, presence of additional absorbers, distortion of the exciter beam and of the collected signal in the case of slow or high spatial variation of the refractive index (optical aberration and scattering) and presence of several spectrally distinct fluorophores, etc.?
• How the properties of the excitation beam should affect reconstruction: axial and lateral extension, spatial sampling when raster scanning the beam across the sample ?

• How would the detection configuration affect the reconstruction: numerical aperture in collection, spatial sampling with the camera, vignetting of the fluorescence signal along the detection path?
• What affects the technique performance:
o What limits the size of the field of view? There seems to be more artifacts at the edge of the field of view? I assume this is due to the diffracted pattern being progressively incompletely detected as you move away from the optical center?
o Detection efficiency decreases progressively with depth in multiphoton microscopy because for each point source in the sample, the signal seems to come from an extended surface and the extension of this apparent surface increases with depth (https://pmc.ncbi.nlm.nih.gov/articles/PMC3482884/). I assume this will also affect the quality of the reconstruction? Does this mean that higher scattering coefficients require higher collection NAs? Does this model also take into account the fact that a part of the forward scattered light is redirected in epi ?
o Ability to distinguish small details ? I suppose we can not define a proper spatial resolution as for incoherent imaging ?
o What is conceptually limiting the imaging depth ?
o How does the acquisition parameters will influence the reconstruction quality: spatial sampling for beam scanning and detection, SNR, camera type of noise ect…

“Our data demonstrate that explicit neural fields not only accelerate computation but also deliver significantly enhanced reconstruction quality. Therefore, we use explicit neural fields for high-resolution 3D reconstruction of RI.” Could the authors better justify why “explicit neural fields” would be conceptually better suited to this task?

The authors use two-photon absorption to obtain the emission from a single point source. As I understand it, the validity of this assumption is crucial for faithful reconstruction. However, for increasing imaging depth, the signal from the sample surface will progressively increase relative to the signal from the focus (https://doi.org/10.1364/JOSAA.23.003139). How will this affect the estimation of the refractive index, since this assumption of localized signal emission is progressively not respected?

Figure 2, panel g). How do the authors interpret the variations in shape between SNRs 5 and 20? Does performance seem to deteriorate at higher SNRs?

The authors mentioned at several places in the manuscript multimodal fluorescence and phase imaging, for example: “FDT is a critical step toward multimodal fluorescence and phase microscopy for bulky tissue and ultimately for in vivo imaging in the future”. Can the authors demonstrate or explain how they would efficiently and conveniently obtain both 3D IR and fluorophore distribution?

Validation with a single type of structure is not sufficiently convincing to assert that this work can be transposed in a general case for different types of samples and structures. The authors should consider more diversified ground truth data for their demonstration, or show conceptually in which framework good and bad results can be expected. Could the authors identify and describe expected reconstruction artifacts and how to remove them? So that non-expert users can identify them unambiguously and know how to optimize data acquisition and reconstruction?

“We quantitatively validate FDT through simulations and experimental data of silica beads with known ground-truth RI, and then demonstrate its capability by reconstructing the 3D RI of living cells and bulky tissue volumes (530×530×300 μm3) with subcellular resolution”. “Our results reveal individual cells with diverse morphological structures and RI during the processes of differentiation and aggregation (Fig. 4e). This demonstrates that FDT not only achieves high resolution but also effectively reconstructs structures across both low and high spatial frequencies”

Imaging the “bulky volume” is not really a demonstration of the technique's validity, as approaches based on post-processing reconstruction of raw data will always produce results, but it must be carefully checked that no artefactual data is produced, which, as I understand it, is not the case for this specific acquisition. Asserting that there is some spatial variation in the signal and that the visible object resembles known cells does not seem to be a strong justification that the reconstruction faithfully reflects the object. Could the authors define more clearly what is meant by a measurement image? Is it a transmission image of the excitation beam? But then the two signals are measured at different wavelengths? Doesn't the fact that the recovered phase reflects the transmitted image necessarily mean that it's correct? Is it possible to have several possible phase distributions for a single transmitted image?

Minor details: “The galvo mirrors and the electrically tunable lens can be functionally replaced by a spatial light modulator for beam steering, as both approaches achieve the same control of light in 3D (Fig. 1e, inset).” -> But not with the same temporal resolution as the refreshing rate of typical commercially available SLM is around 60Hz ie 17ms compare to the time response of standard galvos which goes up to 1µs. Justify more clearly the choice of moving optically the beam to change the axial position. I guess this is to have a fix collection configuration which is critical for the reconstruction ?

(Remarks on code availability)

Reviewer #2

(Remarks to the Author)
The manuscript presents a novel technique, Fluorescence-illuminated Diffraction Tomography (FDT), which reconstructs the 3D refractive index (RI) of thick, label-free biological tissues using diffracted fluorescence images acquired in reflection mode under two-photon excitation. The authors integrate an “explicit neural field” model to improve reconstruction speed

and accuracy, and validate the system through both simulated data and real biological specimens.
While the manuscript introduces a novel concept with innovative implications for deep-tissue multimodal imaging, the proposed method still requires the installation of a fluorescent bead layer on the opposite side of the sample. This raises concerns about whether the method can truly be considered a reflection-mode technique. The authors claim in the abstract, introduction, and conclusion that their approach operates in reflection mode and differs from transmission-mode imaging modalities that require access to the opposite side of the sample. However, because their method still necessitates the placement of fluorescent beads beneath the sample, it is unlikely to be applicable to in vivo imaging. For this reason, the authors' claims regarding reflection-mode operation are significantly compromised, and the manuscript, in its current form, does not appear to meet the publication standards of Nature Communications.

Several critical issues in model validation, methodological assumptions, biological interpretation, and quantitative performance analysis also need to be addressed.

The entire framework is based on a coherent forward model, while fluorescence emission—especially from two-photon excitation—is fundamentally incoherent or partially coherent. Although the authors attempt to resolve this with a partially coherent mask, this approach lacks theoretical justification or quantitative validation. The effect of using the partially coherent mask is presented in Fig. S2, but the effect of the coherence mismatch remains unclear. How does this mismatch affect reconstruction fidelity? Is the Born approximation still valid under this condition?

Claims of subcellular resolution and high z-sectioning capability are repeatedly made, yet no formal resolution analysis (e.g., lateral/axial FWHM, modulation transfer function, Rayleigh limit) is provided. In figures in the manuscript, while images appear visually plausible, resolution is not quantified, and it remains unclear how much of the structure is truly resolved. The authors should investigate the lateral resolution and optical sectioning capabilities systematically and quantitatively.

The explicit neural field method is claimed to be faster than implicit alternatives, yet computational cost is still uncertain (e.g., 50 minutes to 3 hours per reconstruction). What hardware is required? How does performance scale with volume size? The method's feasibility for broader adoption is unclear without reporting detailed runtime benchmarks, GPU memory requirements, and potential limits of reconstruction volume.

(Remarks on code availability)

Reviewer #3

(Remarks to the Author)
The authors developed fluorescence-illuminated diffraction tomography to reconstruct the 3D refractive index (RI) of label-free objects from diffracted fluorescence images acquired in reflection mode under two-photon excitation. They employed the transport of intensity equation (TIE) and a self-supervised neural network to enhance reconstruction accuracy.
1. Firstly, several publications have demonstrated multimodal microscopy in reflection mode. there are reflection-mode techniques specifically designed for imaging 3D thick tissues in phase microscopy. For instance:
o https://opg.optica.org/optica/fulltext.cfm?uri=optica-12-3-406&id=569159
o https://www.pnas.org/doi/10.1073/pnas.2115323118
o https://www.nature.com/articles/s41467-019-12634-3
o https://opg.optica.org/boe/fulltext.cfm?uri=boe-10-7-3605&id=414828
o Tomocube (although works in the transmission mode but can image thick sample)
Therefore, stating that most techniques operate in transmission mode without acknowledging the existing reflection-mode methods may be misleading.
2. When the authors mention various types of reflection-mode phase imaging (line 59), I believe it would be feasible to integrate reflection-mode intensity diffraction tomography (recently introduced by Tian's group) with other fluorescence-based techniques such as SIM or TIRF. This possibility does not appear problematic and could have been discussed.
3. Lines 81–100: The authors attempt to address the challenge of imaging thick tissue by referring to their previously published work, BRIEF. They claim that accurate 3D RI of bulky tissue can be achieved via new algorithmic developments. However, the claimed accuracy is not substantiated in the presented results. The authors imaged MDCK cells and bovine myotubes, but no quantitative assessment is provided to demonstrate the precision of their RI reconstruction compared to established methods such as multi-slice beam propagation or nonlinear Born approximation (Park group, optica 2022).
4. Lines 101–134: The authors present FDT (explicit neural fields from two-photon fluorescence images) for 3D RI reconstruction. However, a clearer explanation of how FDT reconstructs the 3D RI using explicit neural fields is needed. Additionally, the authors should elaborate on the advantages of their method over conventional techniques. The comparison to references 37–42 appears to focus solely on computational speed and scalability, which seems inconsistent with the prior emphasis on accurate RI reconstruction. The manuscript does not convincingly explain how their approach offers superior RI accuracy or reconstruction quality.
5. Lines 137–154: The stated innovation of using a 2D camera instead of a PMT to record RI of label-free structures requires further clarification. Specifically, it is unclear how diffracted fluorescence images—where phase information is inherently lost—can be used to recover phase information required for RI reconstruction. While some discussion appears in lines 285–292, this comes too late to clearly understand the fundamental illumination and collection strategy. Moreover, the manuscript does not specify whether focused or wide-field illumination was used, which affects the reconstruction method. It is also unclear whether each two-photon signal was treated as a point source during RI reconstruction. These are important technical details that should be clarified.

6. The results section does not clearly describe how fluorescence imaging contributed to RI reconstruction. Furthermore, the claim of reconstructing thick samples up to 300 microns deep raises questions, as multiple scattering would likely degrade image quality. The manuscript lacks explanation of how such scattering was mitigated.
7. Lines 156–208: The architecture of the FDT model is presented, but it remains unclear how amplitude information is learned from intensity-only fluorescence images. The fundamental distinction between coherent (label-free) and incoherent (fluorescence) imaging is not adequately addressed. The self-calibration method, though useful, has been previously explored by other groups and should not be considered a novel contribution. Regarding the differential multi-slice model: is there a conceptual or practical comparison with the established multi-slice beam propagation method, which is also designed for thick tissue imaging? A comparative discussion would be beneficial.
The manuscript also refers to a partially coherent mask designed to minimize discrepancies. However, it is unclear what specific discrepancies are being addressed. Is the discrepancy related to the loss of phase in fluorescence imaging compared to phase-preserving coherent imaging?
8. Lines 285–383: The explanation becomes somewhat clearer here. The label-free image is obtained with cells cultured on the bottom of a glass dish, and the fluorescence dye is applied externally. Does this imply that each dye particle acts as a point source in the reconstruction? This could be explicitly stated.
9. Lines 294–341: The fluorescence source is located 105 microns beneath the cells. It would be helpful to know whether using LEDs instead of fluorescence sources would alter the reconstruction results. The claim of higher sensitivity and larger field of view lacks empirical support. For example, can the authors demonstrate that their method can detect RI changes with a sensitivity of 0.005? A quantitative evaluation would significantly strengthen the manuscript.
10. Figure 4 presents the central result, where the authors attempt to reconstruct the 3D RI of thick bovine myotubes. While imaging thick samples is indeed a pressing issue in QPM, the reconstruction quality shown here is insufficient to validate the claimed accuracy and sensitivity of the method.
Therefore, in my opinion, the paper is missing several important details and lack to explain the precise novelty and proof the novelty through their results.

(Remarks on code availability)

Reviewer #4

(Remarks to the Author)
Review of NCOMMS-25-28022 “Fluorescence-illuminated Diffraction Tomography 2 using Explicit Neural Fields,” by R. He et al.

This manuscript presents an approach for using fluorescence illumination to conduct diffraction tomography for reconstructing the refractive index distribution of samples. The approach works by having a fluorescent layer beneath the sample of interest and then exciting it point wise by two photon illumination. The diffracted light is then analyzed to reconstruct the structure of the sample supported by transport of intensity equation and the solved with a self supervised neural network.

The approach builds upon previous work that demonstrated the general principle (Ref 12), while the two photon excitation was introduced in a conference presentation. The advances here are to switch from implicit to explicit fields where a point wise reconstruction is used rather than a continuous function. Additional points of novelty are a self-calibration routine, use of defocused images on a 2D sensor rather than a point sensor and introduction of a partially coherent mask.
In the introductory work, there are several repeated citations (3,18), (1,19), (11, 21) & (2, 22) are re-used. The authors should also compare with the back oblique microscopy approach developed by Prof. Robles at Georgia Tech which operates on a similar principle to obtain quantitative phase information.

Several experiments are presented to demonstrate the approach. The first is a simulation of the approach using a phantom comprising letters which shows good reconstruction. Experiments using a silica bead in PDMS are also shown. While an SNR range is evaluated, the figure of 5.78 is chosen as accurate but this analysis lacks quantitative comparisons and some description of the chosen threshold is needed. For example a halo effect is seen for the images at SNR 10.05 and SNR 20.80 not present in SNR 5.78. No errors are given for these reconstructions.

An experiment with cells is shown next. While the reconstruction appears generally correct, there are errors of up to 0.2. The presented results suggest that the explicit model is qualitatively better than the implicit model. While the error is stated as minimal, 0.2 can be quite substantial and the authors are encouraged to more accurately discuss the fidelity. There is some concern here that the continuous refractive index changes of the cell are not as accurately reproduced as the technical sample. The course to fine approach that is described should do a better job at the cell edges where the largest errors appear.

The final experiment is reconstruction of bovine myotube, approx. 300 microns thick. The volume appears to be reasonably well reconstructed but is limited in that no cell details are visible and the claim that cells and muscle cells are differentiated is not well supported.

The discussion is good but has some points that need to be better justified. In particular, the manuscript in general has a lot of discussion of epi-mode and multimode imaging yet these are not really the focus of the manuscript. The epi-mode is

achieved by locating a fluorescent layer beneath the sample. This is a bit of an artificial simulation as it will not truly allow single ended access without adding a fluor underneath. While the discussion suggests use of endogenous fluorescence for this purpose, it is not likely to be strong enough for this method. Instead an injected contrast agent might be able to do the trick but limits the label free nature of the approach. The combination of fluorescence and quantitative phase imaging is a compelling modality but is not truly employed here. There is discussion of the combination though.

There are claims of subcellular resolution but these are not necessarily supported by the presented cell images. While there are some variations within the cell shown, it is not clear if these are accurately presenting cell structures. There is some question about what subcellular information is obtained and whether it is accurate or useful.

Some additional points that should be addressed:
Line 65-66: An alternative approach would be to use spectrally distant illumination sources which would prevent crosstalk
Figure 2: Perhaps use different colors for Implicit neural field vs Explicit neural field in the legend
Figure 2: Fig2(h) title should say ‘data’ not ‘date’
Line 266: Since the explicit so strongly outperforms implicit model, why hasn’t it been developed before for DT? Are there Potential drawbacks for explicit model or is the reconstruction time a limitations.
Fig 3:
Label z-depth of Fig 3 (d-f).
Label Fig 3 (d-f) explicit, Fig (g) implicit
Give units for z-depth on Fig 3(h) top row
Label explicit vs implicit on Fig 3(h)
Line 307: Cells detached from substrate should have sharp RI increase, shouldn’t course to fine process described in Fig 1 account for this?
Line 315: Higher RI because cell is detached from substrate and intracellular material condenses (other than just chromatin) [IE high RI on cell edge].
Line 348: Does changing the z-depth of the fluorescence source improve the resolution? How/why was 210 micron chosen as illumination plane?

In summary, this is an interesting advance for reconstructing refractive index distributions of thick samples. The images are compelling and there is innovation in the improvements to the approach over previous works. There are limitations in the presentation which could be addressed (as described above). Revision of the manuscript to address these issues could result in a publishable manuscript.

(Remarks on code availability)

Reviewer #5

(Remarks to the Author)
I co-reviewed this manuscript with one of the reviewers who provided the listed reports. This is part of the Nature Communications initiative to facilitate training in peer review and to provide appropriate recognition for Early Career Researchers who co-review manuscripts.

(Remarks on code availability)

Version 1:

Reviewer comments:

Reviewer #1

(Remarks to the Author)
I have now finished carefully reading the new submission provided by Yi Xue and co-authors entitled: "Fluorescence-illuminated Diffraction Tomography using Explicit Neural Fields". I would like to thank the authors for their constructive answer and for taking my comments into account. I believe the article can now be published in Nature Communications.

Minor comment : the code is actually not visible in the provided link.

(Remarks on code availability)
The code is not available or visible via the link provided by the authors : https://github.com/cubhe/fdt_code

Reviewer #2

(Remarks to the Author)
The authors have made substantial efforts to address the concerns raised in my previous review. In particular, the revised manuscript is significantly clearer regarding the imaging geometry, the role of the fluorescent substrate in the original

experiments, and the distinction between epi-collection geometry and true transmission-side independence. I appreciate that the authors have clarified the use of the fluorescent paint layer in the silica bead, MDCK cell, and bovine myotube experiments, and, more importantly, have added a new experiment using fluorescence-labeled HEK293T cells in which the fluorescence originates from within the sample itself without requiring transmission-side access. This additional demonstration directly addresses my major concern regarding the applicability of the method beyond substrate-assisted configurations.
The authors have also satisfactorily strengthened the technical foundation of the work. The discussion of the partially coherent fluorescence source and the role of the partially coherent mask is now much more precise, and the revised manuscript appropriately frames the mask as a practical weighting strategy to improve reconstruction robustness rather than as a complete physical treatment of fluorescence coherence. The authors also clarify the applicability and limitations of the Born approximation in weakly scattering samples. In addition, the newly added quantitative analysis of lateral and axial resolution, including theoretical estimates, PSF/MTF analysis, and simulated bead-separation tests, substantially improves the support for the claimed resolution and z-sectioning capability. Finally, the added information on runtime, GPU memory requirements, and scaling behavior adequately addresses my previous concern regarding computational feasibility and broader adoption.
Overall, I find that the authors have responded carefully and constructively to the major issues raised in my initial report, and the manuscript has been significantly improved.
I have one remaining minor concern. While the revised text is much more careful than the original version, the manuscript would benefit from maintaining a very explicit distinction between two experimentally demonstrated capabilities:
(1) 3D RI reconstruction of thick, label-free biological tissue using a fluorescent substrate beneath the specimen, as demonstrated with the bovine myotube sample; and
(2) 3D RI reconstruction without transmission-side access, as demonstrated using fluorescence-labeled HEK293T cells in which the fluorescence originates from within the sample itself.
These are both important advances, but they are not demonstrated simultaneously in the same experiment. Therefore, I recommend that the authors further ensure that the Abstract, Discussion, and Conclusion do not unintentionally imply that the manuscript has already demonstrated substrate-free, transmission-side-independent 3D RI reconstruction of thick label-free tissue. A brief clarifying statement explicitly distinguishing these two regimes would make the scope of the work more precise and prevent overinterpretation of its current in vivo relevance.
With this minor clarification, I believe the manuscript would be suitable for publication.

(Remarks on code availability)

Reviewer #4

(Remarks to the Author)
This revised manuscript was widely critiqued by four different reviewers with extensive revisions made in an effort to address them. In addition, a 67 page response was included also in an effort to rebut reviewers concerns.

In general, the approach is sound and capable of reconstructing refractive index distributions from fluorescent light. As noted previously, parts of this approach have been published before by these authors and others. Thus, the question remains is the work presented here a novel combination. The use of explicit neural fields appears to have utility but have been shown before. The novelty must then be viewed as the two photon fluorescence used as the illumination source. The data supporting this have appeared in numerous pre-prints and conference presentations. However, there is a novel aspect here that can be viewed as a significant advance, worthy of publication at this level.

The primary concerns of this reviewer previously focused on the overstated claims of the original manuscript. The author addresses some of these by softening the language in some places while in others, a lot more new data were provided. Primary concern is that the approach is touted as label free but requires a contrast agent. The author addresses of this concern by delineating experiments with a fluorescent substrate from those where the label is within the sample. In neither case is the technique truly label-free in the sense that the sample is not perturbed. The experiments without the substrate underneath use bright labels within the cell but these are exogenous. Similarly, the experiment with the bovine myotubule requires a fluorescent substrate. The question remains what is the benefit of this approach when any other incoherent light source could be used on the far side of the sample, with less instrument complexity. New analysis is presented to assert that the myotube images are accurate. It appears that the reconstruction is compared to the original measured data and good metrics are obtained. It is unclear how this justifies that accurate reconstructions are obtained. Fluorescence imaging of the myotubule are now presented but buried at the end of supplemental section. Overall, I do not feel that the shortcomings identified by this reviewer and others were well addressed.

A new experiment is included which uses fluorescently labeled cells. Images are reconstructed but no effort is made to prove that they are accurate. Instead, the features are just asserted to be ‘membrane-scale’. It's unclear if these are cell structures or ringing artifacts. These experimental results are not as well presented as others and are used to justify that images are obtained without the substrate. In my view, these do not add to the clarity of the manuscript.

In summary, the original manuscript was reviewed and produced similar criticisms from multiple reviewers. The new manuscript shows new data and explains a bit more but the central concerns remain that the approach is contorted into a label free description and the accuracy of the reconstructions are not sufficiently characterized.

(Remarks on code availability)

Open Access This Peer Review File is licensed under a Creative Commons Attribution 4.0 International License, which permits use, sharing, adaptation, distribution and reproduction in any medium or format, as long as you give appropriate credit to the original author(s) and the source, provide a link to the Creative Commons license, and indicate if changes were made.
In cases where reviewers are anonymous, credit should be given to 'Anonymous Referee' and the source.
The images or other third party material in this Peer Review File are included in the article's Creative Commons license, unless indicated otherwise in a credit line to the material. If material is not included in the article's Creative Commons license and your intended use is not permitted by statutory regulation or exceeds the permitted use, you will need to obtain permission directly from the copyright holder.
To view a copy of this license, visit https://creativecommons.org/licenses/by/4.0/

Response to the Reviewers

General Response

We thank the Reviewers for their thoughtful comments...

Response to Reviewer 1

Reviewer 1: I have carefully read the document submitted by Dr. Yi Xue and his co-authors, entitled: "Fluorescence-illuminated Diffraction Tomography using Explicit Neural Fields"

As I understand it, the 3D distribution of the refractive index is estimated by first recording a series of defocused diffracted fluorescence images for each position of the laser beam focus, and then using a postprocessing approach based on the use of "explicit neural fields". Compared with previously published work, the authors have made hardware and software improvements that 1) make refractive index reconstruction: more reliable and faster and 2) enable this imaging technique to be used for a wider range of samples: possibility of denser labelling, imaging at greater depths using signals collected in epi (backward direction).

In particular, the authors first mention the use of an explicit neural network, which they claim reproduces what would be done by solving the problem numerically using the intensity transport equation, or by using an implicit neural network, which, again according to the authors, should give similar results to the numerical approach, but at a lower computational cost and better results than with an implicit neural network. Secondly, to achieve better results with dense labeling and at greater depth, the other authors propose the use of two-photon excited fluorescence, which enables emission from a single point source to be obtained robustly through the two-photon absorption process, and limits signal emission to the proximity of the beam focus for each beam position scanned in the sample.

Comment 1. The work presented by the authors is original and interesting, but I have two main concerns about the manuscript presented in its current form: it's not clear that the authors are actually demonstrating that two-photon excited fluorescence can be used for phase imaging on thick tissue, as the fluorescence seems to come from a fine distribution of fluorophore placed under the samples, so point sources would be

obtained in all cases I guess (which I don't think is clearly mentioned in the text and methods)? Furthermore, a convincing demonstration would use the fluorescence of the sample itself, whether endogenous or exogenous, which seems to be implicitly stated at the beginning of the abstract.

Response. We thank the reviewer for this insightful comment. We agree that the samples used in our previous experiments (Figs. 2–4) require a fine distribution of fluorophores beneath the sample (Fig. R1a–c). Specifically, we spray fluorescent paint (Tamiya Color Spray Paint, purchased from Amazon) onto the slide or onto the outer surface of standard glass-bottom Petri dishes (D11130H, Matsunami Glass), thereby creating an approximately homogeneous fluorophore layer. The label-free cells and muscle tubes are placed inside the Petri dishes, while the fluorophore coating is applied to the outer surface, so the biological samples remain intact during this process. Importantly, the fluorescence distribution itself is not required to be point-like. It may also be continuous as shown in Fig. R1. With this fluorophore layer, we can selectively excite specific locations using a tightly focused two-photon beam, and the resulting fluorescence emission from the approximately diffraction-limited excitation volume acts as an effective fluorescent point source for illumination. We have added this clarification to the revised manuscript in the Results section, "Optical setup and fluorescence-illuminator characterization for FDT."

Following the reviewer's suggestion, we have performed additional experiments using the fluorescence of the sample as fluorescent illumination sources without fluorescent paint or transmission-side access (Fig. R1d). Specifically, we imaged 3D cultured HEK293T cells stained by exogenous fluorescence ActinGreen 488 (Fig. R2). We used two-photon excitation to excite fluorescence from cells located at a depth of approximately 130 μm depth within the sample. As the fluorescence propagated upward through the cells above toward the objective lens, it was diffracted by the heterogeneous refractive index (RI) of the cells. The resulting diffracted fluorescence pattern was captured at the sample surface. Compared with a homogeneously distributed fluorophore layer, fluorescence from 3D-distributed cells limits the illumination angles to the cell locations and exhibits brightness variations as expression levels vary across cells. The signal-to-noise ratio (SNR) of diffracted fluorescence images is generally lower than that obtained with a homogeneous fluorescent layer. As shown by the reconstruction results, FDT overcame these challenges and achieved high-resolution, high-sensitivity reconstruction over a large volume. These new data validate that FDT can reconstruct the 3D RI of thick biological samples using fluorescence from the sample itself without transmission-side access. We include the new section below for the reviewer's reference.

3D RI reconstruction of fluorescence-labeled HEK cells without transmission-side access

"To validate that FDT can reconstruct 3D RI when fluorescence originates *within* a thick specimen, we applied FDT to HEK293T cells cultured in a 3D Matrigel matrix and labeled with ActinGreen 488 (Fig. R2c; Extended Data Fig. 4d). Unlike the MDCK and bovine myotube experiments above, this measurement does not require

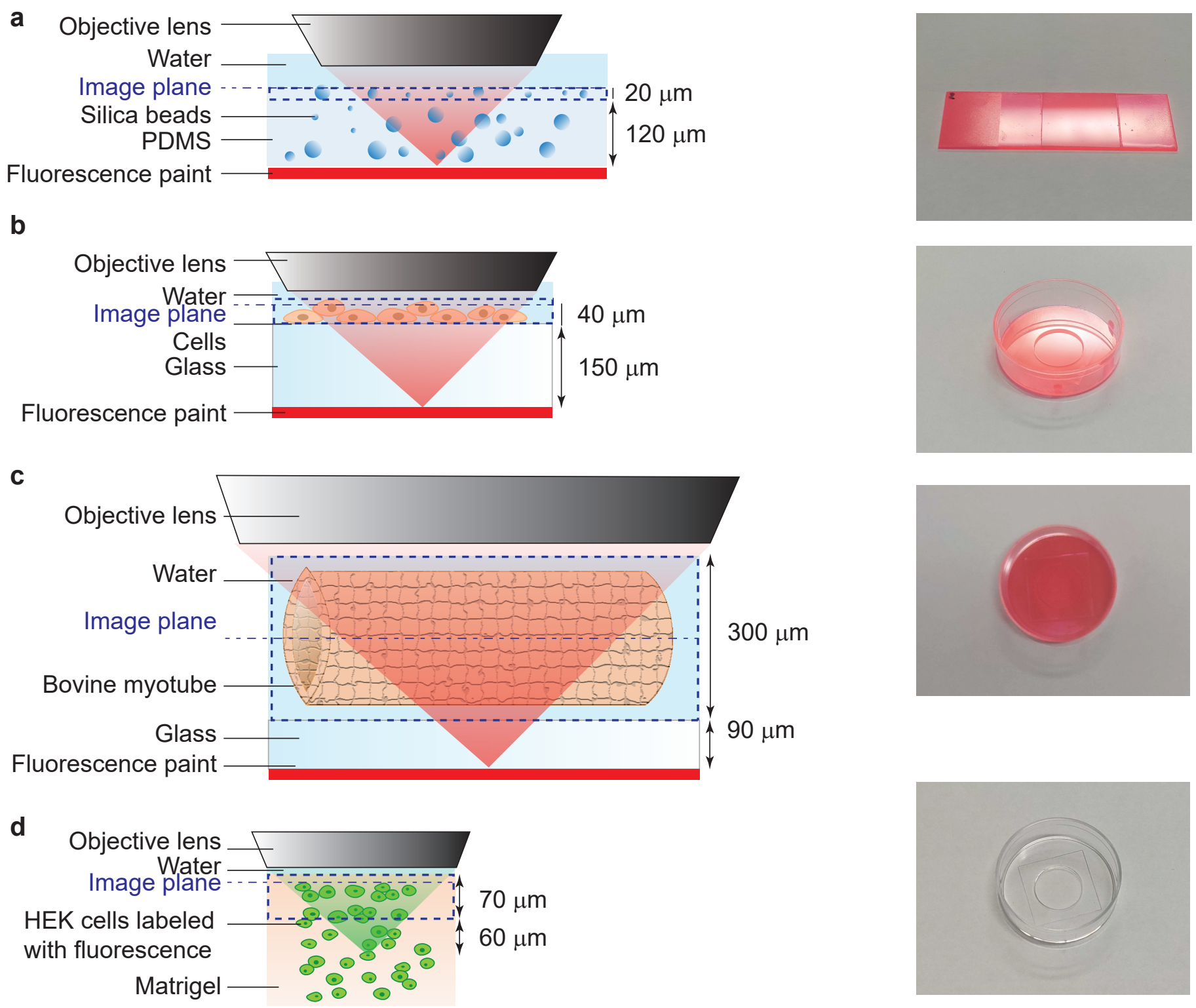

Fig. R1 Sample diagram. In the schematic, the gap between the objective lens and the sample is drawn shorter for illustration. In reality, this distance is approximately 2 mm and is set by the working distance of the objective lens. **a.** The sample used in Fig. 2 consists of silica microbeads fixed in PDMS and placed on top of a slide coated with fluorescent paint. A photograph of the sample is shown on the right. **b.** The sample used in Fig. 3 consists of living, label-free MDCK cells cultured in a coverslip-bottom dish. Fluorescent paint is sprayed onto the outside of the dish. A photograph of the fluorescent-paint dish is shown on the right. **c.** The sample used in Fig. 4 consists of label-free bovine myotubes cultured in 3D from muscle stem cells in Matrigel. During imaging, the sample is placed in a coverslip-bottom dish with fluorescent paint on the outer surface. A photograph of the fluorescent-paint dish is shown on the right. **d.** The sample used in Fig. 5 (Fig. R2) consists of fluorescence-labeled HEK293T cells cultured in 3D in Matrigel. During imaging, cells at a depth of approximately 130 μm are excited to serve as fluorescence sources, while the imaging plane is near the top surface of the sample. Other labeled HEK cells far below the excitation plane and the bottom of the dish are not shown because they are not relevant to the experiment. A photograph of the dish is shown on the right.

access to the transmission side to apply fluorescent paint, making the approach applicable to specimens for which transmission-side access is not available. We used two-photon excitation to generate localized fluorescence within the cells at a depth of 130 μm below the sample surface (Fig. R2c). As fluorescence propagates upward toward the objective, it is diffracted by the RI variations of overlying cells. All diffracted fluorescence images were acquired on a single camera plane near the sample

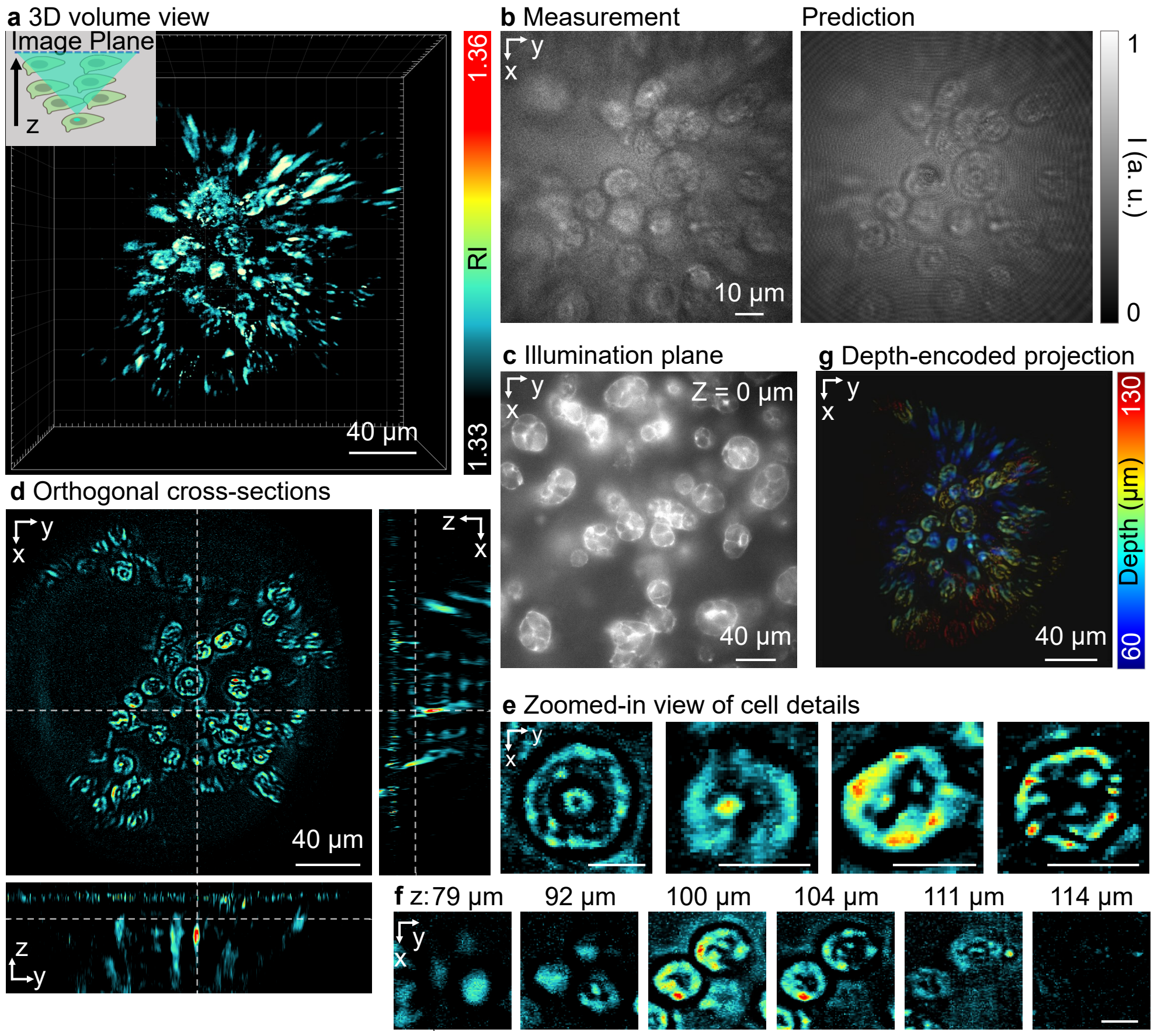

Fig. R2 3D RI reconstruction of ActinGreen 488-labeled HEK cells illuminated by fluorescence originating from the sample itself. (a) Reconstructed 3D RI of HEK cells over a 198 × 198 × 70 μm^3 volume. Inset: imaging geometry in which fluorescence-labeled cells serve as internal illuminators; their emitted fluorescence is diffracted by overlying cells and recorded as defocused images near the sample surface (see Extended Data Fig. 4d for details). (b) Representative diffracted fluorescence measurement (left) and corresponding prediction (right). (c) One-photon wide-field fluorescence image of HEK cells at $z = 0$ that act as fluorescence illuminators. (d) Orthogonal RI cross-sections $(x - y, x - z, y - z)$; dashed lines indicate slice locations. (e) Zoomed-in RI images of representative cells showing clearly resolved membrane-scale features. Scale bar, 10 μm. (f) Zoomed-in RI z-stack demonstrating z-sectioning by resolving three cells at different depths. Scale bar, 10 μm. (g) Depth-encoded projection of the reconstructed RI volume.

surface (Fig. R2b, left; Extended Data Fig. 3d), and 282 images were selected based on SNR for 3D RI reconstruction.

Reconstructing the HEK-cell RI is more challenging than in the experiments above because the fluorescence images have lower SNR (Extended Data Fig. 3d; Fig. R2b), as ActinGreen-labeled actin is substantially dimmer than fluorescent paint. As shown in Fig. 2, low-SNR measurements can degrade RI reconstruction due to overfitting

to noise. To mitigate this effect, we apply an *early-stopping* strategy: empirically, the model recovers physically consistent RI structure within the first several iterations, whereas prolonged optimization increasingly fits noise and degrades reconstruction quality. We therefore stop the optimization at iteration 60 (out of a maximum of 300), which also reduces reconstruction time.

By leveraging the early-stopping strategy, we reconstructed the 3D RI of HEK cells from low-SNR diffracted fluorescence images over a large volume ($198 \times 198 \times 70$ μm^3, $600 \times 600 \times 40$ voxels). Forward-model consistency supports reconstruction fidelity, which shows the predicted diffracted fluorescence images closely match the experimental measurements with a MSE of 3.5858×10^{-3} (Fig. R2b, Table S7). Orthogonal cross-sections (Fig. R2d) resolve individual HEK cells with clear, enclosed cell boundaries. Representative zoomed-in $x - y$ view further reveals membrane-scale features down to $\sim 1 - 2$ μm (Fig. R2e), demonstrating high-resolution RI recovery. Moreover, a zoomed-in RI stack distinguishes cells at different axial planes without out-of-plane crosstalk (Fig. R2f). At the volume scale, the depth-encoded projection (Fig. R2g) localizes cells across the full axial extent, highlighting the strong z-sectioning of FDT in thick samples. Together, these results demonstrate that FDT can reconstruct 3D RI in thick biological specimens using fluorescence originating from the sample itself, without transmission-side access."

The detail of the sample preparation was added to the Experimental Setup Section in the Supplementary document:

"**3D cultured HEK293T cells stained with ActinGreen 488.** HEK293T cells were resuspended in 50 μL Matrigel (Corning 354277) on ice at a density of 6.0×10^4 cells/μL. The resuspension was injected into a glass-bottomed mold 8 mm in diameter and 1 mm deep, followed by gelation at 37 °C for 1 hour. After gelation, the samples were then incubated at 37 °C overnight in Dulbecco's Modified Eagle Medium (DMEM; Thermo Fisher Scientific 11995065) supplemented with 10% fetal bovine serum (FBS; Thermo Fisher Scientific 16140071) and 1% Penicillin–Streptomycin (PS; Thermo Fisher Scientific 15140122). Samples cultured in 35-mm dishes were processed for immunocytochemistry as follows. Culture medium was removed and samples were quickly rinsed three times with phosphate-buffered saline (PBS), then fixed in 4% paraformaldehyde in PBS at room temperature for 20 min. After three additional PBS rinses, samples were either stored in PBS at 4 °C or immediately permeabilized with 0.1% Triton X-100 in PBS at room temperature for 45 min, followed by three PBS rinses. Samples were then incubated with ActinGreen 488 ReadyProbe (Thermo Fisher Scientific, R37110) at room temperature for 45 min in the dark. Finally, samples were rinsed three times with PBS, covered with PBS, and stored in the dark until imaging."

Comment 2. How should sample properties affect the reconstruction: density and spatial homogeneity of the labeling, presence of additional absorbers, distortion of the exciter beam (this is not an issue to be considered given the sample is thin) and of the collected signal in the case of slow or high spatial variation of the refractive

index (optical aberration and scattering) and presence of several spectrally distinct fluorophores, etc.?

Response. We thank the reviewer for this constructive suggestion. We have added new experimental data and discussion to point-by-point address the reviewer's concerns.

Labeling density and spatial homogeneity:

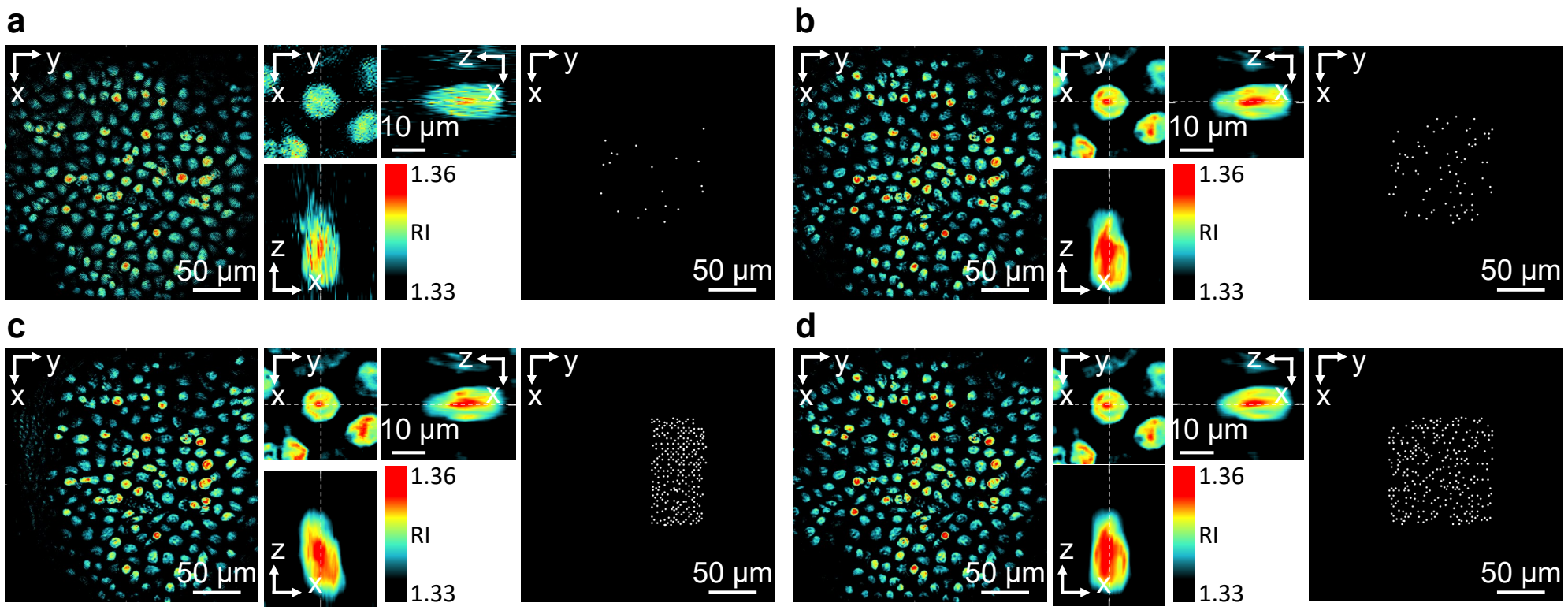

Fig. R3 Impact of sampling number and illumination-position coverage on 3D RI reconstruction of MDCK cells, with corresponding illumination-position maps. For each panel, the left image shows the reconstructed x–y slice at $z = 164$ μm (scale bar, 50 μm), the middle insets show orthogonal views (x–y, x–z, and y–z) of a representative cell with the RI color bar, and the right black-dot map shows the distribution of illumination positions used in that strategy. All illumination positions lie in the plane $z = 0$. **a,** Reconstruction using 20 measured images with the corresponding 20 illumination positions, representing sparse sampling with limited coverage. **b,** Reconstruction using 80 measured images with the corresponding 80 illumination positions. **c,** Reconstruction using only measurements from the right half-space with 300 illumination positions, representing one-sided biased coverage. **d,** Reconstruction using 300 measured images with uniform coverage and 300 illumination positions, representing dense and well-covered sampling.

We first examine the effect of sparse-to-dense sampling by comparing Fig. R3**a**, **b**, and **d**. With only 20 illumination positions (Fig. R3**a**), the x–y slice at $z = 164$ μm shows fragmented cell boundaries and reduced structural continuity; in the x–z view, a pronounced spurious scattering/artifact lobe appears near the top boundary; and in the y–z view, the high-RI nuclear region is more prone to non-physical deformation/elongation. Increasing the sampling to 80 illumination positions (Fig. R3**b**) yields a markedly cleaner reconstruction: the cell outline in the x–y slice becomes more continuous, the top-boundary artifact in the x–z view is substantially reduced. With 300 uniformly distributed illumination positions (Fig. R3**d**), the reconstruction shows the best geometric consistency across all cross-sections, with further suppression of residual top-boundary artifacts and the most clear morphology, resulting in the clearest overall 3D structure.

We then isolate the effect of lateral coverage by comparing one-sided versus full-field sampling at the same sampling count. Both Fig. R3**c** and Fig. R3**d** use 300 illumination positions; however, Fig. R3**c** uses only measurements from the $x > 0$ half-space, whereas Fig. R3**d** uses measurements that uniformly cover the full field. Despite having the same number of illumination positions, the one-sided biased sampling in Fig. R3**c** produces a pronounced tilt artifact along the x direction in the x–z view, while the full-coverage sampling in Fig. R3**d** does not exhibit such a systematic bias and maintains strong z-sectioning. These results demonstrate that lateral coverage is more critical than sampling count alone for eliminating one-sided geometric bias, whereas increasing sampling density primarily suppresses residual artifacts and improves morphological fidelity.

Presence of additional absorbers: Our work focuses on reconstructing the 3D RI of in vitro cultured cells and tissues with thicknesses up to 300 μm using FDT. The absorption coefficient of these mammalian cells and tissues is orders of magnitude lower than the scattering coefficient at visible to near-infrared wavelengths [1]. Therefore, it is reasonable to neglect absorption in our current reconstruction model, similar to most ODT/IDT studies [2–4] that reconstruct the RI of in vitro mammalian cells and tissues. Specifically, we use a differential multi-slice model to calculate light propagation through bulky tissue. As shown in Eq. 2 in Methods (Eq. R1 below), each slice of the bulky tissue is modeled as a phase object:

$$t_k(\mathbf{r}) = \exp\left(\frac{j2\pi}{\lambda}\Delta z(\hat{n}_k(\mathbf{r}) - n_b)\right), \quad k = 1, 2, \ldots, N_z. \tag{R1}$$

In future work, if strong absorbers are present within the ~ 300 μm imaging volume, such as during in vivo imaging with blood vessels near the surface, they could locally attenuate the fluorescence intensity, since the absorption coefficient of hemoglobin is several orders of magnitude higher than that of water at visible wavelength. In such cases, our multi-slice model can incorporate both absorption and scattering (Eq. R2). Each slice of the tissue would be modeled to account for both phase modulation (diffraction) and amplitude attenuation (absorption):

$$t_k(\mathbf{r}) = A(\mathbf{r})\exp\left(\frac{j2\pi}{\lambda}\Delta z(\hat{n}_k(\mathbf{r}) - n_b)\right), \quad k = 1, 2, \ldots, N_z. \tag{R2}$$

Therefore, our framework is readily extendable to account for additional absorbers. We have modified Eq. 2 in the manuscript to Eq. R2. Successful joint reconstruction of phase and absorption has been demonstrated in prior work [5, 6] using the same multi-slice model with transmission-mode IDT when imaging algae, which strongly absorb light for photosynthesis.

Refractive index spatial variations: The goal of FDT is to reconstruct the slow and high spatial variance of RI from diffracted fluorescence images. Such RI spatial variations diffract light and provide the key contrast in the diffracted fluorescence measurements. We explicitly model the spatially heterogeneous RI using the multi-slice model (Eq. R1) through the slice-wise transmission functions $t_k(\mathbf{r}), k = 1, 2, ..., N_z$. To

reconstruct the heterogeneous RI, we solve an inverse problem by our explicit neural-field estimation with local regularization. Therefore, whereas standard fluorescence imaging typically treats aberrations and scattering induced by RI heterogeneity as artifacts to be corrected, FDT (as a quantitative phase imaging approach) is designed to reconstruct the RI distribution from these diffracted images.

FDT models light diffraction under the first Born approximation [7], which is valid for reconstructing 3D RI distribution in weakly scattering samples such as the 3D cultured cells and tissues ($40 - 300\ \mu m$ thick) used in our experiments. It is not intended to recover 3D RI in regimes dominated by strong multiple scattering beyond the first Born approximation. This is a common limitation shared by ODT/IDT methods that reconstruct RI based on the first Born/Rytov approximation [2, 4, 5, 8–10]. We state this limit in the revision and outline possible extensions.

Presence of several spectrally distinct fluorophores: In our experiments, we selected the excitation wavelength to maximize the excitation efficiency of one fluorophore at a time, and we used a narrow emission band to collect fluorescence from the excited fluorophore. Therefore, even with multiplexed fluorescent staining using spectrally distinct fluorophores, only one fluorophore is efficiently excited at a time with minimal crosstalk from other color channels. The excitation and emission wavelengths used in Figs. 2–5 are listed in Table R1. Reconstruction is performed at the central wavelength of the emission filters because fluorescence can be considered as a quasi-monochromatic light source after the color filter. We neglect dispersion in the cells and tissues used in our experiments because it is weak, as also assumed in prior work on white-light diffraction tomography [4]. Specifically, as shown in Eq. R1, the 3D RI $n_k(\mathbf{r})$ is modeled as a function of spatial location $\mathbf{r}$, but not of wavelength λ. The goal of this manuscript is to demonstrate that fluorescence can be used as an illumination source to reconstruct the 3D RI of cells and tissues in reflection geometry. In future work, if samples contain multiple spectrally distinct fluorophores, we can excite them sequentially to acquire multispectral diffracted fluorescence images for RI reconstruction, analogous to white-light diffraction tomography [4]. This strategy could further improve both the resolution and the z-sectioning capability.

Comment 3. How the properties of the excitation beam should affect reconstruction: axial and lateral extension, spatial sampling when raster scanning the beam across the sample ?

Response. We thank the reviewer for this valuable comment. We have added new experimental data and discussion to address these two concerns.

Axial and lateral extension: Since the fluorescence object in our experiments is larger than the excitation focus for two-photon excitation, the larger the axial and lateral extension of the excitation focus is, the more fluorophores are excited simultaneously, resulting in brighter fluorescence images. On the other hand, since fluorescence is only spatially coherence if the size of the focus is smaller or equal to the diffraction limit, the larger the focus is, the fluorescence source becomes more spatially incoherent, reducing the contrast of fluorescence images. We experimentally measured the point spread function (PSF) of the fluorescent focus under two-photon excitation to

Table R1 Imaging parameters used in Figs. 2-5

**Parameters.**	**Fig. 2**	**Fig. 3**	**Fig. 4**	**Fig. 5**
RI sample	Silica beads	Living MDCK cells	3D bovine myotube	3D HEK293T cells
Fluorescence	Paint on the slide	Paint outside the dish	Paint outside the dish	ActinGreen 488 in cells
Exc. (nm)	850	1035	880	900
Emi. (nm)	635 ± 60	635 ± 60	635 ± 60	535 ± 40
NA_{obj}	1.0	1.0	1.0	1.0
NA_{illu}	0.79	0.54	0.44	0.52
Z (μm) [1]	140	190	240	120
Num. of images	41	441	372	282
Time/image (ms)	50	20	100	300
L (μm) [2]	172.85	71.25	56.78	45.97
Recon. volume (μm^3)	$20 \times 20 \times 20$	$264.7 \times 264.7 \times 40$	$530 \times 530 \times 300$	$198 \times 198 \times 70$
Recon. Δx (μm) [3]	0.33	0.33	0.52	0.33
Recon. Δz (μm) [3]	0.5	2	12.5	1.75
RI x resolution (μm) [4]	0.25	0.29	0.32	0.30
RI z resolution (μm) [4]	1.26	1.59	1.73	1.61

1. The distance between the fluorescence source and the imaging plane.
2. The maximum distance between fluorescent sources.
3. These are the sampling size of the reconstructed RI.
4. These are the lower bound of theoretical resolution of RI.

be $1.16 \times 1.16 \times 2.2\ \mu m^3$ (Fig. R4), which is very close to the theoretical diffraction limit of the fluorescence focus $0.55 \times 0.55 \times 1.8\ \mu m^3$ (excitation wavelength 900 nm, $NA_{obj} = 1$, Table R1). Notably, these fluorescent microspheres are used only for two-photon PSF characterization, not for RI imaging. Because the imaging system acts as a low-pass filter, the measured PSF provides an upper bound on the size of the fluorescence sources. Accordingly, the actual size of the fluorescence sources can be smaller than the measured PSF. Under the standard definition of spatial coherence, a source whose effective extent is at or below the diffraction limit is spatially coherent across the collection pupil and can be modeled as emitting a well-defined wavefront. We therefore model the fluorescence source excited by each two-photon excitation focus as a single effective point emitter. We have added this result (Fig. R4) to the revised manuscript.

If the excitation focus is extended significantly in the axial and lateral directions and it is much larger than the diffraction limit, the excitation focus can be modeled as an incoherent summation of multiple point sources with similar intensity. This is a standard approach that has been used to model a LED array as an extended light source of multiple single LEDs in IDT [5]. If the excitation focus is extended to be slightly larger than the diffraction limit, it is considered as an incoherent sum of multiple point sources where the center point source is much brighter than the other ones, which cannot be modeled as an array of similar point sources. In this case, a weighting factor (i.e., partial coherent masks) is used in the reconstruction process to emphasize the contribution from the brightest point source in the center while downplaying the contribution from the dim sources to accelerate convergence.

Spatial sampling when raster scanning the beam across the sample: Both the number and distribution of spatial sampling affect the reconstruction (Fig. R3). With a

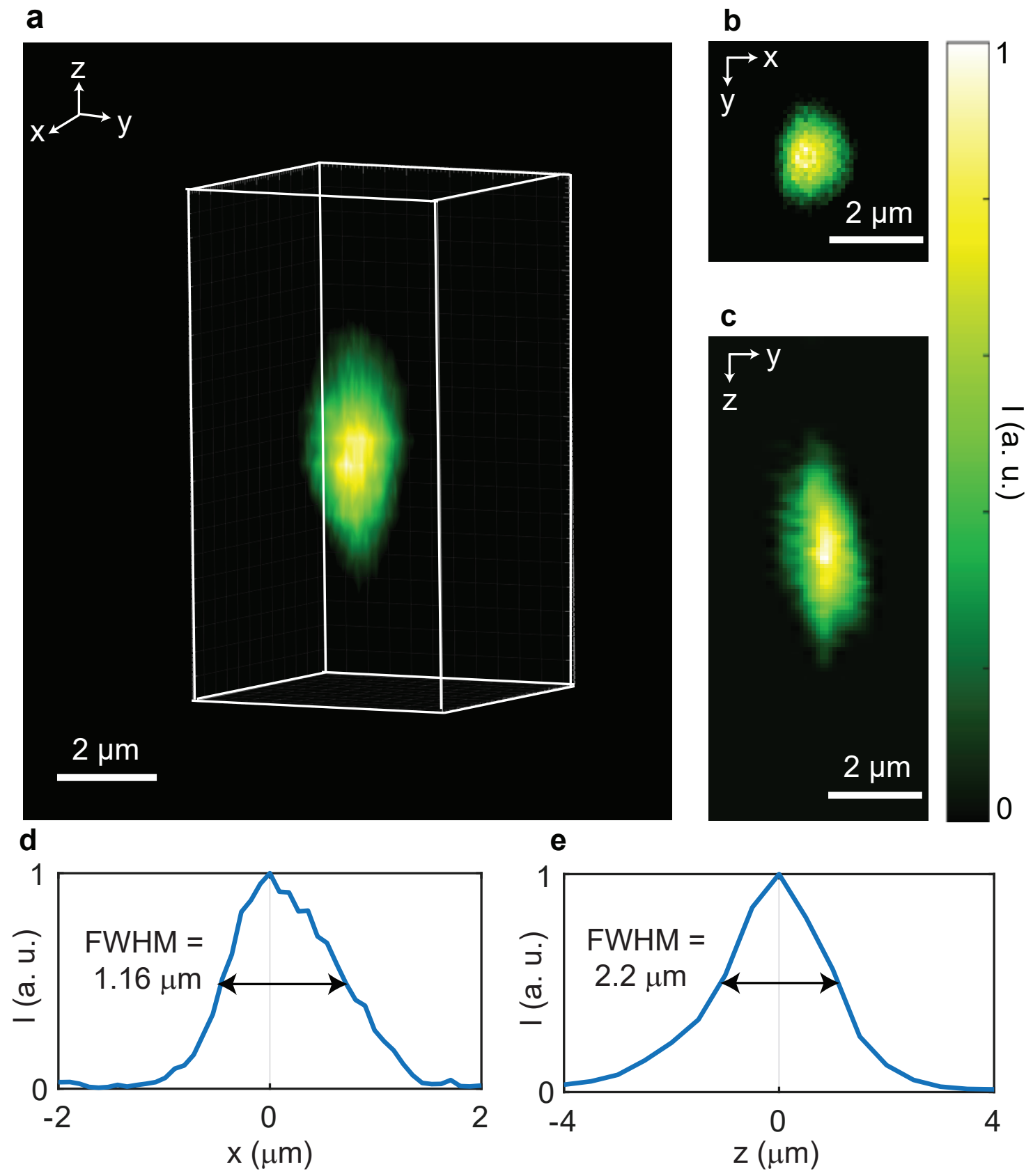

Fig. R4 3D point spread function (PSF) of a fluorescence illuminator under two-photon excitation. a. Volume view of the fluorescence PSF. **b-c. (b)** x–y and **(c)** y–z cross-sections of the fluorescence PSF. **d-e.** Line profiles in the **(d)** lateral and **(e)** axial directions, showing the full width at half maximum (FWHM) of the fluorescence PSF.

homogeneous fluorescence labeling, the scanning positions of excitation light determine the positions of fluorescence sources. Under the same distribution, such as random uniform distribution, a denser spatial sampling will fill up the optical transfer function (OTF, Fig. R5c) more, therefore reducing high-frequency noise and aliasing artifact due to undersampling (Fig. R3a-b, d). On the other hand, a denser spatial sampling requires longer image acquisition time and computational time. With the same number of sampling points, a uniform distribution will provide uniform perspective angles for the tomographic reconstruction, resulting in less distortion artifact from a biased distribution (Fig. R3c-d). More discussion about spatial sampling for beam scanning is provided in the response to Comment 9.

Comment 4. How would the detection configuration affect the reconstruction: numerical aperture in collection, spatial sampling with the camera, vignetting of the fluorescence signal along the detection path?

Response. We thank the reviewer for this important question. The detection configuration directly affects reconstruction quality in the following ways:

Numerical aperture (NA) in collection: The NA in collection affect the 3D resolution of reconstructed RI. We quantitatively show the relation between the NA and the spatial resolution by plotting the OTF on the Ewald's sphere (Fig. R5c). The NA in collection is related to both the objective NA ($NA_{obj} = nsin(\theta_{obj})$) as well as the illumination NA ($NA_{illu} = nsin(\theta_{illu})$) due to oblique illumination, where n is the average RI of the sample and immersion media ($n = 1.33$). As defined in prior work [5, 11–13], the lateral bandwidth Δu_x of OTF in a ODT/IDT imaging system is determined by the sum of NA_{obj} and NA_{illu}:

$$\Delta u_x = 2(NA_{obj} + NA_{illu})/\lambda, \tag{R3}$$

where $NA_{obj} = nsin(\theta_{obj})$, $NA_{illu} = nsin(\theta_{illu})$, and n is the average RI of the sample and immersion media ($n \approx 1.33$). The axial bandwidth is defined as

$$\Delta u_z = n(2 - cos(\theta_{obj}) - cos(\theta_{illu}))/\lambda. \tag{R4}$$

We can rewrite Eq. R4 in the form of NA_{obj} and NA_{illu}:

$$\Delta u_z = (2n - \sqrt{n^2 - NA_{obj}^2} - \sqrt{n^2 - NA_{illu}^2})/\lambda. \tag{R5}$$

Therefore, the higher NA in collection is, the higher resolution FDT can achieve in the reconstruction. The objective NA in this work is 1.0. The illumination NA in Fig. 2-5 ranges from 0.42 to 0.79 measured from the diffracted fluorescence images (Table R1) since it is varies with the fluorescence source distribution and excitation positions. Consequently, the theoretical resolution of reconstructed RI is 0.25-0.32 μm in the lateral direction and 1.26-1.73 μm in the axial direction. More discussion of the resolution is provided in the response to Comment 7.

Camera spatial sampling: Camera spatial sampling defines the sampling of the diffracted fluorescence images, therefore affects the following RI reconstruction. If the camera spatial sampling is too low, the resolution of reconstructed RI will be reduced. On the other hand, if the camera spatial sampling is too high, the reconstruction speed will be slow. Therefore, a proper camera spatial sampling is necessary to balance the reconstruction resolution and speed. In all experiments, the camera spatial sampling is 0.33 μm/pixel, which is determined by the pixel size of the camera and the magnification of the detection path (6.5 $\mu m/20 \approx 0.33$ μm). The camera spatial sampling is also close to the theoretical resolution of the reconstructed RI. More discussion about spatial sampling for detection is provided in the response to Comment 9.

Vignetting Vignetting typically introduces position-dependent attenuation of the fluorescence signal across the detection field of view (FOV), leading to reduced reconstruction quality near the image boundaries. However, as shown in Fig. R6, the fluorescence-illuminated region in our system occupies only a small central area within

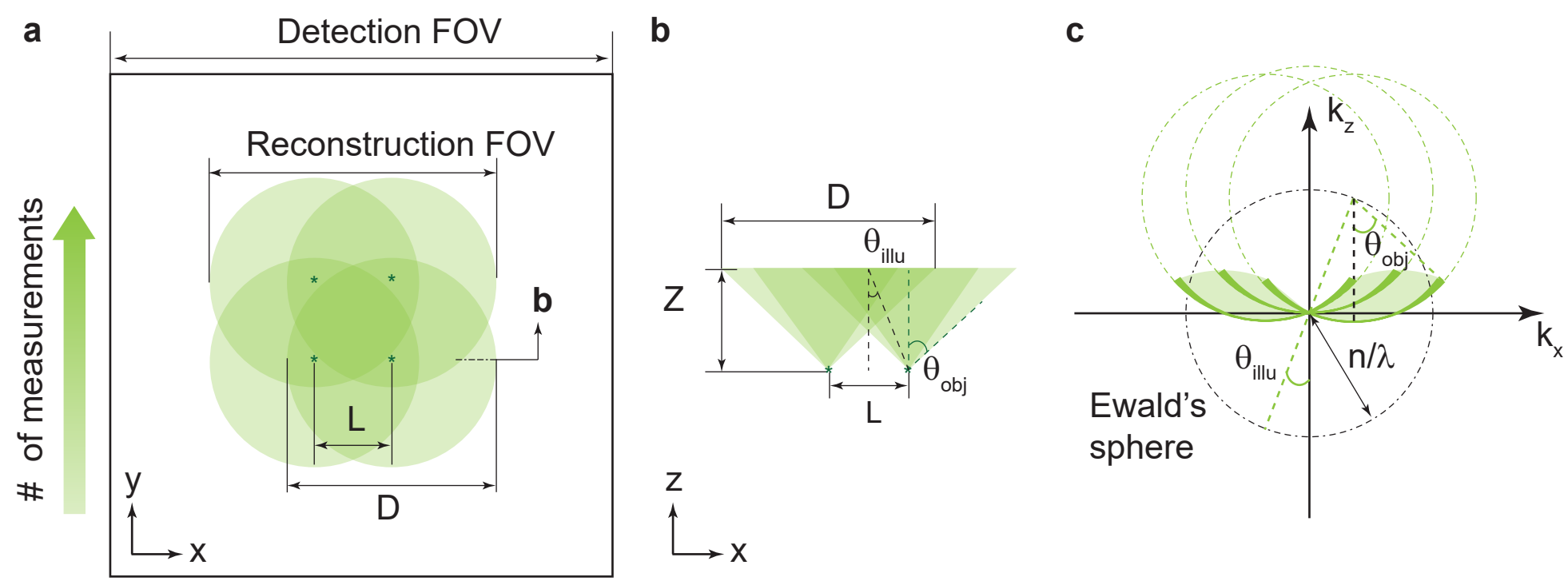

Fig. R5 Schematic diagram of the relationships among field of view (FOV), objective and illumination numerical apertures (NA_{obj} and NA_{illu}), optical transfer function (OTF), and the number of measurements. a. The x–y cross-section at the image plane shows the relationship among the detection FOV, reconstruction FOV, the diameter of the fluorescence-illuminated area D, and the maximum separation between fluorescence sources L. The central region is naturally sampled more frequently than the peripheral regions. **b.** The x–z cross-section along the fluorescence sources in **(a)** shows the relationship among D, L, the separation between the image plane and the fluorescence sources (Z), the objective lens collection angle (θ_{obj}), and the illumination angle under a fluorescence source (θ_{illu}). **c.** The OTF under three different illumination angles. The spatial-frequency coverage at different illumination angles corresponds to shifts in the OTF. The OTF becomes more completely filled with more measurements, resulting in fewer aliasing artifacts. The resolution is determined by θ_{obj}, θ_{illu}, the wavelength (λ), and the average refractive index (n).

the full detection FOV. The effective illumination region is substantially smaller than the camera-limited detection FOV. Therefore, vignetting near the edge of camera-limited detection FOV is not a problem in our experiments. As each fluorescence source generates a spherical illumination, the SNR in the peripheral region is lower than the central region of the spherical illumination. In addition, within the reconstructed FOV, the central region is captured by more fluorescence measurements than the edge of the reconstructed FOV (Fig. R5). Therefore, the slight degradation observed near the reconstruction boundaries is primarily due to limited angular sampling and reduced signal-to-noise ratio on the edge rather than optical vignetting. More discussion about the reconstructed FOV is provided in the response to the next question.

Comment 5. What limits the size of the field of view? There seems to be more artifacts at the edge of the field of view? I assume this is due to the diffracted pattern being progressively incompletely detected as you move away from the optical center?

Response. In our system there are two types of field-of-view (FOV) (Fig. R5a). First, the detection FOV is determined by the size of field stop and the objective's magnification. In our detection path, the field stop is the physical size of the camera sensor (15.6 $mm \times$ 15.6 mm, Kinetix22, Teledyne), and the objective magnification is 20× (XLUMPLFLN20XW, Olympus). Therefore, the detection FOV is $15.6/20 = 780\ \mu m$. This detection FOV is the maximum region from which we can collect light through the detection path, and all diffracted patterns are fully captured within it in our experiments. (Fig. R6).

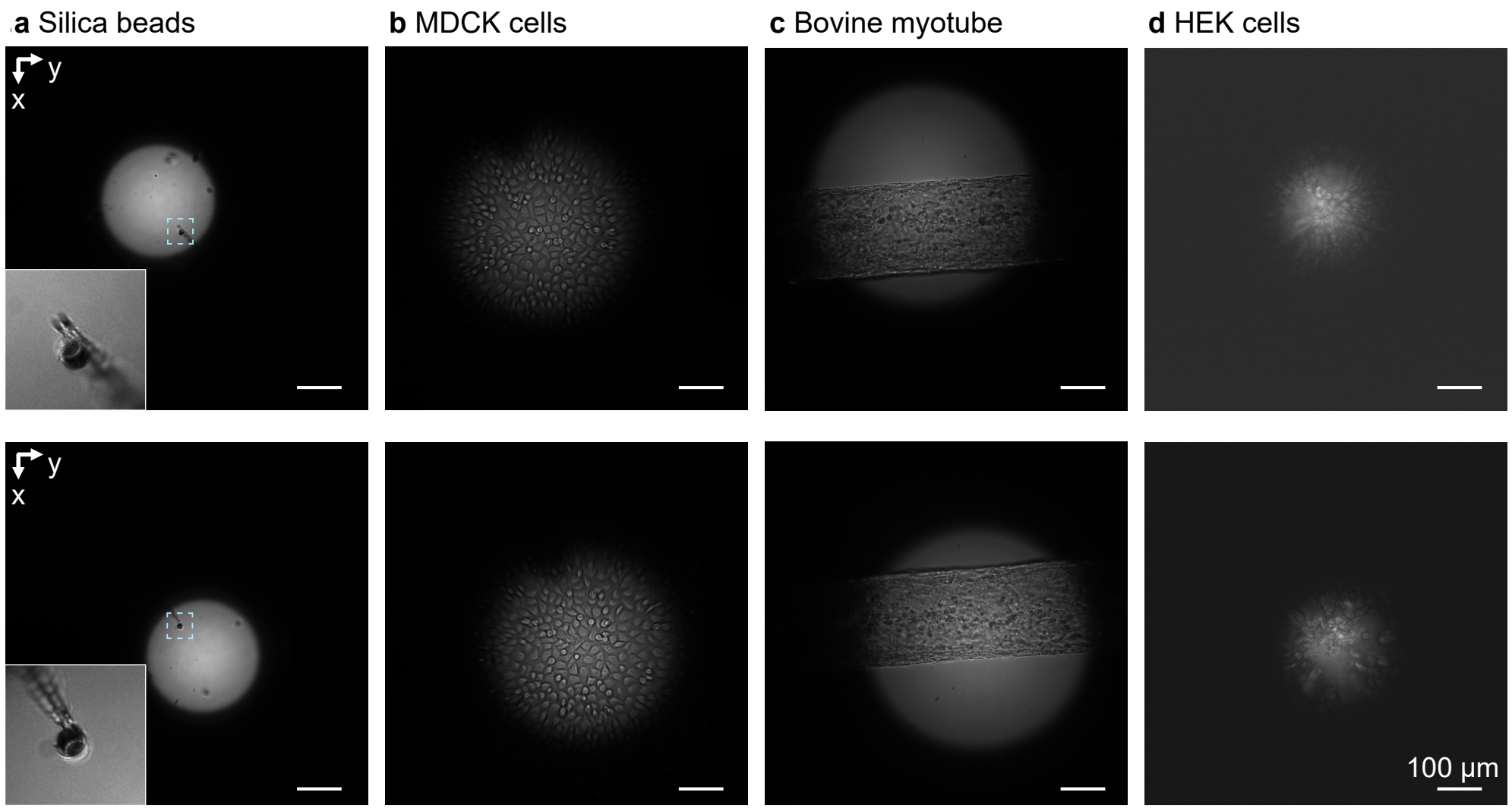

Fig. R6 Representative raw diffracted fluorescence images of experimental samples illuminated by two fluorescence sources at different illumination angles. a. Silica beads. b. MDCK cells. c. Bovine myotube. d. Fluorescence-labeled HEK cells.

Second, the reconstruction FOV is the region over which the RI can be reconstructed, which is what the reviewer refers to. The reconstruction FOV is smaller than the detection FOV and becomes the effective FOV in the reconstructed results. As in other tomographic techniques, FDT requires a sufficient number of measurements of the same object under different illumination angles to reconstruct the 3D object. Consequently, regions that are captured in more fluorescence images are reconstructed more accurately than regions captured in only a few images. This naturally makes the reconstructed RI more accurate near the center of the reconstruction FOV than near its edges, because the center is always covered by more measurements regardless of the total number acquired. Therefore, the RI artifacts near the edge of the reconstruction FOV are caused by the progressive decrease in the number of fluorescence images toward the edge (Fig. R5a-b), rather than by progressively incomplete detection of the diffracted patterns (i.e., a limitation of the detection FOV).

The reconstruction FOV varies case by case, as listed in Table R1. It is related to the diameter of the region illuminated by each fluorescent source (D) and the maximum separation between fluorescence sources (L) (Fig. R5a-b). The upper limit of the reconstruction FOV is $D + L$. However, because the number of measurements decreases toward the edges, the effective FOV with accurate RI reconstruction is typically smaller than this upper limit. Increasing D can enlarge the upper limit of the reconstruction FOV, but it can reduce the SNR of the diffracted fluorescence images because the source power is distributed over a larger area. Using bright fluorophores can help increase the SNR. Increasing L can also enlarge the upper limit, but it reduces the region that receives multiple measurements for a fixed total number

of measurements. Increasing the total number of measurements can improve reconstruction quality. However, the excitation-foci positions are partly determined by the fluorescent staining pattern, so it may not always be possible to acquire many more measurements. Therefore, the effective FOV of the reconstructed RI reflects a trade-off among these factors.

Comment 6. Detection efficiency decreases progressively with depth in multiphoton microscopy because for each point source in the sample, the signal seems to come from an extended surface and the extension of this apparent surface increases with depth (https://pmc.ncbi.nlm.nih.gov/articles/PMC3482884/). I assume this will also affect the quality of the reconstruction? Does this mean that higher scattering coefficients require higher collection NAs? Does this model also take into account the fact that a part of the forward scattered light is redirected in epi?

Response. We thank the reviewer for the helpful comment. If the "extended surface" mentioned by the reviewer refers to the illuminated area produced by a defocused fluorescence source (i.e., the circular region with diameter D in Fig. R5a), we agree that D increases with the separation between the fluorescence source and the imaging plane (Z, Fig. R5b) for a given objective NA ($NA_{\mathrm{obj}} = nsin(\theta_{obj})$). A larger D can enlarge the reconstruction FOV if the SNR of the fluorescence images is sufficient, enabling reconstruction over a larger FOV as discussed in our previous response. On the other hand, a larger D reduces the SNR of the fluorescence images because the source power is distributed over a larger area. We evaluated how the SNR of diffracted fluorescence images affects reconstruction quality (Fig. 2) and found that FDT can accurately reconstruct the 3D RI when the image SNR is above $\sim 5 - 10$ (Fig. 2). Therefore, even if the SNR decreases as Z (and thus D) increases, as long as it remains reasonably high, FDT can still accurately reconstruct the 3D RI.

Regarding the detection-efficiency decrease caused by scattering discussed in the work cited by the reviewer (Zinter, J. and Levene, M., Optics Express, 2011), it differs from our case in several important ways. First, the primary issue addressed by Zinter and Levene is that strong scattering significantly alters fluorescence-photon trajectories, whereas scattering in our samples is very weak. Here, we use "scattering" to refer to multiple scattering beyond the regime that can be modeled by FDT. Although the in vitro cells and tissues imaged by FDT are relatively thick (40-300 μm), they are much less scattering than ex vivo tissues of comparable thickness. As a result, we can model the propagation of fluorescence through the volume and infer the spatial RI variations that give rise to diffraction. Second, Zinter and Levene use a single-pixel detector (a photomultiplier tube, PMT) and form images by point scanning, whereas our method uses a camera to capture the diffracted fluorescence pattern on a defocused plane. In our framework, fluorescence sources act as illuminators rather than imaging targets. FDT is not designed for strongly scattering regimes where even detecting the fluorescence focus with a PMT becomes challenging. Therefore, unlike in Zinter and Levene's work, scattering is not the main cause of reduced detection efficiency in FDT, because FDT operates in non-scattering or very weakly scattering samples.

For samples with higher scattering than those in this work but still within the weak-scattering regime, an objective with a higher collection NA will increase the total number of photons collected. For a given D, a higher collection NA will reduce Z (Fig. R5), which increases the photon flux per unit area and thus improves the SNR of the diffracted fluorescence image. At a fixed depth Z, however, a higher collection NA also increases D (Fig. R5), which tends to spread the signal over a larger area. Therefore, the photon flux per unit area is the same. To improve detection efficiency, using a higher collection NA together with a smaller Z can increase the SNR of the diffracted fluorescence image.

(Does this model also take into account the fact that a part of the forward scattered light is redirected in epi?) If the "forward-scattered light" refers to the near-infrared excitation light, it will be blocked by the dichroic mirror and the color filter in the detection path as its wavelength is different from the wavelength of fluorescence. Therefore, even the excitation light is redirected in epi, it will not be detected and does not affect the diffracted fluorescence images.

If the "forward-scattered light" refers to fluorescence photons initially propagating away from the objective lens and then redirected in epi, we would like to discuss it case by case. In the experiments in Figs. 2-4, where fluorescent paint is applied to the outside of the Petri dish/slide, there is no scattering medium below the fluorescent layer (only air). Therefore, fluorescence photons initially propagating away from the objective are not redirected back into the epi-detection path. In the newly added experiment in Fig. 5 (Fig. R2), although there are cells below the excited cells that serve as fluorescence sources, the cell density is low (i.e., the sample remains weakly scattering), so the probability that photons propagating away from the objective are redirected back and then collected is very small. Even in more strongly scattering ex vivo tissues, only about 10% photons is redirected from its original direction [1], and part of the redirected photons may not be collected by the objective lens as well. Moreover, the total photon budget from fluorescence sources in FDT is substantially lower than that of LEDs or laser diodes used in standard ODT/IDT, so the contribution of such redirected photons is negligible. For these reasons, our model does not include fluorescence photons that initially propagate away from the objective and are subsequently redirected back toward it.

Comment 7. Ability to distinguish small details ? I suppose we can not define a proper spatial resolution as for incoherent imaging ?

Response. FDT is a quantitative phase imaging technique, specifically, a tomography imaging technique. FDT uses fluorescence light as the illumination source, which is a partial/incoherent light source similar to the LEDs and other low-coherent light sources used in prior ODT/IDT work. Even though these low-coherence light sources have a broader spectrum bandwidth than a coherent single-wavelength laser, the spatial resolution is typically computed from the OTF using the center wavelength λ. The spatial resolution of reconstructed RI to distinguish small details is defined in previous ODT/IDT work [5, 11–13]. The 3D bandwidth of OTF is defined in Eq. R3, R4, R5. Correspondingly, the Nyquist sampling distance is the lower bound for an achievable

resolution, which is the reciprocal of the frequency bandwidth:

$$\Delta x = \lambda/2(NA_{obj} + NA_{illu}),$$
$$\Delta z = \lambda/(2n - \sqrt{n^2 - NA_{obj}^2} - \sqrt{n^2 - NA_{illu}^2}). \tag{R6}$$

The lower bound of the theoretical resolution (i.e., the Nyquist sampling distance) of reconstructed RI is 0.25-0.32 μm in the lateral direction and 1.26-1.73 μm in the axial direction, which vary with the illumination angles. We have added new simulated results to quantitatively evaluate the RI resolution using FDT in the Result section. For the reviewer's reference, we include below the newly added paragraph and the revised Fig. 2 related to RI resolution.

"To quantitatively evaluate the resolution of RI reconstruction using FDT, we next reconstruct the 3D RI of simulated microbeads with known RI. The theoretical lower bounds of the resolution are determined by the objective numerical aperture, NA_{obj} ($NA_{obj} = 1$), and the illumination numerical aperture, NA_{illu}, according to prior work [5, 11–13]: $\Delta x = \lambda/[2(NA_{obj} + NA_{illu})]$ in the lateral dimension and $\Delta z = \lambda/(2n - \sqrt{n^2 - NA_{obj}^2} - \sqrt{n^2 - NA_{illu}^2})$ in the axial dimension. In FDT, NA_{illu} is determined by the fluorescence-source positions and the detection plane ($NA_{illu} = n \sin(\theta_{illu})$, Extended Data Fig. 2). In the experiments, the measured NA_{illu} ranges from 0.42 to 0.79, resulting in theoretical lower-bound RI resolutions of 0.25-0.32 μm laterally and 1.26-1.73 μm axially. Under the same parameters, the PSF of the reconstructed RI for a ~ 1 μm-diameter microbead is 0.20 μm laterally and 1.31 μm axially (Fig. R7e, left), indicating that FDT achieves high-resolution RI reconstruction. The corresponding modulation transfer function (MTF) (Fig. R7e, right) shows that FDT fills the "missing cone," thereby achieving excellent z-sectioning capability, as also demonstrated in the "UCDavis" result. We further reconstruct the 3D RI of two adjacent 3 μm-diameter microbeads with different lateral and axial separations (Fig. R9f). As the lateral separation between the two microbeads increases from 0.1 μm to 0.3 μm, the reconstructed RI changes from connected to fully separable. Similarly, for two microbeads overlapping along the axial direction, as their separation increases from 0.5 μm to 1.5 μm, the reconstructed RI changes from inseparable to separable. These results further demonstrate that FDT enables high-resolution 3D RI reconstruction with good z-sectioning ability."

Comment 8. What is conceptually limiting the imaging depth ?

Response. We thank the reviewer for this important question. The imaging depth is conceptually limited first by the weak-scattering condition underlying the multi-slice model based on the first Born approximation [8, 14]. The Born approximation is valid when the total phase delay induced by the specimen is less than $\pi/2$ [8]. Therefore, bulky tissue must be modeled as multiple thin slices so that the diffraction at each slice remains within the validity regime of the Born approximation. As the imaging depth increases, both the number of axial slices and the reconstruction FOV increase (Fig. R5), leading to an approximately cubic growth in the number of voxels to be

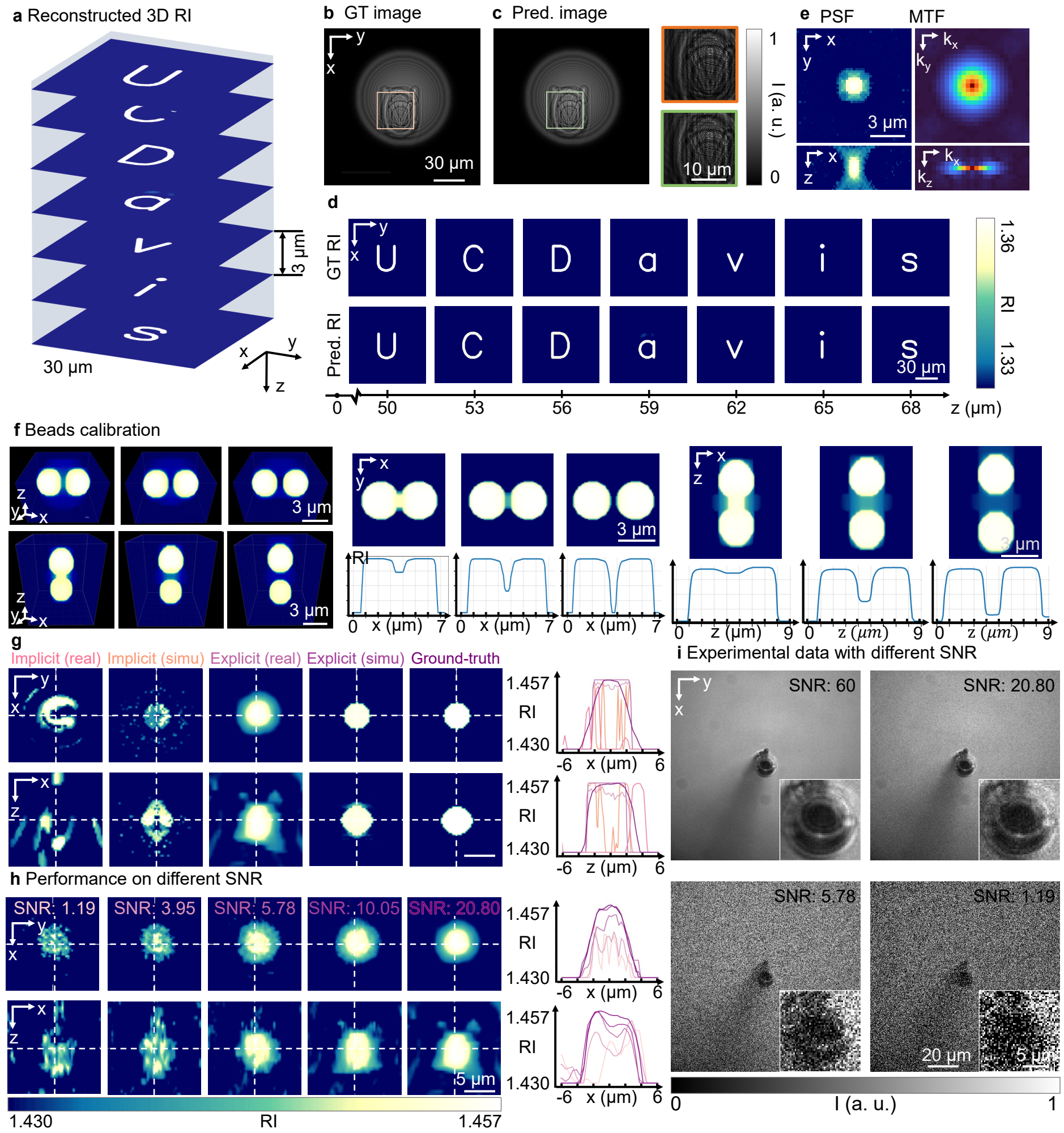

Fig. R7 Reconstruction of the 3D RI of a simulated "UCDavis" pattern. a, Reconstructed 3D RI distribution of the "UCDavis" pattern in 3D. **b,** A representative ground-truth (GT) image. **c,** Predicted fluorescence image together with zoomed-in views of the regions highlighted by the orange and green boxes. The MSE between the GT and predicted images and RI are 6.1125×10^{-7} and 1.5730×10^{-6}, respectively. **d,** Comparison of the ground-truth RI (top row) and the predicted RI (bottom row) on each z-plane, as indicated by the axis below. **e,** Simulated point spread function (PSF, left) and modulation transfer function (MTF, right) of the reconstructed 3D RI of a 1 μm-diameter microbead using FDT. **f,** Volume renderings, cross-sections, and line profiles of the reconstructed 3D RI of two microbeads that are laterally adjacent or axially overlapping at different separations. The lateral separations are 0.1 μm, 0.2 μm, and 0.3 μm, and the axial separations are 0.5 μm, 1 μm, and 1.5 μm. **g,** Comparison of the 3D RI of silica beads reconstructed using explicit and implicit neural fields. From left to right: implicit neural fields (experiment), implicit neural fields (simulation), explicit neural fields (experiment), explicit neural fields (simulation), and ground truth (simulation). The dotted lines in the images mark the x- and z-directions along which the line profiles are plotted. **h,** Comparison of the reconstructed 3D RI using forward images at various signal-to-noise ratios (SNRs), ranging from 20.80 down to 1.19. The dotted lines again indicate the x- and z-directions along which the line profiles are extracted. **i,** Forward images of the same beads at different SNR levels. The top-left image (SNR = 60) is used for the reconstruction in **g**, whereas the other three images are used for the reconstruction in **h**.

reconstructed. Consistent with these considerations, state-of-the-art reflection-mode ODT/IDT methods based on the weak-scattering assumption and using visible light have demonstrated maximum imaging depths of less than 500 μm in biological tissue [15]. This limitation could be alleviated by more efficient computational algorithms and more powerful computing resources.

Second, the imaging depth of FDT is limited by the SNR of the diffracted fluorescence images. As the fluorescence source moves deeper into the sample, its emission spreads over a larger area at the detection plane (Fig. R5), reducing the photon flux per unit area and thus decreasing image SNR. Therefore, the allowable separation between the fluorescence source and the detection plane is constrained by fluorophore brightness and detection sensitivity. This limitation could be mitigated by imaging at multiple planes at different depths, rather than relying on a single detection plane at the sample surface, or by using brighter fluorophores and a more sensitive camera. In addition, as imaging depth increases, scattering and aberrations progressively degrade the two-photon excitation focus, reducing signal strength and increasing out-of-focus background. Under such conditions, new methods will be needed to model the speckled excitation field. More discussion about the scattering of two-photon excited focus is provided in the response to Comment 11. We have added this discussion in the Discussion section of the revised manuscript.

Comment 9. How does the acquisition parameters will influence the reconstruction quality: spatial sampling for beam scanning and detection, SNR, camera type of noise ect. . .

Response. We thank the reviewer for this important comment. The spatial sampling of beam scanning and the fluorescence-labeling density together determine the distribution of fluorescence sources, because the fluorescence sources are generated by excitation of fluorophores with the scanned beam. Under homogeneous fluorescence labeling, we have the flexibility to choose the spatial sampling for beam scanning, as listed in Table R1. In the revised manuscript, we have added new experimental results showing how different beam-scanning sampling strategies affect the reconstruction (Fig. R3). Denser beam-scanning sampling leads to more accurate reconstruction because it fills the OTF more completely (Fig. R5), thereby reducing aliasing caused by insufficient Fourier-domain sampling. Under inhomogeneous fluorescence labeling (Fig. 5 and Fig. R2), some scanned positions may not generate fluorescence if no fluorophores are present. Even in this case, denser beam-scanning sampling improves reconstruction in the labeled regions because it provides better OTF coverage. More details are provided in our responses to Comments 2 and 3.

The spatial sampling in detection is determined by the camera pixel size and the objective magnification. In our experiments, the camera pixel size is $6.5 \times 6.5\ \mu m^2$ and the objective magnification is 20×, resulting in a detection sampling interval of 0.33 μm. We chose this detection sampling because the Nyquist-limited lateral resolution is 0.25–0.32 μm (Eq. R6), and the objective lens is designed to operate at 20× for optimal aberration correction. Reducing the detection sampling density would cause undersampling, leading to aliasing and loss of high-frequency information such as

intracellular structures. In contrast, increasing the detection sampling density would lead to oversampling. Oversampling would not recover additional high-frequency information, but it would increase camera noise per effective feature because photons are distributed over more pixels, and it could also reduce the detection and reconstruction FOV if the finer sampling were achieved by increasing magnification. Therefore, we optimized the detection sampling for the best reconstruction performance. More details are provided in our response to Comment 4.

FDT also requires sufficient signal-to-noise ratio (SNR) to accurately reconstruct the 3D RI. As shown in the experimental results in Fig. 2 (Fig. R7), accurate reconstruction is achieved when the SNR is sufficiently high (SNR $> 5 - 10$). We have discussed the parameters that affect the SNR of the raw measurements in our responses to Comments 4–6 and 8. In the section on the new HEK-cell data (response to Comment 1), we also discuss strategies for handling low-SNR raw measurements during reconstruction without overfitting high-frequency noise.

For image acquisition, we used a scientific CMOS camera (Kinetix22) with a read noise of $0.7e^-$. Camera noise is one of several noise sources that affect image quality, and in general, lower camera noise improves the SNR. In our experiments, however, we focus on the overall SNR rather than on individual noise components. As long as the SNR is sufficiently high (e.g., SNR $> 5 - 10$), FDT can accurately reconstruct the 3D RI.

Comment 10. "Our data demonstrate that explicit neural fields not only accelerate computation but also deliver significantly enhanced reconstruction quality. Therefore, we use explicit neural fields for high-resolution 3D reconstruction of RI." Could the authors better justify why "explicit neural fields" would be conceptually better suited to this task?

Response. We thank the reviewer for giving us the opportunity to clarify why the explicit neural fields conceptually work better for the 3D RI reconstruction. Here, we would like to focus on comparing the explicit neural fields to the implicit neural fields that have been used for 3D RI reconstruction [6]. In our setting, explicit neural fields denote a locally parameterized, multi-pixel-resolution representation of the RI volume, whereas implicit neural fields use fully implicit coordinate multilayer perceptrons (MLPs) to represent the RI volume. Since the RI in biological samples is various locally (the RI in one location is independent from the RI in a distant position), representing the RI with a local, explicit parameterization yields better numerical conditioning and more stable Gauss–Newton/first-order updates than global MLPs whose parameters entangle distant voxels.

Second, explicit fields enable the use of physically meaningful regularizers for direct, voxel-wise or patch-wise regularization. In contrast, these physically meaningful regularizers are harder to enforce on implicit MLP weights. As the regularization acts directly on the field, explicit models are more robust to noise in the low SNR images and model mismatch than implicit neural fields. This is reflected in more stable reconstructions under low SNR scenarios (Fig. 2).

Third, during the training process, we have implemented the coarse-to-fine structure (Fig. 1a) for solving the aliasing artifact. Such coarse-to-fine structure can be directly applied to explicit neural fields since the explicit representation allows us to flexibility change the representation resolution, while it is hard to be applied to the implicit neural field. Furthermore, explicit neural fields enables faster, parallel optimization due to the explicit structure, whereas implicit neural fields require back-propagation through deep networks for every coordinate query. Consequently, explicit neural fields reconstruct the 3D RI much faster (minutes) than the implicit ones (hours), which allows explicit neural fields to be expanded for resolving RI over a large volume.

We have added the discussion in the Introduction section. We have experimentally compared the reconstruction results using explicit neural fields and the implicit ones (Fig. 1f, Fig. 2, Fig. 3). We also included ablation studies showing that explicit fields achieve higher reconstruction fidelity and lower runtime at matched compute compared to implicit ones (Table S4).

Comment 11. The authors use two-photon absorption to obtain the emission from a single point source. As I understand it, the validity of this assumption is crucial for faithful reconstruction. However, for increasing imaging depth, the signal from the sample surface will progressively increase relative to the signal from the focus (https://doi.org/10.1364/JOSAA.23.003139). How will this affect the estimation of the refractive index, since this assumption of localized signal emission is progressively not respected?

Response. We thank the reviewer for the comment. We would like to clarify that the imaging samples in this work (i.e., in vitro cells) are non-scattering or weakly scattering, and our imaging depths are not as large as those discussed in Theer and Denk (2006) [16]. Specifically, the samples in Fig. 3 are 2D-cultured MDCK cells with a thickness of approximately 44 μm placed in a coverslip-bottom dish (Fig. R1a). The fluorescent paint is applied to the outside of the dish. The space between the bottom of the cells and the fluorescent paint is a coverslip with a thickness of approximately 170 μm. The sample in Fig. 4 is a 3D-cultured bovine myotube with a thickness of approximately 300 μm placed on a dish with fluorescent paint on the outside surface (Fig. R1b). The bovine myotube is cultured in Matrigel. The sample in Fig. 5 (Fig. R2) is 3D-cultured HEK293T cells in Matrigel, and the fluorescence sources are from cells located approximately 100 μm below the surface (Fig. R1c). All these samples are non-scattering or weakly scattering. Furthermore, the main background media in these samples are either water ($RI = 1.33$) or Matrigel ($RI = 1.34$), which match the refractive index of the water-immersion objective ($RI = 1.33$) well. As noted in the reference [16], “for a sufficiently large imaging depth, significant fluorescence is generated near the top of the sample”, where “sufficiently large” refers to several scattering mean-free-paths (MFPs), i.e., depths close to the limit of standard two-photon microscopy where in-focus two-photon point-scanning images have low contrast. In contrast, the imaging depths in our manuscript is far shorter than the “sufficiently large imaging depth”, since the defocused diffracted fluorescence images acquired by the camera still have sufficient SNR for RI reconstruction. Therefore, it is valid to

assume that the fluorescence sources can be modeled as localized point sources for the samples and imaging depths used in this work.

In future work, if the imaging depth becomes sufficiently large that substantial fluorescence is generated near the top of the sample, this fluorescence will contribute background noise to diffracted fluorescence images acquired at deeper planes, because the imaging plane is moved further into the sample as the imaging depth increases (Fig. 4; see also our response to Comment 8). In this regime, fluorescence generated near the surface (including regions illuminated by surface fluorescence) is outside the depth of field when the imaging plane is deep within the sample. Consequently, it contributes to background rather than signal in the captured image. This is similar to standard two-photon point-scanning microscopy, where out-of-focus fluorescence contributes background that reduces image contrast [16]. Accordingly, rather than modeling surface fluorescence as additional fluorescence sources, we treat it as background noise. The signal in the diffracted fluorescence images still arises from illumination by point sources within the tissue below the imaging plane. As long as the SNR of the diffracted fluorescence images remains above 5-10 (Fig. 2), FDT can accurately reconstruct the RI from these fluorescence images.

Comment 12. Figure 2, panel g). How do the authors interpret the variations in shape between SNRs 5 and 20? Does performance seem to deteriorate at higher SNRs?

Response. We thank the reviewer for this helpful comment. We agree that the reconstructed RI of the silica beads shows artifacts when using experimentally measured diffracted fluorescence images with SNRs of 5.78 and 20.8, compared with the ground-truth RI of the beads. Reconstructing silica beads from experimental measurements using FDT is challenging due to the large RI differences and self-focusing effects induced by the bead. The artifacts differ between the low-SNR and high-SNR cases. When using diffracted fluorescence images with an SNR of 5.78, the reconstruction slightly overfits the high-frequency noise present in the low-SNR images. As a result, the artifacts appear as loss of low-frequency components together with high-frequency, speckle-like patterns. In contrast, when using diffracted fluorescence images with an SNR of 20.8, the artifacts arise mainly from the limited frequency support of the OTF in the k_z direction. Although low-coherence illumination (i.e., fluorescence sources) helps fill the missing cone, the spatial-frequency coverage in the k_z direction remains lower than that in the k_x direction and limited by the finite number of raw measurements, leading to lower axial resolution than lateral resolution and slight artifacts. Consequently, the reconstructed bead appears elongated along the axial direction with slight sidelobes. In terms of robustness to SNR, the reconstruction from images with SNR = 20.8 is not noticeably limited by noise and instead mainly reflects the OTF of the imaging system, whereas the reconstruction from images with SNR = 5.78 exhibits mild noise-induced high-frequency artifacts. For SNRs below 5.78, the reconstructions exhibit pronounced high-frequency artifacts dominated by noise.

Comment 13. The authors mentioned at several places in the manuscript multimodal fluorescence and phase imaging, for example: “FDT is a critical step toward multimodal fluorescence and phase microscopy for bulky tissue and ultimately for in

vivo imaging in the future". Can the authors demonstrate or explain how they would efficiently and conveniently obtain both 3D IR and fluorophore distribution?

Response. We thank the reviewer for this question regarding multimodal imaging. We previously demonstrated multimodal fluorescence and RI imaging in our prior work [17], termed three-dimensional bi-functional refractive index and fluorescence microscopy (BRIEF). In that framework, fluorescence images are reconstructed by localizing fluorescence sources, analogous to how super-resolution localization microscopy reconstructs fluorescence images [18, 19]. This process is also closely related to the self-calibration of fluorescence illumination described in the section "Self-calibration of fluorescent illumination" in the Methods. Specifically, the goal is to localize each fluorescence source from its corresponding defocused fluorescence image (Fig. R6). The initial position $\hat{\boldsymbol{p}}_{\boldsymbol{i}}$ is estimated by fitting a 2D Gaussian function to the defocused fluorescence image. The mean of the 2D Gaussian gives the lateral position of the fluorescence source, while the standard deviation is related to the defocus distance along the z-axis. For a defocused fluorescence image I_i, the initial fluorescence-source position $\hat{\boldsymbol{p}}_{\boldsymbol{i}}$ is obtained by solving:

$$\underset{\hat{p}_{ix},\hat{p}_{iy},\hat{p}_{iz},A}{\operatorname{argmin}} \sum_{x=1}^{N}\sum_{y=1}^{N} \left|\left| I_i(x,y) - A\exp\left\{-\frac{1}{2\sigma_z(\hat{p}_{iz})^2}\left[(x-\hat{p}_{ix})^2+(y-\hat{p}_{iy})^2\right]\right\}\right|\right|_2^2 . \tag{R7}$$

These positions are then treated as iterative parameters and are continuously refined during the training process.

The 3D RI is reconstructed from the same set of defocused fluorescence images using either conventional gradient-descent optimization [17] or FDT. FDT is the core contribution of this manuscript, because reconstructing RI from fluorescence images is more challenging than reconstructing fluorescence-source positions from the same images. The goal of this work is to develop a new algorithm that reconstructs 3D RI from fluorescence images more accurately and efficiently, and to demonstrate 3D RI reconstruction in bulky tissue samples with thicknesses of several hundred microns. In future work, we will present multimodal fluorescence–RI imaging using FDT, similar to our previous demonstration in BRIEF.

Comment 14. Validation with a single type of structure is not sufficiently convincing to assert that this work can be transposed in a general case for different types of samples and structures. The authors should consider more diversified ground truth data for their demonstration, or show conceptually in which framework good and bad results can be expected. Could the authors identify and describe expected reconstruction artifacts and how to remove them? So that non-expert users can identify them unambiguously and know how to optimize data acquisition and reconstruction?

Response. We thank the reviewer for this comment. We would like to clarify that we have validated FDT using both simulations and experiments on objects with different structural types and known ground-truth RI. Specifically, we first validated FDT by reconstructing the 3D RI of a simulated "UCDavis" pattern (Fig. 2). The

ground-truth RI model of the “UCDavis” pattern consists of 14 layers: each letter of “UCDavis” is centered in the odd-numbered layers, whereas the even-numbered layers are homogeneous. The second type of structure is spherical silica beads embedded in PDMS, which also have known ground-truth RI. We reconstructed the 3D RI of silica beads using both simulated and experimentally measured data (Fig. 2). In the revised manuscript, we also added new data showing 3D RI reconstruction of silica beads with different RI contrasts relative to the background ($\Delta RI = 0.01 - 0.05$, Fig. S3). Because the “UCDavis” pattern and the various silica beads differ in structure, shape, size, and RI, these results validate FDT across a broader range of structures.

We also experimentally demonstrated that FDT can be applied to different sample types and morphologies. We used FDT to reconstruct the 3D RI of living MDCK cells (Fig. 3), an epithelial cell line derived from dog kidney tubules that is widely used to study cell–cell junctions. We also used FDT to reconstruct the 3D RI of 3D-cultured bovine myotubes and revealed RI and morphological differences between muscle stem cells at different differentiation states (Fig. 4). These 3D-cultured bovine myotubes are relevant to cultured-meat technologies aimed at addressing global food demand. In the revised manuscript, we further added new experimental data (Fig. 5, Fig. R2) demonstrating 3D RI reconstruction of 3D-cultured HEK293T cells, a widely used human embryonic kidney cell line for protein expression, gene delivery, viral vector production, and studies of cell signaling and cellular function. Together, these results demonstrate that FDT is applicable to multiple cell lines and a broad range of biological structures.

As described above, we have also discussed several expected reconstruction artifacts and corresponding mitigation strategies. First, the reconstructed RI may exhibit noise-driven artifacts when the fluorescence images have low SNR (Fig. 2). This issue can be mitigated by improving the SNR, for example by increasing the exposure time, increasing the excitation power, or using fluorophores with high quantum yield and large absorption cross-sections. More detailed solutions for specific causes of low SNR are provided in our responses to Comments 6 and 8–12. Second, the reconstructed RI may exhibit artifacts related to incomplete spatial-frequency coverage of the OTF. Insufficient OTF coverage may lead to high-frequency artifacts when the number of fluorescence images is too small, or to distortions when the fluorescence-source distribution is spatially biased. These artifacts can be mitigated by increasing the number of fluorescence images and using more evenly distributed fluorescence sources (see our response to Comment 2 for details). In addition, because the OTF coverage is anisotropic, the lateral resolution of the reconstructed RI is higher than the axial resolution (Fig. 2, Fig. R5), which is a common limitation in imaging systems and may be mitigated by deconvolution. More details are provided in our responses to Comments 3, 4, and 12. Third, the reconstructed RI may become less accurate when the RI contrast between the object and the background is too large, i.e., for strongly scattering objects, because the reconstruction is based on the multi-slice model under the Born approximation for weak scattering (Table S6 and Fig. S3). This limitation could be mitigated by using more sophisticated light-propagation models, such as full-wave Maxwell solvers based on FDTD, at the expense of computational speed.

Comment 15. "We quantitatively validate FDT through simulations and experimental data of silica beads with known ground-truth RI, and then demonstrate its capability by reconstructing the 3D RI of living cells and bulky tissue volumes ($530\times530\times300\ \mu m^3$) with subcellular resolution". "Our results reveal individual cells with diverse morphological structures and RI during the processes of differentiation and aggregation (Fig. 4e). This demonstrates that FDT not only achieves high resolution but also effectively reconstructs structures across both low and high spatial frequencies" Imaging the "bulky volume" is not really a demonstration of the technique's validity, as approaches based on post-processing reconstruction of raw data will always produce results, but it must be carefully checked that no artefactual data is produced, which, as I understand it, is not the case for this specific acquisition. Asserting that there is some spatial variation in the signal and that the visible object resembles known cells does not seem to be a strong justification that the reconstruction faithfully reflects the object. Could the authors define more clearly what is meant by a measurement image? Is it a transmission image of the excitation beam? But then the two signals are measured at different wavelengths? Doesn't the fact that the recovered phase reflects the transmitted image necessarily mean that it's correct? Is it possible to have several possible phase distributions for a single transmitted image?

Response. We thank the reviewer for this important comment, which allows us to further clarify the scope and validation of our technique. All quantitative phase imaging (QPI) methods require computational reconstruction, because phase or RI cannot be measured directly by a camera, which records only light intensity. Our method is a QPI approach, more specifically an IDT-based method, with a rigorous physical foundation in the Fourier diffraction theorem first developed by E. Wolf in 1969 [14]. This framework has been widely used in many prior studies to reconstruct 3D RI from intensity-only measurements [2–6, 8–10, 13, 20–23], including our own previous work [17]. To validate reconstruction accuracy, we follow the standard approach used in quantitative RI imaging by testing the method on simulated and experimental datasets with known ground-truth RI. We assess reconstruction accuracy by comparing the reconstructed RI with the ground truth using quantitative metrics including MSE, SSIM, LPIPS, and PSNR. These results show that the reconstructed RI closely matches the ground-truth RI, as summarized in Table R2. Thus, the validity of the reconstruction framework is established using objects for which the RI is known.

For biological specimens such as MDCK cells, bovine myotubes, and HEK cells, ground-truth RI is not experimentally accessible, and the measured diffracted fluorescence images are the only direct experimental data available. In these cases, we evaluate reconstruction fidelity using the accumulated forward-model consistency over the full set of predicted and measured diffracted fluorescence images, quantified by MSE, SSIM, LPIPS, and PSNR. Because the predicted diffracted fluorescence images are generated from the reconstructed RI and fluorescence-source parameters through the multi-slice forward model, strong agreement between the predicted and measured images supports the fidelity of the reconstructed RI. We summarize these forward-model consistency metrics for the MDCK, bovine myotube, and HEK-cell experiments

in Table R3. For the bovine myotube, the forward-model consistency yields an MSE of 7.7843×10^{-3}, an SSIM of 0.7361, an LPIPS of 0.3024, and a PSNR of 22.7127.

We agree with the reviewer that imaging a bulky biological volume should not by itself be taken as the sole validation of the method. Rather, the purpose of the bovine-myotube experiment is to demonstrate the capability of FDT in a regime that remains challenging for ODT/IDT, namely 3D RI reconstruction of thick samples in epi/reflection geometry. This regime is particularly difficult because of limited optical sectioning, lower SNR, and greater computational complexity, as discussed in the revised Introduction. Accordingly, the manuscript is structured so that Fig. 2 provides validation against known ground truth, whereas Figs. 3–5 demonstrate applicability to increasingly complex biological samples, including bulky tissue-like specimens. The bovine-myotube result is therefore intended as a demonstration of capability in a challenging biological setting, supported by forward-model consistency, rather than as the primary ground-truth validation of the method.

Table R2 Quantitative metrics for samples with known ground-truth RI).

Sample		MSE	SSIM	LPIPS	PSNR
Simulated UCDavis	images	1.0238×10^{-3}	0.9814	0.0078	29.8980
	RI	1.8381×10^{-3}	0.9830	0.0513	27.3563
Simulated beads	images	1.2630×10^{-5}	0.9995	1.6880×10^{-4}	48.9860
	RI	5.0390×10^{-5}	0.9833	0.1306	42.9762
Experimental beads	images	4.3618×10^{-3}	0.9793	0.0220	27.9854
	RI	1.1503×10^{-3}	0.9860	0.0249	29.3918

Table R3 Forward-model consistency metrics for biological specimens (no ground-truth RI).

Sample	MSE	SSIM	LPIPS	PSNR
MDCK cells	6.2900×10^{-4}	0.9160	0.1417	32.0135
Bovine myotube	7.7843×10^{-3}	0.7361	0.3024	22.7127
HEK cells	3.5858×10^{-3}	0.5126	0.5970	24.6652

(Could the authors define more clearly what is meant by a measurement image? Is it a transmission image of the excitation beam? But then the two signals are measured at different wavelengths?)

A measurement image in FDT refers to a diffracted fluorescence image acquired by the camera at a defocused plane (Fig. 2b,c,i; Fig. 3b,c; Fig. 4b; Fig. 5b). It is not a transmission image of the excitation beam, but rather a fluorescence image detected in epi geometry, as shown in Fig. 1c. The excitation focus is used only to excite fluorescence from fluorophores, which then act as new light sources inside the sample. Because fluorescence is emitted isotropically into 4π steradians, it is not strictly appropriate to describe the fluorescence collected by the objective as "reflection-mode" or "transmission-mode" in the same sense as the excitation beam. Instead, we describe the fluorescence propagating toward the objective lens, opposite to the propagation

direction of the excitation light, as being detected in reflection/epi geometry. Thus, the fluorescence emitted by the fluorophores and collected by the objective in epi geometry constitutes the measurement image. The measurement image is acquired at the fluorescence wavelength, and the excitation beam itself is not recorded. By minimizing the difference between the predicted and measured diffracted fluorescence images, the recovered RI distribution is the one that best reproduces the measured data under the forward model based on the Fourier diffraction theorem.

(Doesn't the fact that the recovered phase reflects the transmitted image necessarily mean that it's correct? Is it possible to have several possible phase distributions for a single transmitted image?)

A single "transmitted" image (i.e., a diffracted fluorescence image) cannot uniquely determine the underlying 3D phase/RI distribution. This is precisely why FDT does not rely on a single measurement image, but instead uses hundreds of measurement images acquired under different illumination angles provided by different fluorescence sources. This can also be understood in terms of the OTF (Fig. R5). For a given pixel in the image plane, one measurement image corresponds to one illumination angle θ_{illu} at that pixel, which maps to one of the green arcs in Fig. R5 and provides only limited coverage in the Fourier domain. With multiple measurements, each image provides a different θ_{illu} at that pixel, corresponding to multiple distinct arcs in the Fourier domain. As the number of measurements increases, the OTF becomes more completely filled, thereby reducing aliasing caused by insufficient or biased sampling in the Fourier domain (see our response to Comment 2 for details). Therefore, with a sufficient number of measurement images, we can recover a unique 3D RI distribution that best reproduces all measured diffracted images, as quantified by minimizing the total intensity discrepancy between the predicted and experimentally measured images (Eq. 11-12; Tables R3).

Comment 16. Minor details: "The galvo mirrors and the electrically tunable lens can be functionally replaced by a spatial light modulator for beam steering, as both approaches achieve the same control of light in 3D (Fig. 1e, inset)." $->$ But not with the same temporal resolution as the refreshing rate of typical commercially available SLM is around 60Hz ie 17ms compare to the time response of standard galvos which goes up to 1μs. Justify more clearly the choice of moving optically the beam to change the axial position. I guess this is to have a fix collection configuration which is critical for the reconstruction?

Response. We agree with the reviewer that the temporal response of the galvo mirrors and electrically tunable lens (ETL) differs substantially from that of a spatial light modulator (SLM). We would like to clarify that our original statement was made in the context of the image acquisition time in the present experiments, which is 20–300 ms per image. This acquisition time is longer than the response times of both implementations, so either a galvo-plus-ETL system or an SLM could, in principle, provide the required 3D beam positioning in our current setup.

Regarding axial scanning range, both the ETL (EL-10-30-CI-NIR-LD-MV, Optotune) and the SLM (HSPDM1K-900-1200-PC8, Meadowlark Optics Inc.) provide a maximum experimentally measured axial scanning range of approximately 250μm in our imaging systems. Therefore, both approaches are capable of shifting the excitation focus along the axial direction over a similar range. For single-focus excitation, the galvo-plus-ETL system is a more practical choice and is therefore used in our current implementation.

Response to Reviewer 2

Comment 1. The manuscript presents a novel technique, Fluorescence-illuminated Diffraction Tomography (FDT), which reconstructs the 3D refractive index (RI) of thick, label-free biological tissues using diffracted fluorescence images acquired in reflection mode under two-photon excitation. The authors integrate an "explicit neural field" model to improve reconstruction speed and accuracy, and validate the system through both simulated data and real biological specimens. While the manuscript introduces a novel concept with innovative implications for deep-tissue multimodal imaging, the proposed method still requires the installation of a fluorescent bead layer on the opposite side of the sample. This raises concerns about whether the method can truly be considered a reflection-mode technique. The authors claim in the abstract, introduction, and conclusion that their approach operates in reflection mode and differs from transmission-mode imaging modalities that require access to the opposite side of the sample. However, because their method still necessitates the placement of fluorescent beads beneath the sample, it is unlikely to be applicable to in vivo imaging. For this reason, the authors' claims regarding reflection-mode operation are significantly compromised, and the manuscript, in its current form, does not appear to meet the publication standards of Nature Communications.

Response. We thank the reviewer for recognizing the novelty of our work and giving us the opportunity to address the concern of "reflection-mode" imaging. In the revised manuscript, we added new experiments using in vitro cells in which fluorescence originates **from fluorescence-labeled cells within the sample** instead of installation of fluorescent paint/beads on the opposite side of the sample. Specifically, we imaged 3D cultured HEK293T cells stained by exogenous fluorescence ActinGreen 488 (Fig. R8). We used two-photon excitation to excite fluorescence from cells located at a depth of approximately 130 μm depth within the sample, which become the fluorescence sources for reconstructing the RI of above cells. This new experiment demonstrates that FDT is able to reconstruct the RI of fluorescence-labeled bulky cells/tissue in reflection mode without accessing to the opposite side of the sample, allowing FDT to be translated to in vivo multimodal imaging in the future. We include the newly added section below for the reviewer's reference.

3D RI reconstruction of fluorescence-labeled HEK cells without transmission-side access

"To validate that FDT can reconstruct 3D RI when fluorescence originates *within* a thick specimen, we applied FDT to HEK293T cells cultured in a 3D Matrigel matrix

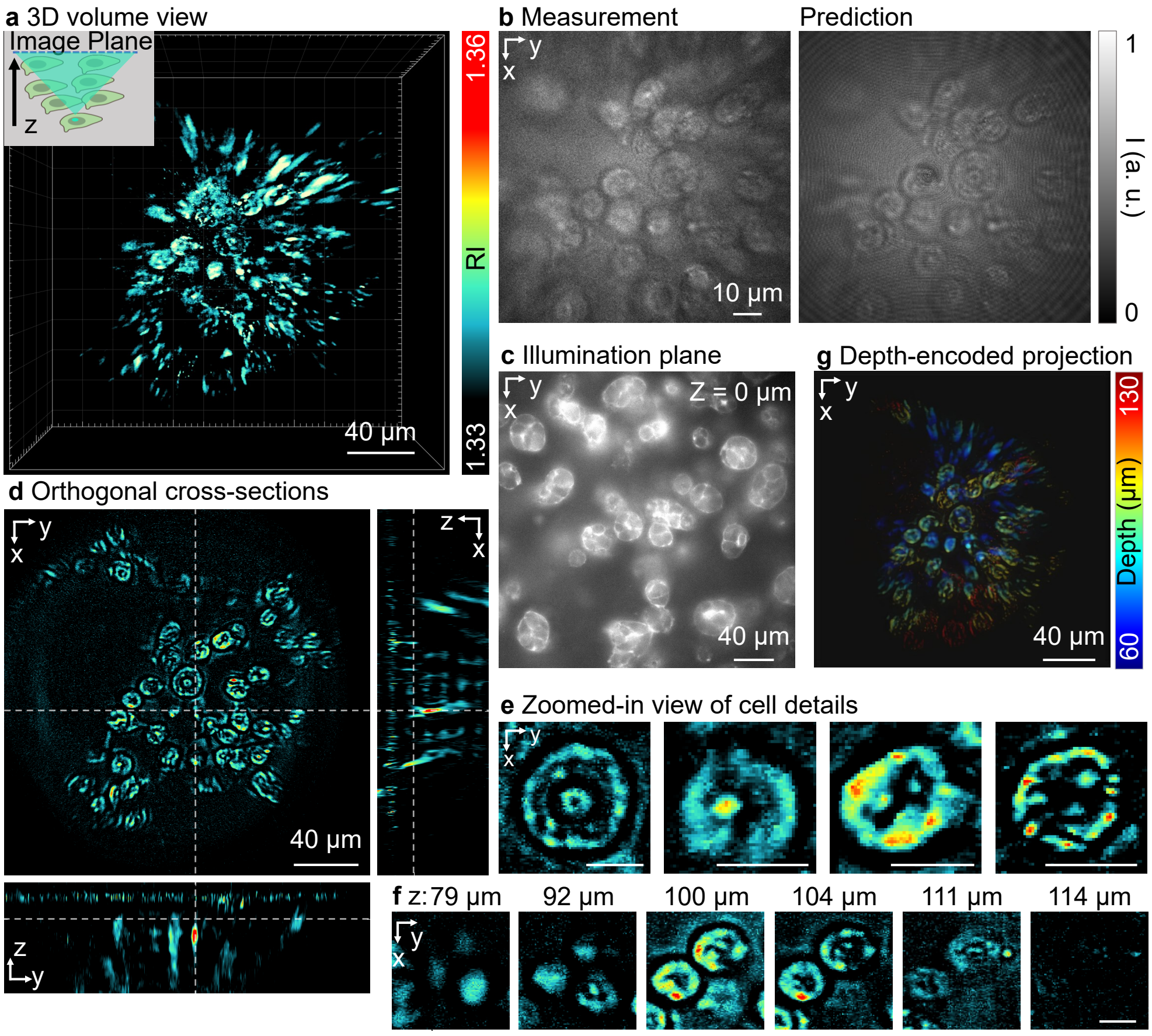

Fig. R8 3D RI reconstruction of ActinGreen 488-labeled HEK cells illuminated by fluorescence originating from the sample itself. (a) Reconstructed 3D RI of HEK cells over a 198 × 198 × 70 μm^3 volume. Inset: imaging geometry in which fluorescence-labeled cells serve as internal illuminators; their emitted fluorescence is diffracted by overlying cells and recorded as defocused images near the sample surface (see Extended Data Fig. 4d for details). (b) Representative diffracted fluorescence measurement (left) and corresponding prediction (right). (c) One-photon wide-field fluorescence image of HEK cells at $z = 0$ that act as fluorescence illuminators. (d) Orthogonal RI cross-sections ($x - y, x - z, y - z$); dashed lines indicate slice locations. (e) Zoomed-in RI images of representative cells showing clearly resolved membrane-scale features. Scale bar, 10 μm. (f) Zoomed-in RI z-stack demonstrating z-sectioning by resolving three cells at different depth. Scale bar, 10 μm. (g) Depth-encoded projection of the reconstructed RI volume.

and labeled with ActinGreen 488 (Fig. R8c; Extended Data Fig. 4d). Unlike the MDCK and bovine myotube experiments above, this measurement does not require access to the transmission side to apply fluorescent paint, making the approach applicable to specimens for which transmission-side access is not available. We used two-photon excitation to generate localized fluorescence within the cells at a depth of 130 μm below the sample surface (Fig. R8c). As fluorescence propagates upward toward the objective, it is diffracted by the RI variations of overlying cells. All

diffracted fluorescence images were acquired on a single camera plane near the sample surface (Fig. R8b, left; Extended Data Fig. 3d), and 282 images were selected based on SNR for 3D RI reconstruction.

Reconstructing the HEK-cell RI is more challenging than in the experiments above because the fluorescence images have lower SNR (Extended Data Fig. 3d; Fig. R8b), as ActinGreen-labeled actin is substantially dimmer than fluorescent paint. As shown in Fig. 2, low-SNR measurements can degrade RI reconstruction due to overfitting to noise. To mitigate this effect, we apply an *early-stopping* strategy: empirically, the model recovers physically consistent RI structure within the first several iterations, whereas prolonged optimization increasingly fits noise and degrades reconstruction quality. We therefore stop the optimization at iteration 60 (out of a maximum of 300), which also reduces reconstruction time.

By leveraging the early-stopping strategy, we reconstructed the 3D RI of HEK cells from low-SNR diffracted fluorescence images over a large volume ($198 \times 198 \times 70$ μm^3, $600 \times 600 \times 40$ voxels). Forward-model consistency supports reconstruction fidelity, which shows the predicted diffracted fluorescence images closely match the experimental measurements with a MSE of 3.5858×10^{-3} (Fig. R8b, Table S7). Orthogonal cross-sections (Fig. R8d) resolve individual HEK cells with clear, enclosed cell boundaries. Representative zoomed-in $x - y$ view further reveals membrane-scale features down to $\sim 1 - 2$ μm (Fig. R8e), demonstrating high-resolution RI recovery. Moreover, a zoomed-in RI stack distinguishes cells at different axial planes without out-of-plane crosstalk (Fig. R8f). At the volume scale, the depth-encoded projection (Fig. R8g) localizes cells across the full axial extent, highlighting the strong z-sectioning of FDT in thick samples. Together, these results demonstrate that FDT can reconstruct 3D RI in thick biological specimens using fluorescence originating from the sample itself, without transmission-side access."

The detail of the sample preparation was added to the Experimental Setup Section in the Supplementary document:

"**3D cultured HEK293T cells stained with ActinGreen 488.** HEK293T cells were resuspended in 50 μL Matrigel (Corning 354277) on ice at a density of 6.0×10^4 cells/μL. The resuspension was injected into a glass-bottomed mold 8 mm in diameter and 1 mm deep, followed by gelation at 37 °C for 1 hour. After gelation, the samples were then incubated at 37 °C overnight in Dulbecco's Modified Eagle Medium (DMEM; Thermo Fisher Scientific 11995065) supplemented with 10% fetal bovine serum (FBS; Thermo Fisher Scientific 16140071) and 1% Penicillin–Streptomycin (PS; Thermo Fisher Scientific 15140122). Samples cultured in 35-mm dishes were processed for immunocytochemistry as follows. Culture medium was removed and samples were quickly rinsed three times with phosphate-buffered saline (PBS), then fixed in 4% paraformaldehyde in PBS at room temperature for 20 min. After three additional PBS rinses, samples were either stored in PBS at 4 °C or immediately permeabilized with 0.1% Triton X-100 in PBS at room temperature for 45 min, followed by three PBS rinses. Samples were then incubated with ActinGreen 488 ReadyProbe (Thermo Fisher

Scientific, R37110) at room temperature for 45 min in the dark. Finally, samples were rinsed three times with PBS, covered with PBS, and stored in the dark until imaging."

In addition, we would like to clarify the terminology of "reflection-mode" and "transmission-mode" used in the manuscript. "Reflection-mode" and "transmission-mode" are typically used in standard ODT/IDT to describe the detection direction of illumination light from a laser diode or LED. However, FDT does not use laser or LED light directly for imaging. Instead, it uses these sources to excite fluorophores and then uses the emitted fluorescence as the illumination light. Because fluorescence is emitted isotropically into 4π steradians after excitation, it is not strictly appropriate to describe the fluorescence collected by the objective as "reflection-mode" or "transmission-mode" in the same sense as the excitation beam. Instead, we describe fluorescence propagating **in epi (reflection) geometry**, where excitation and detection are performed from the same side of the sample without access to the opposite side. We also explicitly state that some proof-of-concept demonstrations (Figs. 2–4) employed fluorescent dye painted beneath the specimen, while the new data (Fig. 5; Fig. R8) demonstrate the feasibility of imaging the RI of fluorescence-labeled cells in epi geometry.

Comment 2. Several critical issues in model validation, methodological assumptions, biological interpretation, and quantitative performance analysis also need to be addressed. The entire framework is based on a coherent forward model, while fluorescence emission—especially from two-photon excitation—is fundamentally incoherent or partially coherent. Although the authors attempt to resolve this with a partially coherent mask, this approach lacks theoretical justification or quantitative validation. The effect of using the partially coherent mask is presented in Fig. S2, but the effect of the coherence mismatch remains unclear. How does this mismatch affect reconstruction fidelity? Is the Born approximation still valid under this condition?

Response. We thank the reviewer for the comment. We would like to clarify that FDT uses a partially coherent mask as **a weighting factor** to solve the inverse problem more computationally robust without changing the physical model of light propagation. It is a computational strategy to mitigate the model mismatching rather than to convert an incoherent field into a coherent field.

We agree with the reviewer that fluorescence is an incoherent or partially coherent light source. According to prior work [24], partially coherent field can use the transport of intensity (TIE) for phase retrieval, which is the theoretical framework of our work. The original TIE [25] is derived for coherent light. The prior work [24] justified phase retrieval can be realized by directly applying the original TIE for partially coherent fields. Specifically, partially coherent light can be described using the Wigner Distribution Function (WDF) [24, 26]. Let $E(\mathbf{r})$ be a temporally stationary field with arbitrary spatial and temporal coherence. The mutual coherence function is defined as

$$\tilde{\Gamma}(\mathbf{r_1}, \mathbf{r_2}, \tau) = \langle E(\mathbf{r_1}, t) E^*(\mathbf{r_2}, t+\tau) \rangle, \tag{R8}$$

where the sharp bracket denotes the ensemble average. Correspondingly, the mutual power spectrum (i.e., cross-spectral density function) $\Gamma(\mathbf{r_1}, \mathbf{r_2}, \omega)$ is defined as the

temporal Fourier transform of the mutual coherence function $\tilde{\Gamma}(\mathbf{r_1}, \mathbf{r_2}, \tau)$:

$$\Gamma(\mathbf{r_1}, \mathbf{r_2}, \omega) = \int \tilde{\Gamma}(\mathbf{r_1}, \mathbf{r_2}, \tau) \exp(j2\pi\omega\tau) d\tau, \tag{R9}$$

where all integrations in the response letter extend from $-\infty$ to ∞ unless otherwise stated. Correspondingly, the WDF is defined as the positional power spectrum of light in a phase-space domain:

$$W(\mathbf{r}, \mathbf{u}) = \int \Gamma(\mathbf{r} - \frac{\mathbf{r'}}{2}, \mathbf{r} + \frac{\mathbf{r'}}{2}, \omega) \exp(-j2\pi\mathbf{u}\mathbf{r'}) d\mathbf{r'}, \tag{R10}$$

where u is the spatial frequency. With the WDF, the positional intensity of the incoherent/partially coherent field is [26]

$$\Gamma(\mathbf{r}, \mathbf{r}) = \frac{1}{2\pi} \int W(\mathbf{r}, \mathbf{u}) d\mathbf{u} \tag{R11}$$

and the generalized phase gradient $\tilde{\phi}(\mathbf{r})$ of the incoherent/partially coherent field can be defined as [24, 27]

$$\frac{\int \mathbf{u} W(\mathbf{r}, \mathbf{u}) d\mathbf{u}}{\int W(\mathbf{r}, \mathbf{u}) d\mathbf{u}} = \frac{1}{2\pi} \nabla_{\mathbf{r}} \tilde{\phi}(\mathbf{r}). \tag{R12}$$

The phase gradient (Eq. R12) is related to the positional intensity (Eq. R11) by

$$\frac{\partial \Gamma(\mathbf{r}, \mathbf{r})}{\partial z} = -\frac{1}{k} \nabla_{\mathbf{r}} \cdot [\Gamma(\mathbf{r}, \mathbf{r}) \nabla_{\mathbf{r}} \tilde{\phi}(\mathbf{r})]. \tag{R13}$$

Therefore, the defocused positional intensity is related to the phase gradient, which is the basic of TIE theory. Based on the theoretical framework, many previous IDT studies use diffracted images illuminated by partially coherent light (e.g., LEDs) to reconstruct 3D RI using the multi-slice model with Born approximation [5, 6, 9, 10, 21, 22], including our previous work under fluorescence illumination [17]. Compared to coherent light, partially coherent light solves the "missing cone" problem (Fig. 2), enabling better optical sectioning and reducing speckle noise [4, 8, 24, 28]. For these reasons, partially coherent illumination has been widely adopted in ODT/IDT. The Born/Rytov approximation assumes weak scattering in the medium, and its validity under partially coherent illumination has been established and applied in these prior studies [4–6, 9, 10, 17, 21, 22, 24].

In FDT, the fluorescence signal is detected after a narrow-band emission filter and can therefore be treated as quasi-monochromatic. In terms of spatial coherence, the fluorescence originates from a two-photon-excited spot. Under the standard definition of spatial coherence, a source whose effective extent is at or below the diffraction limit is spatially coherent across the collection pupil and can be modeled as emitting a well-defined wavefront. The measured PSF is $1.16 \times 1.16 \times 2.2$ μm^3 (Extended Data Fig.1), close to the theoretical diffraction limit of $0.55 \times 0.55 \times 1.8$ μm^3. Because the imaging system acts as a low-pass filter, the measured PSF provides an upper bound

on the size of the fluorescence sources. Accordingly, the actual size of the fluorescence sources can be smaller than the measured PSF. If the effective fluorescence source size is comparable to or slightly larger than the diffraction limit, the fluorescence source is a partially coherent point source. A standard treatment of partially coherent light is to model it as an incoherent mixture of coherent components. For example, in previous IDT work [5, 6, 9, 10, 21, 22], illumination from a single LED is modeled as a quasi-monochromatic coherent plane wave, whereas illumination from an LED array is partially coherent and is modeled as an incoherent sum of coherent plane waves from each LED. By the same reasoning, when each fluorescence source in FDT is slightly larger than the diffraction limit, its spatial incoherence can be modeled as an incoherent sum of coherent fields emitted by a cluster of fluorescence sources with nanoscale lateral shifts relative to the central one:

$$I(\mathbf{r}) \approx \sum_{m=0}^{M} w_m \, I_0\big(\mathbf{r} - \Delta\mathbf{r}_m\big) \qquad \sum_{m} w_m = 1,\ w_m \geq 0, \tag{R14}$$

where $\{\Delta x_m, w_m\}$ discretize the fluorophore distribution within the cluster. Importantly, the size of this fluorescence-source cluster is still close to the diffraction limit, especially relative to the reconstruction sampling grids of $0.33 - 0.52$ μm/pixel laterally and $0.5 - 12.5$ μm/pixel axially (Table R1), unlike an extended light source such as an LED array. Even if the source is represented as a sum of several coherent components, the central component remains dominant. Therefore,

$$I(\mathbf{r}) \approx w_0 \, I_0\big(\mathbf{r}\big), \qquad w_0 \gg w_m, m > 0. \tag{R15}$$

Given this dominance, instead of computing the incoherent sum, we generate a weighting mask to emphasize the high-intensity region associated with the central component while down-weighting low-intensity regions that are more susceptible to noise and model mismatch. Specifically, we generate a binary mask from the predicted coherent illumination pattern at the image plane in the absence of phase objects (i.e., for a homogeneous RI):

$$M(\mathbf{r}) = 1\Big(\hat{I}_{i,homo}(\mathbf{r}) > T\Big), \qquad M(\mathbf{r}) \in \{0, 1\}. \tag{R16}$$

where the threshold T is typically set to half of the normalized peak intensity of $\hat{I}_{i,\mathrm{homo}}(\mathbf{r})$. We then apply the mask to both the measured and predicted diffracted fluorescence images:

$$I_{i,mask}(\mathbf{r}) = M(\mathbf{r})\, I_i(\mathbf{r}), \qquad \hat{I}_{i,mask}(\mathbf{r}) = M(\mathbf{r})\, \hat{I}_i(\mathbf{r}), \tag{R17}$$

The weighted loss for each measurement-prediction pair is then calculated as

$$\begin{aligned} \mathcal{L}(\hat{n}; I_{i,mask}) = {} & \alpha \|\hat{I}_{i,mask} - I_{i,mask}\|_1 + \beta \|\hat{I}_{i,mask} - I_{i,mask}\|_2^2 \\ & + \gamma \ell_{\mathrm{ssim}}(\hat{n}; I_{i,mask}) + \tau_{xy} R_{xy}(\hat{n}) + \tau_z R_z(\hat{n}), \end{aligned} \tag{R18}$$

Therefore, the mask $M(\mathbf{r})$ suppresses these low-confidence regions and emphasizes pixels where the dominant coherent mode provides a more reliable description and higher SNR. Compared with adding-up multiple coherent modes (Eq. (R14)), the mask-based strategy introduces negligible computational overhead and avoids artifacts associated with discretizing. In summary, the mask is not intended as a physical equality relating coherent and incoherent intensities, but rather a weighting factor designed to reduce the influence of pixels where partial coherence introduces systematic residuals relative to a single-mode coherent forward model, consequently improving convergence speed and robustness. The Born approximation remains valid because it concerns the weak-scattering assumption of the sample, rather than requiring the illumination to be fully coherent. We have revised the manuscript accordingly.

Comment 3. Claims of subcellular resolution and high z-sectioning capability are repeatedly made, yet no formal resolution analysis (e.g., lateral/axial FWHM, modulation transfer function, Rayleigh limit) is provided. In figures in the manuscript, while images appear visually plausible, resolution is not quantified, and it remains unclear how much of the structure is truly resolved. The authors should investigate the lateral resolution and optical sectioning capabilities systematically and quantitatively.

Response. We thank the reviewer for this comment. We have added new data (Fig. 2e, f) to the revised manuscript to systematically and quantitatively evaluate the resolution and z-sectioning capability of FDT. Specifically, we calculate the theoretical resolution and evaluate the PSF and MTF of the reconstructed 3D RI for a simulated 1 μm-diameter microbead with known RI. We also assess the resolution and z-sectioning capability by reconstructing the 3D RI of two simulated microbeads that are laterally adjacent or axially overlapping with various separations. We also agree with the reviewer that the current data do not demonstrate that FDT can resolve subcellular organelles. Therefore, we have removed all claims of "subcellular resolution" from the revised manuscript. For the reviewer's reference, we include below the newly added paragraph and the revised Fig. 2 related to RI resolution.

"To quantitatively evaluate the resolution of RI reconstruction using FDT, we next reconstruct the 3D RI of simulated microbeads with known RI. The theoretical lower bounds of the resolution are determined by the objective numerical aperture, NA_{obj} ($NA_{obj} = 1$), and the illumination numerical aperture, NA_{illu}, according to prior work [5, 11–13]: $\Delta x = \lambda/[2(NA_{obj} + NA_{illu})]$ in the lateral dimension and $\Delta z = \lambda/(2n - \sqrt{n^2 - NA_{obj}^2} - \sqrt{n^2 - NA_{illu}^2})$ in the axial dimension. In FDT, NA_{illu} is determined by the fluorescence-source positions and the detection plane ($NA_{illu} = n\sin(\theta_{illu})$, Extended Data Fig. 2). In the experiments, the measured NA_{illu} ranges from 0.42 to 0.79, resulting in theoretical lower-bound RI resolutions of 0.25-0.32 μm laterally and 1.26-1.73 μm axially. Under the same parameters, the PSF of the reconstructed RI for a ~ 1 μm-diameter microbead is 0.20 μm laterally and 1.31 μm axially (Fig. R9e, left), indicating that FDT achieves high-resolution RI reconstruction. The corresponding modulation transfer function (MTF) (Fig. R9e, right) shows that FDT fills the "missing cone," thereby achieving excellent z-sectioning capability, as also demonstrated in the "UCDavis" result. We further reconstruct the 3D

RI of two adjacent 3 μm-diameter microbeads with different lateral and axial separations (Fig. R9f). As the lateral separation between the two microbeads increases from 0.1 μm to 0.3 μm, the reconstructed RI changes from connected to fully separable. Similarly, for two microbeads overlapping along the axial direction, as their separation increases from 0.5 μm to 1.5 μm, the reconstructed RI changes from inseparable to separable. These results further demonstrate that FDT enables high-resolution 3D RI reconstruction with good z-sectioning ability."

Comment 4. The explicit neural field method is claimed to be faster than implicit alternatives, yet computational cost is still uncertain (e.g., 50 minutes to 3 hours per reconstruction). What hardware is required? How does performance scale with volume size? The method's feasibility for broader adoption is unclear without reporting detailed runtime benchmarks, GPU memory requirements, and potential limits of reconstruction volume.

Response. We thank the reviewer for requesting more detailed benchmarking information. All experiments were conducted on a single NVIDIA RTX A6000 GPU (48 GB memory). No multi-GPU or distributed training was used. The batch size was fixed to 20 in all reported experiments.

For reconstructing RI in a volume of $N = N_x \times N_y \times N_z$ voxels, where $N_x \times N_y$ is the number of pixels per z-plane, and N_z is the number of axial planes. Since our method employs an explicit voxel-aligned neural representation, both memory consumption and per-iteration computational cost scale approximately linearly with N (for fixed batch size). For example, increasing the lateral number of pixels from 512×512 to 1024×1024 increases the total voxel count by approximately $4\times$, leading to a corresponding increase in runtime and GPU memory usage. Increasing the number of z-planes N_z results in near-linear growth in runtime and memory usage as well.

In practice, on a 48 GB RTX A6000, volumes up to $1024 \times 1024 \times 60$ are feasible without gradient checkpointing at batch size 20. On a 24 GB GPU (e.g., RTX A5000), practical configurations are typically around $512 \times 512 \times 40$ under the same batch size. Importantly, the method runs on a single workstation-class GPU and does not require specialized hardware or distributed infrastructure. We have added these benchmark details and scaling clarifications in the Supplementary document of the revised manuscript to improve reproducibility and transparency.

Table R4 Runtime and peak GPU memory usage per 100 iterations (batch size = 20). Experiments were conducted on a single NVIDIA RTX A6000 (48 GB).

Volume Size ($N_x \times N_y \times N_z$)	Time / 100 iters	Peak Memory
$512 \times 512 \times 20$	5 min	4 GB
$512 \times 512 \times 40$	10 min	7 GB
$512 \times 512 \times 60$	15 min	10 GB
$1024 \times 1024 \times 20$	8 min	17 GB
$1024 \times 1024 \times 40$	17 min	28 GB
$1024 \times 1024 \times 60$	33 min	39 GB

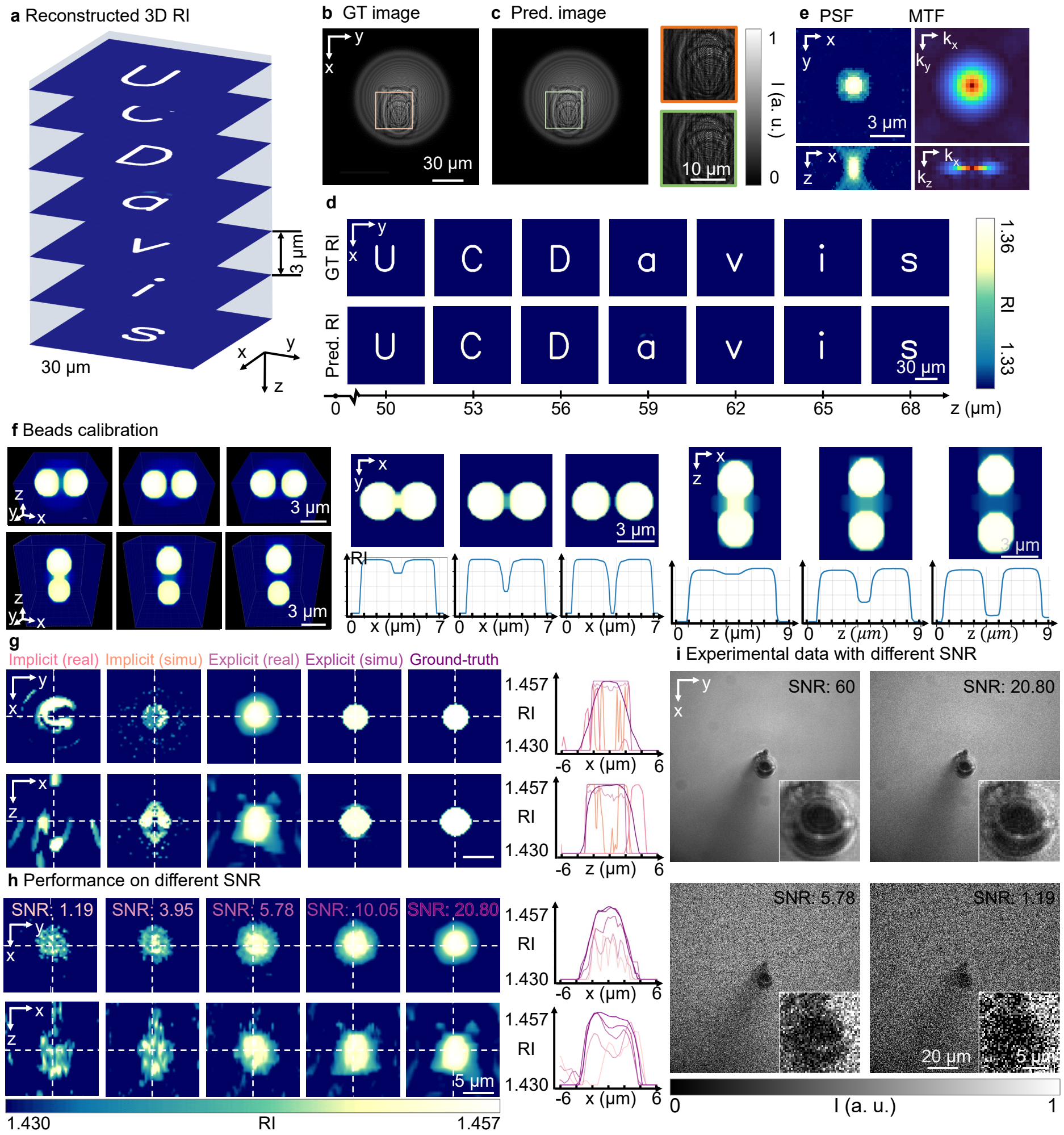

Fig. R9 Reconstruction of the 3D RI of a simulated "UCDavis" pattern. a, Reconstructed 3D RI distribution of the "UCDavis" pattern in 3D. **b,** A representative ground-truth (GT) image. **c,** Predicted fluorescence image together with zoomed-in views of the regions highlighted by the orange and green boxes. The MSE between the GT and predicted images and RI are 6.1125×10^{-7} and 1.5730×10^{-6}, respectively. **d,** Comparison of the ground-truth RI (top row) and the predicted RI (bottom row) on each z-plane, as indicated by the axis below. **e,** Simulated point spread function (PSF, left) and modulation transfer function (MTF, right) of the reconstructed 3D RI of a 1 μm-diameter microbead using FDT. **f,** Volume renderings, cross-sections, and line profiles of the reconstructed 3D RI of two microbeads that are laterally adjacent or axially overlapping at different separations. The lateral separations are 0.1 μm, 0.2 μm, and 0.3 μm, and the axial separations are 0.5 μm, 1 μm, and 1.5 μm. **g,** Calibration on silica beads using both simulated and experimental data. From left to right: implicit neural fields (experiment), implicit neural fields (simulation), explicit neural fields (experiment), explicit neural fields (simulation), ground truth (simulation). The dotted lines in the images mark the x- and z-directions for which the line profiles are plotted. **h,** Reconstruction performance at various signal-to-noise ratios (SNR), ranging from 1.19 up to 20.80. The dotted lines again indicate the x- and z-directions used to extract the line profiles. **i,** Forward images of the same beads at different SNR levels. The top-left image (SNR = 60) is used for the reconstruction in **f**, while the other three images are used for the reconstruction in **g**.

Response to Reviewer 3

Comment 1. The authors developed fluorescence-illuminated diffraction tomography to reconstruct the 3D refractive index (RI) of label-free objects from diffracted fluorescence images acquired in reflection mode under two-photon excitation. They employed the transport of intensity equation (TIE) and a self-supervised neural network to enhance reconstruction accuracy.

1. Firstly, several publications have demonstrated multimodal microscopy in reflection mode. there are reflection-mode techniques specifically designed for imaging 3D thick tissues in phase microscopy. For instance:

Ref. [22]: https://opg.optica.org/optica/fulltext.cfm?uri=optica-12-3-406&id=569159

Ref. [29]: https://www.pnas.org/doi/10.1073/pnas.2115323118

Ref. [30]: https://www.nature.com/articles/s41467-019-12634-3

Ref. [31]: https://opg.optica.org/boe/fulltext.cfm?uri=boe-10-7-3605&id=414828

Tomocube (although works in the transmission mode but can image thick sample)

Therefore, stating that most techniques operate in transmission mode without acknowledging the existing reflection-mode methods may be misleading.

Response. We thank the reviewer for pointing out these important prior works. In the revised manuscript, we have added the suggested references and expanded the Introduction to acknowledge this literature more explicitly. We would like to clarify that the goal of FDT is quantitative 3D RI reconstruction in reflection geometry from intensity-only diffracted fluorescence measurements, which is unique compared to the prior works. Below we summarize the key differences between FDT and the specific references listed by the reviewer (Table R5).

Reference [22]: Although Ref. [22] acquires images in reflection mode, it requires transmission-side access to place a mirror beneath the sample, which limits applicability to scenarios without access to the opposite side (e.g., many in vivo settings). The mirror is used to boost SNR because intrinsic backscattered light is typically much weaker than forward-scattered light in biological samples. In contrast, FDT does not require transmission-side access: it uses fluorescence originating within the specimen as internal illumination, as demonstrated in the newly added HEK experiment (Fig. 5; Fig. R2). In addition, our thick-sample demonstration reconstructs 3D RI in a $\sim 300\ \mu m$bovine myotube, whereas the thickest biological specimen shown in Ref. [22] is a $\sim 25\ \mu m$ fixed C. elegans worm. Finally, Ref. [22] primarily shows clear structure near the in-focus plane with increasing blur away from focus, while FDT resolves distinct structures across multiple z planes with reduced inter-plane crosstalk, consistent with strong z-sectioning. More discussion about Ref. [22] is provided in the response to the next question.

Reference [29]: Ref. [29] is a chip-based multimodal platform that requires culturing cells on a silicon nitride waveguide. The super-resolution fluorescence imaging uses laser light coupled in the waveguide from the sideway to image cells using total internal

reflection fluorescence (TIRF) and direct stochastic optical reconstruction microscopy (dSTORM). The phase imaging is based on Linnik interferometer that uses another partially coherent laser light source illuminating the cells on the waveguide from the top and detects reflected light from the waveguide substrate through the cells. The phase image is reconstructed from the interference pattern between the reflected light through the cells and the reference beam reflected by a mirror. Ref. [29] assumes a homogeneous RI in z (e.g., $n = 1.38$), and infers cell height from phase via ϕ as $h = \phi/(2k\Delta n)$ without considering 3D RI distribution. On contrary, our work does not require culturing cells on a waveguide to reflect light or access to the transmission-side of the sample (Fig. 5, Fig. R2). Our work does not require the interferometry setup but is able to use intensity images to reconstruct both the 3D structural map and the 3D RI of 3D cultured cells and tissues over a large volume. Since h and n are coupled in phase, separating these two and reconstructing both structural and RI map are more challenging than reconstructing phase only. We also developed a more rigorous physical model of light propagating that considers multiple Fresnel diffraction with Born approximation.

Reference [30]: Ref. [30] is a reflection-mode phase microscopy that implements a phase shifting add-on module to a commercial differential interference contrast (DIC) system. It does not measure RI but measures the derivative of phase on the in-focus plane. Volumetric data is acquired by acquire 2D images slice-by-slice on each z plane, which is different from diffraction tomography that reconstructs a 3D volume by 2D images taken from different perspective angles. On contrary, our work reconstruct the 3D RI rather than the derivative of phase, which is a more challenging task as discussed in the last paragraph. Therefore, even in reflection-mode, Ref. [30] does not image 3D RI, which measures different optical parameters from our work. In terms of imaging thick samples, the maximum reported thickness of biological samples in Ref. [30] is 120 μm in brain slice, and to our knowledge, the imaging depth/thickness of imaging mouse tender and larva zebrafish is not reported. On contrary, our work reconstruct the 3D volume from 2D images acquired without moving the z position step-by-step but on the same z plane, which is more challenging than taking a z stack, and reconstructed the 3D RI of 300 μm-thick bovine myotube. Notably, the volumetric images in Ref. [30] does not show distinguishable objects overlapping in z but rather a tilted plane, whereas our work is able to show distinguishable objects overlapping in z, demonstrating a better z sectioning ability. In addition, Ref. [30] cannot take fluorescence images simultaneously because the DIC configuration split both illumination and detected light into two beams using a Nomarski prism, whereas our work is able to conduct simultaneous fluorescence-RI imaging, as demonstrated in our previous work [17].

Reference [31]: Ref. [31] is a reflection-mode differential phase contrast (DPC) microscopy with oblique illumination. Similar to Ref. [29, 30], Ref. [31] also measures the phase of imaging objects rather than RI, and acquires volumetric data by taking a z stack slice-by-slice. Therefore, Ref. [31] is also a different imaging modality from our work because it does not measure 3D RI. The maximum thickness achieved in Ref.

[31] is 60 μm in ex vivo mouse brain, acquired by taken a z stack. On contrary, our work reconstruct the 3D RI of bulky samples as thick as 300 μm, as described above.

Tomocube: Tomocube's products are transmission-mode ODT that requires access to the transmission side of imaging objects, hindering their applications for in vivo imaging. More importantly, reflection-mode RI imaging is more challenging than transmission-mode because the number of intrinsically reflected photons is much fewer than the transmitted photons, and the distance photons propagate doubles in the reflection mode compared to the transmission mode [22, 29–31]. Since our work is in reflection geometry and aims to overcome the limitations of transmission mode, we focus on comparing our work with other reflection-mode techniques here. In terms of sample thickness, to our knowledge, the maximum thickness of 3D RI imaged using Tomocube's product is 200 μm in human pancreas tissue [32], which is below the 300 μm thickness we demonstrated using FDT.

Table R5 Compare FDT with state-of-the-art reflection-mode phase imaging techniques

**Ref.**	**Illumination**	**Reflective substrate**	**Imaging mechanism**	**Measured bio-specimen RI**	Thickness[1] (μm)
[22]	LED array	Mirror	IDT	Fixed cancer cells 1.328-1.332	15.6
[29]	Laser + rotation diffuser + multimode fiber	Waveguide chip	Linnik interferometer	N/A[2]	< 0.2
[30]	LED + diffuser	No	DIC + phase shifting	N/A[3]	120
[31]	LED + multimode fiber	No	DPC	N/A[4]	60
FDT (ours)	Fluorescence	No	IDT	Living MDCK cells 1.33-1.36	44
				Fixed myotube 1.33-1.35	300
				Fixed HEK cells 1.33-1.36	80

Comment 2. When the authors mention various types of reflection-mode phase imaging (line 59), I believe it would be feasible to integrate reflection-mode intensity diffraction tomography (recently introduced by Tian's group) with other fluorescence-based techniques such as SIM or TIRF. This possibility does not appear problematic and could have been discussed.

Response. We thank the reviewer for this insightful suggestion. We agree that, in principle, reflection-mode IDT could be combined with fluorescence modalities such

[0]Note:
1. The maximum thickness of 3D reconstructed RI with distinguishable structures across the volume in a biological sample.
2. The work [29] does not measure RI but measure phase. To calculate the height of cells, the RI is set to be a constant value of 1.38.
3. This work [30] does not measure RI but measure the derivative of phase.
4. This work [31] does not measure RI but measure phase.

as SIM or TIRF. However, we also note several practical considerations that make such a combined system nontrivial and, in most implementations, would likely require sequential (rather than truly simultaneous) acquisition.

First, the illumination of reflection-mode IDT and SIM/TIRF differ substantially. Reflection-mode IDT requires Kohler illumination, whereas SIM requires structured illumination and TIRF requires to generate an evanescent wave. These differences imply different optical hardware, alignment, illumination control, and image acquisition (exposure time, number of images, wavelength, etc). Thus they typically cannot be operated concurrently without crosstalk. In practice, systems that combine phase/RI and fluorescence under disparate illumination schemes generally acquire the two modalities sequentially, as in related multimodal implementations [29, 33–35].

Second, the accessible depth range and effective axial resolution can be mismatched, particularly for TIRF. TIRF typically can only image objects 0.1-0.2 μm (100-200 nm) thick limited by the evanescent-field penetration depth in the sample, whereas reflection-mode IDT targets volumetric phase/RI information over much larger axial extents (e.g., [22] achieves a resolution of 27 μm in axial when measuring 2 μm-diameter beads). This mismatch complicates interpretation of "multimodal" datasets because the two modalities may probe fundamentally different sample volumes with different resolution, as also discussed in prior multimodal platforms [29].

Third, the specific reflection-mode IDT implementation in Ref. [22] uses a reflective substrate (mirror) to enhance SNR. This requirement introduces additional constraints on compatibility with other modalities. For example, TIRF is most commonly implemented in an inverted geometry through a coverslip and relies on a high-NA interface; a mirror-based reflective substrate can obstruct or fundamentally alter the illumination geometry unless the system is redesigned. Moreover, both mirror-assisted reflection-mode IDT and TIRF are inherently best suited to thin samples and are not readily applicable to thick tissues or in vivo settings.

Finally, we would like to emphasize the distinction in scope and philosophy between such "shared-optics but sequential" multimodal systems and FDT. Most existing multimodal fluorescence–phase/RI platforms combine two distinct imaging modes that share part of the optical path but operate independently and sequentially to avoid crosstalk [29, 33–35]. In contrast, FDT uses fluorescence itself as the illumination mechanism for RI reconstruction, enabling RI to be decoded directly from the same fluorescence dataset acquired in epi geometry.

Comment 3. Lines 81-100: The authors attempt to address the challenge of imaging thick tissue by referring to their previously published work, BRIEF. They claim that accurate 3D RI of bulky tissue can be achieved via new algorithmic developments. However, the claimed accuracy is not substantiated in the presented results. The authors imaged MDCK cells and bovine myotubes, but no quantitative assessment is provided to demonstrate the precision of their RI reconstruction compared to established methods such as multi-slice beam propagation or nonlinear Born approximation (Park group, optica 2022).

Response. We thank the reviewer for the comment and would like to clarify these points. First, FDT reconstructs 3D RI using a multi-slice beam-propagation forward model under the Born approximation (Methods). This is the same forward model that has been widely adopted in IDT for weakly scattering samples [5, 6, 9, 10, 21, 22], including our prior BRIEF work [17]. In other words, our "new algorithmic developments" refer to the inverse solver (how we solve the inverse problem given the forward model), not a change to the underlying physical forward model. We have clarified this point in the revised manuscript.

The method cited by the reviewer (Park group, Optica 2022) is developed for ODT, which relies on interferometric measurements (e.g., off-axis Mach–Zehnder) and uses complex-field information obtained from holograms/interferograms. This measurement model and hardware are fundamentally different from IDT/FDT, which uses intensity-only measurements and a TIE framework. In addition, the validation regime and assumptions in that ODT work are tied to the transmission geometry and complex-field measurement, whereas our focus is reflection geometry and fluorescence-illuminated intensity measurements. For these reasons, a direct quantitative comparison to that ODT pipeline is not straightforward or particularly informative for assessing an IDT-based method.

We did not attempt to address the challenge of imaging thick tissue by referring to BRIEF. In fact, extending BRIEF to thick tissues is challenging in both hardware and computation, which motivates FDT. While FDT uses the same multi-slice Born forward model as BRIEF, it introduces a substantially more scalable inverse solver based on explicit neural fields, together with coarse-to-fine optimization, self-calibration of source positions, and partially coherent weighting. These advances make it computationally feasible to process hundreds of measurements with improved OTF coverage and reduced aliasing, which in turn improves reconstruction fidelity in thick specimens.

To evaluate reconstruction accuracy, we provide quantitative assessments using simulated and experimental datasets with known ground-truth RI, which is the standard approach for validating quantitative RI methods. We assess the accuracy of the reconstructed RI by comparing it with the ground-truth RI using quantitative metrics, including MSE, SSIM, LPIPS, and PSNR. The results show that the reconstructed RI closely matches the ground-truth RI, as summarized in Table R6. We further added new data to quantitatively evaluate the uncertainty of the reconstructed RI using simulated beads (Fig. R10, Table R8), which will be discussed in detail in our response to Comment 9. For biological specimens (MDCK, myotube, and HEK cells), ground-truth RI is not accessible, and the measured diffracted fluorescence images are the only experimental ground-truth data available. In this case, we quantify reconstruction fidelity using the accumulated forward-model consistency over the full set of predicted and measured diffracted fluorescence images, evaluated with MSE, SSIM, LPIPS, and PSNR. Because the predicted diffracted fluorescence images are generated from the reconstructed RI and fluorescence-source parameters using the multi-slice model, strong agreement between the predicted and measured images supports the accuracy of the RI reconstruction. We summarize these forward-model consistency metrics for the

MDCK, bovine myotube, and HEK-cell experiments in Table R7, further supporting the accuracy of the reconstructed 3D RI.

Table R6 Quantitative metrics for samples with known ground-truth RI.

Sample		MSE	SSIM	LPIPS	PSNR
Simulated UCDavis	images	1.0238×10^{-3}	0.9814	0.0078	29.8980
	RI	1.8381×10^{-3}	0.9830	0.0513	27.3563
Simulated beads	images	1.2630×10^{-5}	0.9995	1.6880×10^{-4}	48.9860
	RI	5.0390×10^{-5}	0.9833	0.1306	42.9762
Experimental beads	images	4.3618×10^{-3}	0.9793	0.0220	27.9854
	RI	1.1503×10^{-3}	0.9860	0.0249	29.3918

Table R7 Forward-model consistency metrics for biological specimens (no ground-truth RI).

Sample	MSE	SSIM	LPIPS	PSNR
MDCK cells	6.2900×10^{-4}	0.9160	0.1417	32.0135
Bovine myotube	7.7843×10^{-3}	0.7361	0.3024	22.7127
HEK cells	3.5858×10^{-3}	0.5126	0.5970	24.6652

Comment 4. Lines 101–134: The authors present FDT (explicit neural fields from two-photon fluorescence images) for 3D RI reconstruction. However, a clearer explanation of how FDT reconstructs the 3D RI using explicit neural fields is needed. Additionally, the authors should elaborate on the advantages of their method over conventional techniques. The comparison to references 37–42 appears to focus solely on computational speed and scalability, which seems inconsistent with the prior emphasis on accurate RI reconstruction. The manuscript does not convincingly explain how their approach offers superior RI accuracy or reconstruction quality.

Response. We thank the reviewer for the comment. We have revised the Introduction to explain the reconstruction algorithm of FDT, and the details of the method are provided in the following section "Model architecture of FDT" (Fig. 1, Methods, and Supplementary documents). First, as an IDT technique, FDT uses the same multi-slice model with Born approximation for forward rendering as used in other IDT techniques [5, 6, 17, 21, 24, 36], which keeps the rigorous physical model to link the 3D RI to predicted diffraction images. The difference between FDT and these conventional IDT techniques in terms of reconstruction algorithms is how to solve the inverse problem. In our previous work [17], we optimizes the voxelized RI volume directly using FISTA [37], which is a classic first-order proximal gradient method that has been widely used in convex optimization. However, quantitative 3D RI recovery in thick specimens demands improved axial resolution and z-sectioning, which in turn requires hundreds of measurements over a wide range of illumination positions/angles to more fully fill the optical transfer function (OTF) and suppress aliasing artifacts. This shift to substantially larger datasets makes computational efficiency a central limitation: the conventional gradient-descent optimization used in BRIEF requires hours for tens of images and becomes impractical for the hundreds of measurements needed for adequate

OTF coverage. Therefore, a new inverse solver that can efficiently process the large dataset is necessary.

In FDT, while using the same forward model, we change the method to solve the inverse problem by representing the unknown RI with explicit neural fields. Neural fields (NeRF) is a class of deep learning models that are designed for reconstruct 3D scene from 2D images, which is much more computationally efficient and scalable compared to FISTA. NeRF [38] (we refer to it as "implicit neural fields" in the manuscript) represents a 3D scene as a continuous volumetric field parameterized by a fully connected neural network (i.e., a multilayer perceptron (MLP)), which has been widely used for 3D scene reconstruction in computer vision and has been adapted for 3D RI reconstruction from intensity images [6]. However, one limitation of implicit neural fields is its slow convergence as it stores the unknown 3D scenes in the MLP weights, which is like a "black box" ("implicit") that needs long time to infer. Therefore, many modified NeRF algorithms have been developed to solve the limitation of the original NeRF, including the explicit neural fields [39]. Compared to implicit neural fields, the explicit neural fields store the representation explicitly in space (e.g., voxels/grids) rather than in a MLP. For FDT, explicit neural fields offer three practical advantages over implicit MLP-based representations:

1. Explicit neural fields provide a locally parameterized, multi-voxel representation of the RI volume, whereas implicit neural fields encode the entire volume in a global coordinate MLP. Because RI in biological specimens varies predominantly locally, a local parameterization improves numerical conditioning and yields more stable first-order or Gauss–Newton–style updates than a global MLP, whose parameters can couple distant spatial locations.

2. Explicit fields allow physically meaningful regularization to be applied directly to the reconstructed RI volume (e.g., voxel-wise or patch-wise priors). Applying regularization in the spatial domain improves robustness to low-SNR measurements. In contrast, enforcing comparable priors on implicit MLP weights is indirect and typically less effective.

3. Explicit neural fields enable faster and more scalable optimization: rendering and updates can be parallelized on the underlying grid, whereas implicit neural fields require repeated backpropagation through a deep network for each coordinate query. As a result, explicit representations can reduce reconstruction time from hours to minutes in our setting, which gives explicit neural fields good scalability for processing large datasets and resolving RI in bulky samples.

Compared to the results in our prior work [17], the reconstructed results using FDT show much better resolution and z sectioning ability, which distinguishes overlapping cells within a few microns (Fig. 3, Fig. 5). We benchmarked explicit versus implicit neural fields under identical conditions (same forward model, same measurements, same voxel count) on both silica beads with known RI and biological samples. The reconstructed RI using the implicit neural fields show severe cross talk from adjacent planes and high-frequency artifacts, whereas the explicit formulation yields more faithful 3D structure and improved forward-model consistency (Fig. 1f, Fig. 2, Fig. 3).

Quantitative metrics comparing implicit and explicit neural fields on reconstructing silica beads are summarized in Table S4.

Comment 5. Lines 137–154: The stated innovation of using a 2D camera instead of a PMT to record RI of label-free structures requires further clarification. Specifically, it is unclear how diffracted fluorescence images—where phase information is inherently lost—can be used to recover phase information required for RI reconstruction. While some discussion appears in lines 285–292, this comes too late to clearly understand the fundamental illumination and collection strategy. Moreover, the manuscript does not specify whether focused or wide-field illumination was used, which affects the reconstruction method. It is also unclear whether each two-photon signal was treated as a point source during RI reconstruction. These are important technical details that should be clarified.

Response. We thank the reviewer for the comments. FDT is an IDT technique. Although phase is not measured directly, IDT reconstructs phase/RI from intensity-only measurements based on the TIE and related weak-scattering models [24, 25, 40, 41]. In brief, TIE relates the transverse phase to the axial derivative of intensity, so phase can be recovered from defocused intensity measurements without interferometry. This forms the theoretical foundation of IDT, which reconstructs 3D RI from intensity-only images acquired at defocus and/or multiple perspectives [6, 10, 17, 21, 36, 42, 43]. Specifically, the TIE is derived as [25, 40] follows.

A light field $u_z(\mathbf{r})$ satisfies paraxial wave equation:

$$(i\frac{\partial}{\partial z} + \frac{\nabla^2}{2k} + k)u_z(\mathbf{r}) = 0, \tag{R19}$$

where $\nabla^2 = [(\partial^2/\partial x^2)+(\partial^2/\partial y^2)]$ and $k = 2\pi/\lambda$. The light field $u_z(\mathbf{r})$ can be expressed in terms of the intensity I and the phase ϕ:

$$u_z(\mathbf{r}) = \sqrt{I}\exp[i\phi_z(\mathbf{r})]. \tag{R20}$$

From Eq. R19 and Eq. R20, one can derive

$$k\frac{\partial}{\partial z}I = -\nabla_{\mathbf{r}} \cdot I\nabla_{\mathbf{r}}\phi. \tag{R21}$$

Eq. R21 is the equation for the transport of intensity. It reveals the intensity change along the z direction is related to the derivative of phase in the transverse direction. TIE theory works for both coherent and partially coherent illumination [24, 40].

In FDT, the raw measurements are defocused, diffracted fluorescence intensity images, and we model fluorescence propagation through weakly scattering media using the established multi-slice forward model under the Born approximation [14, 17, 21, 36, 42]. We have revised the text to emphasize that the key requirement is capturing fluorescence images on a defocused plane, where heterogeneous RI converts phase modulation into measurable intensity variations; these intensity variations are then used for RI reconstruction within the TIE/IDT framework.

(Moreover, the manuscript does not specify whether focused or wide-field illumination was used, which affects the reconstruction method. It is also unclear whether each two-photon signal was treated as a point source during RI reconstruction.)

FDT involves two conceptually distinct steps. First, fluorophores are excited ("turned on") by a diffraction-limited two-photon excitation focus, as in standard point-scanning two-photon microscopy. Second, the excited fluorophores act as internal secondary light sources and emit fluorescence that propagates toward the objective as spherical waves. This emitted fluorescence then illuminates overlying structures in a wide-field manner and is diffracted by heterogeneous RI, producing the defocused intensity patterns recorded on the camera. Thus, while excitation is generated by a focused scanning beam, the illumination relevant to RI reconstruction is the emitted fluorescence field originating from localized sources within the sample (Fig. 1a; Extended Data Figs. 2–4). In reconstruction, we model each two-photon–excited fluorescence source as an effective point-like emitter (a spherical-wave source), with its position estimated via self-calibration (see "Model architecture of FDT"). We do not model the two-photon excitation field in the RI reconstruction. Instead, the forward model begins with the emitted fluorescence field and propagates it through the sample using the multi-slice Born framework (Fig. 1; Methods). Conceptually, two-photon excitation plays a role analogous to "powering" a localized internal fluorescence emitter, while the reconstruction uses the emitted fluorescence as the illumination field. Details are provided in the revised section "Optical setup and fluorescence-illuminator characterization for FDT."

Comment 6. The results section does not clearly describe how fluorescence imaging contributed to RI reconstruction. Furthermore, the claim of reconstructing thick samples up to 300 microns deep raises questions, as multiple scattering would likely degrade image quality. The manuscript lacks explanation of how such scattering was mitigated.

Response. We thank the reviewer for this comment. FDT reconstructs the RI distribution from diffracted fluorescence images recorded at a defocused plane. In this framework, the diffracted fluorescence images play a role analogous to the raw intensity measurements in conventional IDT/ODT and serve as the key input for RI reconstruction. Specifically, fluorescence excited by two-photon illumination act as internal secondary light sources. As the emitted fluorescence propagates through the sample toward the objective, it is diffracted by RI heterogeneities, producing the measured fluorescence diffraction patterns at the defocused plane. The heterogeneous RI distribution can then be reconstructed from these diffracted fluorescence images using TIE theory. The goal of the reconstruction is to recover the RI distribution whose forward-predicted diffracted fluorescence images best match the measured ones, thereby ensuring forward-model consistency. We have revised the sections "Results: Optical setup and fluorescence-illuminator characterization for FDT" and "Results: Model architecture of FDT" to describe both the acquisition of the diffracted fluorescence images and the reconstruction of the 3D RI from these measurements.

In FDT, we leverage the different scattering behaviors of the near-infrared excitation light and the visible fluorescence emission. The near-infrared excitation beam is relatively robust to tissue scattering because of its longer wavelength and the nonlinearity of two-photon excitation. For the 300 μm-thick bovine myotube studied here, scattering of the excitation light is sufficiently weak [44–48] that the two-photon-excited fluorescence can be approximated as arising from an internal partially coherent point source within the sample. In contrast, the visible fluorescence emission experiences substantially stronger scattering as it propagates through the tissue. Rather than mitigating this scattering, FDT explicitly leverages it: the heterogeneous RI distribution is encoded into the diffracted fluorescence images through this propagation process. To model this, FDT uses a multi-slice light-propagation framework that accounts for multiple scattering. Although the cumulative scattering through the full tissue thickness is strong and produces complex fluorescence diffraction patterns, each individual thin slice contributes comparatively weak scattering that can be approximated under the Born approximation. This is the basis of the multi-slice model for multiple scattering [17, 21, 36]. As shown in Fig. 4b, the diffracted fluorescence images from the 300 μm-thick bovine myotube exhibit complex features that are not directly interpretable without reconstruction, precisely because multiple scattering strongly scrambles the emitted light. By incorporating the multi-slice model into the forward model, FDT can account for this multiple-scattering process and reconstruct the 3D RI distribution from these measurements. The reconstructed cellular-level RI structures shown in Fig. 4a and Fig. 4c–e therefore demonstrate the feasibility of FDT for 3D RI reconstruction in a 300 μm-thick bovine myotube. We added a discussion of scattering in the section “Results: Optical setup and fluorescence-illuminator characterization for FDT” and in the Discussion section.

Comment 7. Lines 156–208: The architecture of the FDT model is presented, but it remains unclear how amplitude information is learned from intensity-only fluorescence images. The fundamental distinction between coherent (label-free) and incoherent (fluorescence) imaging is not adequately addressed. The self-calibration method, though useful, has been previously explored by other groups and should not be considered a novel contribution. Regarding the differential multi-slice model: is there a conceptual or practical comparison with the established multi-slice beam propagation method, which is also designed for thick tissue imaging? A comparative discussion would be beneficial. The manuscript also refers to a partially coherent mask designed to minimize discrepancies. However, it is unclear what specific discrepancies are being addressed. Is the discrepancy related to the loss of phase in fluorescence imaging compared to phase-preserving coherent imaging?

Response. We thank the reviewer for this thoughtful comment. FDT reconstructs phase information from intensity-only fluorescence images by combining TIE theory with a multi-slice forward model, as discussed in our response to Comment 5 above. In principle, this framework can reconstruct both amplitude and phase. However, for the biological samples studied here, amplitude modulation (absorption) is orders of magnitude smaller than phase modulation (scattering). We therefore focus on reconstructing the RI distribution, which primarily modulates the optical phase.

The governing equations of the forward model, which are compatible with both amplitude and phase reconstruction, are provided in Eqs. 1–7 in the Methods and have been demonstrated in prior work [5] using the same modeling framework.

We also clarify that FDT does not rely on coherent imaging. Instead, it uses incoherent or partially coherent fluorescence-based imaging to reconstruct the RI of label-free structures, as discussed in our response to Comment 6. More broadly, partially coherent illumination has been widely used in phase and RI imaging of label-free samples (Table R5, [4, 17, 21, 22, 24, 28–30, 36, 42]) because it offers higher resolution, improved axial sectioning, and reduced speckle compared with fully coherent illumination. The distinctive feature of FDT is that it integrates fluorescence-labeled and label-free imaging at a deeper level by reconstructing the 3D RI of label-free structures directly from fluorescence images. This differs from prior multimodal approaches in transmission geometry [29, 33–35], where fluorescence and label-free images are typically acquired sequentially.

We further clarify the role of self-calibration. We do not intend to claim self-calibration itself as a fundamentally new concept. Rather, the novelty in FDT lies in how fluorescence-source position calibration is incorporated into the inverse problem, particularly relative to prior IDT approaches [21, 36] and our previous work [17]. In these studies, illumination angles were typically calibrated manually in a separate preprocessing step. In FDT, by introducing the explicit neural-field formulation, we are able to treat source-position calibration parameters as trainable variables that are jointly optimized with the RI reconstruction. This integrated self-calibration improves reconstruction accuracy, as shown in Figure S1 and Table S3.

The differential multi-slice model used in FDT for forward rendering is the same as the established multi-slice beam propagation method [5, 14, 17, 21, 24, 36], as discussed in our responses to Comments 3–6. We have clarified this point in the revised manuscript.

Finally, we clarify the meaning of the "discrepancy" addressed by the partially coherent mask. This discrepancy does not arise from the loss of phase in fluorescence imaging relative to coherent imaging, since phase and RI information can still be recovered from intensity-only measurements through TIE-based inversion. Instead, the discrepancy refers to model mismatch between the experimentally measured fluorescence images and the idealized source model used in the forward calculation. In the model, fluorescence emission is approximated as spherical waves originating from a diffraction-limited point source at a central wavelength, effectively treating the fluorescence source as a spatially coherent quasi-monochromatic illuminator. In practice, however, the fluorescence source may be slightly larger than the diffraction limit, introducing a small degree of spatial incoherence. To reduce this mismatch, we introduce the partially coherent mask as a weighting factor. A detailed explanation has been added to the section "Results: Model architecture of FDT," as shown below:

"To improve convergence speed and robustness, we apply an intensity-based weighting mask ("partially coherent mask") to both predicted and measured images before computing the loss (Fig. 1d; Supplementary 5.5). Because the effective fluorescence source size is comparable to or slightly larger than the diffraction limit (upper bound

$1.16 \times 1.16 \times 2.2\ \mu m^3$ from the measured two-photon PSF; Extended Data Fig. 1), the fluorescence source is a partially coherent point source. The detected intensity can be approximated as an incoherent sum of a dominant central component and weaker, shifted components, $I(\mathbf{r}) \approx \sum_{m=0}^{M} w_m I_0(\mathbf{r} - \Delta\mathbf{r}_m), \sum_m w_m = 1,\ w_m \geq 0$ with $w_0 \gg w_m$ for $m > 0$, such that $I(\mathbf{r}) \approx w_0 I_0(\mathbf{r})$. Given this dominance, instead of computing the incoherent sum, we generate a weighting mask to emphasize the high-intensity region associated with the central component while down-weighting low-intensity regions that are more susceptible to noise and model mismatch. Specifically, we generate a binary mask from the predicted coherent illumination pattern at the image plane in the absence of phase objects, apply it to the predicted image $\hat{I}$ and the measured image I to obtain the weighted images $\hat{I}_{mask}$ and I_{mask}. We then compute ℓ_1, ℓ_2, and SSIM losses between $\hat{I}_{mask}$ and I_{mask} with a total variance (TV) regularizer to the predicted RI along all three dimensions (Fig. 1d; details in Supplementary 5.5). ”

Comment 8. Lines 285–383: The explanation becomes somewhat clearer here. The label-free image is obtained with cells cultured on the bottom of a glass dish, and the fluorescence dye is applied externally. Does this imply that each dye particle acts as a point source in the reconstruction? This could be explicitly stated.

Response. We thank the reviewer for this comment. We agree with the reviewer's understanding that the label-free image is obtained with cells cultured on the bottom of a glass dish, while the fluorescence dye is applied externally. We have added new diagrams to illustrate the sample configuration (Fig. R1). Importantly, it is the cluster of dye particles within the two-photon excitation focus, rather than each individual dye particle, that acts as a point source in the reconstruction. This is because each dye molecule is only a few nanometers in size, whereas the two-photon excitation focus is a few hundred nanometers wide and is approximately diffraction-limited. As a result, all dye particles within the two-photon focus are excited together. We therefore do not model individual dye particles on the nanometer scale as separate point sources; instead, we treat the diffraction-limited excitation volume as a single point source. According to the definition of spatial coherence, an emitter with a size smaller than or comparable to the diffraction limit can be treated as spatially coherent. In other words, the fluorescence originating from a diffraction-limited focus, although generated by many dye molecules, can be approximated as a spatially coherent point source.

Comment 9. Lines 294–341: The fluorescence source is located 105 microns beneath the cells. It would be helpful to know whether using LEDs instead of fluorescence sources would alter the reconstruction results. The claim of higher sensitivity and larger field of view lacks empirical support. For example, can the authors demonstrate that their method can detect RI changes with a sensitivity of 0.005? A quantitative evaluation would significantly strengthen the manuscript.

Response. We thank the reviewer for this comment. We respectfully disagree that replacing fluorophore-based illumination with LEDs would serve as a validation of the reconstructed RI, because RI reconstruction under either illumination scheme still depends on forward-model approximations, such as the Born approximation,

Rytov approximation, paraxial approximation, or quasi-monochromatic and plane-wave approximations for LED illumination. A more standard and direct way to validate RI reconstruction accuracy is to reconstruct objects with known RI, such as microbeads used as fiducial markers.

To address this point, we have added new results that quantitatively evaluate the reconstruction accuracy of FDT using simulated microbeads with known RI (Fig. R10, Table R8). Specifically, we simulated five microbeads with RI contrasts relative to the background ranging from $\Delta RI = 0.01 - 0.05$, in increments of 0.01. We evaluated reconstruction accuracy using the standard deviation (STD) of the reconstructed voxel-wise ΔRI. The reconstruction results show the STD of reconstructed RI is in the order of 10^{-4}. When the ground-truth ΔRI increases from 0.01 to 0.02, the reconstructed ΔRI increases from $0.0080 \pm 5.84 \times 10^{-4}$ to $0.0202 \pm 5.29 \times 10^{-4}$, indicating sufficient sensitivity to distinguish an RI difference of 0.005 for weakly scattering objects. A similar conclusion holds when the ground-truth ΔRI increases from 0.02 to 0.03.

For stronger-scattering beads with $\Delta RI > 0.03$, the root-mean-square error (RMSE) between the ground-truth and reconstructed ΔRI becomes substantially larger ($4.80 \times 10^{-3} - 1.30 \times 10^{-2}$), indicating breakdown of the weak-scattering assumption in this regime. Accordingly, when the ground-truth ΔRI increases from 0.04 to 0.05, the reconstructed ΔRI changes only from $0.0352 \pm 2.64 \times 10^{-4}$ to $0.0370 \pm 2.82 \times 10^{-4}$, which is insufficient to resolve a RI difference of 0.005.

We would like to clarify that the manuscript does not claim that FDT provides a higher sensitivity or a larger field of view than existing ODT/IDT techniques. The goal of FDT is to provide a proof-of-concept demonstration that the 3D RI of bulky biological samples can be reconstructed from diffracted fluorescence images in epi (reflection) geometry, rather than to compete with existing ODT/IDT methods in sensitivity or field of view. In particular, we do not make any claim regarding a larger field of view anywhere in the manuscript. Our comparison is limited to FDT implemented with explicit versus implicit neural fields, where the results show that the explicit neural-field formulation enables aliasing-suppressed 3D RI reconstruction with stronger z-sectioning, higher resolution, and higher sensitivity relative to the implicit neural-field approach. We have revised the text to make this distinction clearer.

Table R8 Quantitative evaluation of reconstructed RI accuracy with simulated beads

Bead	GT ΔRI	Num. of Voxels	ΔRI RMSE	Pred. ΔRI Mean ± STD
Bead 1	0.01	418	2.11×10^{-3}	$0.0080 \pm 5.84 \times 10^{-4}$
Bead 2	0.02	420	5.80×10^{-4}	$0.0202 \pm 5.29 \times 10^{-4}$
Bead 3	0.03	434	4.95×10^{-4}	$0.0298 \pm 4.66 \times 10^{-4}$
Bead 4	0.04	434	4.80×10^{-3}	$0.0352 \pm 2.64 \times 10^{-4}$
Bead 5	0.05	420	1.30×10^{-2}	$0.0370 \pm 2.82 \times 10^{-4}$

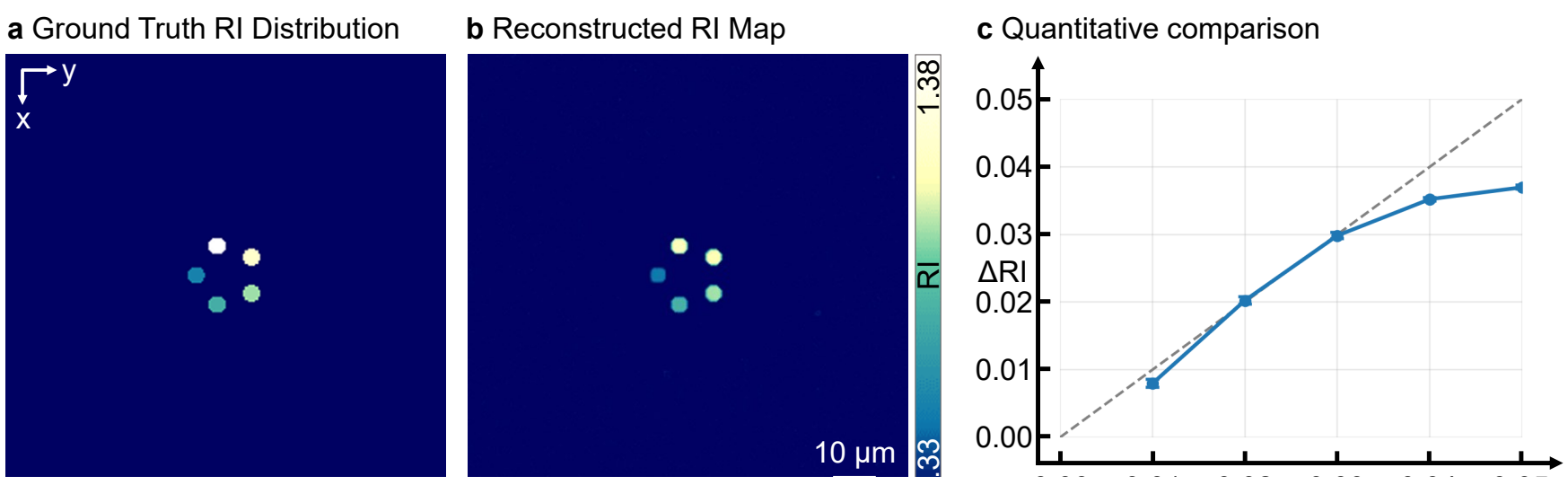

Fig. R10 Refractive index reconstruction of beads with varying RI. a. Ground-truth refractive index (RI) distribution of beads with RI contrasts ranging from 0.01 to 0.05. **b**. Reconstructed RI distribution of the simulated beads. **c**. Cross-sectional RI profiles of the beads comparing the reconstructed results with the ground truth.

Comment 10. Figure 4 presents the central result, where the authors attempt to reconstruct the 3D RI of thick bovine myotubes. While imaging thick samples is indeed a pressing issue in QPM, the reconstruction quality shown here is insufficient to validate the claimed accuracy and sensitivity of the method. Therefore, in my opinion, the paper is missing several important details and lack to explain the precise novelty and proof the novelty through their results.

Response. We thank the reviewer for this comment. To address this concern, we have added new results that quantitatively evaluate RI reconstruction accuracy against ground-truth RI, as discussed in our response to the previous comment. When ground-truth RI is unavailable, as in the case of the bovine myotube, we assess reconstruction fidelity using the accumulated forward-model consistency between the predicted and measured diffracted fluorescence images. The optimization is considered converged when the reconstructed RI distribution and fluorescence-source parameters jointly reproduce the measured diffracted fluorescence images through the multi-slice forward model. For the bovine myotube shown in Fig. 4, the accumulated forward-model consistency between the experimentally measured images and the images predicted from the reconstructed 3D RI is high, with MSE $= 2.8607 \times 10^{-3}$, SSIM $= 0.7361$, LPIPS $= 0.3024$, and PSNR $= 22.7127$ (Table R7). These results indicate strong agreement between the measured data and the forward predictions from the reconstructed 3D RI, supporting the accuracy of the reconstructed RI.

We have also revised this section to describe the myotube results more carefully. The goal of this experiment is to demonstrate that FDT can reconstruct cellular-resolution RI distributions over a $\sim 300\ \mu m$-thick volume and across various biologically relevant morphologies. The orthogonal views reveal the tubular morphology of the sample, and the circular cross-section in the $x - z$ view supports the integrity of the reconstructed tube structure. The clear separation between two distinct z-planes in the tube further illustrates the z-sectioning capability of the method. In addition, we have added a control experiment on myotubes cultured under the same conditions and subsequently characterized by immunofluorescence staining, which corroborates that the observed morphological transitions track molecular markers of myogenic differentiation (Fig. S6). Together, these results support the conclusion that FDT can resolve cellular

structure in thick specimens while quantitatively capturing RI variations associated with cellular state.

In summary, we have revised the manuscript to better explain the scope, novelty, and supporting evidence of FDT, and we hope that the revised manuscript and response letter adequately address the reviewer's concerns.

Response to Reviewer 4

Comment 1. This manuscript presents an approach for using fluorescence illumination to conduct diffraction tomography for reconstructing the refractive index distribution of samples. The approach works by having a fluorescent layer beneath the sample of interest and then exciting it point wise by two photon illumination. The diffracted light is then analyzed to reconstruct the structure of the sample supported by transport of intensity equation and the solved with a self supervised neural network.

The approach builds upon previous work that demonstrated the general principle (Ref 12), while the two photon excitation was introduced in a conference presentation. The advances here are to switch from implicit to explicit fields where a point wise reconstruction is used rather than a continuous function. Additional points of novelty are a self-calibration routine, use of defocused images on a 2D sensor rather than a point sensor and introduction of a partially coherent mask. In the introductory work, there are several repeated citations (3,18), (1,19), (11, 21) (2, 22) are re-used. The authors should also compare with the back oblique microscopy approach developed by Prof. Robles at Georgia Tech which operates on a similar principle to obtain quantitative phase information.

Response. We thank the reviewer for the comment. We have removed the reused citations.

We agree that our method (FDT) and quantitative oblique back-illumination microscopy (qOBM) developed by Prof. Robles' group look similar as they both operate in epi geometry. However, they differ in several key aspects in their imaging and reconstruction mechanisms. First, the illumination strategy is fundamentally different. qOBM uses obliquely incident LED illumination, and the diffusely scattered light carrying RI information reaches the objective through a banana-shaped path [31, 49]. In contrast, FDT uses fluorescence emitters within the specimen as internal illumination sources. Second, the raw measurements are different. For 3D reconstruction, qOBM acquires a through-focus stack slice-by-slice at each z plane under each oblique illumination, while FDT acquires diffracted fluorescence images at a single defocused plane, which is the same image plane for all raw measurements without translating the sample or objective. Third, most importantly, the reconstruction models to calculate the 3D RI from raw measurements are different. qOBM estimates a 3D optical transfer function (OTF) and then deconvolutes the raw image stack with the 3D OTF to calculate a 3D scattering potential, which is approximately linearly related to RI over a finite thickness (i.e., a thin slice). However, accurate experimental measurement of the 3D OTF is challenging because it would require an embedded point-like

phase object as a fiducial, which may not exist in many samples. therefore, qOBM typically relies on Monte Carlo simulations informed by literature scattering properties [49]. While recent work measures the oblique intensity distribution in the Fourier plane [50], the full 3D OTF remains difficult to obtain experimentally because the true tissue scattering properties are generally unknown. In addition, deconvolution-based reconstruction assumes a spatially invariant OTF, which may be violated in practical, heterogeneous specimens. In contrast, FDT reconstructs RI without estimating an OTF or assuming spatial invariance. FDT models wave propagation through the sample using a multi-slice forward model under the Born approximation [5, 14, 17, 21, 36], and solves a physics-constrained inverse problem in which the 3D RI (represented by an explicit neural field) and illumination parameters are jointly optimized to minimize the discrepancy between predicted and measured diffracted fluorescence images. Fourth, the demonstrated imaging depth differs. qOBM has reported thicknesses up to 145 μm in human skin [51], whereas FDT reconstructs volumes up to 300 μm in 3D-cultured bovine myotubes. Finally, qOBM is a single-modality phase/RI imaging technique, whereas FDT is inherently compatible with multimodal fluorescence-RI imaging by design, building on our prior demonstration of simultaneous fluorescence and RI decoding [17]. We have added a brief discussion of qOBM in the Introduction to clarify the relationship between these approaches.

Comment 2. Several experiments are presented to demonstrate the approach. The first is a simulation of the approach using a phantom comprising letters which shows good reconstruction. Experiments using a silica bead in PDMS are also shown. While an SNR range is evaluated, the figure of 5.78 is chosen as accurate but this analysis lacks quantitative comparisons and some description of the chosen threshold is needed. For example a halo effect is seen for the images at SNR 10.05 and SNR 20.80 not present in SNR 5.78. No errors are given for these reconstructions.

Response. We thank the reviewer for this comment. To address this concern, we have added new data that quantitatively analyze the reconstruction errors of the 3D RI of silica beads in PDMS from experimentally acquired fluorescence images at different SNR levels. Specifically, we use the root-mean-square error (RMSE) to evaluate the discrepancy between the reconstructed RI and the ground-truth RI of the silica bead, and the standard deviation (STD) to evaluate the consistency of the reconstructed RI across all voxels within the bead. According to this quantitative analysis, both the RMSE and the STD decrease substantially as the SNR increases. The ground-truth RI of the silica bead is 1.4570. When the SNR is low (SNR = 1.19), the reconstructed RI of the silica bead is 1.4442±0.0048, whereas at high SNR (SNR = 10.05), the reconstructed RI of the silica bead is 1.4584±0.00128, indicating the reconstruction error is substantially reduced. These results show that RI reconstruction accuracy improves significantly with increasing SNR.

Regarding the choice of threshold, we believe that the appropriate SNR threshold depends on the accuracy required for a given application. Therefore, in the revised manuscript, we removed the statement that the boundary case occurs at an image SNR of 5.78. For example, if one requires an RMSE between the reconstructed RI and the ground-truth RI below 0.01 and an STD of the reconstructed RI below 0.005,

then the corresponding threshold SNR is 10.05. For applications that do not require this level of accuracy, an SNR of 5.78 may still be acceptable.

Regarding the halo-like artifact observed in reconstructions from higher-SNR measurements, we would like to clarify that the dominant artifacts differ across SNR regimes. When using diffracted fluorescence images with low SNR, the reconstruction slightly overfits the high-frequency noise present in the low-SNR images. As a result, the artifacts appear as loss of low-frequency components together with high-frequency, speckle-like patterns. In contrast, when using diffracted fluorescence images with high SNR, the artifacts arise mainly from the limited frequency support of the OTF in the k_z direction. Although low-coherence illumination (i.e., fluorescence sources) helps fill the missing cone, the spatial-frequency coverage in the k_z direction remains lower than that in the k_x direction and limited by the finite number of raw measurements, leading to lower axial resolution than lateral resolution. Consequently, the reconstructed bead appears elongated along the axial direction with slight sidelobes ("halo effect"). In terms of robustness to SNR, the reconstruction from images with SNR $= 20.8$ is not noticeably limited by noise and instead mainly reflects the OTF of the imaging system, whereas the reconstruction from images with SNR $= 5.78$ exhibits mild noise-induced high-frequency artifacts. For SNRs below 5.78, the reconstructions exhibit pronounced high-frequency artifacts dominated by noise. In addition, reconstructing silica beads from experimental measurements is intrinsically challenging because of the relatively large RI contrast and the self-focusing effects induced by the beads, which may also contribute to the observed reconstruction artifacts. We have added these new quantitative results and discussion to the revised manuscript.

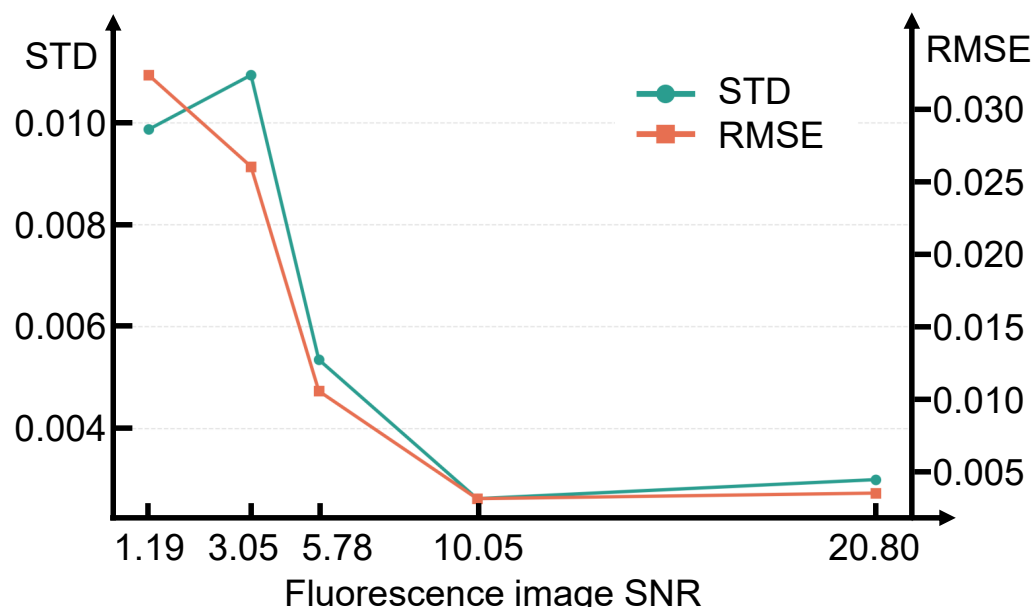

Fig. R11 Standard deviation (STD, blue) and root-mean-square-error (RMSE, red) of the reconstructed RI of silica beads in PDMS from experimentally acquired fluorescence images at different SNR levels.

Comment 3. An experiment with cells is shown next. While the reconstruction appears generally correct, there are errors of up to 0.2. The presented results suggest that the explicit model is qualitatively better than the implicit model. While the error is stated as minimal, 0.2 can be quite substantial and the authors are encouraged to more accurately discuss the fidelity. There is some concern here that the continuous refractive index changes of the cell are not as accurately reproduced as the technical

Image SNR	ΔRI RMSE	Pred. RI Mean ± STD
1.19	1.37×10^{-2}	$1.4442 \pm 4.80 \times 10^{-3}$
3.95	1.07×10^{-2}	$1.4477 \pm 5.32 \times 10^{-3}$
5.78	3.43×10^{-3}	$1.4548 \pm 2.60 \times 10^{-3}$
10.05	1.88×10^{-3}	$1.4584 \pm 1.28 \times 10^{-3}$
20.80	3.41×10^{-3}	$1.4601 \pm 1.46 \times 10^{-3}$

Table R9 Statistics of the reconstructed RI of silica beads in PDMS from experimentally acquired fluorescence images at different SNR levels (ground-truth RI = 1.4570).

sample. The course to fine approach that is described should do a better job at the cell edges where the largest errors appear.

Response. We thank the reviewer for this comment and helpful feedback. The error map was obtained by pixel-wise subtraction of the measured and predicted fluorescence intensity images. Therefore, the reported value of 0.2 reflects an absolute intensity difference rather than an RI difference. This error map is not an ideal measure of reconstruction fidelity, because even a slight lateral shift or magnification mismatch between two otherwise similar images can produce large pixel-wise errors, especially at cell edges. As shown in Fig. 1, the coarse-to-fine strategy enables reconstruction of both low-frequency components (i.e., continuous refractive index variations within the cells) and high-frequency components (i.e., cell edges). We therefore believe that the previous error map is misleading and have removed it. In the revised manuscript, we instead provide more rigorous quantitative evaluations.

Specifically, for samples with known ground-truth RI, such as simulated objects or silica microspheres with known RI, we validate reconstruction accuracy by directly comparing the reconstructed RI with the ground truth using quantitative metrics including MSE, SSIM, LPIPS, and PSNR. These metrics evaluate not only pixel-wise consistency but also overall structural similarity. We have added new results that quantitatively assess the reconstruction accuracy of FDT using simulated microbeads with known RI (Fig. R12, Table R10). The results show that the RI uncertainty is on the order of 10^{-4}, indicating that FDT achieves high RI reconstruction accuracy.

For biological samples, ground-truth RI is not accessible, and the measured diffracted fluorescence images are the only experimental ground-truth data available. In this case, we quantify reconstruction fidelity using the accumulated forward-model consistency between the full set of predicted and measured diffracted fluorescence images, with quantitative metrics including MSE, SSIM, LPIPS, and PSNR. Because the predicted diffracted fluorescence images are generated from the reconstructed RI and fluorescence-source parameters, strong agreement between the predicted and measured images supports the accuracy of the RI reconstruction. For the MDCK cells, the predicted diffracted fluorescence images closely match the experimentally measured fluorescence images, with MSE = 6.2900×10^{-4}, SSIM = 0.9160, LPIPS = 0.1417, and PSNR = 32.0135 (Table R11), supporting accurate RI reconstruction.

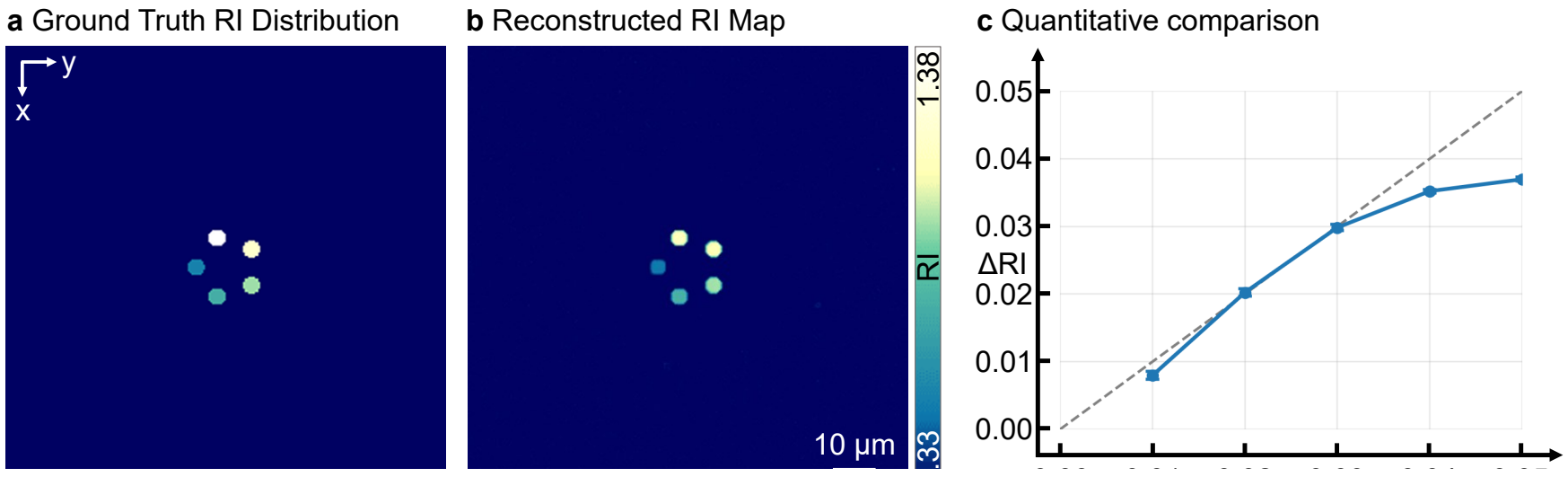

Fig. R12 Refractive index reconstruction of beads with varying RI. a. Ground-truth refractive index (RI) distribution of beads with RI contrasts ranging from 0.01 to 0.05. **b**. Reconstructed RI distribution of the simulated beads. **c**. Cross-sectional RI profiles of the beads comparing the reconstructed results with the ground truth.

Table R10 Quantitative evaluation of reconstructed RI accuracy with simulated beads

Bead	GT ΔRI	Num. of Voxels	ΔRI RMSE	Pred. ΔRI Mean ± STD
Bead 1	0.01	418	2.11×10^{-3}	$0.0080 \pm 5.84 \times 10^{-4}$
Bead 2	0.02	420	5.80×10^{-4}	$0.0202 \pm 5.29 \times 10^{-4}$
Bead 3	0.03	434	4.95×10^{-4}	$0.0298 \pm 4.66 \times 10^{-4}$
Bead 4	0.04	434	4.80×10^{-3}	$0.0352 \pm 2.64 \times 10^{-4}$
Bead 5	0.05	420	1.30×10^{-2}	$0.0370 \pm 2.82 \times 10^{-4}$

Table R11 Forward-model consistency metrics for biological specimens (no ground-truth RI).

**Sample**	**MSE**	**SSIM**	**LPIPS**	**PSNR**
MDCK cells	6.2900×10^{-4}	0.9160	0.1417	32.0135
Bovine myotube	7.7843×10^{-3}	0.7361	0.3024	22.7127
HEK cells	3.5858×10^{-3}	0.5126	0.5970	24.6652

Comment 4. The final experiment is reconstruction of bovine myotube, approx. 300 microns thick. The volume appears to be reasonably well reconstructed but is limited in that no cell details are visible and the claim that cells and muscle cells are differentiated is not well supported.

Response. We thank the reviewer for this comment and agree that our myotube results should be described more carefully. Our intent in this experiment is to demonstrate cellular-resolution RI reconstruction over a ~ 300 μm-thick volume and also FDT can reconstruct structures across various morphologies rather than to claim subcellular detail. We have therefore revised the text to remove any implication of subcellular resolution in the myotube dataset and to clarify that the resolvable features are at the cellular morphology level.

Regarding the claim of differentiation, We added a control experiment on a bovine myotube cultured under the same conditions and followed by immunofluorescence staining, which corroborates that these morphological transitions track molecular markers of myogenic differentiation (Fig. R13). First, the bright-field images show a

clear morphological transition of bovine muscle stem cells during differentiation. Before differentiation, cells exhibit a dispersed or loosely aggregated single-cell morphology. In contrast, after differentiation, the cells display elongated, fiber-like structures characteristic of myogenic differentiation. This morphological change is evident in bright-field images across different culture time points. Second, immunofluorescence staining further supports successful differentiation at both the molecular and structural levels; images were acquired with a confocal microscope (Leica SP8 STED 3X). MF20 staining indicates expression of myosin heavy chain, a marker of terminal myogenic differentiation. In parallel, phalloidin staining highlights the organization of F-actin, revealing aligned and elongated cytoskeletal structures consistent with myotube formation. Together with DAPI nuclear staining, these results demonstrate differentiated, fiber-like muscle structures rather than undifferentiated single cells. Therefore, based on the morphological differences observed with FDT, we can distinguish undifferentiated single stem cells from differentiated, fibrous muscle cells. We have added this new figure to the Supplementary Information.

Comment 5. The discussion is good but has some points that need to be better justified. In particular, the manuscript in general has a lot of discussion of epi-mode and multimode imaging yet these are not really the focus of the manuscript. The epi-mode is achieved by locating a fluorescent layer beneath the sample. This is a bit of an artificial simulation as it will not truly allow single ended access without adding a fluor underneath. While the discussion suggests use of endogenous fluorescence for this purpose, it is not likely to be strong enough for this method. Instead an injected contrast agent might be able to do the trick but limits the label free nature of the approach. The combination of fluorescence and quantitative phase imaging is a compelling modality but is not truly employed here. There is discussion of the combination though.

Response. We thank the reviewer's positive comment on the discussion section. To address the reviewer's concern, we have added new experiments demonstrating 3D RI reconstruction in thick biological specimens using fluorescence originating from within the sample itself, without any fluorescent substrate or transmission-side access. Specifically, we reconstruct the 3D RI of 3D-cultured HEK293T cells embedded in Matrigel and labeled with ActinGreen 488 (Fig. R14). This widely used cell line and standard labeling protocol provide a representative example of a fluorescence-labeled specimen with an arbitrary fluorescence distribution. In this experiment, two-photon excitation generates localized fluorescence from cells at $\sim 130\ \mu m$ depth below the sample surface, and this fluorescence acts as internal illumination for FDT. We then reconstruct the RI of overlying cellular structures from the resulting defocused, diffracted fluorescence images acquired in epi geometry. Because these measurements have lower SNR than those obtained with fluorescent paint (Figs. 2–4), we incorporate an early-stopping strategy during optimization to mitigate late-stage overfitting to measurement noise and to preserve reconstruction quality.

The long-term goal of our technique is to bridge labeled and label-free imaging modalities as they provide complementary biological information. The focus of the present manuscript is therefore a proof-of-concept demonstration that FDT can reconstruct 3D RI from fluorescence measurements in thicker, more complex specimens and with

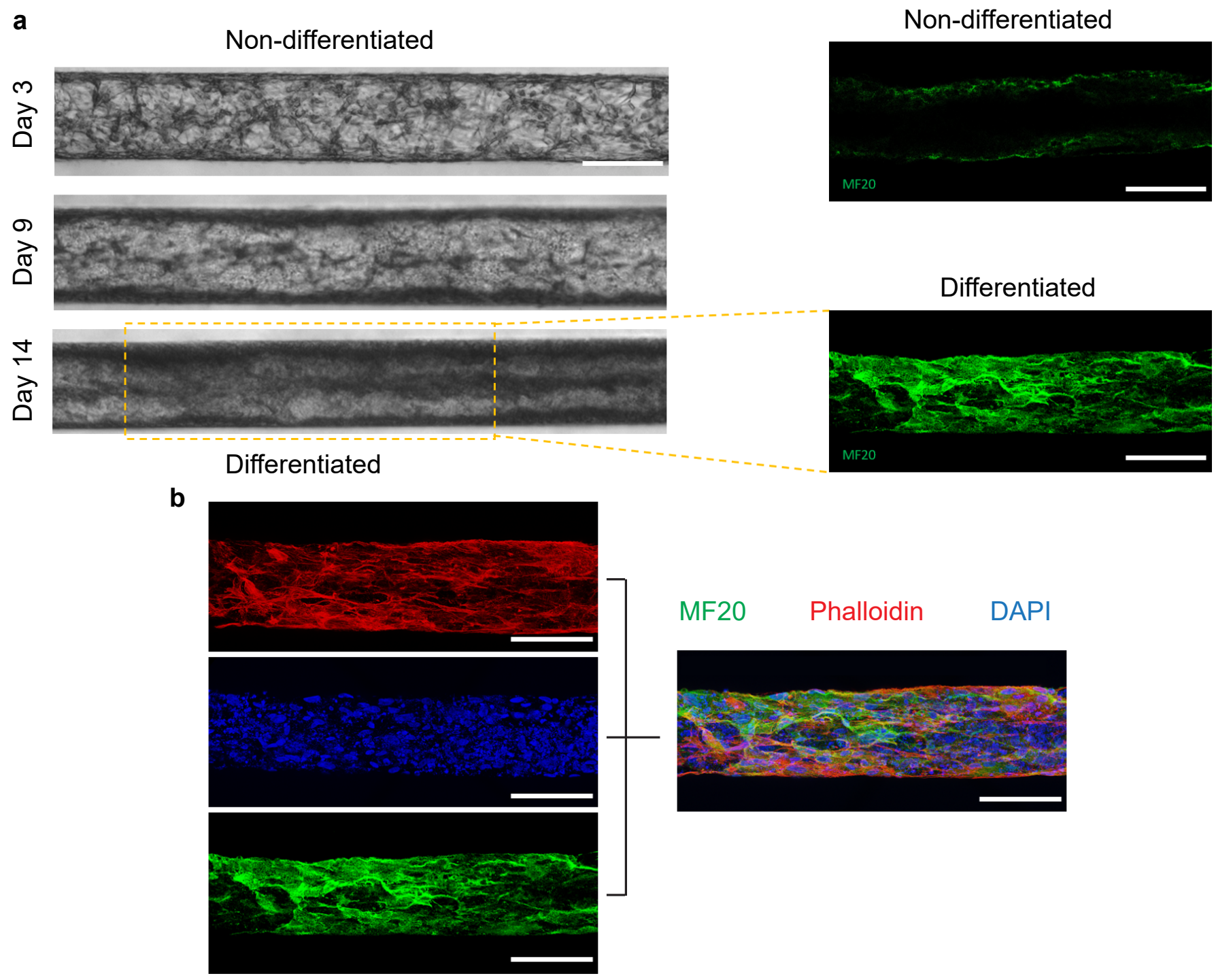

Fig. R13 Morphological changes of bovine muscle stem cells are closely linked to their differentiation status. a, The bright-field images show a clear morphological transition of bovine muscle stem cells during differentiation. Before differentiation, cells exhibit a dispersed or loosely aggregated single-cell morphology (top row). In contrast, after differentiation, the cells display elongated, fiber-like structures characteristic of myogenic differentiation (bottom row). This morphological change is evident from the bright-field images across different culture time points. The bright-field images are acquired using a Leica DMi8 microscope with LAS X software (Leica Microsystems). **b,** Confocal images of immunofluorescence staining support successful differentiation at both molecular and structural levels with the differentiated bovine myotube. MF20 (green channel) staining indicates the expression of myosin heavy chain, a marker of terminal myogenic differentiation. In parallel, Phalloidin (red channel) staining highlights the organization of F-actin, revealing aligned and elongated cytoskeletal structures consistent with myotube formation. Together with DAPI nuclear staining (blue channel), these results demonstrate differentiated, fiber-like muscle structures rather than undifferentiated single cells. The confocal images are acquired using a Leica SP8 STED 3X inverted microscope and LAS X software (Leica Microsystems) and analyzed in ImageJ. Scale bar in all images is 100 μm.

arbitrary fluorescence distributions, which substantially extends our prior work [17]. We include the newly added section below for the reviewer's reference.

3D RI reconstruction of fluorescence-labeled HEK cells without transmission-side access

To validate that FDT can reconstruct 3D RI when fluorescence originates *within* a thick specimen, we applied FDT to HEK293T cells cultured in a 3D Matrigel matrix

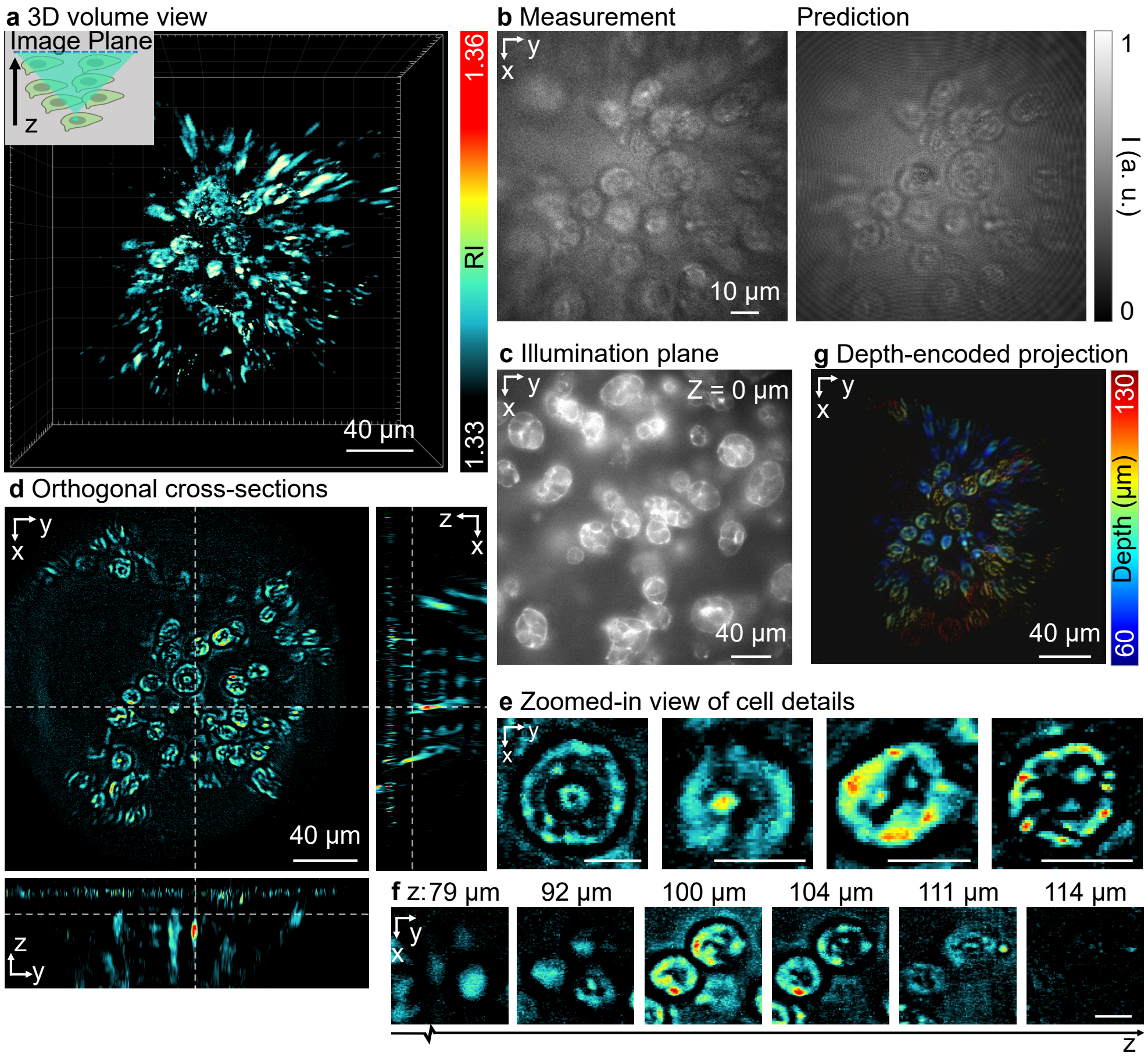

Fig. R14 3D RI reconstruction of ActinGreen 488-labeled HEK cells illuminated by fluorescence originating from the sample itself. (a) Reconstructed 3D RI of HEK cells over a 198 × 198 × 70 μm^3 volume. Inset: imaging geometry in which fluorescence-labeled cells serve as internal illuminators; their emitted fluorescence is diffracted by overlying cells and recorded as defocused images near the sample surface (see Extended Data Fig. 4d for details). (b) Representative diffracted fluorescence measurement (left) and corresponding prediction (right). (c) One-photon wide-field fluorescence image of HEK cells at $z = 0$ that act as fluorescence illuminators. (d) Orthogonal RI cross-sections ($x-y, x-z, y-z$); dashed lines indicate slice locations. (e) Zoomed-in RI images of representative cells showing clearly resolved membrane-scale features. Scale bar, 10 μm. (f) Zoomed-in RI z-stack demonstrating z-sectioning by resolving three cells at different depth. Scale bar, 10 μm. (g) Depth-encoded projection of the reconstructed RI volume.

and labeled with ActinGreen 488 (Fig. R14c; Extended Data Fig. 4d). Unlike the MDCK and bovine myotube experiments above, this measurement does not require access to the transmission side to apply fluorescent paint, making the approach applicable to specimens for which transmission-side access is not available. We used two-photon excitation to generate localized fluorescence within the cells at a depth of 130 μm below the sample surface (Fig. R14c). As fluorescence propagates upward

toward the objective, it is diffracted by the RI variations of overlying cells. All diffracted fluorescence images were acquired on a single camera plane near the sample surface (Fig. R14b, left; Extended Data Fig. 3d), and 282 images were selected based on SNR for 3D RI reconstruction.

Reconstructing the HEK-cell RI is more challenging than in the experiments above because the fluorescence images have lower SNR (Extended Data Fig. 3d; Fig. R14b), as ActinGreen-labeled actin is substantially dimmer than fluorescent paint. As shown in Fig. 2, low-SNR measurements can degrade RI reconstruction due to overfitting to noise. To mitigate this effect, we apply an *early-stopping* strategy: empirically, the model recovers physically consistent RI structure within the first several iterations, whereas prolonged optimization increasingly fits noise and degrades reconstruction quality. We therefore stop the optimization at iteration 60 (out of a maximum of 300), which also reduces reconstruction time.

By leveraging the early-stopping strategy, we reconstructed the 3D RI of HEK cells from low-SNR diffracted fluorescence images over a large volume ($198 \times 198 \times 70\ \mu m^3$, $600 \times 600 \times 40$ voxels). Forward-model consistency supports reconstruction fidelity, which shows the predicted diffracted fluorescence images closely match the experimental measurements with a MSE of 3.5858×10^{-3} (Fig. R14b, Table S6). Orthogonal cross-sections (Fig. R14d) resolve individual HEK cells with clear, enclosed cell boundaries. Representative zoomed-in $x - y$ view further reveals membrane-scale features down to $\sim 1 - 2\ \mu m$ (Fig. R14e), demonstrating high-resolution RI recovery. Moreover, a zoomed-in RI stack distinguishes cells at different axial planes without out-of-plane crosstalk (Fig. R14f). At the volume scale, the depth-encoded projection (Fig. R14g) localizes cells across the full axial extent, highlighting the strong z-sectioning of FDT in thick samples. Together, these results demonstrate that FDT can reconstruct 3D RI in thick biological specimens using fluorescence originating from the sample itself, without transmission-side access.

Comment 6. There are claims of subcellular resolution but these are not necessarily supported by the presented cell images. While there are some variations within the cell shown, it is not clear if these are accurately presenting cell structures. There is some question about what subcellular information is obtained and whether it is accurate or useful.

Response. We agree with the reviewer that our current data do not support a strong claim of subcellular resolution. We have therefore revised the manuscript to remove/-soften this wording and to consistently describe our demonstrated performance as cellular resolution. Furthermore, the newly added HEK-cell results (Fig. R14e) show membrane-scale RI contrast at cell boundaries, which is the smallest feature robustly supported by the data. This observation is consistent with the expected RI contrast between the lipid bilayer at the membrane and the surrounding cytoplasm.

Some additional points that should be addressed:

Line 65-66: An alternative approach would be to use spectrally distant illumination sources which would prevent crosstalk

Response. We agree with the reviewer that such crosstalk can be mitigated by using spectrally well-separated illumination sources. We have revised the statement accordingly to make it more rigorous.

Figure 2: Perhaps use different colors for Implicit neural field vs Explicit neural field in the legend Figure 2: Fig2(h) title should say 'data' not 'date'

Response. We thank the reviewer for the comment and have corrected the typo in the figure caption. We also used different colors for the implicit and explicit neural-field methods in the legend, as the reviewer suggested.

Line 266: Since the explicit so strongly outperforms implicit model, why hasn't it been developed before for DT? Are there Potential drawbacks for explicit model or is the reconstruction time a limitations.

Response. We thank the reviewer for this important question. We believe explicit neural fields have not been widely adopted in DT primarily for two reasons. First, explicit neural fields are relatively new. The explicit voxel/grid-based neural-field formulation (e.g., Plenoxels) was introduced for natural-scene reconstruction in computer vision in mid-2022 [39]. In contrast, implicit neural fields were introduced earlier in 2020 [38], and their adaptation to IDT/DT appeared in 2022 [6], as well as to other microscopy modalities since 2022 (e.g., ptychography [52], fluorescence microscopy [53], and structured illumination microscopy [54]). In our view, there is an inevitable lag between the development of new representations in computer vision and their adoption in computational imaging, especially when substantial changes to the forward model and optimization are required. Second, the earliest neural-field formulations were developed for intensity-only multi-view rendering, which aligns more directly with IDT than with standard ODT. Both implicit and explicit neural fields were originally designed to reconstruct 3D scenes from multiple intensity images acquired from different viewpoints. This setting is conceptually closer to IDT with intensity-only measurements than to standard ODT, which relies on interferometric measurements.

The primary practical limitation of the explicit representation is GPU memory (and correspondingly runtime) that scales with the reconstructed volume size. Because the RI volume is represented on a voxel-aligned grid, both memory usage and per-iteration computation scale approximately linearly with the number of voxels $N = N_x \times N_y \times N_z$ (for fixed batch size). For example, increasing the lateral number of pixels from 512×512 to 1024×1024 increases the total voxel count by approximately $4\times$, leading to a comparable increase in GPU memory footprint and runtime.. Increasing the number of z-planes N_z likewise produces near-linear growth in memory and runtime (Table R12). All experiments in this manuscript were performed on a single NVIDIA RTX A6000 GPU (48,GB) without multi-GPU or distributed training; the batch size was fixed to 20 for all reported results. In practice, on a 48,GB RTX A6000, volumes up to approximately $1024 \times 1024 \times 60$ voxels are feasible at batch size 20 without gradient checkpointing. We have added this discussion of scalability and practical limitations to the revised Discussion section and included additional details in the Supplementary document.

Table R12 Runtime and peak GPU memory usage per 100 iterations (batch size = 20). Experiments were conducted on a single NVIDIA RTX A6000 (48 GB).

Volume Size ($N_x \times N_y \times N_z$)	Time / 100 iters	Peak Memory
$512 \times 512 \times 20$	5 min	4 GB
$512 \times 512 \times 40$	10 min	7 GB
$512 \times 512 \times 60$	15 min	10 GB
$1024 \times 1024 \times 20$	8 min	17 GB
$1024 \times 1024 \times 40$	17 min	28 GB
$1024 \times 1024 \times 60$	33 min	39 GB

Fig 3: Label z-depth of Fig 3 (d-f). Label Fig 3 (d-f) explicit, Fig (g) implicit Give units for z-depth on Fig 3(h) top row Label explicit vs implicit on Fig 3(h)

Response. We thank the reviewer for the suggestion. We have added the labels in Fig. 3 as the reviewer suggested.

Fig 3: Line 307: Cells detached from substrate should have sharp RI increase, shouldn't course to fine process described in Fig 1 account for this?

Response. We thank the reviewer for the comment. If we understand correctly, the reviewer is asking whether the coarse-to-fine strategy should recover the sharp RI contrast expected for cells detached from the substrate. We indeed observe higher reconstructed RI in detached cells compared to live adherent cells (Fig. 3). The coarse-to-fine strategy primarily improves numerical conditioning and suppresses aliasing, which enables stable reconstruction and strong z-sectioning. In the zoomed-in RI z-stack, detached cells appear at higher z plane, and are clearly separable from adherent cells with minimal inter-plane crosstalk (Fig. 3h). We also resolve increased RI at cell boundaries (membrane-scale contrast), indicating that the reconstruction achieves high spatial resolution within the supported bandwidth.

Fig 3: Line 315: Higher RI because cell is detached from substrate and intracellular material condenses (other than just chromatin) [IE high RI on cell edge].

Response. We agree with the reviewer and have revised the statement accordingly to: "The relatively higher RI of dead cells compared with live cells probably indicates intracellular condensation during cell death."

Line 348: Does changing the z-depth of the fluorescence source improve the resolution? How/why was 210 micron chosen as illumination plane?

Response. We thank the reviewer for the comment. Changing the z-depth of the fluorescence source can affect the RI resolution, because it changes the effective illumination numerical aperture (NA, NA_{illu}). In our framework, resolution is set by both the objective NA (NA_{obj}) and the illumination NA (with $NA_{obj} \geq NA_{illu}$ in our experiments). According to prior work [5, 11–13], the lower bounds are $\Delta x = \lambda/[2(NA_{obj} + NA_{illu})]$ laterally and $\Delta z = \lambda/(2n - \sqrt{n^2 - NA_{obj}^2} - \sqrt{n^2 - NA_{illu}^2})$ axially. In FDT, $NA_{illu} = nsin(\theta_{illu})$ is determined by the separation between the

image plane and the fluorescence source (Fig. R15). While keeping all the other parameters the same, moving the fluorescence sources closer to the image plane (i.e., smaller Z) increases θ_{illu} and thus increases NA_{illu}, which improves the theoretical resolution. We have added an expanded discussion on reconstruction resolution in Results "Model validation with simulations and experiments using "UCDavis" patterns and microspheres of known RI".

In practice, the illumination plane is determined by both sample structures (Fig. R16) as well as the SNR of defocused, diffracted fluorescence images. In the bovine myotube experiments, the "illumination plane" is not freely chosen but rather set by the fluorescence paint layer on the dish surface beneath the specimen (Fig. R16c). What we can choose is the image plane, which sets the separation (Z) between the illumination plane and the detection plane. This choice is a practical trade-off among resolution, reconstructed FOV, and measurement SNR. Specifically, for fixed NA_{obj}, increasing Z increases reconstruction FOV but reduces the raw measurement SNR because the same fluorescence power is distributed over a larger area. It also increases defocus for structures far from the image plane, which appear blurred in the raw measurements and consequently reduces the effective resolution. For the bovine myotube, we therefore select the image plane to be near the center of the bovine myotube rather than near the top of the myotube to ensure high resolution reconstruction through the whole volume and optimize the SNR of forward images. In the newly added HEK experiment (Fig. R16d), fluorescence emitters are distributed throughout the sample volume, so we have more flexibility on selecting illumination positions and the image plane to achieve adequate SNR while maintaining sufficient resolution and FOV. We have clarified this point in the section of "Optical setup and fluorescence-illuminator characterization for FDT" in the revised manuscript.

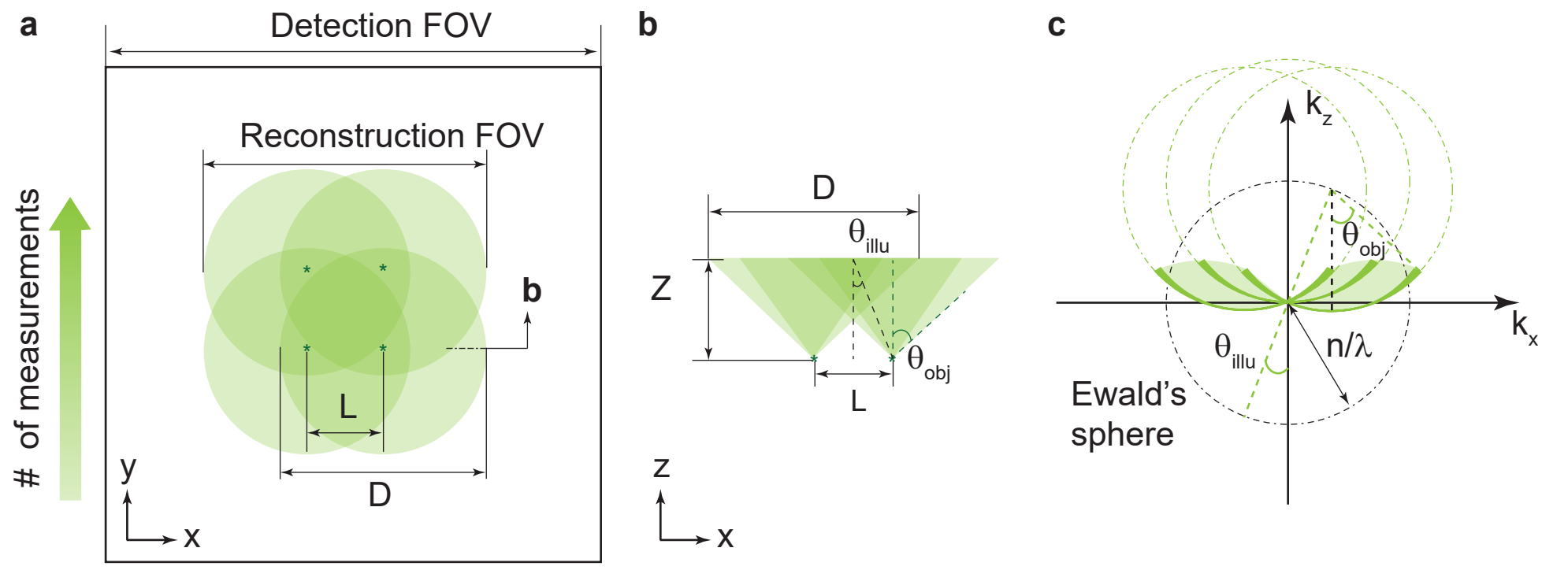

Fig. R15 Schematic diagram of the relationship between field-of-view (FOV), objective and illumination numerical aperture (NA_{obj}, NA_{illu}), optical transfer function (OTF), and the number of measurements.

In summary, this is an interesting advance for reconstructing refractive index distributions of thick samples. The images are compelling and there is innovation in the

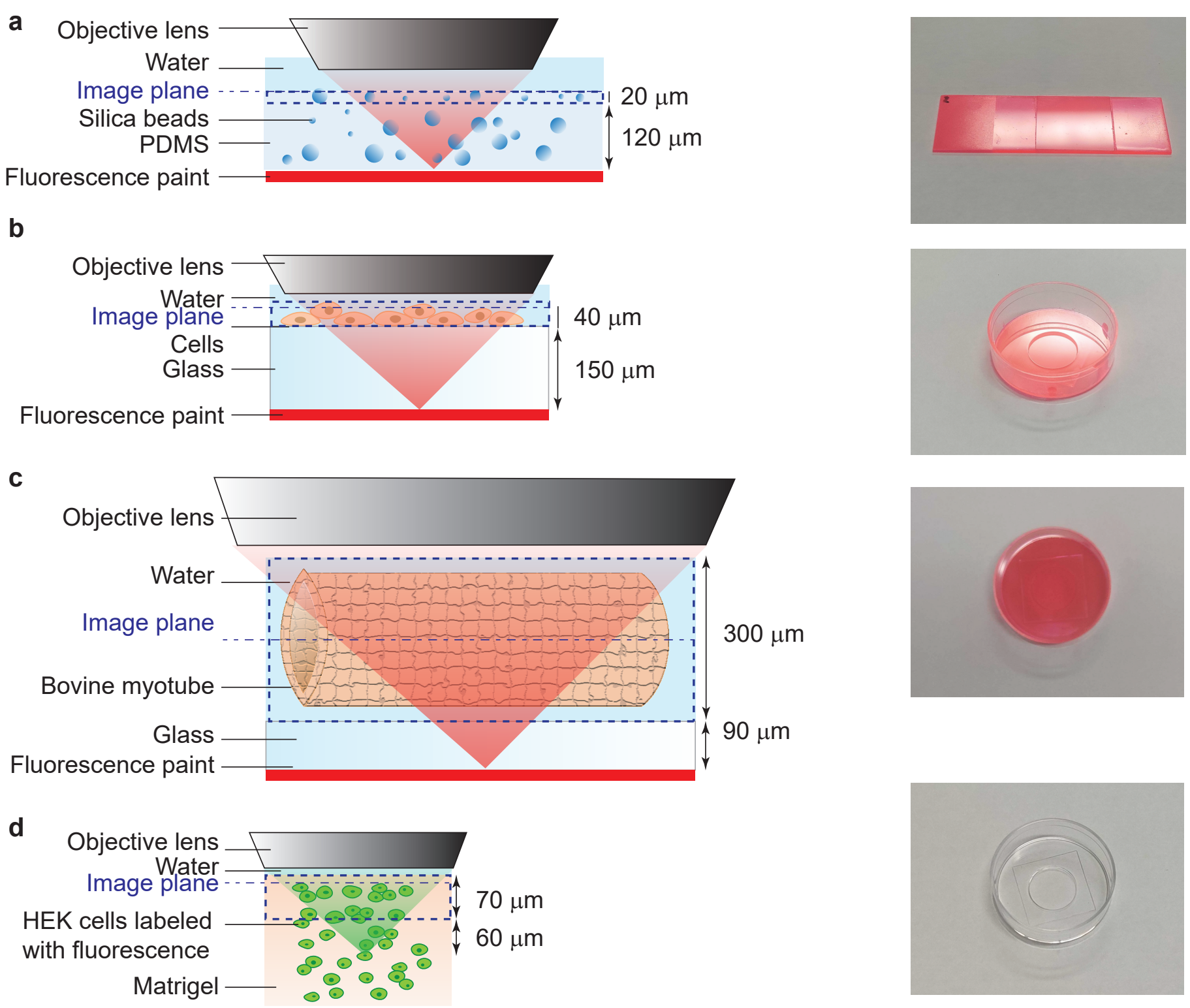

Fig. R16 Diagrams of samples and the position of fluorescence illumination source and image plane.

improvements to the approach over previous works. There are limitations in the presentation which could be addressed (as described above). Revision of the manuscript to address these issues could result in a publishable manuscript.

Response. We thank the reviewer for the thoughtful assessment and for recognizing the innovation and potential impact of our work. We have addressed each concern raised in the review point-by-point and revised the manuscript accordingly.

References

[1] Martins, I.S., Silva, H.F., Lazareva, E.N., Chernomyrdin, N.V., Zaytsev, K.I., Oliveira, L.M., Tuchin, V.V.: Measurement of tissue optical properties in a wide spectral range: a review [invited]. Biomed. Opt. Express **14**(1), 249–298 (2023)

[2] Choi, W., Fang-Yen, C., Badizadegan, K., Oh, S., Lue, N., Dasari, R.R., Feld, M.S.: Tomographic phase microscopy. Nat. Methods **4**(9), 717–719 (2007)

[3] Choi, Y., Hosseini, P., Choi, W., Dasari, R.R., So, P.T.C., Yaqoob, Z.: Dynamic speckle illumination wide-field reflection phase microscopy. Opt. Lett. **39**(20),

6062–6065 (2014)

[4] Kim, T., Zhou, R., Mir, M., Derin Babacan, S., Scott Carney, P., Goddard, L.L., Popescu, G.: White-light diffraction tomography of unlabelled live cells (2014)

[5] Tian, L., Waller, L.: Quantitative differential phase contrast imaging in an led array microscope. Opt. Express **23**(9), 11394–11403 (2015) https://doi.org/10.1364/OE.23.011394

[6] Liu, R., Sun, Y., Zhu, J., Tian, L., Kamilov, U.S.: Recovery of continuous 3d refractive index maps from discrete intensity-only measurements using neural fields. Nature Machine Intelligence **4**(9), 781–791 (2022)

[7] Born, M., Wolf, E.: Principles of Optics: Electromagnetic Theory of Propagation, Interference and Diffraction of Light. Elsevier, ??? (2013)

[8] Sung, Y., Choi, W., Fang-Yen, C., Badizadegan, K., Dasari, R.R., Feld, M.S.: Optical diffraction tomography for high resolution live cell imaging. Opt. Express **17**(1), 266–277 (2009) https://doi.org/10.1364/OE.17.000266

[9] Chen, M., Tian, L., Waller, L.: 3d differential phase contrast microscopy. Biomed. Opt. Express **7**(10), 3940–3950 (2016) https://doi.org/10.1364/BOE.7.003940

[10] Li, J., Zhou, N., Sun, J., Zhou, S., Bai, Z., Lu, L., Chen, Q., Zuo, C.: Transport of intensity diffraction tomography with non-interferometric synthetic aperture for three-dimensional label-free microscopy. Light Sci. Appl. **11**(1), 154 (2022)

[11] Sheppard, C.J.R., Gu, M., Kawata, Y., Kawata, S.: Three-dimensional transfer functions for high-aperture systems. J. Opt. Soc. Am. A, JOSAA **11**(2), 593–598 (1994)

[12] Lauer, V.: New approach to optical diffraction tomography yielding a vector equation of diffraction tomography and a novel tomographic microscope. J. Microsc. **205**(Pt 2), 165–176 (2002)

[13] Park, C., Shin, S., Park, Y.: Generalized quantification of three-dimensional resolution in optical diffraction tomography using the projection of maximal spatial bandwidths. J. Opt. Soc. Am. A Opt. Image Sci. Vis. **35**(11), 1891–1898 (2018)

[14] Wolf, E.: Three-dimensional structure determination of semi-transparent objects from holographic data. Optics Communications **1**(4), 153–156 (1969) https://doi.org/10.1016/0030-4018(69)90052-2

[15] Kang, Y.G., Park, K., Hyeon, M.G., Yang, T.D., Choi, Y.: Three-dimensional imaging in reflection phase microscopy with minimal axial scanning. Opt. Express **31**(26), 44741–44753 (2023) https://doi.org/10.1364/OE.510519

[16] Theer, P., Denk, W.: On the fundamental imaging-depth limit in two-photon

microscopy. J. Opt. Soc. Am. A Opt. Image Sci. Vis. **23**(12), 3139–3149 (2006)

[17] Xue, Y., Ren, D., Waller, L.: Three-dimensional bi-functional refractive index and fluorescence microscopy (brief). Biomed. Opt. Express **13**(11), 5900–5908 (2022) https://doi.org/10.1364/BOE.456621

[18] Betzig, E., Patterson, G.H., Sougrat, R., Lindwasser, O.W., Olenych, S., Bonifacino, J.S., Davidson, M.W., Lippincott-Schwartz, J., Hess, H.F.: Imaging intracellular fluorescent proteins at nanometer resolution. Science **313**(5793), 1642–1645 (2006)

[19] Rust, M.J., Bates, M., Zhuang, X.: Sub-diffraction-limit imaging by stochastic optical reconstruction microscopy (STORM). Nat. Methods **3**(10), 793–795 (2006)

[20] Kamilov, U.S., Papadopoulos, I.N., Shoreh, M.H., Goy, A., Vonesch, C., Unser, M., Psaltis, D.: Learning approach to optical tomography. Optica **2**(6), 517–522 (2015) https://doi.org/10.1364/OPTICA.2.000517

[21] Chen, M., Ren, D., Liu, H.-Y., Chowdhury, S., Waller, L.: Multi-layer born multiple-scattering model for 3d phase microscopy. Optica **7**(5), 394–403 (2020) https://doi.org/10.1364/OPTICA.383030

[22] Li, T., Zhu, J., Shen, Y., Tian, L.: Reflection-mode diffraction tomography of multiple-scattering samples on a reflective substrate from intensity images. Optica **12**(3), 406–417 (2025) https://doi.org/10.1364/OPTICA.547372

[23] Park, Y., Popescu, G., Badizadegan, K., Dasari, R.R., Feld, M.S.: Diffraction phase and fluorescence microscopy. Opt. Express **14**(18), 8263–8268 (2006) https://doi.org/10.1364/OE.14.008263

[24] Zuo, C., Chen, Q., Tian, L., Waller, L., Asundi, A.: Transport of intensity phase retrieval and computational imaging for partially coherent fields: The phase space perspective. Opt. Lasers Eng. **71**, 20–32 (2015)

[25] Teague, M.R.: Deterministic phase retrieval: a green's function solution. J. Opt. Soc. Am. **73**(11), 1434–1441 (1983) https://doi.org/10.1364/JOSA.73.001434

[26] Bastiaans, M.J.: Application of the wigner distribution function to partially coherent light. JOSA A **3**(8), 1227–1238 (1986)

[27] Boashash, B.: Estimating and interpreting the instantaneous frequency of a signal. I. fundamentals. Proc. IEEE Inst. Electr. Electron. Eng. **80**(4), 520–538 (1992)

[28] Wax, A., Yang, C., Backman, V., Badizadegan, K., Boone, C.W., Dasari, R.R., Feld, M.S.: Cellular organization and substructure measured using angle-resolved low-coherence interferometry. Biophys. J. **82**(4), 2256–2264 (2002)

[29] Butola, A., Coucheron, D.A., Szafranska, K., Ahmad, A., Mao, H., Tinguely, J.-C., McCourt, P., Senthilkumaran, P., Mehta, D.S., Agarwal, K., Ahluwalia, B.S.: Multimodal on-chip nanoscopy and quantitative phase imaging reveals the nanoscale morphology of liver sinusoidal endothelial cells. Proc. Natl. Acad. Sci. U. S. A. **118**(47), 2115323118 (2021)

[30] Kandel, M.E., Hu, C., Naseri Kouzehgarani, G., Min, E., Sullivan, K.M., Kong, H., Li, J.M., Robson, D.N., Gillette, M.U., Best-Popescu, C., Popescu, G.: Epi-illumination gradient light interference microscopy for imaging opaque structures. Nat. Commun. **10**(1), 4691 (2019)

[31] Ledwig, P., Robles, F.E.: Epi-mode tomographic quantitative phase imaging in thick scattering samples. Biomed. Opt. Express **10**(7), 3605–3621 (2019)

[32] Lee, M., Hugonnet, H., Park, Y.: Inverse problem solver for multiple light scattering using modified born series. Optica **9**(2), 177 (2022)

[33] Kim, K., Park, W.S., Na, S., Kim, S., Kim, T., Heo, W.D., Park, Y.: Correlative three-dimensional fluorescence and refractive index tomography: bridging the gap between molecular specificity and quantitative bioimaging. Biomed. Opt. Express **8**(12), 5688–5697 (2017) https://doi.org/10.1364/BOE.8.005688

[34] Shin, S., Kim, D., Kim, K., Park, Y.: Super-resolution three-dimensional fluorescence and optical diffraction tomography of live cells using structured illumination generated by a digital micromirror device. Sci. Rep. **8**(1), 9183 (2018)

[35] Chowdhury, S., Eldridge, W.J., Wax, A., Izatt, J.A.: Structured illumination multimodal 3d-resolved quantitative phase and fluorescence sub-diffraction microscopy. Biomed. Opt. Express **8**(5), 2496–2518 (2017) https://doi.org/10.1364/BOE.8.002496

[36] Chowdhury, S., Chen, M., Eckert, R., Ren, D., Wu, F., Repina, N., Waller, L.: High-resolution 3d refractive index microscopy of multiple-scattering samples from intensity images. Optica **6**(9), 1211–1219 (2019) https://doi.org/10.1364/OPTICA.6.001211

[37] Beck, A., Teboulle, M.: A fast iterative shrinkage-thresholding algorithm for linear inverse problems. SIAM J. Imaging Sci. **2**(1), 183–202 (2009)

[38] Mildenhall, B., Srinivasan, P.P., Tancik, M., Barron, J.T., Ramamoorthi, R., Ng, R.: Nerf: Representing scenes as neural radiance fields for view synthesis. In: ECCV (2020)

[39] Fridovich-Keil, S., Yu, A., Tancik, M., Chen, Q., Recht, B., Kanazawa, A.: Plenoxels: Radiance fields without neural networks. In: Proceedings of the IEEE/CVF Conference on Computer Vision and Pattern Recognition, pp. 5501–5510 (2022)

[40] Streibl, N.: Phase imaging by the transport equation of intensity. Optics Communications **49**(1), 6–10 (1984) https://doi.org/10.1016/0030-4018(84)90079-8

[41] Waller, L., Tian, L., Barbastathis, G.: Transport of intensity phase-amplitude imaging with higher order intensity derivatives. Opt. Express **18**(12), 12552–12561 (2010) https://doi.org/10.1364/OE.18.012552

[42] Tian, L., Waller, L.: 3D intensity and phase imaging from light field measurements in an LED array microscope. Optica, OPTICA **2**(2), 104–111 (2015)

[43] Ling, R., Tahir, W., Lin, H.-Y., Lee, H., Tian, L.: High-throughput intensity diffraction tomography with a computational microscope. Biomed. Opt. Express **9**(5), 2130–2141 (2018) https://doi.org/10.1364/BOE.9.002130

[44] Zepeda, D., Li, Y., Xue, Y.: Scattering correction through fourier-domain intensity coupling in two-photon microscopy (2P-FOCUS). Photonics Res. **13**(4), 845–864 (2024)

[45] He, R., Li, Y., Urbina, B., Wan, J., Xue, Y.: Compressive fourier-domain intensity coupling (C-FOCUS) enables near-millimeter deep imaging in the intact mouse brain in vivo. arXiv [physics.optics] (2025) [physics.optics]

[46] Xue, Y., Berry, K.P., Boivin, J.R., Wadduwage, D., Nedivi, E., So, P.T.C.: Scattering reduction by structured light illumination in line-scanning temporal focusing microscopy. Biomed. Opt. Express, BOE **9**(11), 5654–5666 (2018)

[47] Xue, Y., Boivin, J.R., Wadduwage, D.N., Park, J.K., Nedivi, E., So, P.T.C.: Multiline orthogonal scanning temporal focusing (mosTF) microscopy for scattering reduction in in vivo brain imaging. Sci. Rep. **14**(1), 10954 (2024)

[48] Zheng, C., Park, J.K., Yildirim, M., Boivin, J.R., Xue, Y., Sur, M., So, P.T.C., Wadduwage, D.N.: De-scattering with excitation patterning enables rapid wide-field imaging through scattering media. Sci Adv **7**(28) (2021)

[49] Ledwig, P., Robles, F.E.: Quantitative 3D refractive index tomography of opaque samples in epi-mode. Optica **8**(1), 6–14 (2021)

[50] Li, Z., Casteleiro Costa, P., Serafini, C., Bharadwaj, S., Guang, Z., Robles, F.E.: Experimental assessment of the optical transfer function for quantitative oblique back illumination microscopy (qOBM). Opt. Express **33**(3), 5088–5098 (2025)

[51] Casteleiro Costa, P., Bharadwaj, S., Li, Z., Kaza, N., Lopez-Esteva, M., Lien, A., Haider, B., Robles, F.E.: Single capture quantitative oblique back-illumination microscopy. bioRxivorg, 2025–0729667497 (2025)

[52] Zhou, H., Feng, B.Y., Guo, H., Lin, S.S., Liang, M., Metzler, C.A., Yang, C.:

Fourier ptychographic microscopy image stack reconstruction using implicit neural representations. Optica **10**(12), 1679–1687 (2023) https://doi.org/10.1364/OPTICA.505283

[53] Zhang, O., Zhou, H., Feng, B.Y., Larsson, E.M., Alcalde, R.E., Yin, S., Deng, C., Yang, C.: Single-shot volumetric fluorescence imaging with neural fields. arXiv preprint arXiv:2405.10463 (2024)

[54] Cao, R., Liu, F.L., Yeh, L.-H., Waller, L.: Dynamic structured illumination microscopy with a neural space-time model. In: 2022 IEEE International Conference on Computational Photography (ICCP), pp. 1–12 (2022). IEEE

Response to the Reviewers

General Response

Reviewer #1 (Remarks to the Author):

I have now finished carefully reading the new submission provided by Yi Xue and co-authors entitled: "Fluorescence-illuminated Diffraction Tomography using Explicit Neural Fields". I would like to thank the authors for their constructive answer and for taking my comments into account. I believe the article can now be published in Nature Communications.

We thank the reviewer for recognizing our efforts to revise the manuscript and address the concerns. We are grateful for the reviewer's positive assessment and recommendation for publication in *Nature Communications*.

Minor comment : the code is actually not visible in the provided link.

We thank the reviewer for pointing this out. We have corrected the source code link to https://github.com/Xue-Lab-COBI/fdt_code and uploaded the code to this repository. The revised Code Availability statement is:

"The code used for reproducing the results in the manuscript is available at FDT code."

Reviewer #1 (Remarks on code availability):

The code is not available or visible via the link provided by the authors: https://github.com/cubhe/fdt_code

We thank the reviewer for this comment. Please see our response to the previous comment, where we addressed this issue by correcting the source-code link and uploading the code to the repository.

Reviewer #2 (Remarks to the Author):

The authors have made substantial efforts to address the concerns raised in my previous review. In particular, the revised manuscript is significantly clearer regarding

the imaging geometry, the role of the fluorescent substrate in the original experiments, and the distinction between epi-collection geometry and true transmission-side independence. I appreciate that the authors have clarified the use of the fluorescent paint layer in the silica bead, MDCK cell, and bovine myotube experiments, and, more importantly, have added a new experiment using fluorescence-labeled HEK293T cells in which the fluorescence originates from within the sample itself without requiring transmission-side access. This additional demonstration directly addresses my major concern regarding the applicability of the method beyond substrate-assisted configurations.

The authors have also satisfactorily strengthened the technical foundation of the work. The discussion of the partially coherent fluorescence source and the role of the partially coherent mask is now much more precise, and the revised manuscript appropriately frames the mask as a practical weighting strategy to improve reconstruction robustness rather than as a complete physical treatment of fluorescence coherence. The authors also clarify the applicability and limitations of the Born approximation in weakly scattering samples. In addition, the newly added quantitative analysis of lateral and axial resolution, including theoretical estimates, PSF/MTF analysis, and simulated bead-separation tests, substantially improves the support for the claimed resolution and z-sectioning capability. Finally, the added information on runtime, GPU memory requirements, and scaling behavior adequately addresses my previous concern regarding computational feasibility and broader adoption.

Overall, I find that the authors have responded carefully and constructively to the major issues raised in my initial report, and the manuscript has been significantly improved. I have one remaining minor concern. While the revised text is much more careful than the original version, the manuscript would benefit from maintaining a very explicit distinction between two experimentally demonstrated capabilities:

(1) 3D RI reconstruction of thick, label-free biological tissue using a fluorescent substrate beneath the specimen, as demonstrated with the bovine myotube sample; and

(2) 3D RI reconstruction without transmission-side access, as demonstrated using fluorescence-labeled HEK293T cells in which the fluorescence originates from within the sample itself.

These are both important advances, but they are not demonstrated simultaneously in the same experiment. Therefore, I recommend that the authors further ensure that the Abstract, Discussion, and Conclusion do not unintentionally imply that the manuscript has already demonstrated substrate-free, transmission-side-independent 3D RI reconstruction of thick label-free tissue. A brief clarifying statement explicitly distinguishing these two regimes would make the scope of the work more precise and prevent overinterpretation of its current in vivo relevance.

With this minor clarification, I believe the manuscript would be suitable for publication.

We thank the reviewer for recognizing our efforts to revise the manuscript and address the concerns. We are grateful for the reviewer's positive assessment, thorough summary of our work, and recommendation for publication in *Nature Communications* pending one final minor revision.

To address this concern, we have revised the Abstract, Discussion, and Conclusion to explicitly distinguish the two experimental regimes: (1) 3D RI reconstruction of thick, label-free biological tissue using a fluorescent substrate beneath the specimen and (2) 3D RI reconstruction without transmission-side access using fluorescence emitted by fluorescence-labeled cells within the sample. We now clearly state that these two capabilities were demonstrated separately. The revised statements are provided below for the reviewer's convenience.

Abstract: "We demonstrate FDT in two experimental regimes: reconstructing the 3D RI of label-free tissues up to 300 μm thick placed on a fluorescent substrate, and reconstructing the 3D RI of fluorescence-labeled, 3D-cultured cells without transmission-side access using fluorescence emitted by the cells themselves."

Discussion: "We have demonstrated that FDT enables 3D RI reconstruction from diffracted fluorescence images acquired in two separate experimental regimes: (1) label-free tissue within a $530 \times 530 \times 300$ μm^3 volume placed on a fluorescent substrate and (2) fluorescence-labeled, 3D-cultured HEK cells within a $198 \times 198 \times 70$ μm^3 volume imaged without transmission-side access, using fluorescence emitted by the cells themselves."

Conclusion: "In summary, we present FDT as a new imaging modality for reconstructing the 3D RI of biological specimens from diffracted fluorescence images acquired in an epi-detection (reflection-mode) geometry. In separate experiments, the fluorescence originated either from a fluorescent substrate beneath a label-free specimen or from fluorescence-labeled cells within the specimen."

Reviewer #4 (Remarks to the Author):

This revised manuscript was widely critiqued by four different reviewers with extensive revisions made in an effort to address them. In addition, a 67 page response was included also in an effort to rebut reviewers concerns.

In general, the approach is sound and capable of reconstructing refractive index distributions from fluorescent light. As noted previously, parts of this approach have been published before by these authors and others. Thus, the question remains is the work presented here a novel combination. The use of explicit neural fields appears to have utility but have been shown before. The novelty must then be viewed as the two photon fluorescence used as the illumination source. The data supporting this have appeared in numerous pre-prints and conference presentations. However, there is a novel aspect here that can be viewed as a significant advance, worthy of publication at this level.

We thank the reviewer for recognizing our efforts to revise the manuscript and for acknowledging that the proposed approach is technically sound. We agree that using two-photon-excited fluorescence as the illumination source is a major novelty of FDT.

Compared with our previous work [1], which required fluorescent beads embedded within the sample, FDT can use either a fluorescent substrate or fluorescence-labeled cells with different spatial distributions and densities as the fluorescence source. We have also compared FDT with existing multimodal fluorescence–phase imaging methods [2–12], which primarily operate in transmission mode. In contrast, FDT operates in reflection mode and therefore provides a pathway toward imaging specimens without transmission-side access. Existing reflection-mode phase-imaging methods [13–21] are generally phase-only techniques, are limited to thin samples, or require axial scanning. FDT instead enables multimodal fluorescence–phase imaging of thick biological specimens without axial scanning.

FDT also adapts explicit neural fields, originally developed for 3D scene reconstruction [22], to 3D RI reconstruction. To our knowledge, this is the first application of explicit neural fields to this problem. Compared with implicit neural-field methods [23], FDT reconstructs substantially thicker specimens with higher computational efficiency, as demonstrated in Figs. 1 and 3 and Table S5.

The preprint and conference presentations describe the development of this same work and do not constitute separate prior publications. We appreciate the reviewer's recognition that the novel aspect represents a significant advance worthy of publication in *Nature Communications*.

The primary concerns of this reviewer previously focused on the overstated claims of the original manuscript. The author addresses some of these by softening the language in some places while in others, a lot more new data were provided. Primary concern is that the approach is touted as label free but requires a contrast agent. The author addresses of this concern by delineating experiments with a fluorescent substrate from those where the label is within the sample. In neither case is the technique truly label-free in the sense that the sample is not perturbed. The experiments without the substrate underneath use bright labels within the cell but these are exogenous. Similarly, the experiment with the bovine myotubule requires a fluorescent substrate. The question remains what is the benefit of this approach when any other incoherent light source could be used on the far side of the sample, with less instrument complexity. New analysis is presented to assert that the myotube images are accurate. It appears that the reconstruction is compared to the original measured data and good metrics are obtained. It is unclear how this justifies that accurate reconstructions are obtained. Fluorescence imaging of the myotubule are now presented but buried at the end of supplemental section. Overall, I do not feel that the shortcomings identified by this reviewer and others were well addressed.

We thank the reviewer for pointing out the remaining concerns. To address the primary concern, we have revised the manuscript to clarify explicitly that FDT is a multimodal fluorescence–phase imaging technique rather than a purely label-free imaging technique. FDT requires a fluorescence source in all demonstrated configurations. To avoid potential misunderstanding, we have also removed from the Discussion the description of a possible implementation of FDT using autofluorescence as a fully label-free approach.

Multimodal fluorescence and phase imaging provides distinct and complementary information about biological samples. Fluorescence labeling provides molecular specific information, whereas quantitative phase or RI imaging provides label-free structural and biophysical information, including information related to cellular mass and mass transport. FDT is motivated by the value of integrating these two complementary imaging contrasts, as are many previously reported multimodal fluorescence–phase imaging techniques [2–12]. By demonstrating 3D RI reconstruction of label-free bovine myotubes placed on a fluorescent substrate and of fluorescence-labeled cells using fluorescence emitted by the cells themselves, FDT shows flexibility with respect to the spatial distribution and density of the fluorescence sources. Therefore, FDT is not intended to be described as a purely label-free technique. Rather, it is a multimodal technique that combines fluorescence-labeled imaging with label-free RI imaging. For example, when imaging HEK cells, fluorescence serves not only as an incoherent illumination source for RI reconstruction but also provides spatially localized, molecular specific information about actin within the cells.

(The question remains what is the benefit of this approach when any other incoherent light source could be used on the far side of the sample, with less instrument complexity.)

If we understand the reviewer correctly, "any other incoherent light source could be used on the far side of the sample" refers to placing an incoherent illumination source on the transmission side of the specimen, such that the source and detector are located on opposite sides. This is the conventional geometry used in most quantitative phase-imaging systems.

The first advantage of FDT over such an approach is that FDT operates in an epi-detection (reflection-geometry) and therefore does not necessarily require transmission-side access to the specimen. Consequently, FDT has the potential to image specimens for which the transmission side is inaccessible, including bulky tissues and in vivo animals, where few transmitted photons can be collected or the opposite side of the specimen cannot be physically accessed. Specifically, FDT has demonstrated 3D RI reconstruction of biological specimens up to approximately 300 μm thick using a fluorescent substrate and of specimens up to approximately 70 μm thick without a fluorescent substrate. In comparison, previously reported multimodal fluorescence–phase imaging techniques operating in transmission geometry have generally reconstructed the 3D RI of single layers of cells < 20 μm thick. The reconstruction of the 3D RI of fluorescence-labeled HEK cells demonstrates that FDT can operate without transmission-side access.

The second advantage of FDT is that it enables multimodal fluorescence–phase imaging. In the HEK-cell experiment, two-photon-excited fluorescence from fluorescently labeled actin serves both as an incoherent illumination source for RI reconstruction and as a source of spatially localized, molecularly specific information about actin within the cells. This configuration enables fluorescence and RI information from the same specimen to be acquired simultaneously using the same excitation and detection platform, potentially allowing biologists to investigate relationships between

fluorescence-labeled structures and surrounding label-free structures. We have clarified this point in the revised Discussion.

(New analysis is presented to assert that the myotube images are accurate. It appears that the reconstruction is compared to the original measured data and good metrics are obtained. It is unclear how this justifies that accurate reconstructions are obtained.)

We thank the reviewer for pointing out the need to clarify the accuracy of reconstruction. Throughout this work, we evaluate reconstruction fidelity using accumulated forward-model consistency, which quantifies the agreement between the experimentally measured diffracted fluorescence intensity images and the corresponding images predicted from the reconstructed 3D RI distribution using the forward model. This approach is well established in intensity diffraction tomography (IDT) [2–10], in which the phase or RI distribution cannot be measured directly but must instead be reconstructed from intensity-only measurements. Because the RI distribution is not directly accessible experimentally, whereas the intensity images are directly measured, IDT methods evaluate reconstruction fidelity by using the estimated RI distribution to predict the measured intensity images through the forward model. The relationship between this measurement-domain consistency and RI-domain accuracy is validated using samples with known RI. We have revised the section "Model architecture of FDT for RI reconstruction" and Discussion to clarify this point.

During reconstruction, FDT solves an inverse problem by iteratively updating the 3D RI distribution to minimize the discrepancy between the measured diffracted fluorescence intensity images and those predicted by the forward model under different illumination conditions. Because hundreds of images are acquired at different illumination positions and angles, the reconstruction is constrained by a large and diverse set of independent measurements. If a single estimated 3D RI distribution consistently reproduces the measurements across all illumination conditions, this demonstrates that the reconstruction is strongly constrained by and consistent with the complete experimental dataset. A detailed description of the forward model and reconstruction procedure is provided in the section "Model architecture of FDT for RI reconstruction."

To validate this approach, we reconstruct the 3D RI of samples of known ground-truth RI. Specifically, in simulations and experiments using silica microspheres with known refractive indices, we compare the RI reconstructed by minimizing the accumulated forward-model discrepancy with the corresponding ground-truth RI. The close agreement between the reconstructed and ground-truth RI distributions demonstrates that accumulated forward-model consistency can reliably reflect RI reconstruction accuracy under the tested conditions. Details are provided in the section "Model validation with simulations and experiments using 'UCDavis' patterns and microspheres of known RI."

After validating this relationship using samples with known RI, we use accumulated forward-model consistency to evaluate reconstructions of heterogeneous biological specimens, such as the myotube sample, for which an independent ground-truth 3D RI distribution is unavailable.

(Fluorescence imaging of the myotubule are now presented but buried at the end of supplemental section. Overall, I do not feel that the shortcomings identified by this reviewer and others were well addressed.)

We thank the reviewer for raising this point. We would like to clarify that the fluorescence images of the myotubes were placed in the Supplementary Information rather than in the main figure to avoid creating the misleading impression that the myotube reconstructed in Fig. 4 was fluorescence-labeled or that its diffracted fluorescence images were acquired by exciting fluorophores within the myotube.

In Fig. 4, the myotube specimen is label-free and is placed on a fluorescent substrate. The diffracted fluorescence images shown in Fig. 4b are acquired by exciting the fluorescent substrate beneath the specimen. In contrast, Supplementary Fig. S6 shows immunofluorescence images of separate myotube samples cultured under the same conditions. These control experiments in Fig. S6 are included only to illustrate the morphological changes associated with myogenic differentiation. The myotubes shown in Supplementary Fig. S6 are not the same specimen reconstructed in Fig. 4. While all subplots in Fig. 4 are acquired or reconstructed from the same myotube, the fluorescence images in Fig. S6 were neither used in the 3D RI reconstruction nor acquired using the FDT system during the experiment, therefore they were included in the Supplementary Information.

By contrast, Fig. 5 presents 3D RI reconstruction of fluorescence-labeled HEK cells. In this experiment, the diffracted fluorescence images are acquired by exciting fluorophores within the cells themselves. The fluorescence image showing the fluorescence distribution in the same specimen is therefore included directly in Fig. 5c.

We believe that retaining the images of the immunofluorescence-stained myotube samples in the Supplementary Information most clearly distinguishes the experimental configurations used in Figs. 4 and 5 and avoids implying that the label-free myotube reconstruction was performed using fluorescence originating from within the myotube itself. We hope that these additional explanations and manuscript revisions more clearly address the reviewer's concerns and clarify the scope, validation, and limitations of the proposed method.

A new experiment is included which uses fluorescently labeled cells. Images are reconstructed but no effort is made to prove that they are accurate. Instead, the features are just asserted to be 'membrane-scale'. It's unclear if these are cell structures or ringing artifacts. These experimental results are not as well presented as others and are used to justify that images are obtained without the substrate. In my view, these do not add to the clarity of the manuscript.

We thank the reviewer for raising this concern. We address the concern point-by-point below.

(Images are reconstructed but no effort is made to prove that they are accurate.)

Regarding the accuracy of the reconstructed HEK-cell images, we evaluate reconstruction fidelity using accumulated forward-model consistency, as we do for all

reconstructions in this work and discussed in our response to the preceding comment. This metric quantifies the discrepancy between the experimentally measured diffracted fluorescence images and the corresponding images predicted from the reconstructed 3D RI through the forward model. Because the ground-truth 3D RI of a heterogeneous biological specimen cannot be measured directly, forward-model consistency is the primary metric used in intensity diffraction tomography to evaluate whether the reconstructed RI is consistent with the measured data. In this experiment, a single reconstructed 3D RI distribution is required to reproduce hundreds of diffracted fluorescence images acquired under different excitation positions and illumination angles. The predicted images closely match the experimental measurements, with a mean-squared error of 3.5858×10^{-3}; all reconstruction parameters are provided in Table S9. We have revised the manuscript to explain this evaluation more clearly.

(Instead, the features are just asserted to be 'membrane-scale'. It's unclear if these are cell structures or ringing artifacts.)

We have replaced the term "membrane-scale features" with the more general term "cellular features" in the revised manuscript. We interpret the reconstructed RI distributions as cellular structures rather than ringing artifacts based on the three-dimensional morphology shown in the orthogonal views in Fig. 5d and the axial image stack in Fig. 5f. The reconstructed structures do not consistently appear as rings. Instead, their morphology changes continuously across successive axial planes. For example, in Fig. 5f, one cell exhibits a disk-like RI distribution at $z = 79$ μm, a ring-like cross-section at $z = 92$ μm, and a progressively enclosed structure at $z = 100$ μm. The neighboring cells undergo similar morphological transitions at different axial positions because they are located at different heights within the reconstructed volume. The orthogonal views in Fig. 5d further show that these features are connected in three dimensions and form continuous volumetric cellular structures. This physically consistent evolution in both the orthogonal views and the z-stack supports the interpretation that the reconstructed features originate from the cells rather than from isolated ringing artifacts.

(These experimental results are not as well presented as others and are used to justify that images are obtained without the substrate. In my view, these do not add to the clarity of the manuscript.)

The HEK-cell results are noisier than those obtained using a fluorescent substrate because of the lower signal-to-noise ratio of the raw fluorescence measurements. ActinGreen-labeled actin within the cells produces substantially weaker fluorescence than the fluorescent paint used as the substrate. As shown in Fig. 2, low-SNR measurements can degrade RI reconstruction because the optimization may begin to overfit measurement noise. We therefore apply an early-stopping strategy to mitigate this effect. This limitation and the corresponding mitigation strategy are described in the Results section.

The primary purpose of this experiment is to demonstrate that FDT can reconstruct 3D RI without a fluorescent substrate and without transmission-side access when the fluorescence originates from labeled structures within the specimen itself.

The fluorescence therefore provides both the incoherent illumination required for RI reconstruction and spatially localized information about the labeled actin structures. This result establishes a proof of concept that, with relatively minor modifications, a standard two-photon microscope can be used as a reflection-mode multimodal fluorescence–phase imaging platform as well and acquire multimodal fluorescence-phase images.

In summary, the original manuscript was reviewed and produced similar criticisms from multiple reviewers. The new manuscript shows new data and explains a bit more but the central concerns remain that the approach is contorted into a label free description and the accuracy of the reconstructions are not sufficiently characterized.

We thank the reviewer for summarizing the two central concerns. First, we have clarified that FDT is a multimodal fluorescence–phase imaging technique rather than a label-free imaging technique. FDT integrates these two complementary imaging contrasts: two-photon-excited fluorescence serves as an incoherent illumination source for 3D RI reconstruction while also providing spatially localized, molecularly specific information about the specimen, as demonstrated by the HEK cell results in Fig. 5.

Second, we have clarified how reconstruction accuracy is evaluated using accumulated forward-model consistency, a well-established metric in IDT. Because the ground-truth RI distributions of heterogeneous biological specimens are unavailable, reconstruction accuracy is evaluated by comparing hundreds of intensity images predicted from the reconstructed RI with the corresponding experimentally measured images. We validated that accumulated forward-model consistency reflects RI reconstruction accuracy by reconstructing samples with known ground-truth RI values.

A detailed response has been provided above. We have revised the manuscript accordingly and hope that these revisions and clarifications directly address the reviewer's remaining concerns.

References

[1] Xue, Y., Ren, D., Waller, L.: Three-dimensional bi-functional refractive index and fluorescence microscopy (brief). Biomed. Opt. Express **13**(11), 5900–5908 (2022) https://doi.org/10.1364/BOE.456621

[2] Park, Y., Popescu, G., Badizadegan, K., Dasari, R.R., Feld, M.S.: Diffraction phase and fluorescence microscopy. Opt. Express **14**(18), 8263–8268 (2006) https://doi.org/10.1364/OE.14.008263

[3] Kim, K., Park, W.S., Na, S., Kim, S., Kim, T., Heo, W.D., Park, Y.: Correlative three-dimensional fluorescence and refractive index tomography: bridging the gap between molecular specificity and quantitative bioimaging. Biomed. Opt. Express **8**(12), 5688–5697 (2017) https://doi.org/10.1364/BOE.8.005688

[4] Chowdhury, S., Eldridge, W.J., Wax, A., Izatt, J.A.: Structured illumination multimodal 3d-resolved quantitative phase and fluorescence sub-diffraction

microscopy. Biomed. Opt. Express **8**(5), 2496–2518 (2017) https://doi.org/10.1364/BOE.8.002496

[5] Yeh, L.-H., Chowdhury, S., Waller, L.: Computational structured illumination for high-content fluorescence and phase microscopy. Biomed. Opt. Express **10**(4), 1978–1998 (2019) https://doi.org/10.1364/BOE.10.001978

[6] Shaffer, E., Pavillon, N., Depeursinge, C.: Single-shot, simultaneous incoherent and holographic microscopy. Journal of microscopy **245**(1), 49–62 (2012)

[7] Marthy, B., Bénéfice, M., Baffou, G.: Single-shot quantitative phase-fluorescence imaging using cross-grating wavefront microscopy. Scientific Reports **14**(1), 2142 (2024)

[8] Quan, X., Kumar, M., Rajput, S.K., Tamada, Y., Awatsuji, Y., Matoba, O.: Multimodal microscopy: Fast acquisition of quantitative phase and fluorescence imaging in 3d space. IEEE Journal of Selected Topics in Quantum Electronics **27**(4), 1–11 (2021) https://doi.org/10.1109/JSTQE.2020.3038403

[9] Tayal, S., Singh, V., Kaur, T., Singh, N., Mehta, D.S.: Simultaneous fluorescence and quantitative phase imaging of mg63 osteosarcoma cells to monitor morphological changes with time using partially spatially coherent light source. Methods and Applications in Fluorescence **8**(3), 035004 (2020)

[10] Rajput, S.K., Matoba, O., Kumar, M., Quan, X., Awatsuji, Y., Tamada, Y., Tajahuerce, E.: Multi-physical parameter cross-sectional imaging of quantitative phase and fluorescence by integrated multimodal microscopy. IEEE Journal of Selected Topics in Quantum Electronics **27**(4), 1–9 (2021) https://doi.org/10.1109/JSTQE.2021.3064406

[11] Liu, Y., Suo, J., Zhang, Y., Dai, Q.: Single-pixel phase and fluorescence microscope. Opt. Express **26**(25), 32451–32462 (2018) https://doi.org/10.1364/OE.26.032451

[12] Dong, D., Huang, X., Li, L., Mao, H., Mo, Y., Zhang, G., Zhang, Z., Shen, J., Liu, W., Wu, Z., Liu, G., Liu, Y., Yang, H., Gong, Q., Shi, K., Chen, L.: Super-resolution fluorescence-assisted diffraction computational tomography reveals the three-dimensional landscape of the cellular organelle interactome. Light Sci Appl **9**, 11 (2020)

[13] Yaqoob, Z., Yamauchi, T., Choi, W., Fu, D., Dasari, R.R., Feld, M.S.: Single-shot full-field reflection phase microscopy. Opt. Express **19**(8), 7587–7595 (2011) https://doi.org/10.1364/OE.19.007587

[14] Choi, Y., Hosseini, P., Choi, W., Dasari, R.R., So, P.T.C., Yaqoob, Z.: Dynamic speckle illumination wide-field reflection phase microscopy. Opt. Lett. **39**(20), 6062–6065 (2014)

[15] Kandel, M.E., Hu, C., Naseri Kouzehgarani, G., Min, E., Sullivan, K.M., Kong, H., Li, J.M., Robson, D.N., Gillette, M.U., Best-Popescu, C., Popescu, G.: Epi-illumination gradient light interference microscopy for imaging opaque structures. Nat. Commun. **10**(1), 4691 (2019)

[16] Singh, V.R., Yang, Y.A., Yu, H., Kamm, R.D., Yaqoob, Z., So, P.T.: Studying nucleic envelope and plasma membrane mechanics of eukaryotic cells using confocal reflectance interferometric microscopy. Nature communications **10**(1), 3652 (2019)

[17] Ledwig, P., Robles, F.E.: Epi-mode tomographic quantitative phase imaging in thick scattering samples. Biomed. Opt. Express **10**(7), 3605–3621 (2019)

[18] Ledwig, P., Robles, F.E.: Quantitative 3D refractive index tomography of opaque samples in epi-mode. Optica **8**(1), 6–14 (2021)

[19] Kang, S., Zhou, R., Brelen, M., Mak, H.K., Lin, Y., So, P.T., Yaqoob, Z.: Mapping nanoscale topographic features in thick tissues with speckle diffraction tomography. Light: Science & Applications **12**(1), 200 (2023)

[20] Butola, A., Coucheron, D.A., Szafranska, K., Ahmad, A., Mao, H., Tinguely, J.-C., McCourt, P., Senthilkumaran, P., Mehta, D.S., Agarwal, K., Ahluwalia, B.S.: Multimodal on-chip nanoscopy and quantitative phase imaging reveals the nanoscale morphology of liver sinusoidal endothelial cells. Proc. Natl. Acad. Sci. U. S. A. **118**(47), 2115323118 (2021)

[21] Li, T., Zhu, J., Shen, Y., Tian, L.: Reflection-mode diffraction tomography of multiple-scattering samples on a reflective substrate from intensity images. Optica **12**(3), 406–417 (2025) https://doi.org/10.1364/OPTICA.547372

[22] Fridovich-Keil, S., Yu, A., Tancik, M., Chen, Q., Recht, B., Kanazawa, A.: Plenoxels: Radiance fields without neural networks. In: Proceedings of the IEEE/CVF Conference on Computer Vision and Pattern Recognition, pp. 5501–5510 (2022)

[23] Liu, R., Sun, Y., Zhu, J., Tian, L., Kamilov, U.S.: Recovery of continuous 3d refractive index maps from discrete intensity-only measurements using neural fields. Nature Machine Intelligence **4**(9), 781–791 (2022)
