# Supplementary material for: Fluorescence-illuminated diffraction tomography using explicit neural fields
Source: Nat Commun. 2026 Oct 10;17:11519. doi: 10.1038/s41467-026-78326-x (PMC13745583; doi:10.1038/s41467-026-78326-x)
Supplement: Supplementary file 2 [file 41467_2026_78326_MOESM1_ESM.pdf]

Supplementary Information for: Fluorescence-illuminated Diffraction Tomography using Explicit Neural Fields

Renzhi He[1], Yucheng Li[1], Junjie Chen[1], Truc M. Dinh[1], Charlotte R. Stevens[1], Aidan E. Gilchrist[1], Yi Xue[1*]

[1]Department of Biomedical Engineering, University of California, Davis, 451 Health Sciences Dr., Davis, 95616, CA, United States.

*Corresponding author(s). E-mail(s): yxxue@ucdavis.edu;
Contributing authors: cubhe@ucdavis.edu; ycsli@ucdavis.edu; jujchen@ucdavis.edu; tmhdinh@ucdavis.edu; crstevens@ucdavis.edu; gilchrist@ucdavis.edu;

Contents for Supplementary Material

1 Supplementary Fig. 1 3

2 Supplementary Fig. 2 4

3 Supplementary Fig. 3 5

4 Supplementary Fig. 4 7

5 Experimental setup 8
- 5.1 Optical setup used for imaging MDCK cells and 3D cultured bovine myotube . . . 8
- 5.2 Optical setup used for imaging silica beads . . . 8
- 5.3 Measuring the point-spread function (PSF) of two-photon excited fluorescence . . . 9
- 5.4 Sample preparation. . . . 9
- 5.5 Computational Setting . . . 10

5.5.1 Evaluation metrics. 10
5.5.2 Explicit representations used in FDT 10
5.5.3 Scalability and practical limitations 13

6 Evaluation Metrics and Visual Analysis **14**
Table: Quantitative results for self-calibration ablation study 14
Table: Performance metrics for different reconstruction methods (polystyrene beads) 14
Fig.: RI reconstruction of the “UCDavis” pattern with and without self-calibration 15
Fig.: Comparison of reconstructed results using FDT with and without partially coherent masks 19
Table: Performance metrics with/without partially coherent mask (MDCK cells) 20

1 Supplementary Fig. 1

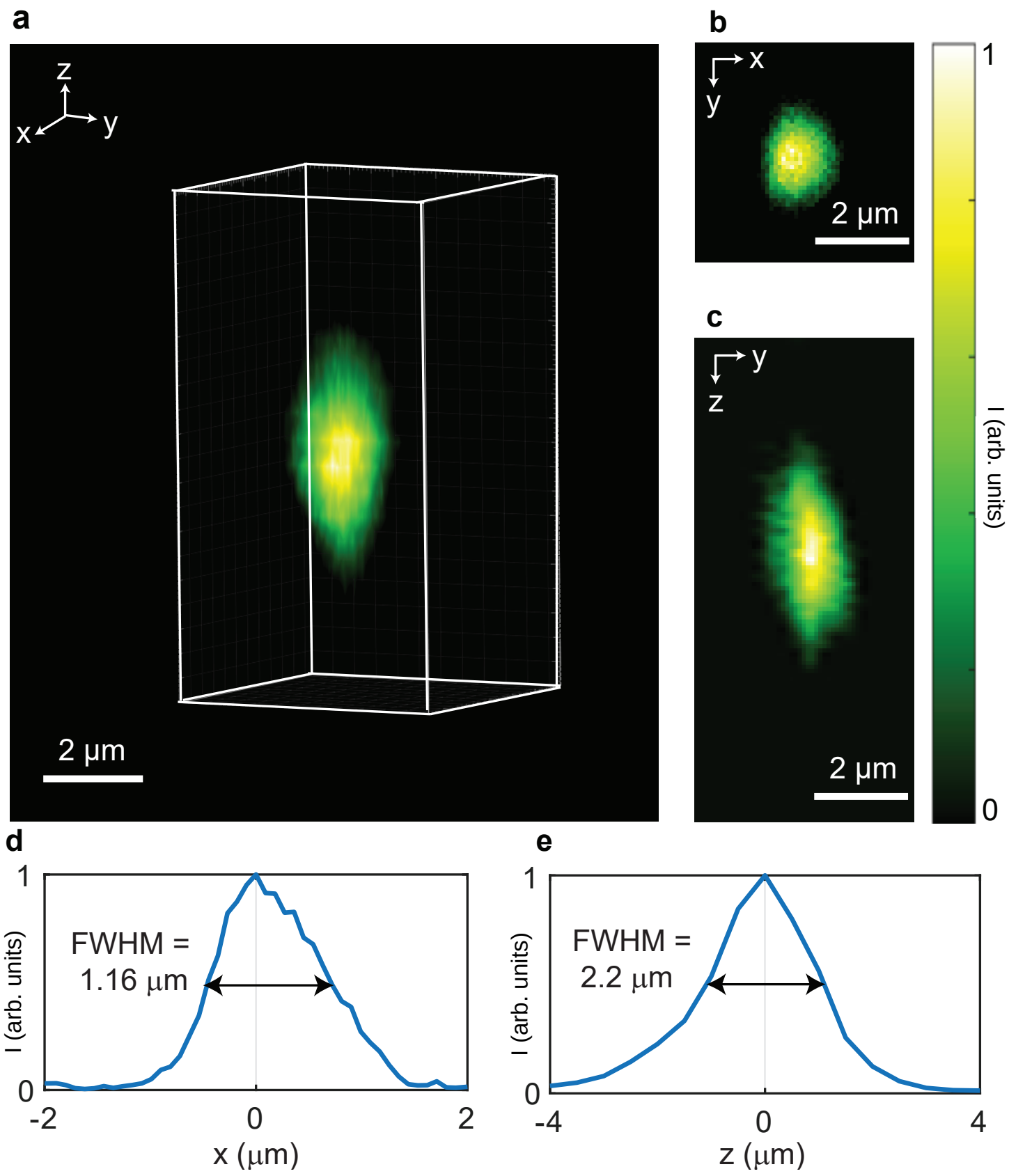

Supplementary Fig. 1 3D point spread function (PSF) of a fluorescence illuminator under two-photon excitation. a. Volume view of the fluorescence PSF. **b-c. (b)** x–y and **(c)** y–z cross-sections of the fluorescence PSF. **d-e.** Line profiles in the **(d)** lateral and **(e)** axial directions, showing the full width at half maximum (FWHM) of the fluorescence PSF.

2 Supplementary Fig. 2

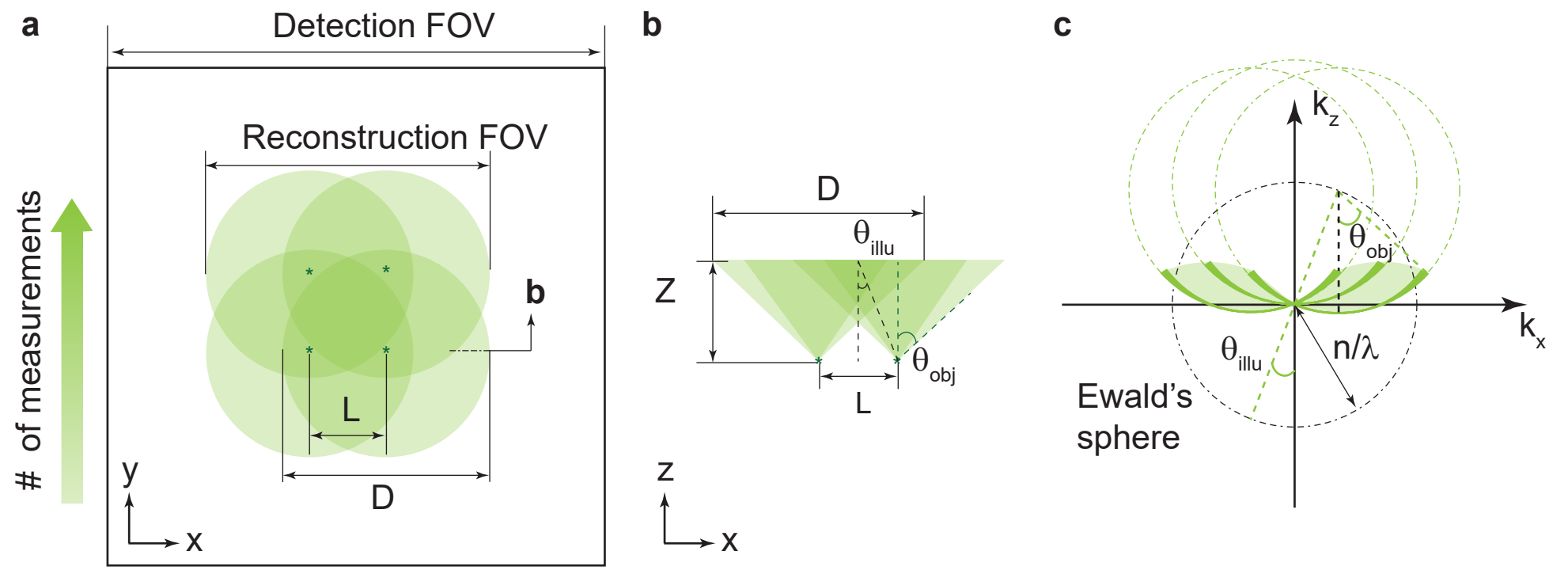

Supplementary Fig. 2 Schematic diagram of the relationships among field of view (FOV), objective and illumination numerical apertures (NA_{obj} and NA_{illu}), optical transfer function (OTF), and the number of measurements. a. The x–y cross-section at the image plane shows the relationship among the detection FOV, reconstruction FOV, the diameter of the fluorescence-illuminated area (D), and the maximum separation between fluorescence sources (L). The central region is naturally sampled more frequently than the peripheral regions. **b.** The x–z cross-section along the fluorescence sources in **(a)** shows the relationship among D, L, the separation between the image plane and the fluorescence sources (Z), the objective lens collection angle (θ_{obj}), and the illumination angle under a fluorescence source (θ_{illu}). **c.** The OTF under three different illumination angles. The spatial-frequency coverage at different illumination angles corresponds to shifts in the OTF. The OTF becomes more completely filled with more measurements, resulting in fewer aliasing artifacts. The resolution is determined by θ_{obj}, θ_{illu}, the wavelength (λ), and the average refractive index (n).

3 Supplementary Fig. 3

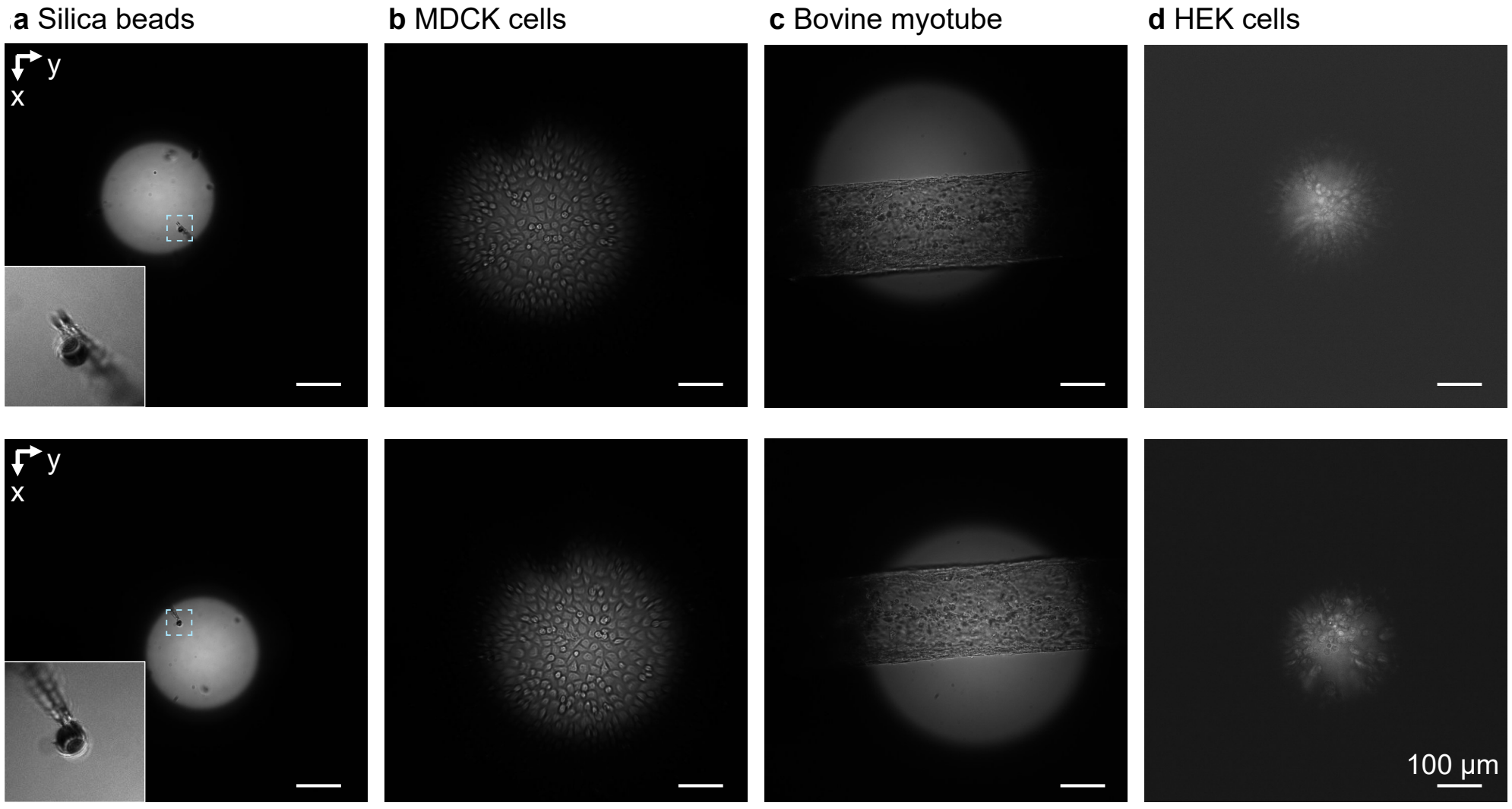

Supplementary Fig. 3 Representative raw diffracted fluorescence images of experimental samples illuminated by two fluorescence sources at different illumination angles. Tens to hundreds of diffracted fluorescence images were collected for each sample, as specified in Table 1. **a.** Silica beads. **b.** MDCK cells. **c.** Bovine myotube. **d.** Fluorescence-labeled HEK cells.

Supplementary Table 1 Imaging parameters used in Figs. 2-5

**Parameters.**	**Fig. 2**	**Fig. 3**	**Fig. 4**	**Fig. 5**
RI sample	Silica beads	Living MDCK cells	3D bovine myotube	3D HEK293T cells
Fluorescence	Paint on the slide	Paint outside the dish	Paint outside the dish	ActinGreen 488 in cells
Exc. (nm)	850	1035	880	900
Emi. (nm)	635 ± 60	635 ± 60	635 ± 60	535 ± 40
NA_{obj}	1.0	1.0	1.0	1.0
NA_{illu}	0.79	0.54	0.44	0.52
Z (μm) [1]	140	190	240	120
Num. of images	419	390	348	282
Time/image (ms)	50	20	100	300
L (μm) [2]	172.85	71.25	56.78	45.97
Recon. volume (μm^3)	$20 \times 20 \times 20$	$264.7 \times 264.7 \times 40$	$530 \times 530 \times 300$	$198 \times 198 \times 70$
Recon. Δx (μm) [3]	0.33	0.33	0.52	0.33
Recon. Δz (μm) [3]	0.5	2	12.5	1.75
RI x resolution (μm) [4]	0.25	0.29	0.32	0.30
RI z resolution (μm) [4]	1.26	1.59	1.73	1.61

1. The distance between the fluorescence source and the imaging plane.
2. The maximum distance between fluorescent sources.
3. These are the sampling size of the reconstructed RI.
4. These are the lower bound of theoretical resolution of RI.

4 Supplementary Fig. 4

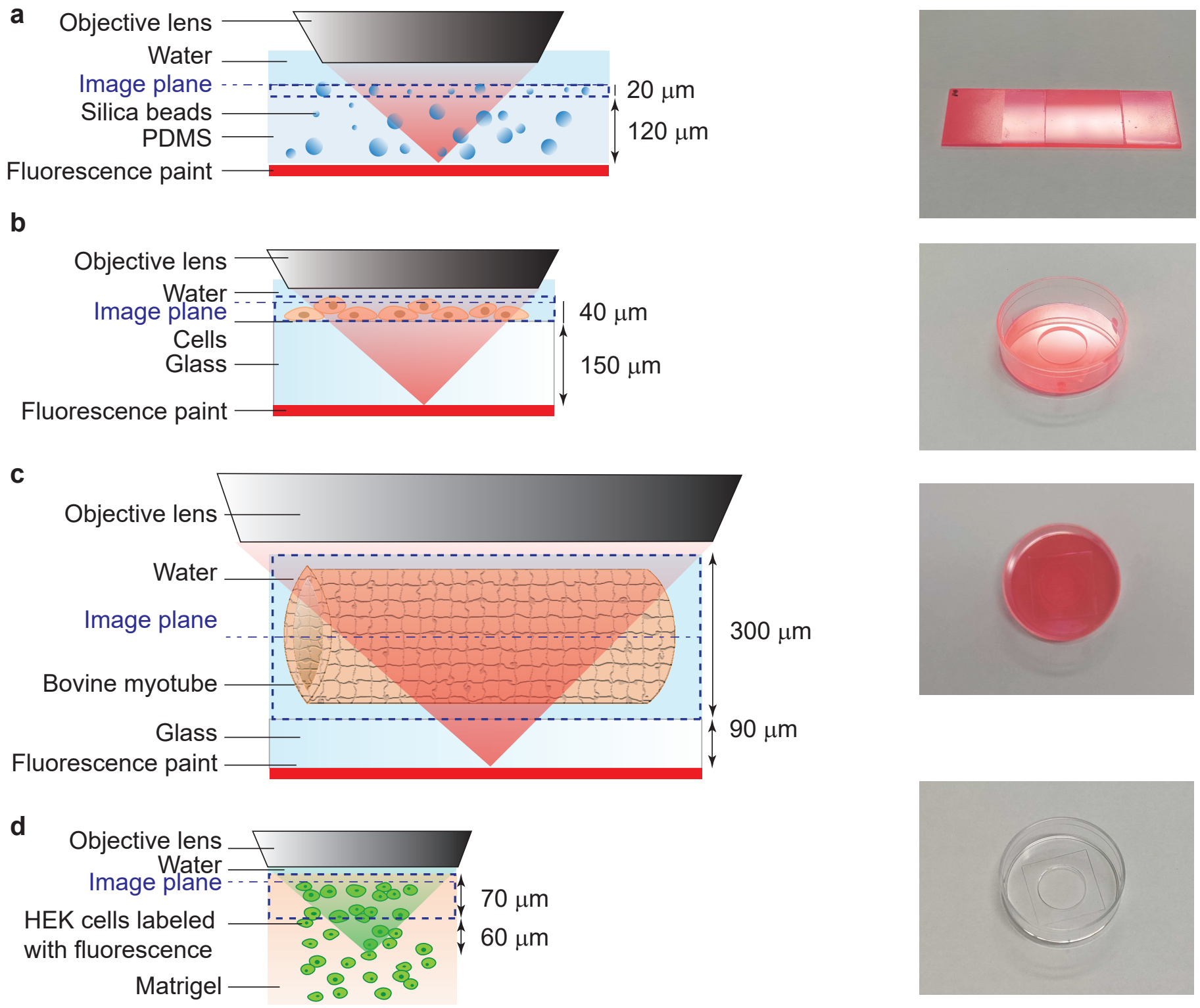

Supplementary Fig. 4 Sample diagram. In the schematic, the gap between the objective lens and the sample is drawn shorter for illustration. In reality, this distance is approximately 2 mm and is set by the working distance of the objective lens. **a.** The sample used in Fig. 2 consists of silica microbeads fixed in PDMS and placed on top of a slide coated with fluorescent paint. A photograph of the sample is shown on the right. **b.** The sample used in Fig. 3 consists of living, label-free MDCK cells cultured in a coverslip-bottom dish. Fluorescent paint is sprayed onto the outside of the dish. A photograph of the fluorescent-paint dish is shown on the right. **c.** The sample used in Fig. 4 consists of label-free bovine myotubes cultured in 3D from muscle stem cells in Matrigel. During imaging, the sample is placed in a coverslip-bottom dish with fluorescent paint on the outer surface. A photograph of the fluorescent-paint dish is shown on the right. **d.** The sample used in Fig. 5 consists of fluorescence-labeled HEK293T cells cultured in 3D in Matrigel. During imaging, cells at a depth of approximately 130 μm are excited to serve as fluorescence sources, while the imaging plane is near the top surface of the sample. Other labeled HEK cells far below the excitation plane and the bottom of the dish are not shown because they are not relevant to the experiment. A photograph of the dish is shown on the right.

5 Experimental setup

5.1 Optical setup used for imaging MDCK cells and 3D cultured bovine myotube

The laser source for FDT is a femtosecond laser at 1035 nm wavelength and 1 MHz repetition rate (Monaco 1035-40-40 LX, Coherent NA Inc., U.S.). A polarizing beam splitter cube (PBS123, Thorlabs) and a half-wave-plate (WPHSM05-1310) mounted on a rotation mount (PRM05, Thorlabs) are used to manually adjust the input power to the following optics in the system. The laser beam is collimated and expanded by a 4-f system (L1, LC1120-C, f_1 = -100 mm, Thorlabs; L2, LA1256-B, f_2 = 300 mm, Thorlabs) before reaching a SLM (HSPDM1K-900-1200-PC8, Meadowlark Optics Inc., U.S.) placed on the Fourier plane. The SLM modulates the phase of the beam to selectively excite fluorophore at any given position in 3D. After the SLM, the laser beam is relayed by two 4-f systems (L3, LB-1889-C, f_3 =250 mm, Thorlabs; L4, AC508-250-C-ML, f_4 = 250 mm, Thorlabs; L5, AC508-200-C-ML, f_5 =200 mm, Thorlabs; L6, AC508-200-C-ML, f_6 = 200 mm, Thorlabs). Additionally, a black spot is painted on a mirror placed at the back focal plane of L3 to block the zero-order beam after the SLM. Next, the modulated laser beam is reflected by a dichroic mirror (FF880-SDI01-T3-35X52, Chroma, U.S.) to the back-aperture of the objective lens (XLUMPLFLN20XW, 20×, NA 1.0, Olympus Inc., Japan). Samples are placed on a manual 3-axis translation stage (MDT616, Thorlabs). In the emission path, a shortpass filter (ET750sp-2p8, CHROMA) is used to block the reflected excitation light. A bandpass filter (AT635/60m, CHROMA) is placed before the camera to pass through red fluorescence. The defocused fluorescence images are captured by a camera (Kinetix22, Teledyne Photometrics, U.S.) controlled by the Micro-Manager software. In addition, a one-photon widefield microscope is implemented, merged with the FDT setup, to locate the sample and find the focal plane before two-photon imaging. The one-photon system consists of an LED (M565L3, Thorlabs), an aspherical condenser lens (ACL25416U-A) to collimate the LED light, and a dichroic mirror (AT600dc, CHROMA) to combine the one-photon path to the two-photon path. The FDT system is controlled by a computer (OptiPlex 5000 Tower, Dell) using MATLAB (MathWorks, U.S.) and a data acquisition card (PCIe-6363, X series DAQ, National Instruments) for signal input/output. Customized MATLAB code is used to generate digital signals, and the NI-DAQ card outputs the signals to synchronously trigger the laser, the SLM, and the camera. For the MDCK cell experiment, the exposure time of each fluorescence image is 20 ms, and the total imaging time to collect 441 fluorescence images is 8.82 s. For the 3D myotube experiment, the exposure time of each fluorescence image is 100 ms, and the total imaging time to collect 441 fluorescence images is 44.1 s.

5.2 Optical setup used for imaging silica beads

The laser source for imaging silica beads is an optical parametric amplifier (Opera-F, Coherent NA Inc.) with a pumped laser at 1035 nm wavelength (Monaco 1035-40-40 LX, Coherent NA Inc., U.S.). The output laser beam wavelength is 880 nm. A polarizing beam splitter cube (PBS122, Thorlabs) and a half-wave-plate (WPHSM05-780)

mounted on a rotation mount (PRM05, Thorlabs) are used to manually adjust the input power to the following optics in the system. A two-axis galvanometer system (GVS002, Thorlabs, Inc., USA) was integrated to implement a traditional point-scanning setup for actively modulating the excitation spot position within a single xy plane. Following the galvanometer, the laser beam is collimated and expanded by a 4-f system (L1, LA1401-B, f_1 = 60 mm, Thorlabs; L2, LA1979-B, f_2 = 200 mm, Thorlabs) before reaching an electrical tunable lens (ETL). The ETL (Optotune, EL-10-30-CI-NIR-LD-MV) is added at the Fourier domain to shift the focal point axially. The laser beam is relayed by two 4-f systems (L3, LA1979-B, f_3 =200 mm, Thorlabs; L4, LA1256-B, f_4 = 300 mm, Thorlabs; L5, AC508-200-C-ML, f_5 =200 mm, Thorlabs; L6, AC508-200-C-ML, f_6 = 200 mm, Thorlabs). Next, the modulated laser beam is reflected by a dichroic mirror (DMSP680B, Thorlabs, Inc.) to the back-aperture of the objective lens (XLUMPLFLN20XW, 20×, NA 1.0, Olympus Inc., Japan). The other parts of the setup, including the sample stage, detection path, and data acquisition parts, are the same as those in the optical setup used for imaging MDCK cells and 3D cultured bovine myotube.

5.3 Measuring the point-spread function (PSF) of two-photon excited fluorescence

We measured the PSF of two-photon excited fluorescence following the standard procedure [1, 2]. We use the optical setup used for imaging silica to measure the PSF, while replacing the camera with a photomultiplier tube (PMT, H15460, Hamamatsu) to detect the in-focus fluorescence. The excitation wavelength is 900 nm. We imaged a fluorescence bead (P7220, ThermoFisher Scientific, ex/em 505/515 nm, 0.175 μm diameter) by scanning the excitation focus in 3D with a step size of 0.09 μm laterally and 0.5 μm axially and exposure time of 1 ms/pixel. Two-photon excited fluorescence is detected through a 535/40 nm color filter (AT535/40m, Chroma). The image stack is generated using MATLAB and processed by ImageJ and Imaris.

5.4 Sample preparation.

Silica microspheres in PDMS. Silica beads (440345-100G, Sigma Aldrich Inc) with PDMS were prepared as a testing phantom. To prepare the polymer matrix, PDMS base and curing agent were mixed at a weight ratio of 10:1. The glass beads, PDMS base, and curing agent were thoroughly combined and degassed for 30 minutes under vacuum to remove air bubbles. The resulting mixture was applied onto a fluorescence-coated glass slide. Fluorescence slides were prepared by uniformly spraying red fluorescent paint onto blank glass slides. A coverslip was gently placed on top of the PDMS mixture, and the entire assembly was cured in an oven at 70 °C for 2 hours.

MDCK cells. MDCK (Madin-Darby Canine Kidney) GII cells were cultured at 37°, 5% CO_2 in DMEM (Gibco) supplemented with 10% fetal bovine serum (FBS-RD Biosciences) and antibiotics. The cell line was checked for mycoplasma and routinely treated with mycoplasma removal agent (MRA) for preventive maintenance. The cells

were plated onto a glass bottom dish (CellVis) pre-coated with rat tail collagen to promote cell adhesion to the substrate.

3D cultured bovine myotube. Bovine muscle stem cells were isolated from freshly slaughtered Angus cow semitendinosus muscle received from the UC Davis Meat Lab by adapting a previously reported protocol [3, 4]. Myotubes differentiated from the stem cells were fabricated employing an advanced Matrigel-agarose core-shell bio-printing technique. The filaments were subsequently cultured in Ham's F10 medium, enriched with 2% fetal bovine serum (FBS) and 1% penicillin-streptomycin (P/S). After 14 days, the well-differentiated bovine myotubes were carefully harvested from the filaments by dismantling the agarose shell.

3D cultured HEK293T cells stained with ActinGreen 488. HEK293T cells were resuspended in 50 μL Matrigel (Corning 354277) on ice at a density of 6.0×10^4 cells/μL. The resuspension was injected into a glass-bottomed mold 8 mm in diameter and 1 mm deep, followed by gelation at 37 °C for 1 hour. After gelation, the samples were then incubated at 37 °C overnight in Dulbecco's Modified Eagle Medium (DMEM; Thermo Fisher Scientific 11995065) supplemented with 10% fetal bovine serum (FBS; Thermo Fisher Scientific 16140071) and 1% Penicillin–Streptomycin (PS; Thermo Fisher Scientific 15140122). Samples cultured in 35-mm dishes were processed for immunocytochemistry as follows. Culture medium was removed and samples were quickly rinsed three times with phosphate-buffered saline (PBS), then fixed in 4% paraformaldehyde in PBS at room temperature for 20 min. After three additional PBS rinses, samples were either stored in PBS at 4 °C or immediately permeabilized with 0.1% Triton X-100 in PBS at room temperature for 45 min, followed by three PBS rinses. Samples were then incubated with ActinGreen 488 ReadyProbe (Thermo Fisher Scientific, R37110) at room temperature for 45 min in the dark. Finally, samples were rinsed three times with PBS, covered with PBS, and stored in the dark until imaging.

5.5 Computational Setting

Our model is trained on an NVIDIA A6000 GPU with 48 GB of Memory. We use the Adam optimizer with an initial learning rate of 5×10^{-3}, a momentum decay rate of 0.9, and a decay rate of 0.99 for squared gradients. The batch size is set to 30.

5.5.1 Evaluation metrics.

The evaluation metrics used in this study include MSE for measuring the average squared differences between predicted and actual values; SSIM for assessing perceptual similarity by considering luminance, contrast, and structure; Learned Perceptual Image Patch Similarity (LPIPS) for evaluating perceptual similarity based on deep features; and Peak Signal-to-Noise Ratio (PSNR) for indicating the peak error between images. These metrics collectively provide a comprehensive evaluation of the model's predictions in terms of both numerical accuracy and perceptual quality.

5.5.2 Explicit representations used in FDT

State-of-the-art methods use implicit neural networks [5] to reconstruct the RI from intensity images, leveraging their ability to represent high-dimensional data. In our

approach, the multi-slice model imposes a strong constraint on the RI. The multi-slice model enforces consistency across multiple 2D slices of the sample and ensures that the RI values are accurately aligned and correlated throughout the 3D volume. This inherent constraint reduces the complexity and ambiguity typically associated with high-dimensional data. Thus, instead of using implicit representations, we use explicit representations [6] to model the 3D RI (Fig. 1). Here, we provide more details about the framework (Fig. 5).

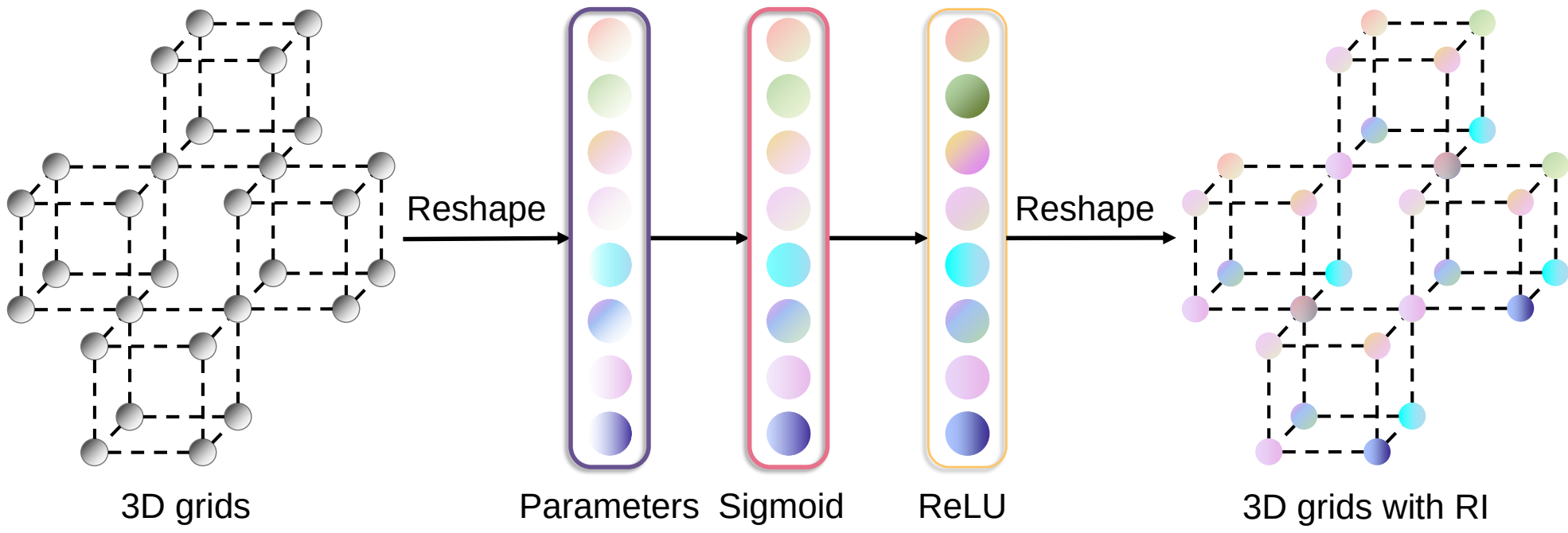

Supplementary Fig. 5 Structure of the explicit representation and neural field of FDT. Initial 3D grids depict interconnected nodes arranged in a spatial structure. The grids are then reshaped and used as parameters of the neural field, which are processed through parameter layers with sigmoid and ReLU activations to map the values to the range of 0 to 1. The resulting RI distribution is subsequently reshaped for use in the multi-slice model.

We first define a sparse voxel grid corresponding to the region of interest in the biological sample. Each voxel is then initialized as a network parameter using the Xavier method, representing the initial estimation of the unknown 3D RI. We then combine the explicit neural field with a coarse-to-fine structure, as described in Section "Model architecture of FDT for RI reconstruction", to adaptively upsample the grid as the number of iterations increases.

The explicit approach omits traditional neural network layers, such as linear or convolutional layers, which significantly accelerates the model's training speed. To prevent large or negative values, we apply a combination of sigmoid and ReLU activation functions to the parameters:

$$\text{Out} = \text{ReLU}(\text{Sigmoid}(x) - 0.5) \tag{1}$$

After this transformation, the parameters are reshaped back into grid format and then fed into the differential multi-slice model.

5.5.3 Scalability and practical limitations

All experiments were conducted on a single NVIDIA RTX A6000 GPU (48 GB memory). No multi-GPU or distributed training was used. The batch size was fixed to 20 in all reported experiments.

Because the RI volume is represented on a voxel-aligned grid, both memory usage and per-iteration computation scale approximately linearly with the number of voxels $N = N_x \times N_y \times N_z$ (for fixed batch size). For example, increasing the lateral number of pixels from 512×512 to 1024×1024 increases the total voxel count by approximately $4\times$, leading to a corresponding increase in runtime and GPU memory usage. Increasing the number of z-planes N_z likewise produces near-linear growth in memory and runtime.

In practice, on a 48 GB RTX A6000, volumes up to $1024 \times 1024 \times 60$ are feasible without gradient checkpointing at batch size 20. On a 24 GB GPU (e.g., RTX A5000), practical configurations are typically around $512 \times 512 \times 40$ under the same batch size. Importantly, the method runs on a single workstation-class GPU and does not require specialized hardware or distributed infrastructure.

Supplementary Table 2 Runtime and peak GPU memory usage per 100 iterations (batch size = 20). Experiments were conducted on a single NVIDIA RTX A6000 (48 GB).

Volume Size ($N_x \times N_y \times N_z$)	Time / 100 iters	Peak Memory
$512 \times 512 \times 20$	5 min	4 GB
$512 \times 512 \times 40$	10 min	7 GB
$512 \times 512 \times 60$	15 min	10 GB
$1024 \times 1024 \times 20$	8 min	17 GB
$1024 \times 1024 \times 40$	17 min	28 GB
$1024 \times 1024 \times 60$	33 min	39 GB

6 Evaluation Metrics and Visual Analysis

Supplementary Table 3 Quantitative metrics for samples with known ground-truth RI).

Sample		MSE	SSIM	LPIPS	PSNR
Simulated UCDavis	images	1.0238×10^{-3}	0.9814	0.0078	29.8980
	RI	1.8381×10^{-3}	0.9830	0.0513	27.3563
Simulated beads	images	1.2630×10^{-5}	0.9995	1.6880×10^{-4}	48.9860
	RI	5.0390×10^{-5}	0.9833	0.1306	42.9762
Experimental beads	images	4.3618×10^{-3}	0.9793	0.0220	27.9854
	RI	1.1503×10^{-3}	0.9860	0.0249	29.3918

Simulated data		MSE↓	SSIM↑	LPIPS↓	PSNR↑
w/o coarse-to-fine	images	1.0238e-03	0.9814	0.0078	29.8980
	RI	1.8381e-03	0.9830	0.0513	27.3563
w/ coarse-to-fine	images	6.1080e-06	0.9990	0.0014	52.1410
	RI	1.5315e-03	0.9838	0.0478	28.1489
w/o self-calibration on parameters	images	2.1896e-03	0.8812	0.0637	26.5964
	RI	2.2373e-02	0.2319	0.8693	16.5027
w/ self-calibration on parameters	images	2.0912e-03	0.9658	0.0088	26.7961
	RI	2.9116e-03	0.8407	0.3664	25.3587
w/o self-calibration on positions ($\sigma = 0.01$)	images	9.9200e-04	0.9581	0.0083	30.0349
	RI	2.4363e-03	0.8157	0.3899	26.1327
w/ self-calibration on positions ($\sigma = 0.01$)	images	5.0604e-04	0.9755	0.0049	32.9582
	RI	1.9631e-03	0.9291	0.1759	27.0706

Supplementary Table 4 Quantitative results from the ablation study on the coarse-to-fine structure and self-calibration using the simulated data "UCDavis". The "w/" and "w/o" coarse-to-fine conditions are evaluated without applying self calibration (i.e., using perfect illumination source positions). For the self-calibration study, random Gaussian noise is added to parameters related to the optical system and illumination positions.

Supplementary Table 5 Quantitative comparison of reconstructed RI accuracy in simulated beads using implicit and explicit neural fields.

Method	MSE	SSIM	LPIPS	PSNR
Implicit	6.1572×10^{-3}	0.8724	0.2363	32.0476
Explicit	5.6387×10^{-4}	0.9834	0.0463	47.5725

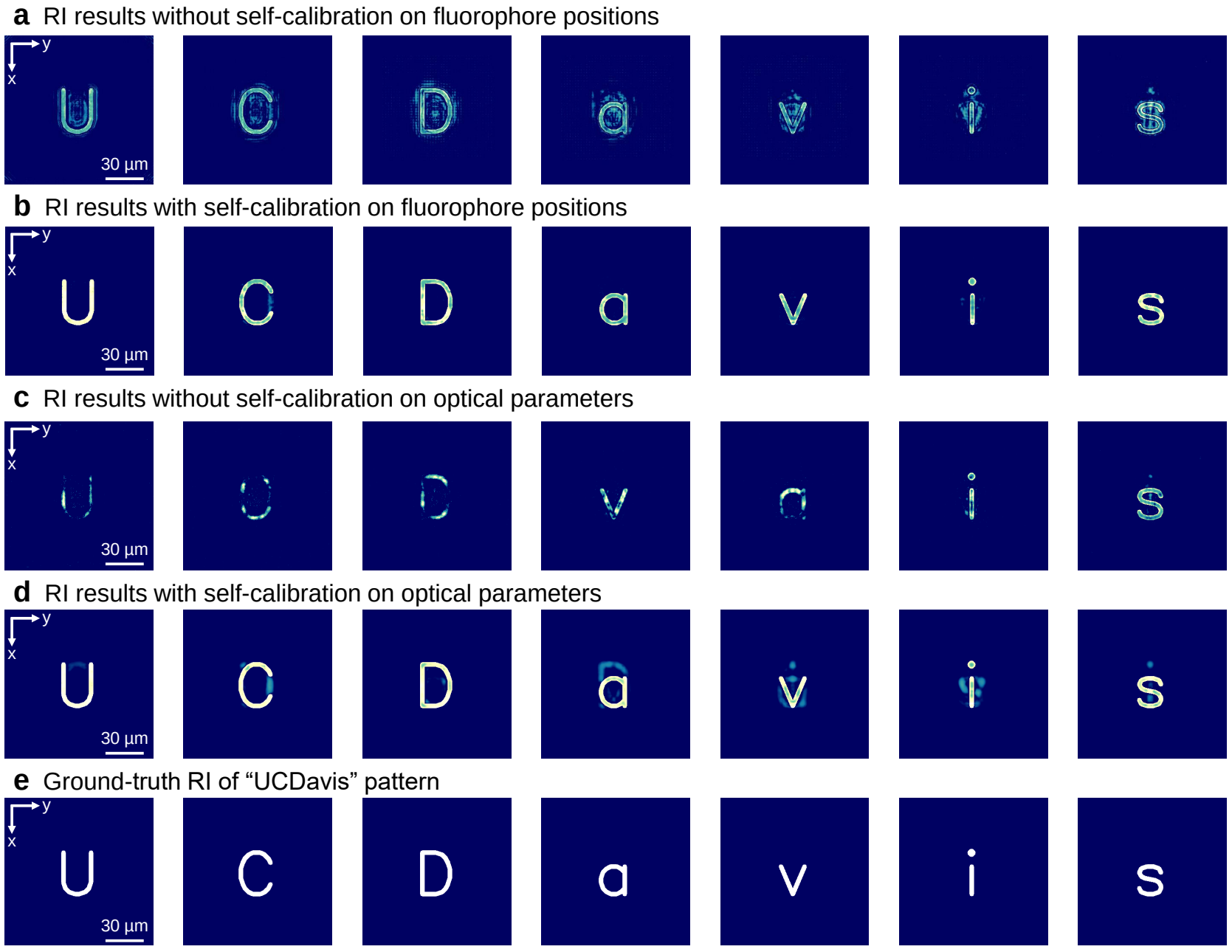

Supplementary Fig. 6 RI reconstruction of the "UCDavis" pattern with and without self-calibration. a. Reconstructed RI without self-calibration on fluorophore positions shows significant distortion and noise. **b**. Reconstructed RI with self-calibration on fluorophore positions displays improved clarity and accuracy of the pattern. **c**. Reconstructed RI without self-calibration on optical parameters (e.g., the resolution of sampling grid) suffers from similar distortions seen in **a**. **d**. Reconstructed RI with self-calibration on optical parameters demonstrates further enhancement, closely matching the expected output. **e**. Ground-truth RI of the "UCDavis" pattern, showing the ideal pattern for comparison.

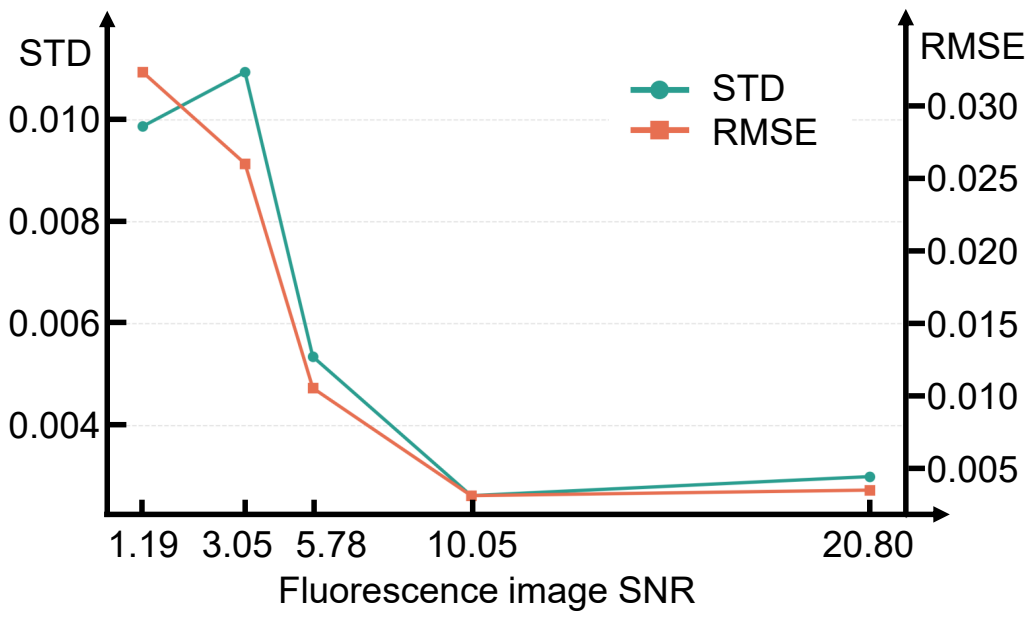

Supplementary Fig. 7 Standard deviation (STD, blue) and root-mean-square-error (RMSE, red) of the reconstructed RI of silica beads in PDMS from experimentally acquired fluorescence images at different SNR levels. For each SNR level, the STD and RMSE were calculated across all pixels within the reconstructed silica bead ($n = 441$ pixels).

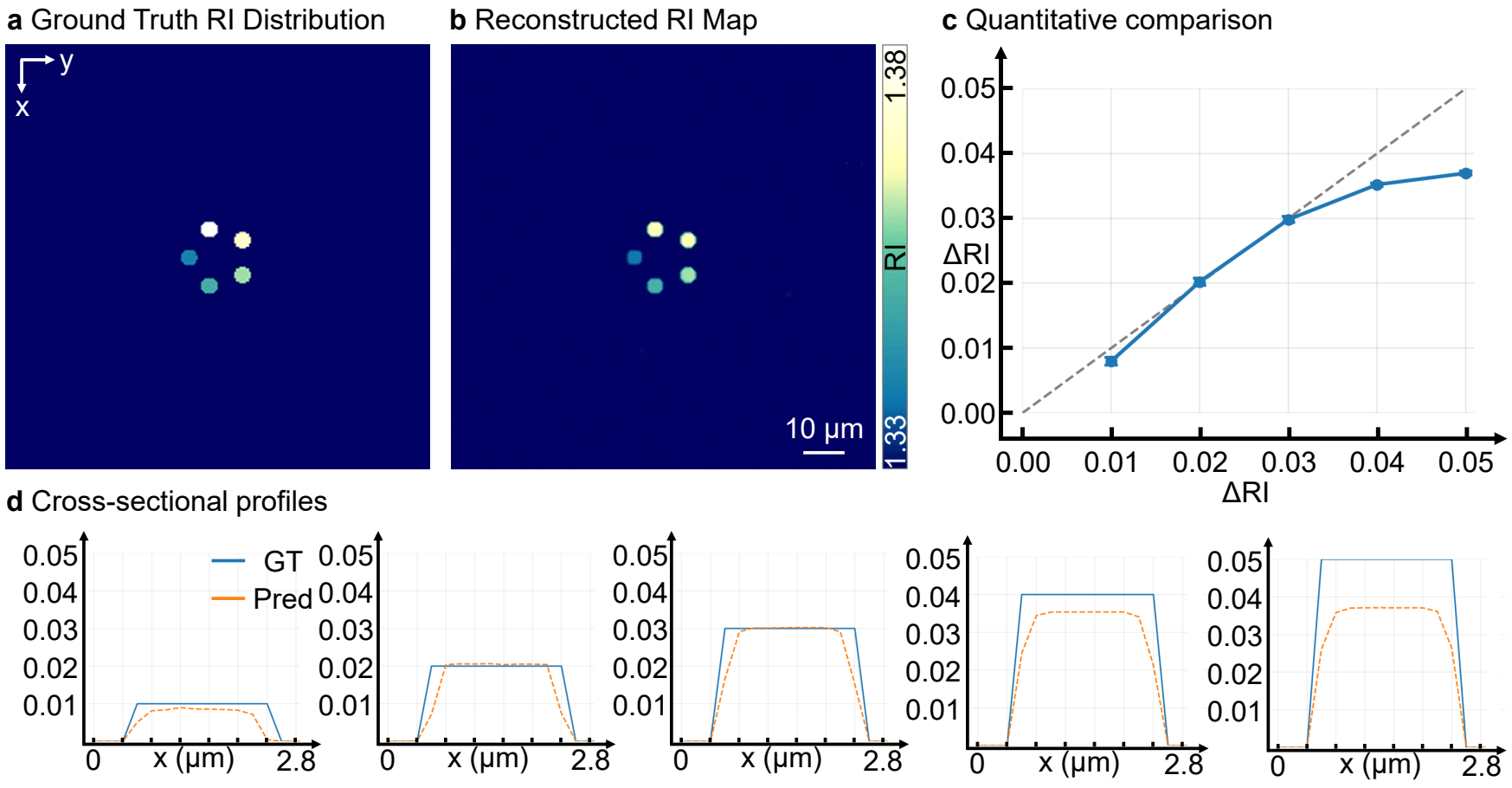

Supplementary Fig. 8 Refractive index reconstruction of beads with varying RI. a. Ground-truth RI distribution of beads with RI contrasts ranging from 0.01 to 0.05. **b**. Reconstructed RI distribution of the simulated beads. **c**. Cross-sectional RI profiles of the beads comparing the reconstructed results with the ground truth.

Image SNR	ΔRI RMSE	Pred. RI Mean ± STD
1.19	1.37×10^{-2}	$1.4442 \pm 4.80 \times 10^{-3}$
3.95	1.07×10^{-2}	$1.4477 \pm 5.32 \times 10^{-3}$
5.78	3.43×10^{-3}	$1.4548 \pm 2.60 \times 10^{-3}$
10.05	1.88×10^{-3}	$1.4584 \pm 1.28 \times 10^{-3}$
20.80	3.41×10^{-3}	$1.4601 \pm 1.46 \times 10^{-3}$

Supplementary Table 6 Statistics of the reconstructed RI of silica beads in PDMS from experimentally acquired fluorescence images at different SNR levels (ground-truth RI = 1.4570). The mean and STD are calculated across the pixels of each reconstructed silica bead ($n = 441$ pixels).

Supplementary Table 7 Quantitative evaluation of reconstructed RI accuracy with simulated beads. The mean and STD are calculated across the pixels of each reconstructed silica bead ($n = 425$ pixels).

Bead	GT ΔRI	Num. of Voxels	ΔRI RMSE	Pred. ΔRI Mean ± STD
Bead 1	0.01	418	2.11×10^{-3}	$0.0080 \pm 5.84 \times 10^{-4}$
Bead 2	0.02	420	5.80×10^{-4}	$0.0202 \pm 5.29 \times 10^{-4}$
Bead 3	0.03	434	4.95×10^{-4}	$0.0298 \pm 4.66 \times 10^{-4}$
Bead 4	0.04	434	4.80×10^{-3}	$0.0352 \pm 2.64 \times 10^{-4}$
Bead 5	0.05	420	1.30×10^{-2}	$0.0370 \pm 2.82 \times 10^{-4}$

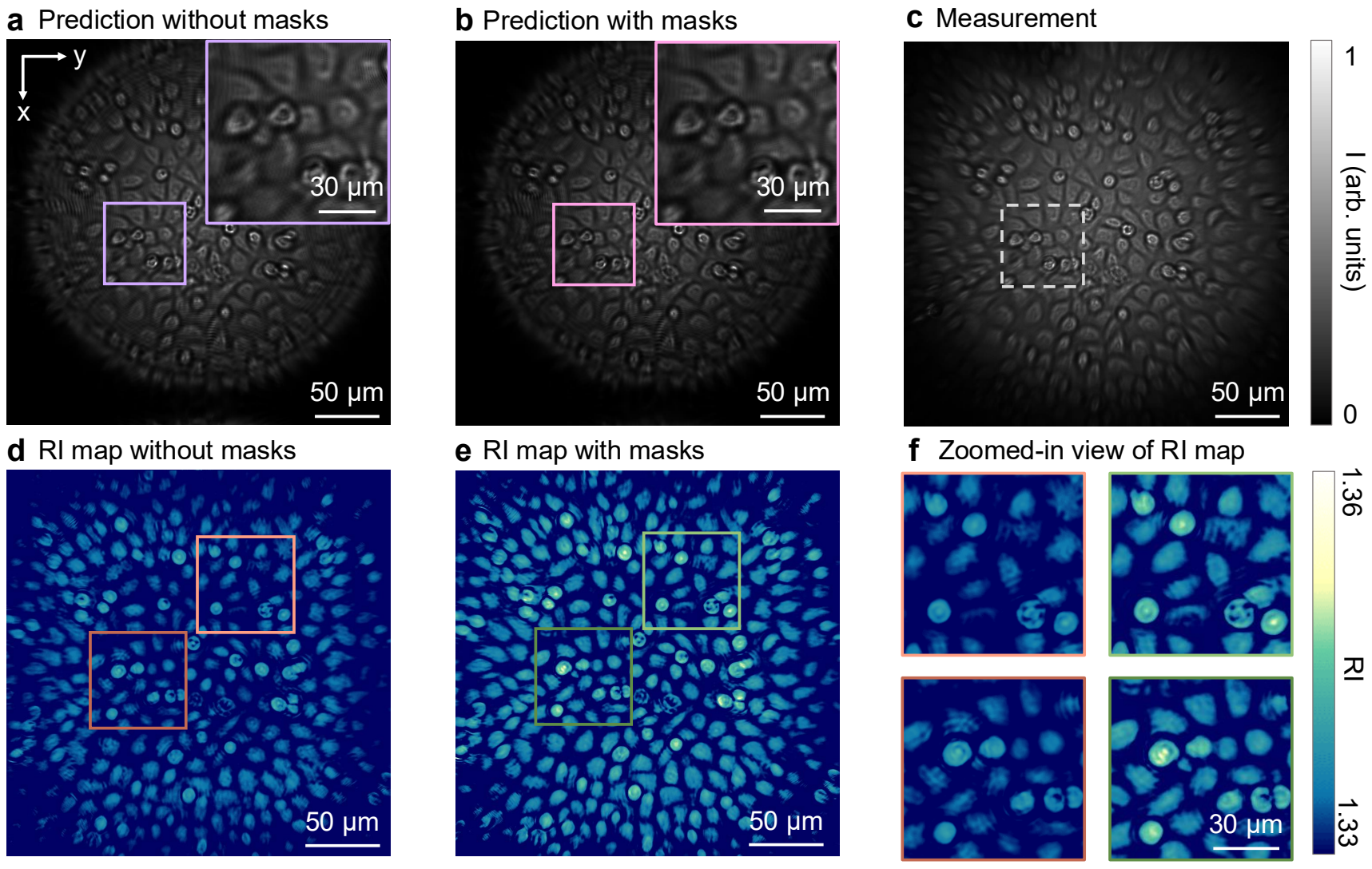

Supplementary Fig. 9 Comparison of reconstructed results using FDT trained with and without partially coherent masks. The reconstruction was performed once on the sample. **a-b**. Predicted image generated using the 3D RI calculated with FDT, trained **a** without and **b** with partially coherent masks. **c**. Experimentally measured image under the same illumination as in **a-b**. **d-e**. Reconstructed RI **d** without and **e** with partially coherent masks. **f**. Zoomed-in view of the reconstructed RI in **d-e**.

Supplementary Table 8 Performance metrics with and without the partially coherent mask for MDCK cell reconstruction.

Method	MSE	SSIM	LPIPS	PSNR
w/o mask	1.3453×10^{-3}	0.8177	0.1776	28.7119
w/ mask	6.2900×10^{-4}	0.9160	0.1417	32.0135

Supplementary Table 9 Forward-model consistency metrics for biological specimens (no ground-truth RI).

Sample	MSE	SSIM	LPIPS	PSNR
MDCK cells	6.2900×10^{-4}	0.9160	0.1417	32.0135
Bovine myotube	7.7843×10^{-3}	0.7361	0.3024	22.7127
HEK cells	3.5858×10^{-3}	0.5126	0.5970	24.6652

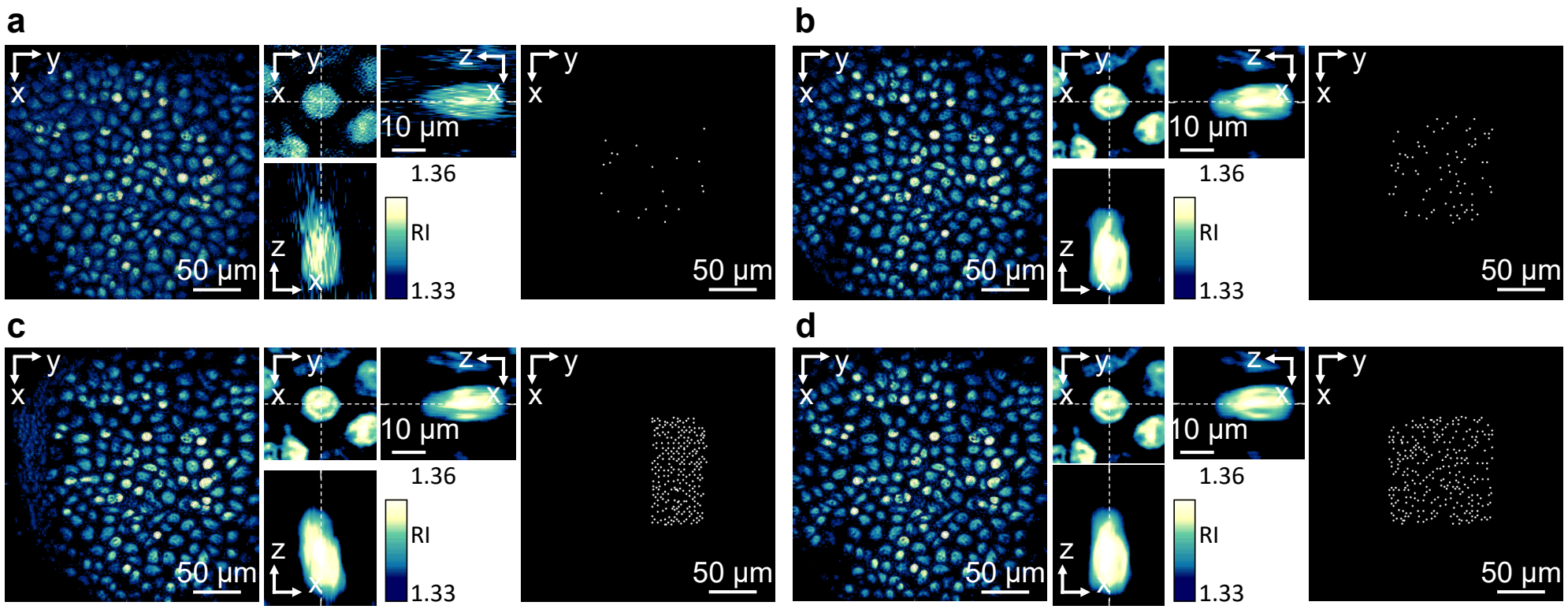

Supplementary Fig. 10 Impact of sampling number and illumination-position coverage on 3D RI reconstruction of MDCK cells, with corresponding illumination-position maps. For each panel, the left image shows the reconstructed x–y slice at $z = 164$ μm (scale bar, 50 μm), the middle insets show orthogonal views (x–y, x–z, and y–z) of a representative cell with the RI color bar, and the right black-dot map shows the distribution of illumination positions used in that strategy. The reconstruction was performed once on the sample. All illumination positions lie in the plane $z = 0$. **a,** Reconstruction using 20 measured images with the corresponding 20 illumination positions, representing sparse sampling with limited coverage. **b,** Reconstruction using 80 measured images with the corresponding 80 illumination positions. **c,** Reconstruction using only measurements from the right half-space with 300 illumination positions, representing one-sided biased coverage. **d,** Reconstruction using 300 measured images with uniform coverage and 300 illumination positions, representing dense and well-covered sampling.

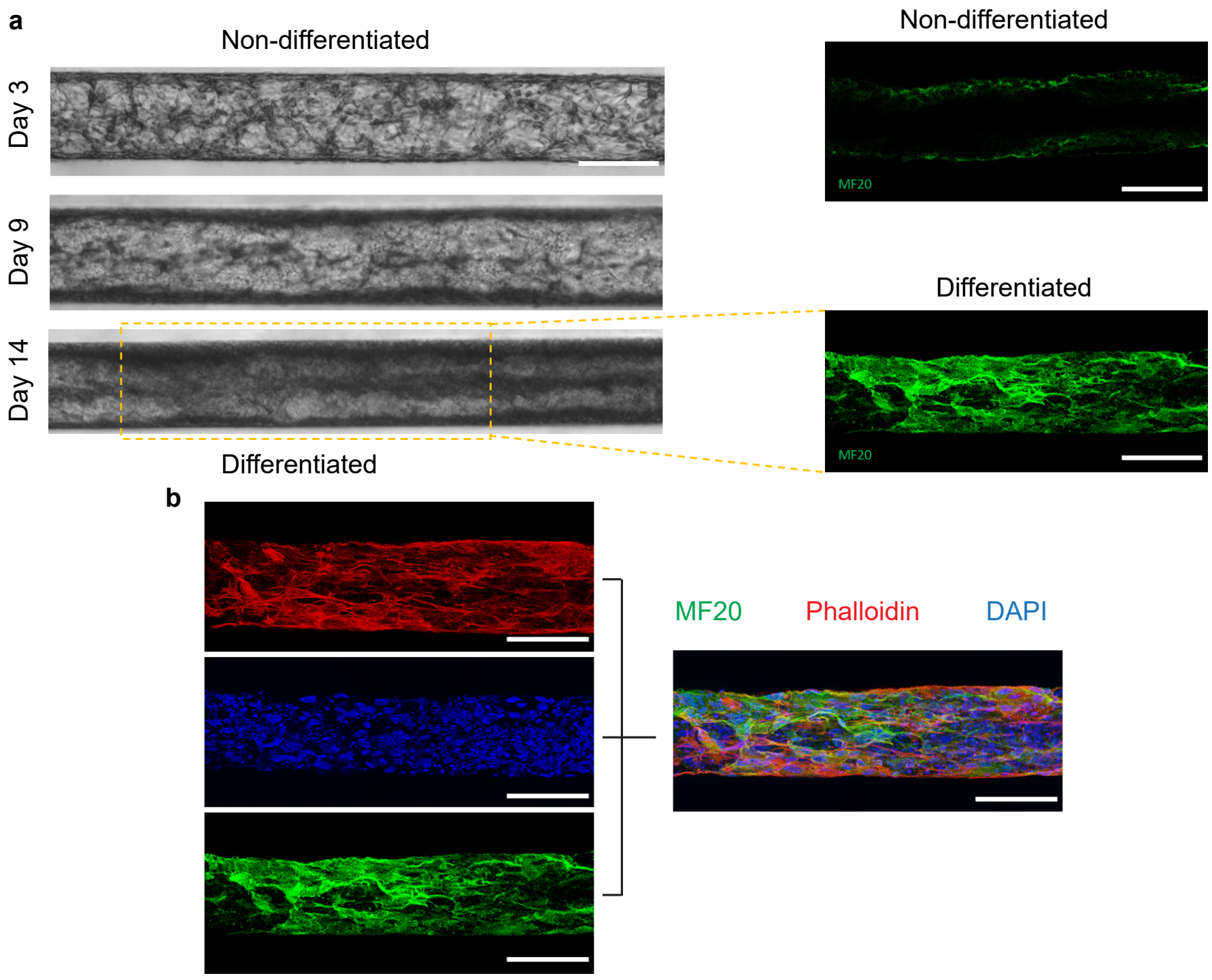

Supplementary Fig. 11 Morphological changes of bovine muscle stem cells are closely linked to their differentiation status. a, The bright-field images show a clear morphological transition of bovine muscle stem cells during differentiation. Before differentiation, cells exhibit a dispersed or loosely aggregated single-cell morphology (top row). In contrast, after differentiation, the cells display elongated, fiber-like structures characteristic of myogenic differentiation (bottom row). This morphological change is evident from the bright-field images across different culture time points. The bright-field images are acquired using a Leica DMi8 microscope with LAS X software (Leica Microsystems). **b,** Confocal images of immunofluorescence staining support successful differentiation at both molecular and structural levels with the differentiated bovine myotube. MF20 (green channel) staining indicates the expression of myosin heavy chain, a marker of terminal myogenic differentiation. In parallel, Phalloidin (red channel) staining highlights the organization of F-actin, revealing aligned and elongated cytoskeletal structures consistent with myotube formation. Together with DAPI nuclear staining (blue channel), these results demonstrate differentiated, fiber-like muscle structures rather than undifferentiated single cells. The confocal images are acquired using a Leica SP8 STED 3X inverted microscope and LAS X software (Leica Microsystems) and analyzed in ImageJ. Scale bar in all images are 100 μm.

References

[1] Zepeda, D., Li, Y., Xue, Y.: Scattering correction through fourier-domain intensity coupling in two-photon microscopy (2P-FOCUS). Photonics Res. **13**(4), 845–864 (2024)

[2] He, R., Li, Y., Urbina, B., Wan, J., Xue, Y.: Compressive fourier-domain intensity coupling (C-FOCUS) enables near-millimeter deep imaging in the intact mouse brain in vivo. arXiv [physics.optics] (2025) [physics.optics]

[3] Li, J., Gonzalez, J., Walker, D., Hersom, M., Ealy, A., Johnson, S.: Evidence of heterogeneity within bovine satellite cells isolated from young and adult animals. Journal of animal science **89**, 1751–7 (2011) https://doi.org/10.2527/jas.2010-3568

[4] Brassard, J.A., Nikolaev, M., Hübscher, T., Hofer, M., Lutolf, M.P.: Recapitulating macro-scale tissue self-organization through organoid bioprinting. Nature Materials **20**(1), 22–29 (2021)

[5] Liu, R., Sun, Y., Zhu, J., Tian, L., Kamilov, U.S.: Recovery of continuous 3d refractive index maps from discrete intensity-only measurements using neural fields. Nature Machine Intelligence **4**(9), 781–791 (2022)

[6] Fridovich-Keil, S., Yu, A., Tancik, M., Chen, Q., Recht, B., Kanazawa, A.: Plenoxels: Radiance fields without neural networks. In: Proceedings of the IEEE/CVF Conference on Computer Vision and Pattern Recognition, pp. 5501–5510 (2022)
