# Supplementary material for: Fluorescence-illuminated diffraction tomography using explicit neural fields
Source: Nat Commun. 2026 Oct 10;17:11519. doi: 10.1038/s41467-026-78326-x (PMC13745583; doi:10.1038/s41467-026-78326-x)
Supplement: Supplementary file 3 [file 41467_2026_78326_MOESM2_ESM.pdf]

Description of Additional Supplementary Files

Supplementary Movie 1. Axial scan through the 3D refractive index of live MDCK cells reconstructed with implicit neural fields, showing high-frequency artefacts and crosstalk between adjacent planes (related to Fig. 3g,h).

Supplementary Movie 2. Three-dimensional rendering of the refractive index of live MDCK cells reconstructed with implicit neural fields (related to Fig. 3g,h).

Supplementary Movie 3. Axial scan through the 3D refractive index of live MDCK cells reconstructed with FDT, resolving individual cells with strong optical sectioning (related to Fig. 3a,d–f,h).

Supplementary Movie 4. Three-dimensional rendering of the refractive index of live MDCK cells reconstructed with FDT (related to Fig. 3a).

Supplementary Movie 5. Axial scan through the 3D refractive index of a 3D-cultured bovine myotube reconstructed with FDT (related to Fig. 4c,d).

Supplementary Movie 6. Three-dimensional rendering of the refractive index of a 3D-cultured bovine myotube reconstructed with FDT (related to Fig. 4a).
