# Supplementary material for: Fluorescence-illuminated diffraction tomography using explicit neural fields
Source: Nat Commun. 2026 Oct 10;17:11519. doi: 10.1038/s41467-026-78326-x (PMC13745583; doi:10.1038/s41467-026-78326-x)
Supplement: Supplementary file 4 [file 41467_2026_78326_MOESM9_ESM.pdf]

nature portfolio

Corresponding author(s): Yi Xue

Last updated by author(s): Aug 17, 2026

Reporting Summary

Nature Portfolio wishes to improve the reproducibility of the work that we publish. This form provides structure for consistency and transparency in reporting. For further information on Nature Portfolio policies, see our Editorial Policies and the Editorial Policy Checklist.

Statistics

For all statistical analyses, confirm that the following items are present in the figure legend, table legend, main text, or Methods section.

n/a	Confirmed	
☐	☒	The exact sample size (*n*) for each experimental group/condition, given as a discrete number and unit of measurement
☐	☒	A statement on whether measurements were taken from distinct samples or whether the same sample was measured repeatedly
☒	☐	The statistical test(s) used AND whether they are one- or two-sided<br>*Only common tests should be described solely by name; describe more complex techniques in the Methods section.*
☒	☐	A description of all covariates tested
☒	☐	A description of any assumptions or corrections, such as tests of normality and adjustment for multiple comparisons
☐	☒	A full description of the statistical parameters including central tendency (e.g. means) or other basic estimates (e.g. regression coefficient) AND variation (e.g. standard deviation) or associated estimates of uncertainty (e.g. confidence intervals)
☒	☐	For null hypothesis testing, the test statistic (e.g. *F*, *t*, *r*) with confidence intervals, effect sizes, degrees of freedom and *P* value noted<br>*Give P values as exact values whenever suitable.*
☒	☐	For Bayesian analysis, information on the choice of priors and Markov chain Monte Carlo settings
☒	☐	For hierarchical and complex designs, identification of the appropriate level for tests and full reporting of outcomes
☒	☐	Estimates of effect sizes (e.g. Cohen's *d*, Pearson's *r*), indicating how they were calculated

Our web collection on statistics for biologists contains articles on many of the points above.

Software and code

Policy information about availability of computer code

Data collection	Image data were collected with customized MATLAB (R2022b) code as described in Methods. The customized code for data collection is uploaded to https://xue-lab-cobi.github.io/fdt_code.
Data analysis	No dedicated statistical software was used. Data processing and visualization were performed in MATLAB R2022b and Python 3.12 as described in the Methods. The customized code for data processing is uploaded to https://xue-lab-cobi.github.io/fdt_code/.

For manuscripts utilizing custom algorithms or software that are central to the research but not yet described in published literature, software must be made available to editors and reviewers. We strongly encourage code deposition in a community repository (e.g. GitHub). See the Nature Portfolio guidelines for submitting code & software for further information.

Data

Policy information about availability of data

All manuscripts must include a data availability statement. This statement should provide the following information, where applicable:

- Accession codes, unique identifiers, or web links for publicly available datasets
- A description of any restrictions on data availability
- For clinical datasets or third party data, please ensure that the statement adheres to our policy

The source data generated in this study are provided in the main text and Supplementary Information and are also available on the project website https://xue-lab-cobi.github.io/fdt/.

Research involving human participants, their data, or biological material

Policy information about studies with human participants or human data. See also policy information about sex, gender (identity/presentation), and sexual orientation and race, ethnicity and racism.

Reporting on sex and gender	N/A
Reporting on race, ethnicity, or other socially relevant groupings	N/A
Population characteristics	N/A
Recruitment	N/A
Ethics oversight	N/A

Note that full information on the approval of the study protocol must also be provided in the manuscript.

Field-specific reporting

Please select the one below that is the best fit for your research. If you are not sure, read the appropriate sections before making your selection.

[x] Life sciences [ ] Behavioural & social sciences [ ] Ecological, evolutionary & environmental sciences

For a reference copy of the document with all sections, see nature.com/documents/nr-reporting-summary-flat.pdf

Life sciences study design

All studies must disclose on these points even when the disclosure is negative.

Sample size	No a priori statistical sample-size calculation was performed because this study was designed to demonstrate and validate an imaging and reconstruction method rather than to test hypotheses based on repeated biological or experimental samples. No a priori statistical sample-size calculation was performed because this study was designed to demonstrate and validate an imaging and reconstruction method rather than to test hypotheses based on repeated biological or experimental samples. For each reconstruction, the number of fluorescence measurements was selected according to the illumination and acquisition requirements of the reconstruction and is reported for the corresponding experiment. For quantitative evaluation, all pixels within each reconstructed object or predicted images were included in the calculation of the mean, standard deviation, and reconstruction error, with the corresponding n values specified in the manuscript.
Data exclusions	Raw acquisition with extreme low SNR is excluded from the reconstruction. No reconstructed results are excluded.
Replication	Reproducibility was assessed through simulations, experiments using samples with known refractive indices, and evaluation of forward-model consistency across various experimental samples using the same imaging and reconstruction procedures. Independent repeat reconstructions were not generally performed, as each reconstruction was intended to demonstrate and validate the imaging and reconstruction performance for the indicated sample rather than to derive statistical conclusions from repeated measurements. In the analysis of RI accuracy, the mean and standard deviation of the reconstructed RI were calculated across all pixels within each bead, with the corresponding sample sizes specified in the manuscript.
Randomization	Randomization was not applicable because the study did not involve allocation of animals or samples into experimental groups.
Blinding	Blinding was not used because there were no experimental or control groups and no subjective scoring or outcome classification.

Reporting for specific materials, systems and methods

We require information from authors about some types of materials, experimental systems and methods used in many studies. Here, indicate whether each material, system or method listed is relevant to your study. If you are not sure if a list item applies to your research, read the appropriate section before selecting a response.

Materials & experimental systems

n/a	Involved in the study	
[x]	[ ]	Antibodies
[ ]	[x]	Eukaryotic cell lines
[x]	[ ]	Palaeontology and archaeology
[x]	[ ]	Animals and other organisms
[x]	[ ]	Clinical data
[x]	[ ]	Dual use research of concern
[x]	[ ]	Plants

Methods

n/a	Involved in the study	
[x]	[ ]	ChIP-seq
[x]	[ ]	Flow cytometry
[x]	[ ]	MRI-based neuroimaging

Eukaryotic cell lines

Policy information about cell lines and Sex and Gender in Research

Cell line source(s)	MDCK (Madin–Darby canine kidney) GII cells (female) were provided by Dr. Soichiro Yamada at the University of California, Davis, who obtained them from the James Nelson laboratory at Stanford University. Primary bovine muscle stem cells used to generate the 3D-cultured myotube samples were provided by Dr. Jiandi Wan at the University of California, Davis. The donor animal was a 15-month-old Angus heifer. Skeletal muscle tissue was collected from the shank post-slaughter at the UC Davis Meat Lab, and the stem cells were isolated in Professor Lucas Smith's laboratory on July 25, 2023 (sample ID: ISO10 7252023). HEK293T cells were provided by Dr. Aidan Gilchrist at the University of California, Davis; these cells were purchased from ATCC (cat. no. CRL-3216).
Authentication	The MDCK and HEK293T cells were not independently authenticated for this study; their identities were based on records from the laboratories that provided and maintained the cells. The bovine muscle stem cells were primary cells rather than an established cell line; therefore, cell-line authentication was not applicable.
Mycoplasma contamination	The cells were not specifically tested for mycoplasma contamination as part of this study.
Commonly misidentified lines (See ICLAC register)	No commonly misidentified cell lines were used in this study. The primary bovine muscle stem cells were not an established cell line.

Plants

Seed stocks	N/A
Novel plant genotypes	N/A
Authentication	N/A
